# Exhaustive search for epistatic effects on the human methylome

**DOI:** 10.1038/s41598-017-13256-9

**Published:** 2017-10-20

**Authors:** Tobias Egli, Vanja Vukojevic, Thierry Sengstag, Martin Jacquot, Rubén Cabezón, David Coynel, Virginie Freytag, Angela Heck, Christian Vogler, Dominique J.-F. de Quervain, Andreas Papassotiropoulos, Annette Milnik

**Affiliations:** 1 Division of Molecular Neuroscience, Department of Psychology, University of Basel, CH-4055 Basel, Switzerland; 2 Transfaculty Research Platform Molecular and Cognitive Neurosciences, University of Basel, CH-4055 Basel, Switzerland; 3 Psychiatric University Clinics, University of Basel, CH-4055 Basel, Switzerland; 4 Department Biozentrum, Life Sciences Training Facility, University of Basel, CH-4056 Basel, Switzerland; 5 Division of Cognitive Neuroscience, Department of Psychology, University of Basel, CH-4055 Basel, Switzerland; 6 sciCORE, Scientific Computing Center, University of Basel, CH-4056 Basel, Switzerland; 7 SIB - Swiss Institute of Bioinformatics, CH-1015 Lausanne, Switzerland

## Abstract

Studies assessing the existence and magnitude of epistatic effects on complex human traits provide inconclusive results. The study of such effects is complicated by considerable increase in computational burden, model complexity, and model uncertainty, which in concert decrease model stability. An additional source introducing significant uncertainty with regard to the detection of robust epistasis is the biological distance between the genetic variation and the trait under study. Here we studied CpG methylation, a genetically complex molecular trait that is particularly close to genomic variation, and performed an exhaustive search for two-locus epistatic effects on the CpG-methylation signal in two cohorts of healthy young subjects. We detected robust epistatic effects for a small number of CpGs (*N* = 404). Our results indicate that epistatic effects explain only a minor part of variation in DNA-CpG methylation. Interestingly, these CpGs were more likely to be associated with gene-expression of nearby genes, as also shown by their overrepresentation in DNase I hypersensitivity sites and underrepresentation in CpG islands. Finally, gene ontology analysis showed a significant enrichment of these CpGs in pathways related to HPV-infection and cancer.

## Introduction

Statistical epistasis describes a higher-order dependency in which the effect of a single-locus genotype depends on the genotype at another locus, a phenomenon also called statistical interaction^1^. There is hitherto little evidence for robust and replicable epistatic effects on complex human traits^1^, although epistasis is often used as a potential explanation for missing heritability or for the instability of main effects in genetic association studies^2–4^. It has been suggested that higher-order dependencies will explain only a minor part of complex phenotypic variation, in comparison to independent additive genetic effects^5,6^.

Genetic variation (e.g. single nucleotide polymorphisms, SNPs) and DNA-CpG methylation can be investigated at high resolution and throughput, which allows a hypothesis-free and exhaustive screening for epistatic effects on a genome-wide scale. However, the study of such epistatic effects is complicated by considerable increase in computational burden^7,8^, model complexity, and model uncertainty, which in concert decrease model stability^9–11^. The success of these association analyses crucially relies on the expected effect size, a suitable sample size and the availability of a well-matched replication study^4,7^.

For the majority of complex human traits, such as neuropsychiatric diseases, only small effect sizes can be expected for single genomic loci^12–15^. Hence, such complex human traits and diseases^16^ are not well amenable to screening for epistatic effects. Gene expression also represents a genetically complex trait, however it is functionally closely related to the DNA sequence variation^17^. Two-locus epistatic effects have been reported for gene expression^1,18,19^, although part of these effects might be due to spurious associations mediated by main effects of SNPs^20^.

To further investigate the relevance of two-locus epistatic effects on complex human traits, we focused on the epigenome, a complex molecular phenotype under close genetic control^21–24^, and here more specifically on DNA-CpG methylation, measured with the Infinium HumanMethylation450 BeadChip Kit (Illumina 450 K), with DNA derived from blood in two independent samples. We performed an exhaustive search of epistatic effects by applying several screening and replication steps and subsequent model-confirmation. Additionally, we performed a search for epistatic effects based on SNPs proximal to CpGs, which are exhibiting main effects.

## Results

**Figure 1:**
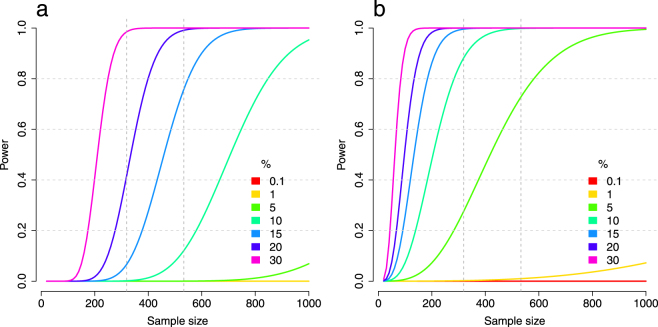
Power analysis exhaustive search for epistatic effects. In **(a)** we adjusted alpha to reach genome-wide and methylome-wide Bonferroni correction (discovery phase, *p* = 6.8 × 10^−18^). In **(b)** we adjusted alpha to reach a per-CpG Bonferroni correction threshold (replication phase, *p* = 3.8 × 10^−6^). The legends depict the variance that can be explained (in percentage) for different effect sizes (*r*_*min*_ = 0.03, 0.1%; *r*_*max*_ = 0.55, 30%). The vertical gray bars correspond to a sample size of *N* = 533 (discovery sample) and *N* = 319 (replication sample).

We used data from two independent samples of healthy young adults from the general population (discovery sample *N* = 533, replication sample *N* = 319; see Table 1; DNA was derived from blood, see Methods)^25^. With these sample-sizes we were adequately powered to detect and replicate medium to strong effect sizes in an exhaustive search (see Fig. 1). Both samples have a comparable genetic (see Supplementary Fig. S1) and phenotypic background (see Table 1). SNP-data (*N* = 517,504 SNPs) as well as CpG-data (*N* = 395,431 CpGs; see Supplementary Figs S2 and S3 for diagnostic plots of the data) was selected to reach high quality metrics in both samples (see Methods).

**Table 1:** Sample description.

	Discovery sample		Replication sample	
	All subjects data freeze 2013-08	Selected subjects	All subjects data freeze 2014-04	Selected subjects
Sample size N	1174	533	1935	319
Sex female	59.8%	58.3%	66.1%	69.6%
Blood sampled	63.7%	100%	36.1%	100%
Affymetrix 6.0 data	84.3%	100%	89.9%	100%
Genetic outlier	6.5%	0%	7.8%	0%
Age at main investigation	22 (18–35)	22 (18–35)	23 (18–35)	23 (18–35)
Age at blood sampling	23 (18–36)	23 (18–36)	24 (18–39)	24 (18–36)
Days between main investigation and blood drawing	336 (1–1392)	350 (2–1385)	642 (1–1992)	380 (1–954)
Smoking behavior at main investigation	1.6 (1–5)	1.6 (1–5)	1.8 (1–5)	1.7 (1–5)

Phenotypic information was collected at the time-point of the main investigation (see Methods). Subjects were later re-invited for an additional blood sampling to investigate e.g. blood-related methylation and expression values. Reported are the numbers from the data freezes used to select subjects for the blood-DNA-methylation study (discovery sample 2013-08; replication sample 2014-04). Quantitative variables are reported as mean (min - max). Smoking behavior was measured on a 5-point Likert-scale ranging from 1 (never) up to 5 (20 cigarettes per day).

### Discovery phase of the exhaustive search

In the discovery sample, we applied a two-step approach to first identify and then confirm interaction effects. First, based on a pruned set of SNPs (*N* = 192,955 SNPs) with sufficient minor allele frequency (see Methods), we performed an exhaustive screening (*N* = 7.36 × 10^15^ interaction analyses) with EpiGPU^26^. EpiGPU is optimized for high-speed genome-wide interaction analyses, but uses a simplified parameterization of the interaction term (see Methods). We filtered SNP-pairs that fulfill basic criteria with respect to the number of groups (all 3 × 3 genotype combinations existing) and estimated *F*-statistic of their interaction term (*F*_*int*_ > 22, which was close to Bonferroni correction). Analyses that survived this filtering step (*N* = 9.54 × 10^9^) entered a recalculation phase, in which we reproduced the interaction *F*-statistic with a linear model approach featuring an increased accuracy at the cost of a higher computational burden. Only analyses that survived genome-wide Bonferroni-correction (*p*_*int*_ < 6.8 × 10^−18^, correcting for all 7.36 × 10^15^ initial interaction analyses) after recalculation and in which the minimal group size was above 3 in both the discovery and replication sample entered the replication phase (*N* = 8,608,567 analyses; *N* = 13,112 unique CpGs; see Table 2). To rule out that the above described two-step procedure is overly conservative, we additionally applied the Bonferroni-correction directly to the *p*-value derived from the original EpiGPU analysis, which resulted in a nearly identical outcome: *N* = 8,658,122 analyses survived based on *N* = 13,214 unique CpGs.

**Table 2:** Main results exhaustive search for epistatic effects.

	*N* unique CpGs	Average *N* hits per CpG	Max *N* hits per CpG	*N* hits in total	Both SNPs in *cis*	One SNP in *cis* and in *trans*	Both SNPs in *trans*
Before replication	13,112	657	46,314	8,608,567	0.03%	0.20%	99.78%
After replication	1,477	3	131	4,816	43.60%	10.36%	46.03%
+Permutation and sign-test	802	3	49	2,262	90.45%	3.98%	5.57%
Per-CpG model approach	174	1	5	239	88.28%	4.18%	7.53%
+Exclusion of LD-block associated effects	47	1	3	55	63.64%	18.18%	18.18%

The results shown refer to significant interaction effects, depending on the different analytical steps. *cis* is defined as 500 KB around the CpG.

### Replication phase of the exhaustive search

Replication was also done in two steps. Firstly, we selected interaction effects that survived a per-CpG Bonferroni-correction in the replication sample (i.e. correcting for at least 13,112 tests; see Methods*; p*_*int*_ < 3.8 × 10^−6^; *N* = 4,816 analyses survived). Because of the unequal distribution of subjects in the 9 combined genotype-groups, assignment of few subjects with phenotypes from an extreme end of the distribution to one cell may lead to false-positive findings. Hence, we additionally applied a per-CpG permutation approach to confirm the significance of these findings with an empirical *p*-value (same per-CpG *p*-value threshold with empirical *p*_*int*_ < 3.8 × 10^−6^; 51% of analyses discarded), as well as a sign-test to filter out results that show inconsistent directions of effect between samples (22% of analyses discarded; see Supplementary Figure S4). 2,262 analyses (47%), based on 802 unique CpGs survived both steps (see Table 2; see Methods for a comparison between the above described procedure with a Bonferroni correction for all tests performed).

### Per-CpG modeling

**Figure 2:**
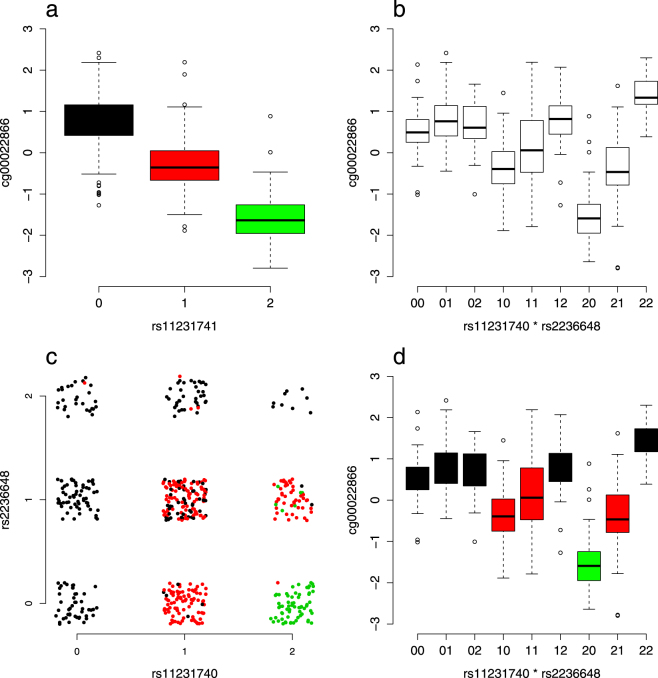
Example of a main effect of a SNP causing a spurious significant interaction effect between two other SNPs. Data is shown for cg00022866 from the discovery sample. **(a)** rs11231741 shows a strong main effect (*p* = 4.5 × 10^−112^). This causes a spurious significant interaction (**b** *p* = 3.3 × 10^−18^) because rs11231741 is in LD with both interacting SNPs (rs11231740: *r* ^2^ = 0.55; rs2236648: *r* ^2^ = 0.25). Of note, the two interacting SNPs show low LD only (*r* ^2^ = 0.024). Panel **(c)** depicts the dependencies between the 9 SNP-groups build from rs11231740 and rs2236648 and the 3 SNP-groups from rs11231741 (color-coded in black, red and green; a jitter has been added to the data): the 9 SNP-groups of the interacting SNPs mimic the three SNP-groups of the main effect, with 5 of the 9 groups mainly corresponding to the homozygous common allele carrier (black), 3 of the 9 groups mainly corresponding to the heterozygous group (red) and 1 group mainly corresponding to the homozygous rare allele carrier (green). Panel **(d)** shows the same data as in **(b)**, but now with color-coding of the three SNP-groups from rs11231741.

Importantly, two SNPs in linkage disequilibrium (LD) with a third SNP that shows a main effect on the trait of interest might, under certain circumstances, mimic an interaction effect that is in fact fully attributable to the main effect^20,27^ (see Fig. 2 for one example). Therefore, in the next validation step of epistatic effects, we aimed at simultaneously taking into account main effects as well as interaction effects by generating one comprehensive model of SNP-effects for each single CpG. For this analysis we used the entire SNP-set yielding a higher resolution compared to the smaller SNP-set (*N* = 517,504 SNPs instead of *N* = 192,955 SNPs). We applied a stepwise-forward regression approach, starting with main effects before adding interaction effects. For *N* = 174 CpGs at least one significant interaction effect was detectable (239 SNP-pairs in total; see Table 2) in both samples, when also taking into account significant main effects of SNPs (see Supplementary Table S1 and Supplementary Fig. S6 for the full models).

Estimation of epistatic effects can be biased for non-independent genomic loci^27,28^. Therefore, we calculated the LD for the SNP-pairs showing interaction effects. We empirically determined the threshold of LD that was unlikely to occur by chance in the discovery sample (*r* ^2^ > 0.021, *p* < 0.001, see Supplementary Fig. S5). 184 out of the 239 SNP-pairs (77%) showed an *r* ^2^ > 0.021 in the discovery sample; these pairs were all located in close proximity (mean = 53 KB, min = 0.5 KB, max = 444 KB) to each other on the same chromosome. Hence, we classified these effects as LD-block associated effects^1,29^. After exclusion of LD-block associated effects 47 CpGs (based on 55 SNP-pairs) still showed at least one significant epistatic effect (see Table 2). For the remaining 127 CpGs we identified only LD-block associated effects.

When considering all 174 CpGs, significant main effects explained on average 57% of variance from the CpG signals whereas interaction effects additionally explained on average 8% of variance (see Table 3).

**Table 3:** Exhaustive search average variance explained by main effects and interaction effects.

	Discovery sample average variance explained	Replication sample average variance explained
**All significant main effects**	57.1%	57.8%
- Most-significant main effect	44.9%	45.4%
**All significant interaction effects**	8.2%	8.3%
- Most-significant LD-block associated effect	4.4%	4.5%
- Most-significant epistatic effects	7.8%	7.4%
**All significant main effects and interaction effects**	65.2%	66%

The results are based on the *N* = 174 CpGs that showed at least one significant interaction effect when taking into account also main effects. For only 7 out of 174 CpGs (4%) no significant main effect of a SNP was detectable. 12 out of 174 CpGs (6.9%) showed both, a LD-block based effect as well as an epistatic effect. All significant main effects: average variance explained by all main effects that were kept in the final model. Most-significant main effect: average variance explained by the main effect that exhibited the smallest *p*-value. All significant interaction effects: average variance explained by all interaction effects that were kept in the final model; these were further separated in LD-block associated effects with SNP-pairs showing an *r* ^2^ > 0.021, or epistatic effects (*r* ^2^ ≤ 0.021), most-significant corresponds to the effect with the smallest *p*-value, if more than one of these effects were kept in the final model. All significant main effects and interaction effects: average variance explained by all main effects and interaction effects that were kept in the final model.

### Search for epistatic effects based on SNPs exhibiting main effects

We performed an additional search for interaction effects, based on SNPs in proximity to the CpG (defined as 3.5 MB window, number of SNPs per CpG ranging from 1 to 3,290, mean = 1,223) that show a significant main effect. The 3.5 MB window comprises >95% of all main effects from SNPs on CpGs^25^. By focusing on SNPs being located in proximity to CpGs and exhibiting main effects, this approach was computationally less expensive (*N* = 4.84 × 10^8^ main effects and *N* = 7,173,795 interaction effects tested) in comparison to the exhaustive search (*N* = 7.36 × 10^15^ interaction analyses).

We first applied Bonferroni-correction per CpG in the discovery sample (*p*-value ranging from 0.05 to 1.5 × 10^−5^) to identify significant main effects and then tested all significant findings in the replication sample. After replication, *N* = 59,134 CpGs showed at least one significant main effect of a SNP in the 3.5 MB window (mean = 9, max = 508). The observation that ~15% of CpGs were associated with a genetic marker is in agreement with previous results^24,30^, considering differences in sample-sizes between studies and hence differences in power to detect such effects.

Out of these 59,134 CpGs, *N* = 48,293 CpGs showed more than one significant main effect of a SNP. For these CpGs, we identified all significant interaction effects between SNPs that show a significant main effect (see Methods). *N* = 24,892 interaction effects based on *N* = 3,564 CpGs survived this step (number of interaction effects per CpG: mean = 7, max = 327). Based on these results, we again performed a forward regression approach for each CpG. We included all identified main effects and interaction effects in this analysis, testing main effects first. In the forward regression approach *N* = 364 interaction effects remained significant, based on *N* = 281 unique CpGs (number of interaction effects per CpG: mean = 1, max = 6; see Supplementary Table S2 for the full models). *N* = 255 SNP-pairs (70%) revealed an *r* ^2^ > 0.021, and were classified as LD-block associated effects. After exclusion of LD-block associated effects, *N* = 91 CpGs showed at least one epistatic effect. The remaining *N* = 190 CpGs showed LD-block associated effects only. We determined the amount of variance that can be explained by single-SNPs, by all significant main effects of SNPs and by full models that include also interaction effects when applying the forward regression approach (see Table 4). The overall variance explained and the variance explained by single-SNP hits systematically increased with increased model complexity.

**Table 4:** Search for epistatic effects based on SNPs exhibiting main-effects.

Per-CpG model in 3.5 MB window	N unique CpGs	Discovery sample Average variance explained by SNPs			Replication sample Average variance explained by SNPs		
		Most-signif. main effect	All signif. main effects	All signif. main effects and interaction effects	Most-signif. main effect	All signif. main effects	All signif. main effects and interaction effects
- CpGs showing at least one significant main effect	59,134	16%	17.7%	17.7%	16%	18.1%	18.1%
- CpGs showing at least two significant main effect	17,938	22.6%	28.2%	28.3%	22.3%	29%	29.1%
- CpGs showing significant interaction effects	281	31.2%	41.9%	46.8%	31.2%	43.3%	49.1%

Average variance explained by main effects of SNPs or interaction effects of SNP-pairs. Results are shown for three different filtering steps, which were based on the number of significant main effects or interaction effects per CpG, identified with a forward-linear regression approach. Most-signif. main effect: average variance explained by the main effect that exhibited the smallest *p*-value. All signif. main effects: average variance explained by all main effects that were kept in the final model. All signif. main effects and interaction effects: average variance explained by all main effects and interaction effects that were kept in the final model. Signif.: significant.

The detection rate for CpGs exhibiting significant interaction effects was comparable for the exhaustive search (*N* = 174, 0.044% of all CpGs analyzed) and the search based on SNPs that show a main effect (*N* = 281, 0.071% of all CpGs analyzed). Taking both approaches together, we identified a total of *N* = 404 CpGs showing interaction effects (0.1% of all CpGs analyzed), with 51 CpGs being identified in both analyses (see Supplementary Table S3).

### Enrichment analyses

The *N* = 404 CpGs showing interaction effects could be assigned to *N* = 350 clusters, when assigning CpGs with an *r* > 0.8 to one cluster (CpGs per cluster: mean = 1.15, max = 5; the maximal base-pair distance per cluster was 4,820). For the following enrichment-analyses (see Table 5), we randomly chose one CpG per cluster. These *N* = 350 CpGs were significantly underrepresented in CpG islands (*p* = 1.1 × 10^−6^) and significantly overrepresented in DNase I hypersensitivity sites (*p* = 0.025). Furthermore, these CpGs showed a strong enrichment in significant associations with gene expression (*p* = 1.2 × 10^−127^), with the top-hit of the transcripts being located in *cis* of the CpG in all cases except two (see Supplementary Table S3). Gene-set enrichment analysis (see Table 6) on those 350 CpGs showed significant association signals (*p*_*FDR*_ < 0.05) for 7 KEGG-pathways, with the top-hits being associated with Human papillomavirus (HPV) infection (hsa05165, *p*_*fdr*_ = 0.0014) and cancer (hsa05200, *p*_*fdr*_ = 0.0036).

**Table 5:** Enrichment analyses.

	Expected	Observed	*p*
CpG Island	32.9%	20.6%	1.1 × 10^−6^
TFBS	63.3%	66.9%	0.19
DNase I	70.4%	76%	0.025
Gene expression	10.8%	50.9%	1.2 × 10^−127^

For *N* = 404 CpGs we identified a significant interaction between SNPs. These 404 CpGs could be assigned to *N* = 350 clusters. For each cluster we randomly assigned one CpG as representative. For these 350 CpGs we compared the observed percentage of being located in CpG-dense regions (CpG Island), transcription factor binding sites (TFBS), DNase I hypersensitivity sites (DNase I) or being associated with gene expression against the expected numbers that are based on all remaining CpGs (*N* = 395,027), by using *Chi* ^2^-tests.

**Table 6:** Results for the gene-set enrichment analysis.

Term	Pathway	*N* genes	*N* hits	*p*_*nominal*_	*p*_*FDR*_
hsa05165	Human papillomavirus infection	303	10	1.7 × 10^−7^	0.0014
hsa05200	Pathways in cancer	384	10	1.1 × 10^−6^	0.0036
hsa05224	Breast cancer	141	7	1.5 × 10^−6^	0.0036
hsa01100	Metabolic pathways	1190	14	1.7 × 10^−6^	0.0036
hsa04014	Ras signaling pathway	217	7	9.9 × 10^−6^	0.017
hsa04151	PI3K-Akt signaling pathway	317	8	1.2 × 10^−5^	0.018
hsa05203	Viral carcinogenesis	191	6	2.9 × 10^−5^	0.036

Significant gene-sets (*p*_*FDR*_ < 0.05) are reported.

## Discussion

Our results demonstrate the existence of higher-order genetic effects on DNA CpG methylation. However, it is important to stress that the absolute number of CpGs that showed replicable higher-order genetic effects was low (*N* = 404 CpG in total, 0.1% of all CpGs). The major fraction of SNP-pairs showing significant interactions were located in *cis* of the CpG, with both SNPs being in LD, which might lead to biased estimates^27,28^. Interestingly, for CpGs exhibiting interaction effects we detected significant enrichment for DNase I hypersensitivity sites paralleled with strong enrichment of significant associations with gene expression. The underrepresentation of these CpG-sites in CpG islands further support their association with transcriptionally active regions. Finally, these CpGs were enriched in pathways related to HPV-infection and cancer.

We stress that the success of the analysis performed herein strongly relies on effect-sizes. With our data we were adequately powered to detect and replicate medium to strong effect sizes in the exhaustive search. However, especially for effects in *trans* we can expect a more complex picture with an accumulation of small effect sizes^31^. Under this scenario, the sample size needed to detect these effects is considerably larger.

Despite the fact that we focused an a complex trait that is biologically very close to the genetic variation^21–23^, we did not find strong evidence for the existence of epistatic effects. This result adds to the ongoing debate of the existence and relevance of epistasis^5,6^. We note that the computational effort to approach questions related to epistatic effects is considerably larger in comparison to investigating independent additive models, especially in the context of an exhaustive search. This speaks more in favor of refining the simple additive model before adding another level of complexity by including epistatic effects. Focusing on SNPs that show a significant main effect on the CpG resulted in a similar detection rate in comparison to performing an exhaustive search. This strategy optimizes the ratio between computational burden and the overall detection rate of interaction effects.

Taken together, our results demonstrate the existence of higher-order influences of structural genetic variation on the CpG signal. However, they also show that the impact of these higher-order dependencies is much weaker in comparison to main effects. Interestingly, filtering for CpGs that were under strong and more complex genetic control increased the power to detect CpGs that are associated with gene expression and biological pathways associated to HPV-infection and cancer.

## Methods

### Subjects and study design

The subjects included in this blood-DNA-methylation study (see Table 1) represent subsets of two ongoing studies, which were described previously^32,33^. The purpose of the ongoing studies is to identify biological correlates of cognitive performance by using genetics, electroencephalography and imaging techniques in healthy young adults from the general population. Saliva samples and phenotypic information were collected at the time-point of the main investigation. Subjects were later re-invited for additional saliva and blood sampling (see Table 1). Aim of this re-invitation was to additionally collect high-quality DNA from blood e.g. for the study of DNA-methylation and DNA-expression, without assessing further phenotypes. Blood and saliva samples were collected between midday and evening (mean time = 2:30 p.m., range 1:00 p.m.–8.00 p.m.). Hematological analysis, including blood cell counts, was performed with Sysmex pocH-100*i™* Automated Hematology Analyzer (Sysmex Co, Kobe, Japan).

Subjects were of good general health, did not self-report any neurological or psychiatric illness and did not take any medication (apart from oral contraception) at both time points. The phenotypic data reported here is based on the data freezes that have been used to select the subjects for the blood-DNA-methylation study (discovery sample data freeze 2013-08, *N* = 1,174 subjects; replication sample data freeze 2014-04, *N* = 1,935 subjects). Subjects were only included in the DNA-methylation study if they had been genotyped previously (Affymetrix 6.0, after QC, see below) and blood had been sampled. For the replication sample, additional requirements were a European genetic background (see below) and a time-distance of less than four years between the main investigation and the blood sampling. About 55% of the subjects from the discovery sample and 28% of the subjects from the replication sample fulfilled these requirements when planning the DNA-methylation study. Individuals were selected randomly from these pools of subjects.

The ethics committee of the Cantons of Basel-Stadt and Basel-Landschaft approved the studies. All participants received general information about the study and gave written informed consent. All methods were performed in accordance with the relevant guidelines and regulations of the participating institutions.

### Affymetrix SNP 6.0 based genotyping and imputation

Saliva samples were collected using the Oragene DNA Kit (DNA Genotek, Kanata, Canada). Saliva DNA was extracted from the Oragene DNA Kit using the standard precipitation protocol recommended by the producer. DNA isolated from saliva was investigated with Affymetrix SNP 6.0 array as described in the Genome-Wide Human SNP Nsp/Sty 6.0 User Guide (Affymetrix, Santa Clara, CA USA; see Supplementary text). The mean call-rate per subject was 98.5% (90.1–99.7%).

The genotypic data was projected on the two first PCA components inferred from HapMap reference populations (YRI, CEU and CHB-JPT populations). Outliers were identified using a Bayesian Clustering Algorithm^34^. *N* = 35 subjects out of the discovery sample for which DNA-methylation data was available were identified as outliers and excluded from the statistical analyses.

To reduce the computational burden for the interaction analyses, we used stringent QC-criterion for the exhaustive search for SNP-SNP-interactions: in both samples MAF > 2%, *p*_HWE_ > 0.001, missing rate per SNP < 1%, size of smallest SNP-group ≥ 15. To further eliminate highly redundant information, we additionally applied LD-based-pruning in the discovery sample with the following settings: window-size 50 KB, number of SNPs to shift 5, SNP-SNP *r* ^2^ = 0.95, resulting in *N* = 192,955 SNPs. For the in-depth modeling of additional main effects of SNPs we used a more-comprehensive SNP-set based on the following settings: in both samples MAF > 2%, *p*_HWE_ > 0.001, missing rate per SNP < 1% (*N* = 517,504 SNPs).

To determine a sample-specific threshold for Linkage Disequilibrium, we estimated for 10,000 randomly drawn SNPs from the larger SNP-set the association (*r* ^2^) with 10,000 random SNPs located on a different chromosome, based on the data from the discovery sample. We set the LD-threshold to *r* ^2^ > 0.021, which was very unlikely (*p* < 0.001) to happen by chance between independent SNPs in discovery sample (see Supplementary Fig. S5).

### HumanMethylation Infinium 450 K BeadChip based methylation analyses

DNA isolated from peripheral blood was investigated with the Illumina 450 K array (Illumina, Inc., San Diego, CA, U.S.A; see Supplementary text). Postprocessing was done for each sample separately, combining the β-values of the preprocessed data of all batches per sample (see Supplementary text). The β-values were processed step-by-step in order to correct for further influential and putative confounding factors: (1) using logit-transformation (M-value^35^, done with the R-package car^36^); (2) z-transformation per plate (correcting for plate and batch effects); (3) regressing out the first 8 (discovery sample) or 7 (replication sample) axes of a principal component analysis (PCA, done with the R-package pcaMethods^37^). The PCA was based on CpGs with no missing values ( >95% of the included CpGs). The PCA-based approach corrected for technical biases as well as for part of the variability induced by blood cell composition^25^ (4) regressing out the effects of sex and age; (5) regressing out the effects of variants in the 50mer probe sequence, if the total variance explained by these variants exceeded 0.1% (see below). The accepted missing rate per CpG was set to <1% in both samples. We further excluded cross-hybridizing probes and polymorphic CpGs sites^38,39^ (*N*_*max*_ = 63,974). Only CpGs surviving all filtering steps in both samples were used for the downstream analyses (*N* = 395,431).

### Correction for genetic variants in the 50mer probe sequence

We performed imputation of the genetic data: Prior to autosome-wide genotype imputation, haplotype estimation was performed using SHAPEITv2^40^, allowing a per individual and a per SNP missing rate for observed markers of max. 5%. After pre-phasing, genotype imputation was performed using IMPUTE v2.3.0, which imputes missing genotypes using a multi-population reference panel^41,42^. The integrated variant callset of 1,092 individuals from the 1000 Genomes Project (release v3 in NCBI build 37/hg19 coordinates, March 2012) served as panel data (http://mathgen.stats.ox.ac.uk/impute/ALL_1000G_phase1integrated_v3_impute_macGT1.tgz).

Based on the genomic location we filtered for all 50mer probe-sequences that comprises imputed variants. As a very basic QC, we excluded imputed variants with a minor allele frequency (MAF) <0.03% in our population (based on information of *N* = 3,166 subjects). For each 50mer probe sequence containing at least one variant, we build a linear model with the probability information of all imputed genotypes of this probe sequence as independent variables, and the CpG signal as dependent variable. If the overall explained variance of this model exceeded 0.1%, we used the residuals of this model as 50mer-corrected CpG signal. This procedure was done independently for the discovery sample (*N* = 533 subjects after outlier exclusion) and the replication sample (*N* = 319 subjects). Out of the 395,431 CpGs, *N* = 121,868 were corrected for 50mer variants in at least one of the two studies (discovery sample *N* = 98,532; replication sample *N* = 103,462; overlap *N* = 80,126).

### Exhaustive search for epistatic effects – Discovery phase

In the discovery sample we performed an exhaustive genome-wide search for two-locus (SNP-SNP) epistatic effects (*N* = 192,955 SNPs; *N* = 1.85 × 10^10^ SNP-pairs) on single CpG methylation levels (*N* = 395,431 CpGs), resulting in 7.36 × 10^15^ tests (*N*_*CpGs*_ * *N*_*snps*_ * (*N*_*snps*_ - 1)/2) to calculate. Accordingly, the Bonferroni-corrected threshold (alpha 5%) was set to *p* < 6.8 × 10^−18^. The statistical analysis in the discovery phase was based on a simplified interaction analysis strategy that corresponds to an analysis of variance based on 9 genotype groups as fixed effects^26^. The 8 degrees of freedom (df) test determines the joint genetic effects at two loci (combination of main and interaction effects). For the interaction analyses only, the additive and dominance effects at each locus were subtracted from the mean effects of each pairwise genotype. This 4 df parameterization is an approximation to a true interaction test^1,26^. Independence between the SNPs is a prerequisite for an accurate 4 df test, which is often not fulfilled, especially for SNPs in proximity to each other. Additionally, the accuracy of the interaction approximation decreases if there is a large (additive or dominant) main effect of a SNP. To minimize the bias of the simplified interaction analysis on the results, we recalculated all results with an F-value > 22 (4 df; *p*_*approx*_ < 8.0 × 10^−17^) of the simplified EpiGPU interaction analysis strategy with a linear regression in combination with an ANOVA-approach. Only analyses that remained significant after Bonferroni-correction entered the next analytical step. In most of these cases (99.89%) the simplified *F*-value was larger than the *F*-value based on recalculation, indicating an over-estimation of the true effects when using the simplified *F*-value only.

### Exhaustive search for epistatic effects – Replication phase

In the replication sample we applied a per-CpG Bonferroni-corrected *p-*value threshold. As baseline value for the multiple testing correction we used the number of *N* = 13′112 unique CpGs (*p* < 3.8 × 10^−6^). In case a CpG showed multiple interaction signals, we applied a more stringent correction by additionally accounting for the number of interaction hits per CpG (*p*_*int*_ < [0.05/(13′112 + number of interaction hits per CpG]). This procedure resulted in a per-CpG *p*-value threshold ranging from 3.8 × 10^−6^ (one SNP-pair only) up to 8.4 × 10^−7^ (46′314 SNP-pairs). To additionally derive an empirical *p*-value we performed on average 1.70 × 10^7^ permutations of the CpG-signal and recalculated the interaction analysis with the permuted signals to derive an empirical *F*-distribution, for each CpG separately. We performed a sign-test by comparing the average methylation level of the 9 SNP-groups between the two samples with Pearson correlations *r* (see Supplementary Fig. S4). Analyses had to pass all three filters (per-CpG Bonferroni correction based on *p*-value derived from *F*-distribution and from empirical *F*-distribution and sign-test *r* > 0.85).

We tested the stringency of the per-CpG empirical *p*-value (*p*_*emp*_ < 3.8 × 10^−6^) in comparison to a global Bonferroni-correction for all tests (alpha 5%, *p*_*bonf*_ < 5.8 × 10^−9^, correcting for 8′608′567 tests, with *p*-values derived from the F-distribution). The empirical *p*-value was as at least as stringent (49% of analysis results survived) as Bonferroni-correction for all tests (52% of all analysis results survived) and had a higher concordance rate with the sign-test: only 4% of analysis surviving the empirical *p*-value failed the sign-test, in comparison to 10% when applying Bonferroni-correction for all tests.

### Per CpG modeling

For this analysis we used the entire SNP-set (*N* = 517′504 SNPs instead of *N* = 192′955 SNPs), allowing for a better resolution. We applied a stepwise-forward regression approach, starting with main-effects. For each CpG, we first tested main effects of SNPs in a 500 KB window around the CpG and around the SNPs showing an interaction effect, as well as all SNPs that showed a significant main effect on a genome-wide scale (Bonferroni correction *p*_*main-effect*_ < 9.7 × 10^−8^). We then also included interaction effects by testing all already identified interacting SNP-pairs as well as all possible SNP-pairs for SNPs that were in LD (*r* ^2^ > 0.021 in the discovery sample) with at least one of the identified interacting SNPs. SNPs and SNP-pairs entering the forward regression model were sorted by their main effect *p*-value and interaction effect *p*-value of the discovery sample. We kept SNPs in the model based on their main effect and SNP-pairs based on their interaction effect, if their *p*-value in the forward-regression analysis was smaller than *p* = [0.05/(number of main-effects + number of interaction-effects tested per CpG)] in both the discovery and the replication sample.

### Search for epistatic effects based on SNPs exhibiting main effects

For each CpG we restricted the analysis to SNPs located within a ± 3.5 MB window around the CpG, using the larger SNP-set (*N* = 517,504 SNPs). For these SNPs we evaluated linear models with ANOVAs by using a 2-df parameterization of the SNP’s main effect on the CpG signal. We searched for main effects of SNPs that survived a per-CpG Bonferroni correction accepting an alpha error of 5% per CpG, by correcting for the number of main effects tested per CpG in the discovery sample. SNP-effects surviving this analysis were tested in the replication sample, again by using a per-CpG Bonferroni correction accepting an alpha error of 5%, correcting for the number of main effects tested in the replication sample. For CpGs showing at least two significant main effects, we tested for significant interaction effects in the discovery and replication sample, restricting the analyses to interaction effects in which the minimal group size was larger than 3 in both, the discovery and replication sample. We applied a per-CpG Bonferroni correction (alpha error 5%, correcting for the number of interaction effects tested) in both samples to identify significant interactions. Next, for each CpG we run a stepwise forward regression approach that included all significant main effects and interaction effects. We sorted all SNPs by their main effect and all SNP-pairs by their interaction effect *p*-value of the discovery sample. Main effects entered the forward regression analysis first. In the model we kept SNPs based on their main effect and SNP-pairs based on their interaction effect, if their *p*-value in the forward-regression analysis was smaller than *p* = [0.05/(number of main effects + number of interaction effects tested per CpG)] in both the discovery and the replication sample.

### Affymetrix HTA 2.0 array transcriptome analysis

Blood samples were collected using PAXgene Blood RNA Tubes (PreAnalytix Qiagen/BD, Switzerland). Expression profiles were measured with the Affymetrix GeneChip Human Transcriptome Array 2.0 (see Supplementary text). Individual expression values of each transcript were adjusted for age, sex and 23 components of a PCA by using linear regression models, based on data from *N* = 416 unrelated subjects. The PCA was derived from the expression data, 23 components of the PCA were chosen to optimize the signal-to-noise ratio for association analyses with genetic marker.

### Enrichment analyses

We used the genomic hg19 database (genome-mysql.cse.ucsc.edu; accessed 2016-08) to retrieve data about the location of CpG Islands (table cpgIslandExt), DNase I hypersensitivity sites (table wgEncodeR- egDnaseClusteredV3) and transcription factor binding sites (table wgEncodeRegTfbsClusteredV3). For *N* = 408 of our subjects, phenotypic data (data freeze 2015-09), transcriptomic data (*N* = 63,280 transcripts; see above) as well as DNA-methylation data (*N* = 395,431 CpGs) was available. For each CpG we calculated a genome-wide association analysis (Pearson’s correlation coefficient) with the transcriptomic data (*N* = 2.50 × 10^10^ analyses). We identified significant CpG-transcriptome associations on a genome-wide scale (alpha = 5%, Bonferroni correction, *p*_*bonf*_ < 2.0 × 10^−12^) and in *cis* (alpha = 5%, *p*_*cis*_ = 0.05/N_local_transcripts_; N_local_transcripts_ is the number of transcripts per CpG within a 500 KB-window, mean = 40, min = 0, max = 186).

For CpGs showing significant interactions, we estimated the similarity between CpGs with Pearson’s correlations. CpGs with an *r* > 0.8 were assigned to one cluster. For each cluster we randomly chose one CpG before performing the enrichment analyses. Each CpG was classified as being located within a CpG Island, a DNase I hypersensitivity site or a transcription factor binding site and whether it was significantly associated with a transcript. We compared the observed frequencies from CpGs that show an interaction effect against the expected frequency from all other CpGs by using *Chi* ^*2*^-tests.

We performed a gene ontology (GO) enrichment analysis using the ‘gometh’-function from the R-package missMethyl^43^. The algorithm corrects for the varying numbers of CpGs that can be mapped to single genes. We used both, the GO-database as well as the KEGG-database provided in the package missMethyl, restricting the analysis to pathways with at least 10 members (*N* = 8,596 in total, GO-database *N* = 8,288 out of 21,671 pathways; KEGG-database *N* = 308 out of 320 pathways). We applied FDR-correction based on the total number of *N* = 8,596 pathways included in the analysis.

### Software

If not mentioned differently, analyses were conducted in R (version: 2.15.1 and higher, R Development Core Team, 2012).

### Data availability

The data that support the findings of this study are available from the corresponding author upon request.

## Electronic supplementary material

Supplementary information

Supplementary Table 1

Supplementary Table 2

Supplementary Table 3
