# Supplementary material for: Exhaustive search for epistatic effects on the human methylome
Source: Sci Rep. 2017 Oct 20;7:13669. doi: 10.1038/s41598-017-13256-9 (PMC5651902; doi:10.1038/s41598-017-13256-9)
Supplement: Supplementary file 1 — Supplementary information [file 41598_2017_13256_MOESM1_ESM.pdf]

SUPPLEMENTARY INFORMATION FOR

Exhaustive search for epistatic effects on the human methylome

Authors:

Tobias Egli[1,2], Vanja Vukojevic[1,2,4], Thierry Sengstag[6,7], Martin Jacquot[6], Rubén Cabezón[6], David Coynel[2,5], Virginie Freytag[1,2], Angela Heck[1,2,3], Christian Vogler[1,2,3], Dominique J.-F. de Quervain[2,3,5], Andreas Papassotiropoulos[1,2,3,4], Annette Milnik[1,2,3,*]

Affiliations:

1. Division of Molecular Neuroscience, Department of Psychology, University of Basel, CH-4055 Basel, Switzerland
2. Transfaculty Research Platform Molecular and Cognitive Neurosciences, University of Basel, CH-4055 Basel, Switzerland
3. Psychiatric University Clinics, University of Basel, CH-4055 Basel, Switzerland
4. Department Biozentrum, Life Sciences Training Facility, University of Basel, CH-4056 Basel, Switzerland
5. Division of Cognitive Neuroscience, Department of Psychology, University of Basel, CH-4055 Basel, Switzerland
6. sciCORE, Scientific Computing Center, University of Basel, CH-4056
7. SIB - Swiss Institute of Bioinformatics

*Corresponding author

Annette Milnik

University of Basel, Division of Molecular Neuroscience

Birmansgasse 8, CH-4009 Basel

Phone: + 41 61 267 0267

annette.milnik@unibas.ch

Supplementary Text

Affymetrix SNP 6.0 based genotyping. Genomic DNA concentration was adjusted to 50 ng/µl in water. Digestion was done with 250 ng of DNA, in parallel with 10 units of Sty I and Nsp I restriction enzymes (New England Biolabs, Knowl Piece, UK) for 2 hours at 37 °C. Following, enzyme specific adaptor oligonucleotides were ligated onto the digested ends with T4 DNA Ligase for 3 hours at 16°C. After adjusting volume to 100 µl with water, 10 µl of the diluted ligation reactions were subjected to PCR. Three PCR reactions of 100 µl were performed for Sty digested products and four PCR reactions for Nsp. PCR was performed with Titanium Taq DNA Polymerase (Clontech, Mountain View, CA) in the presence of 4.5 µM PCR primer 002 (Affymetrix, Santa Clara, CA), 350 µM each dNTP (Clontech), 1M G-C Melt (Clontech), and 1 X Titanium Taq PCR Buffer (Clontech). Following cycling parameters were used: 94 °C, 3 min; 30 x (94 °C, 30 s; 60 °C, 45 s; 68 °C, 15 s); 68 °C, 7 min. PCR products expected in the range size between 200-1100 bp were verified using 2 % (weight/volume) TBE gel electrophoresis. PCR products were pooled and purified with the Filter Bottom Plate (Millipore, Billerica, MA; P/N MDRLN0410) using Agencourt AMPure XP Beads (Beckman Coulter, Fuillerton, CA). Quantification of purified PCR products was done on a Zenith 200rt microplate reader (Anthos-Labtec, Cambridge, UK), with average yield of 4 to 5 µg/µl per sample. Subsequently, the SNP Nsp/Sty 5.0/6.0 Assay Kit (Affymetrix) was used. For the fragmentation around 250 µg of purified PCR products was digested using 0.5 units of DNAse I at 37°C for 35 minutes. Average size of fragmentation products less than 180 bps was verified using 4 % (weight/volume) TBE gel electrophoresis. Following, the DNA was end labeled with 105 units of terminal deoxynucleotidyl transferase at 37 °C for 4 hours. The labeled DNA was then hybridized onto Genome-Wide Human SNP 6.0 Array at 50 °C for 18 hours at 60 rpm. The hybridized array was washed, stained, and scanned according to the manufacturer's (Affymetrix) instructions using Affymetrix GeneChip Command Console (AGCC, version 3.2.0.1515). Generation of SNP calls and array quality control were performed using the command line programs of the Affymetrix Power Tools package (version: apt-1-14.4.1). According to the manufacturer's recommendation,

Contrast QC was chosen as QC metric, using the default value of greater or equal than 0.4. All samples passing QC criteria were subsequently genotyped using the Birdseed (v2) algorithm.

HumanMethylation Infinium 450K BeadChip based methylation analyses. Samples were collected using BD Vaccutainer Push Button blood collection set and 10.0 mL BD Vacutainer® Plus plastic whole blood tube, BD Hemogard™ closure with spray-coated K_2EDTA (Becton, Dickinson and Company , New Jersey, NJ). Hematological analysis, including blood cell counts, was performed at the collection time point with Sysmex pocH-100*i*™ Automated Hematology Analyzer (Sysmex Co, Kobe, JP.) DNA was isolated from the remaining fraction, upon plasma removal. The isolation was performed with QIAmp Blood Maxi Kit (Qiagen AG, Hilden, DE), using the recommended spin protocol.

In order to obtain high purity DNA prior to bisulfite conversion, isolated DNA samples were additionally re-purified. For this purpose, 2 µg of DNA isolated with QIAmp/Oragene procedure, was incubated overnight at 50 °C with proteinase K (Lysis buffer: 30 mM Tris·Cl; 10 mM EDTA; 1 % SDS, pH 8.0; 150ng/µl Proteinase K), agitated by gentle orbital shaking. Next, the DNA was purified using Genomic DNA Clean & Concentrate Kit (Zymo Research, Irvine, CA). The quality and concentration of DNA were assessed using gel electrophoresis, NanoDrop ND- 1000 (Thermo Scientific, Waltham, MA) and fluorometry measurements (Qubit dsDNA BR Assay Kit; Invitrogen, Carlsbad, CA), respectively.

The bisulfite conversion was performed with 500 ng genomic DNA input using the EZ DNA Methylation Gold Kit (Zymo Research). A bisulfite conversion quality control on the probes was performed with DNA qPCR reaction and subsequent melting curve analysis [1]. The bisulfite-converted DNA was processed and hybridized to the HumanMethylation450 BeadChip (Illumina, Inc, San Diego, CA), according to the manufacturer's instructions. The BeadChip images were scanned on the iScan system (performed at ServiceXS B.V., Leiden, Netherlands).

N = 568 subjects of the discovery sample were processed in two batches (2 plates and 4 plates, respectively). For the replication sample, N = 319 subjects were processed within a single

batch (4 plates). Within a batch samples were processed with a randomized plate assignment and with a single bisulfite conversion. Preprocessing was done separately for each batch. Data were extracted and analyzed from the generated idat files using the R package RnBeads version 0.99.9 [2]. CpG annotation was based on the manufactures annotation file (HumanMethylation450_15017482_v.1.2). During preprocessing, the background was subtracted using the "noob" method in the methylumi package [3], and the signal was further normalized using the SWAN algorithm [4]. The following probe categories were excluded from the final data sets, based on the annotation provided within the RnBeads package: non-CpG context probes (due to underrepresentation on the 450K array, 0.6 %, [5], functional differences when compared to CpG context as well as very low abundance of non-CpG methylation in somatic tissues [6]; $N = 3{,}091$), probes with a SNP mapping directly to the target CpG site, as well as probes with three and more SNPs mapping within the 50mer probe (MAF threshold was set to 0.01; $N = 18{,}998$ CpGs), gonosomal probes ($N = 11{,}473$ CpGs), non-specific probes. Using the Greedycut algorithm, we iteratively removed the probes and data sets of the highest impurity (rows and columns in the detection p-value table that contain the largest fraction of unreliable measurements; $p < 0.05$ [2]). We performed a cross-platform validation of genotyping and methylation data using the reported sex and the sex-predictions based on the arrays data, as well as matching of all SNPs represented on the Illumina 450K array to the corresponding Affymetrix SNP 6.0 or imputation derived genotype calls. This crosscheck allowed an unambiguous assignment of each methylation dataset to the corresponding genetic and phenotypic dataset.

Affymetrix HTA 2.0 array transcriptome analysis. Total RNA was further isolated with the PAXgene Blood miRNA Kit (PreAnalytix, Switzerland). Following, a second, additional purification was performed with the miRNeasy Micro Kit (Qiagen, Germany). The concentration and quality of the RNA was determined using Nanodrop 2000 (ThermoScientific, USA) and RNA Nano 6000 Kit on Bioanalyzer 2100 instrument (Agilent, USA). Next, GLOBINclear™-Human Kit (Ambion, USA) was used for a non-enzymatic depletion of the alpha and beta globin mRNA

starting from 1 μg of total RNA preparations derived from whole blood, following a standard procedure. The concentration and quality of the "globin-free" RNA was assessed as described above. Following, the alpha and beta globin mRNA depletion was measured by qPCR. In brief: for reverse transcription, 350 ng of total RNA was denaturized for 8 min at 70 °C followed by ice incubation in the presence of 25 ng Anchored Oligo(dT)20 Primer (Invitrogen, USA) and 75 ng Random Decamers Primers (Ambion, USA). In the RT reaction, cDNA was generated in 25 μl reaction using Super RT kit (HT Biotechnology, Santa Cruz, CA USA). Upon completion of the reaction, the volume was adjusted to 200 μl in Lambda DNA solution (5 ng/μl final concentration; Promega, Fitchburg, WI USA). The primers were designed against splice variants that contain alpha-Globin gene: alpha-Globin Forward: 5'- GCACGCGCACAAGCT-3', and alpha-Globin Reverse: 5'- GGGTCACCAGCAGGCA-3' (Microsynth, Switzerland). The expression levels were normalized to RPLPO gene (human large ribosomal protein) using the following primers: RPLP0-Ex3-4_FW, 5'-CTCTGGAGAAACTGCTGC-3' and RPLP0-Ex3-4_RV, 5'-CTGATCTCAGTGAGGTCC-3' (Sigma Aldrich, USA). qPCR was performed using the Power SYBR Green PCR Master Mix (Life Technologies, USA) according to standard recommendations, in 12 μl final volume of reaction, using 2 μl of cDNA template, on RotorGene 6000A instrument (Corbett Research Pty Ltd, Sydney Australia). Cycling conditions were as follows: 95 °C, 60 s – 40x (95 °C, 3 s – 56 °C, 10s – 72°C, 4 s) followed by a melting curve analysis (61 °C to 95 °C, rising by 0.7 °C / 3s) to attest amplification specificity. Threshold cycles (crossing point) were determined using Rotor-Gene software version 6.1 (Corbett Research, Australia). RPLPO was selected as reference gene for normalization after we tested several candidate-reference genes, as had been previously described [7]. Expression levels were normalized using a geometric mean level of expression [7]. Fold differences were calculated using the delta-delta Ct method [8] with the help of qBasePlus software (Biogazelle, Ghent, Belgium).

Target synthesis was performed using Ambion® WT Expression Kit (Ambion, Life Technologies, USA) starting from 250ng of high-quality "globin-free" RNA, following the standard procedure. Next, 5.16μg of target cDNA was further labeled and prepared for hybridization with the GeneChip® WT Terminal Labeling and Hybridization Kit (Affymetrix,

USA). The prepared samples were loaded on Affymetrix GeneChip Human Transcriptome Array 2.0 (Cat# 902162) and hybridized for 16 hours (45 °C, 60 rpm) in Hybridization oven 640 (Affymetrix, USA). The arrays were washed and stained on Fluidics Stations 450 (Affymetrix) by using the Hybridization Wash and Stain Kit (Affymetrix, USA) under FS450_0001 protocol. The GeneChips were processed with an Affymetrix GeneChip Scanner 3000 7G (Affymetrix, USA). DAT images and CEL files of the microarrays were generated using Affymetrix GeneChip Command Control software (Affymetrix, USA). In order to account for technical inter-array variation we performed a full quantile-normalization; feature quantification was conducted using a median-polish on transcript-level according to the HTA 2.0. lib-set-version 0.3. (Affymetrix Power Tools version: 1.16.0). Cross-platform validation of genotyping and expression data was assessed using the MixUpMaper algorithm [9].

References

1. Kristensen, L. S., Mikeska, T., Krypuy, M. & Dobrovic, A. Sensitive Melting Analysis after Real Time- Methylation Specific PCR (SMART-MSP): high-throughput and probe-free quantitative DNA methylation detection. *Nucleic Acids Res.* **36,** e42 (2008).
2. Assenov, Y. *et al.* Comprehensive analysis of DNA methylation data with RnBeads. *Nat. Methods* **11,** 1138–1140 (2014).
3. Davis S, Du P, Bilke S, Triche T Jr & Bootwalla M. methylumi: Handle Illumina methylation data. R package version 2.12.0. (2014).
4. Maksimovic, J., Gordon, L. & Oshlack, A. SWAN: Subset-quantile within array normalization for illumina infinium HumanMethylation450 BeadChips. *Genome Biol.* **13,** R44 (2012).
5. Bibikova, M. *et al.* High density DNA methylation array with single CpG site resolution. *Genomics* **98,** 288–295 (2011).
6. Ziller, M. J. *et al.* Genomic distribution and inter-sample variation of non-CpG methylation across human cell types. *PLoS Genet.* **7,** e1002389 (2011).
7. Vandesompele, J. *et al.* Accurate normalization of real-time quantitative RT-PCR data by geometric averaging of multiple internal control genes. *Genome Biol.* **3,** RESEARCH0034 (2002).
8. Pfaffl, M. W. A new mathematical model for relative quantification in real-time RT-PCR. *Nucleic Acids Res.* **29,** e45 (2001).
9. Westra, H.-J. *et al.* MixupMapper: correcting sample mix-ups in genome-wide datasets increases power to detect small genetic effects. *Bioinformatics* **27,** 2104–2111 (2011).

Supplementary Figures

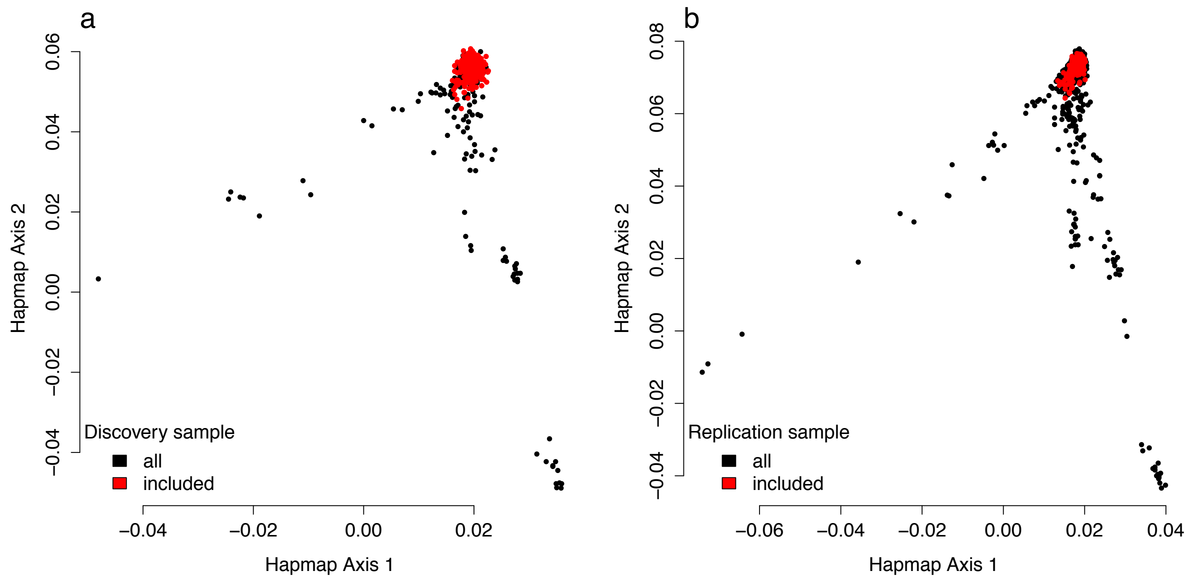

Supplementary Figure S1. Genetic background. Genetic data from both samples (**a**, discovery sample; **b**, replication sample) is projected on the two first PCA components inferred from HapMap reference populations (YRI, CEU and CHB-JPT populations). Only subjects with European ancestry (red dots) were included in the analyses.

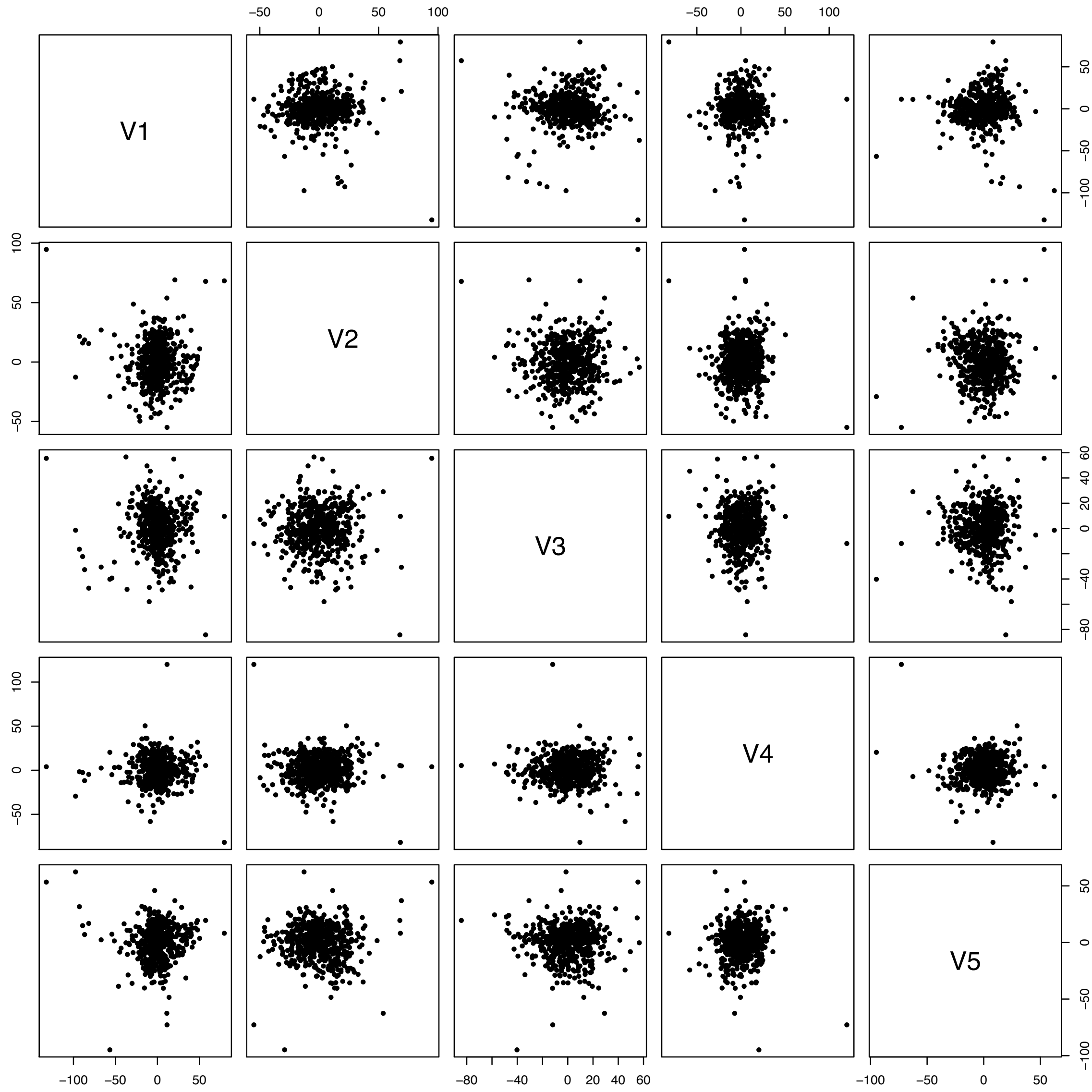

Supplementary Figure S2. CpG-data discovery sample, multidimensional scaling over samples. Depicted are scatterplots between the first five dimensions. The analysis is based on 40,000 randomly chosen CpGs without missing values.

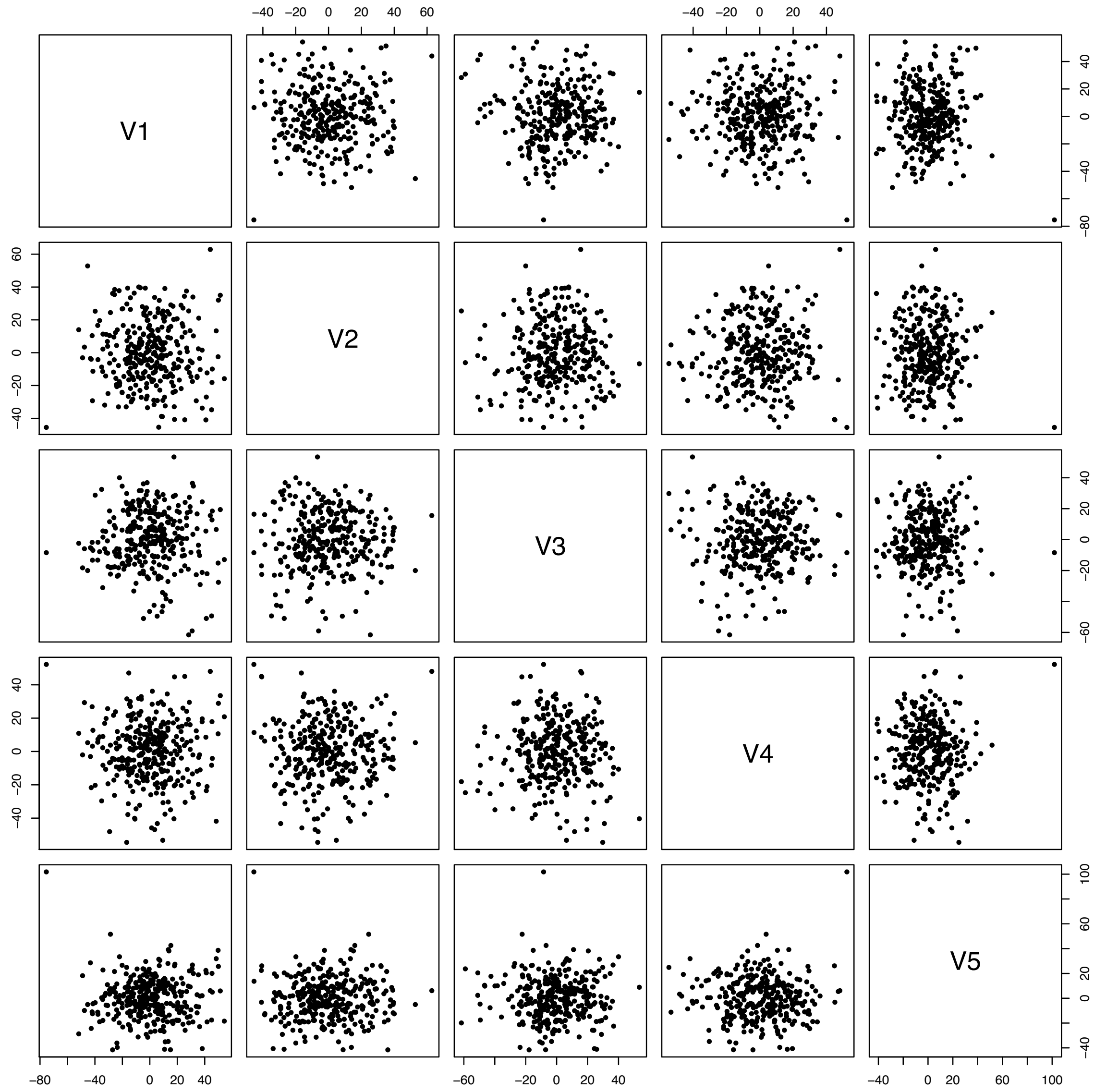

Supplementary Figure S3. CpG-data replication sample, multidimensional scaling over samples. Depicted are scatterplots between the first five dimensions. The analysis is based on 40,000 randomly chosen CpGs without missing values.

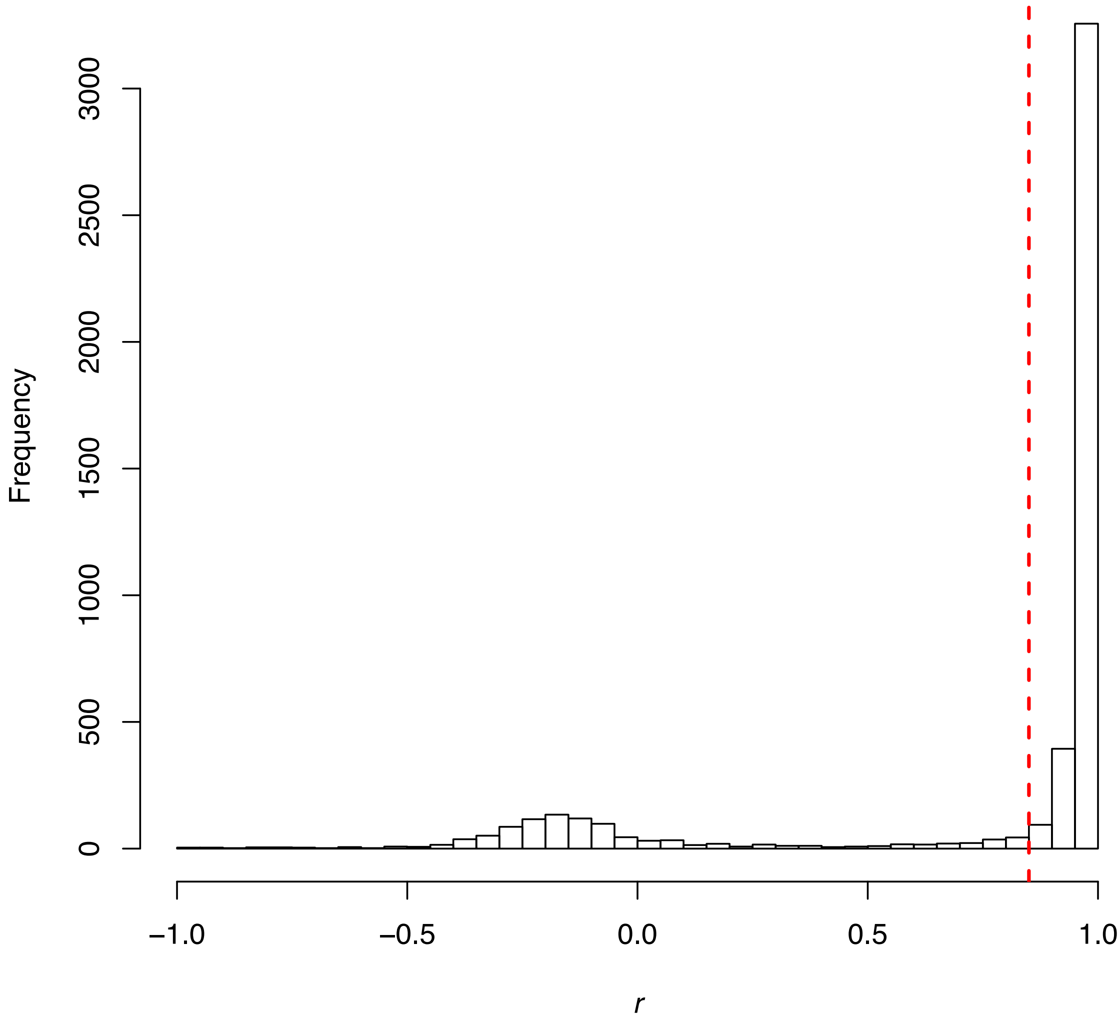

Supplementary Figure S4. Exhaustive search for epistatic effects, sign-test for the interaction term. Results are shown for the N = 4,816 interaction analyses that survived the initial replication based on a per-CpG Bonferroni-correction (before sign-test and permutation). We compared the average CpG-values of the 9 combined genotype-groups between the screening and replication sample with Pearson's correlations. The red dashed line corresponds to r = 0.85, which we used as threshold to determine interaction effects that show the same direction of effect in both samples.

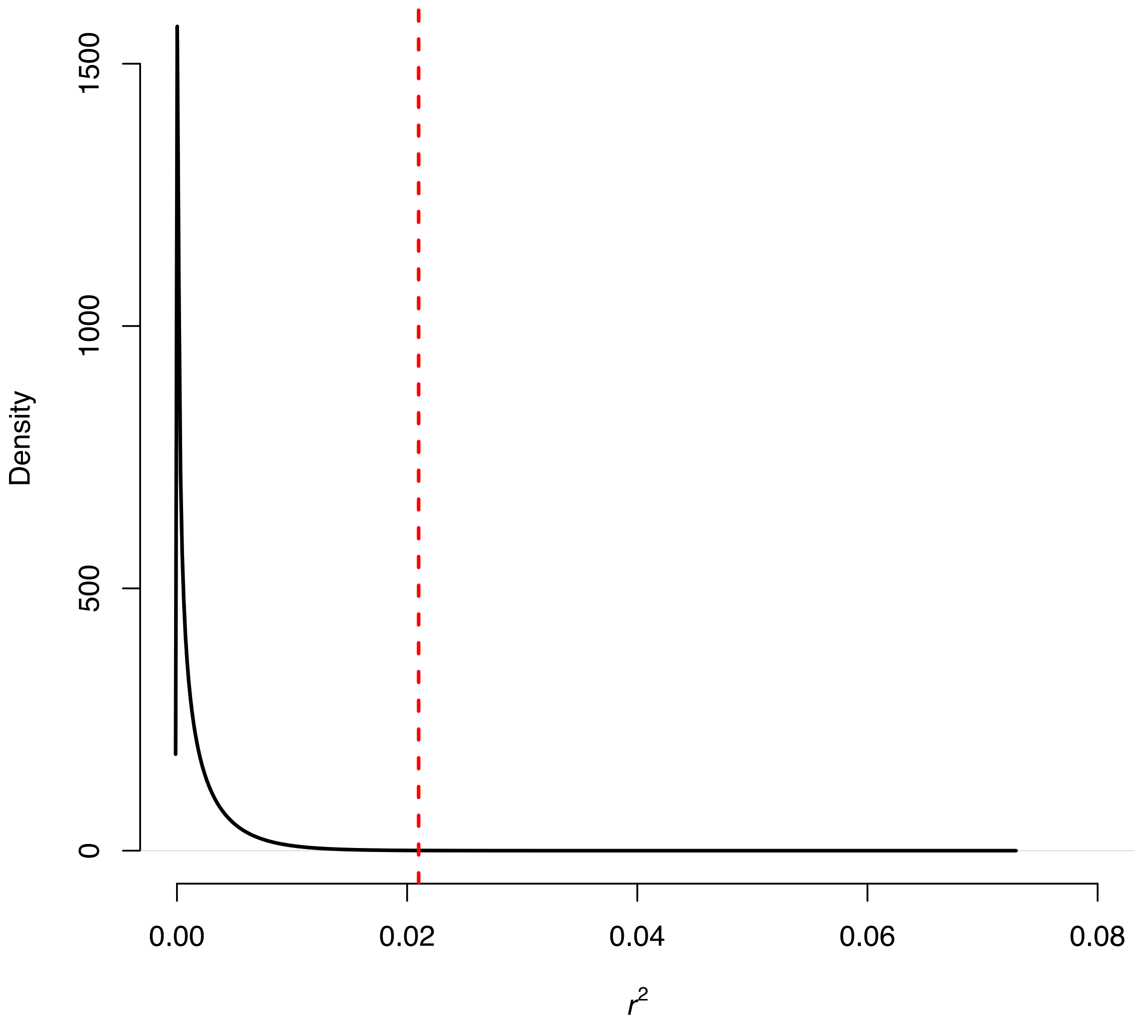

Supplementary Figure S5. Distribution of r^2-values between independent SNPs. In the discovery sample we determined a sample-specific threshold for Linkage Disequilibrium (LD) by estimating for 10,000 randomly drawn SNPs the association (r^2) with 10,000 random SNPs located on a different chromosome. Based on this result SNP-pairs showing values large than $r^2 = 0.021$ ($p < 0.001$) were considered as showing LD.

Supplementary Figures S6. Exhaustive search per-CpG models. Shown are the genomic locations for each of the 174 CpGs and all SNPs that were kept in the final per-CpG model. The top panel depicts information from the knownGene table (hg19) derived from UCSC genome browser (UCSC-GB). The second to panel (grey horizontal bar) depicts transcripts from the Affymetrix HTA 2.0 array that were significantly associated with the CpG signal in the discover sample. The next three panels depict information for CpG Islands, transcription factor binding sites (TFBS) and DNase I hypersensitivity sites (DNase I) derived from UCSC-GB. The next panel (CpG beta discovery sample) shows the average beta-values of CpGs and the 5 % - 95 % percentile in orange. The genomic location of each CpG is depicted with vertical grey lines. Data is shown for the discovery sample. The next panel shows the CpG (brown dot) and all SNPs (triangles and rhombi) that were kept in the final per-CpG-model. Interacting SNPs are shown as triangles in the same color. Black rhombi describe SNPs with a significant main effect in the full model. The next two panels (R^2 discovery and R^2 replication sample) depict the main effects of each SNP on the CpG signal, independently estimated. The red dashed line shows the variance explained by the per-CpG-model including main effects only, whereas the blue dashed line depicts the variance explained by the full model (including interaction effects). The bottom panel shows the LD between SNPs, an $r^2 < 0.021$ is shown in white.

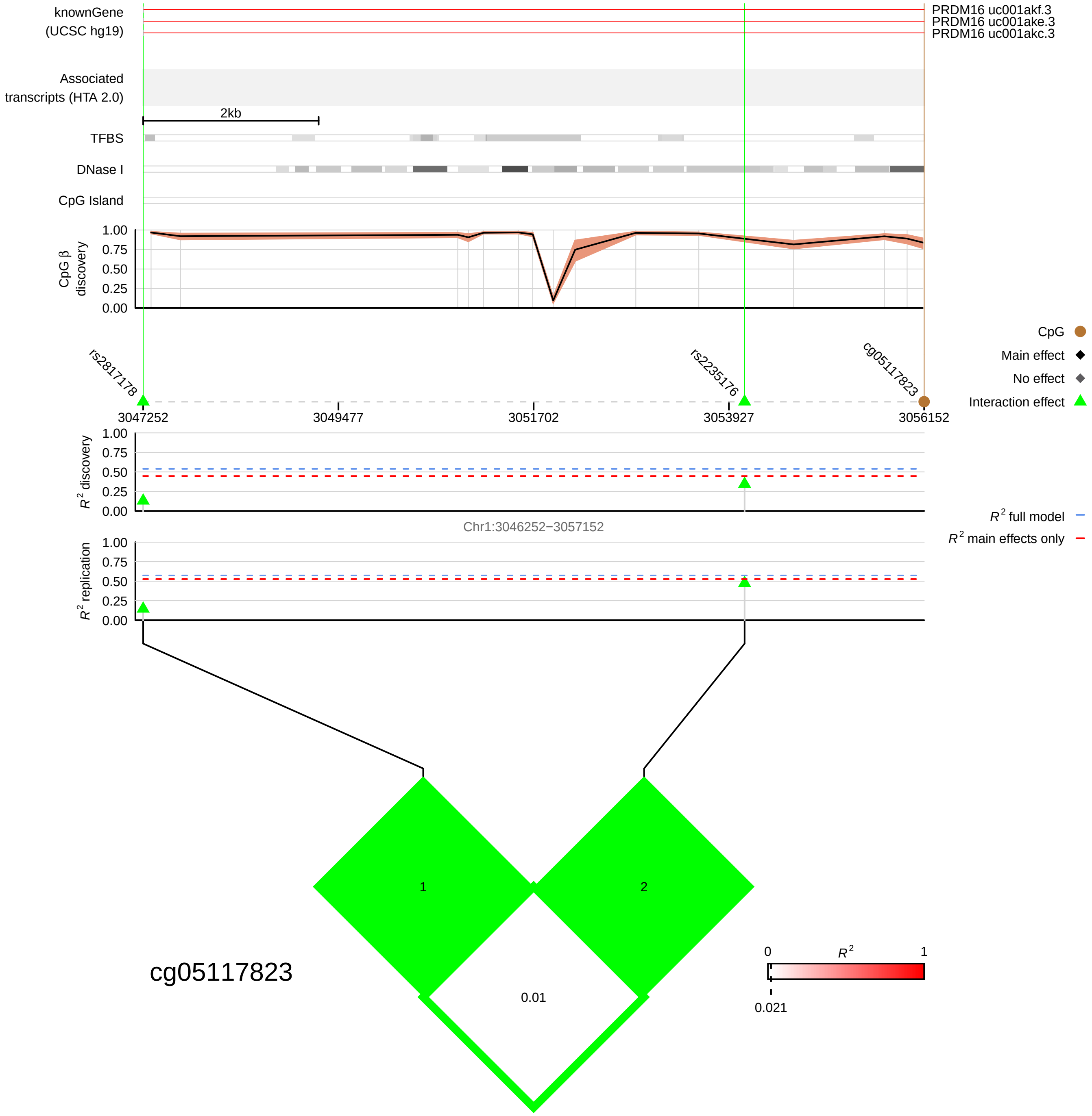

knownGene
(UCSC hg19)
PRDM16 uc001akf.3
PRDM16 uc001ake.3
PRDM16 uc001akc.3
Associated
transcripts (HTA 2.0)
2kb
TFBS
DNase I
CpG Island
CpG β
discovery
1.00
0.75
0.50
0.25
0.00
CpG
Main effect
No effect
Interaction effect
rs2817178
rs2235176
cg05117823
3047252
3049477
3051702
3053927
3056152
R2 discovery
Chr1:3046252−3057152
R2 full model
R2 main effects only
R2 replication
1
2
0.01
cg05117823
0
R2
1
0.021

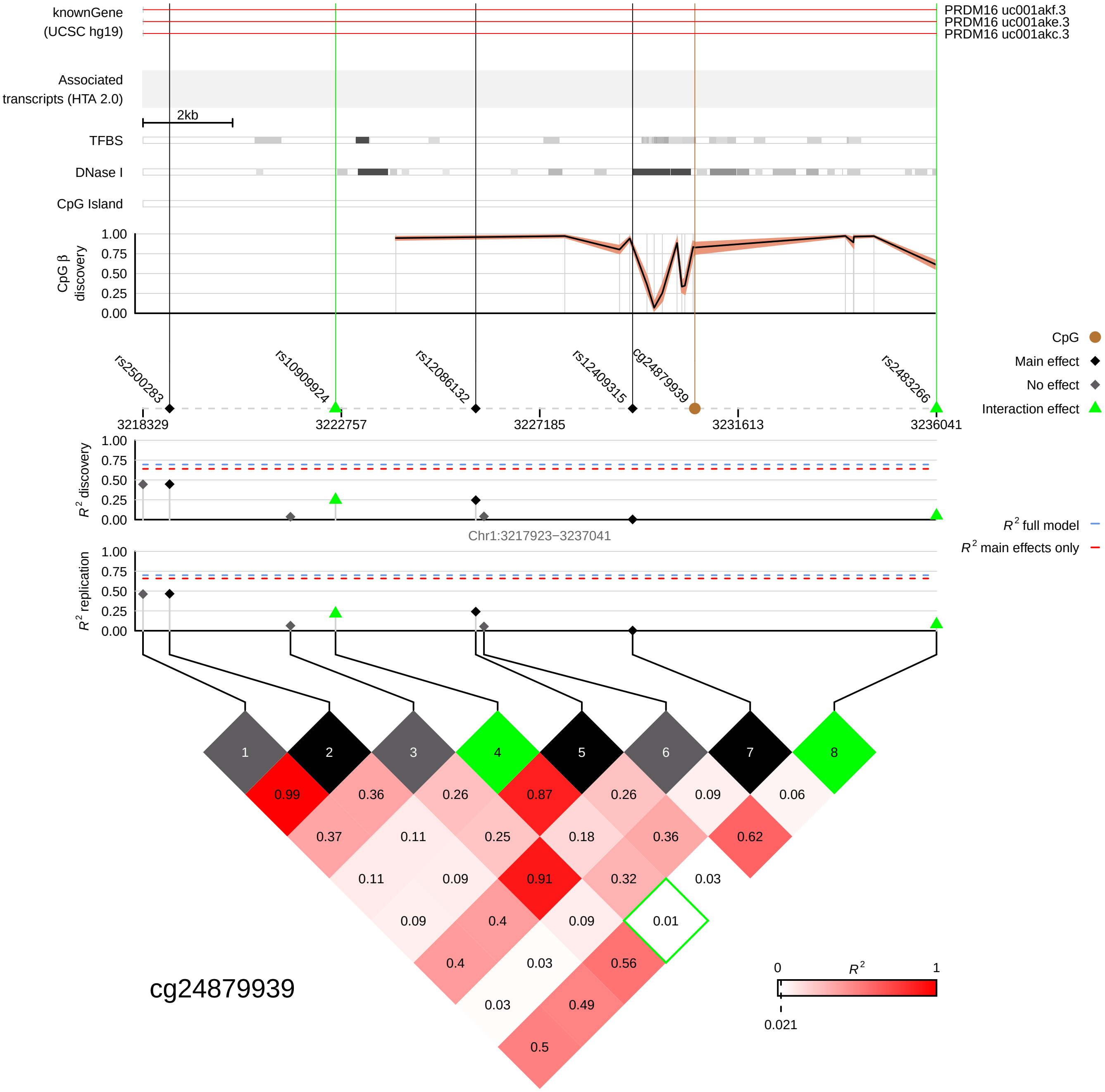
knownGene
(UCSC hg19)
PRDM16 uc001akf.3
PRDM16 uc001ake.3
PRDM16 uc001akc.3
Associated
transcripts (HTA 2.0)
2kb
TFBS
DNase I
CpG Island
CpG β
discovery
rs2500283
rs10909924
rs12086132
rs12409315
cg24879939
rs2483266
3218329
3222757
3227185
3231613
3236041
CpG
Main effect
No effect
Interaction effect
R^2 discovery
Chr1:3217923−3237041
R^2 replication
R^2 full model
R^2 main effects only
R^2
0.021
cg24879939

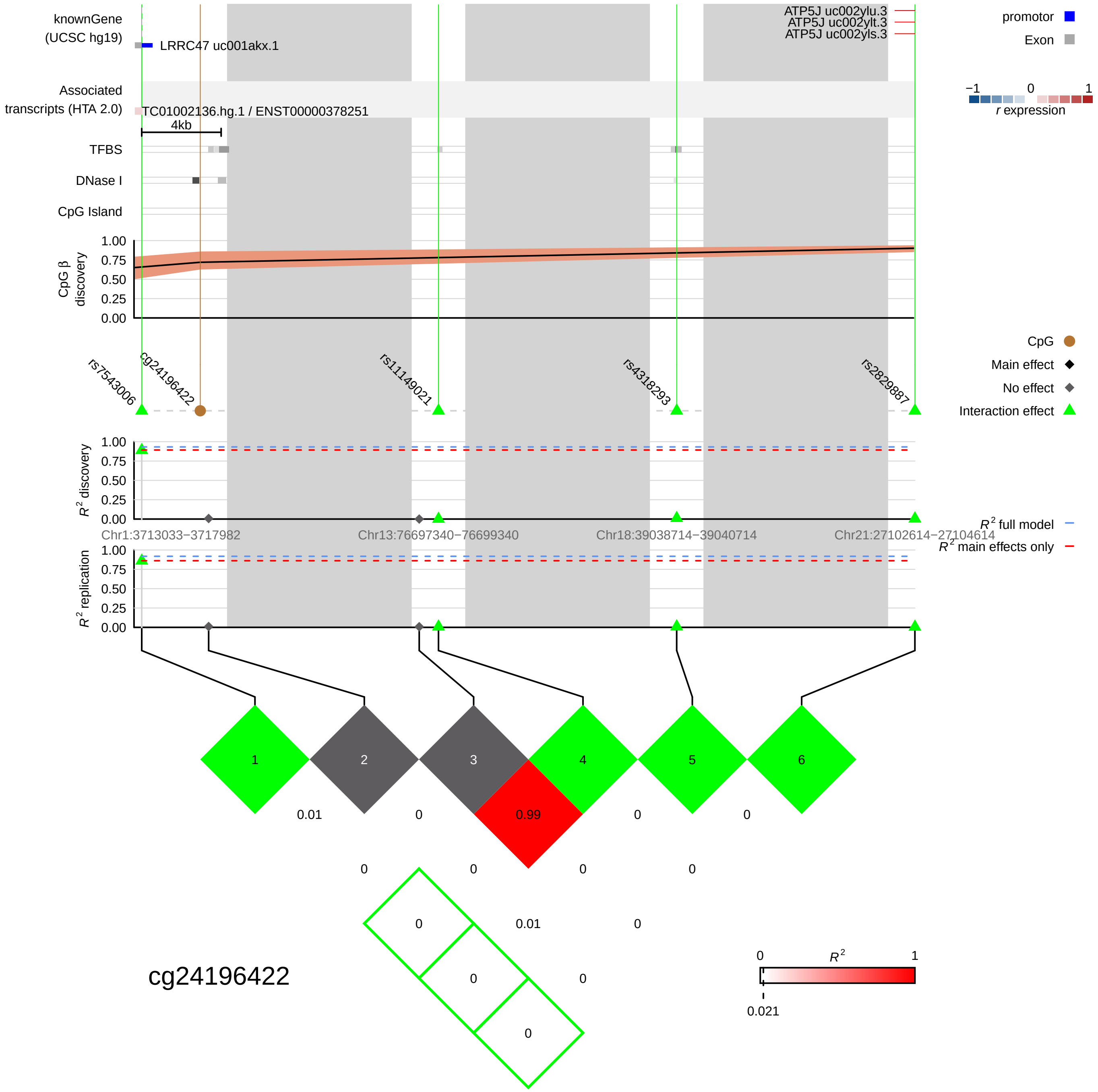
knownGene (UCSC hg19)
LRRC47 uc001akx.1
ATP5J uc002ylu.3
ATP5J uc002ylt.3
ATP5J uc002yls.3
promotor
Exon
Associated transcripts (HTA 2.0)
TC01002136.hg.1 / ENST00000378251
4kb
−1
0
1
r expression
TFBS
DNase I
CpG Island
CpG β discovery
rs7543006
cg24196422
rs11149021
rs4318293
rs2829887
CpG
Main effect
No effect
Interaction effect
R2 discovery
Chr1:3713033−3717982
Chr13:76697340−76699340
Chr18:39038714−39040714
Chr21:27102614−27104614
R2 full model
R2 main effects only
R2 replication
0.01
0.99
0.021
R2
cg24196422

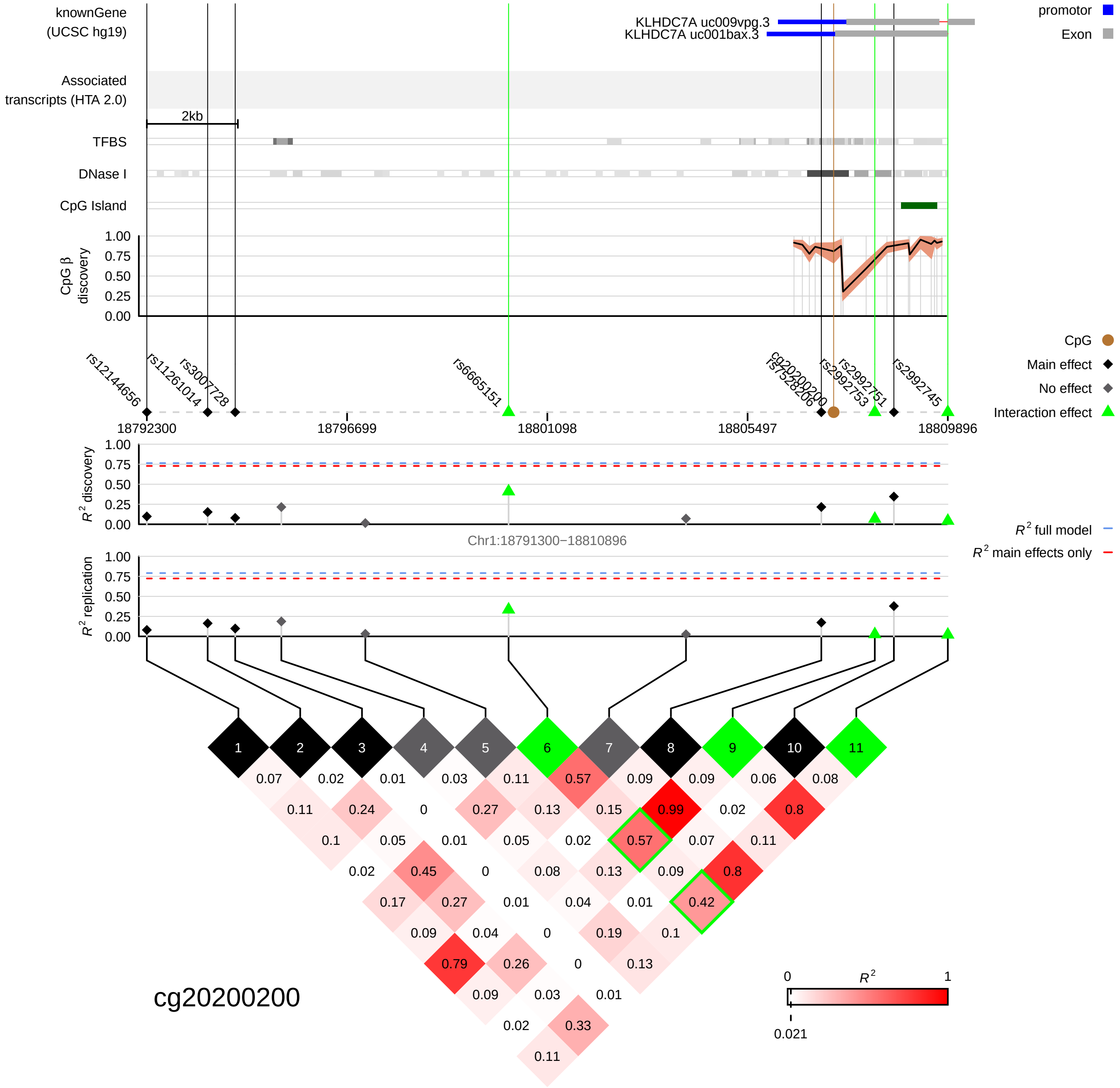
knownGene (UCSC hg19)
KLHDC7A uc009vpg.3
KLHDC7A uc001bax.3
promotor
Exon
Associated transcripts (HTA 2.0)
2kb
TFBS
DNase I
CpG Island
CpG β discovery
rs12144656
rs11261014
rs3007728
rs6665151
cg20200200
rs7528206
rs2992753
rs2992751
rs2992745
18792300
18796699
18801098
18805497
18809896
CpG
Main effect
No effect
Interaction effect
R² discovery
Chr1:18791300−18810896
R² full model
R² main effects only
R² replication
cg20200200
R²
0.021

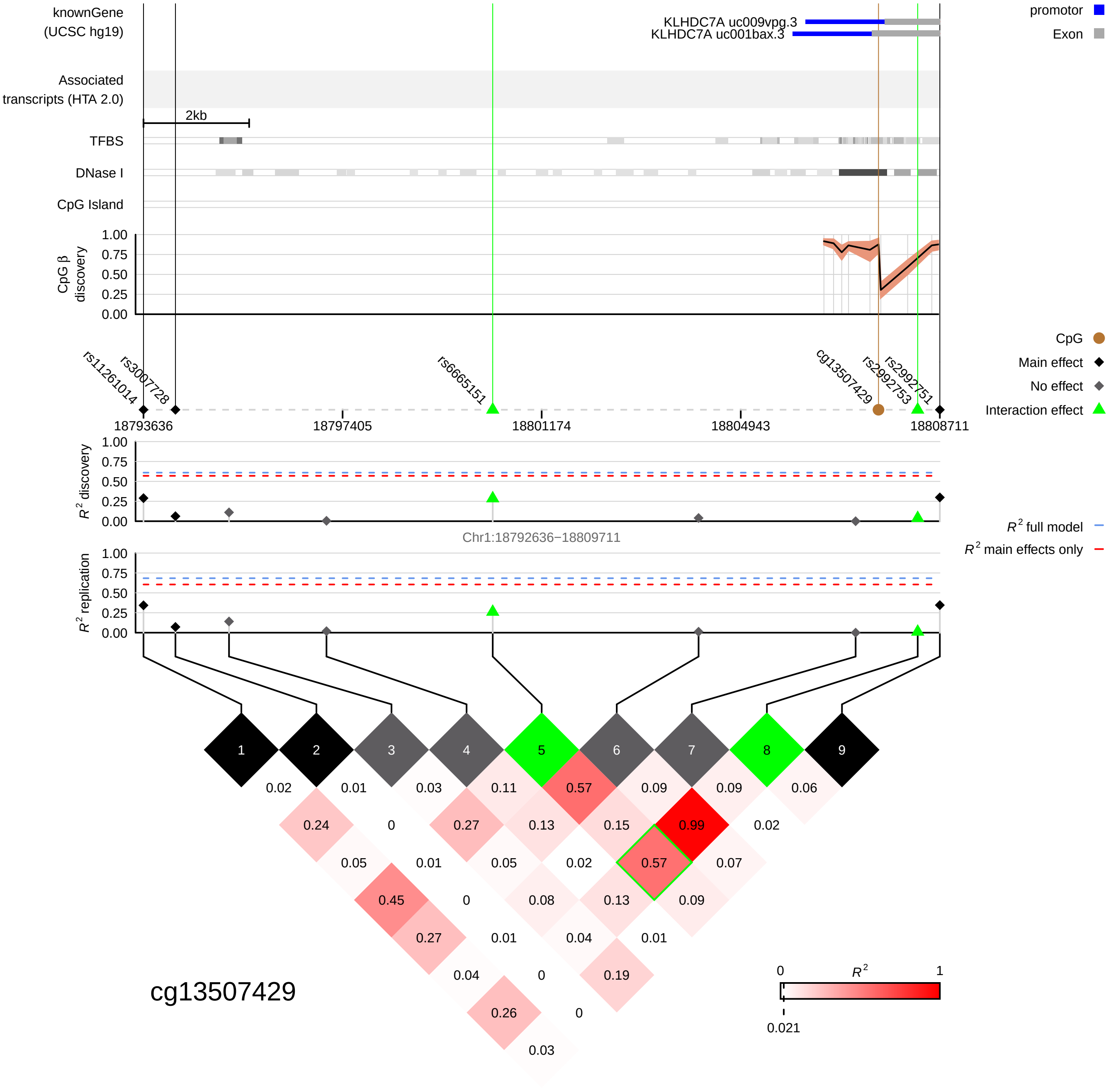
knownGene (UCSC hg19)
KLHDC7A uc009vpg.3
KLHDC7A uc001bax.3
promotor
Exon
Associated transcripts (HTA 2.0)
2kb
TFBS
DNase I
CpG Island
CpG β discovery
rs11261014
rs3007728
rs6665151
cg13507429
rs2992753
rs2992751
CpG
Main effect
No effect
Interaction effect
18793636
18797405
18801174
18804943
18808711
R² discovery
Chr1:18792636−18809711
R² full model
R² main effects only
R² replication
cg13507429
R²
0.021

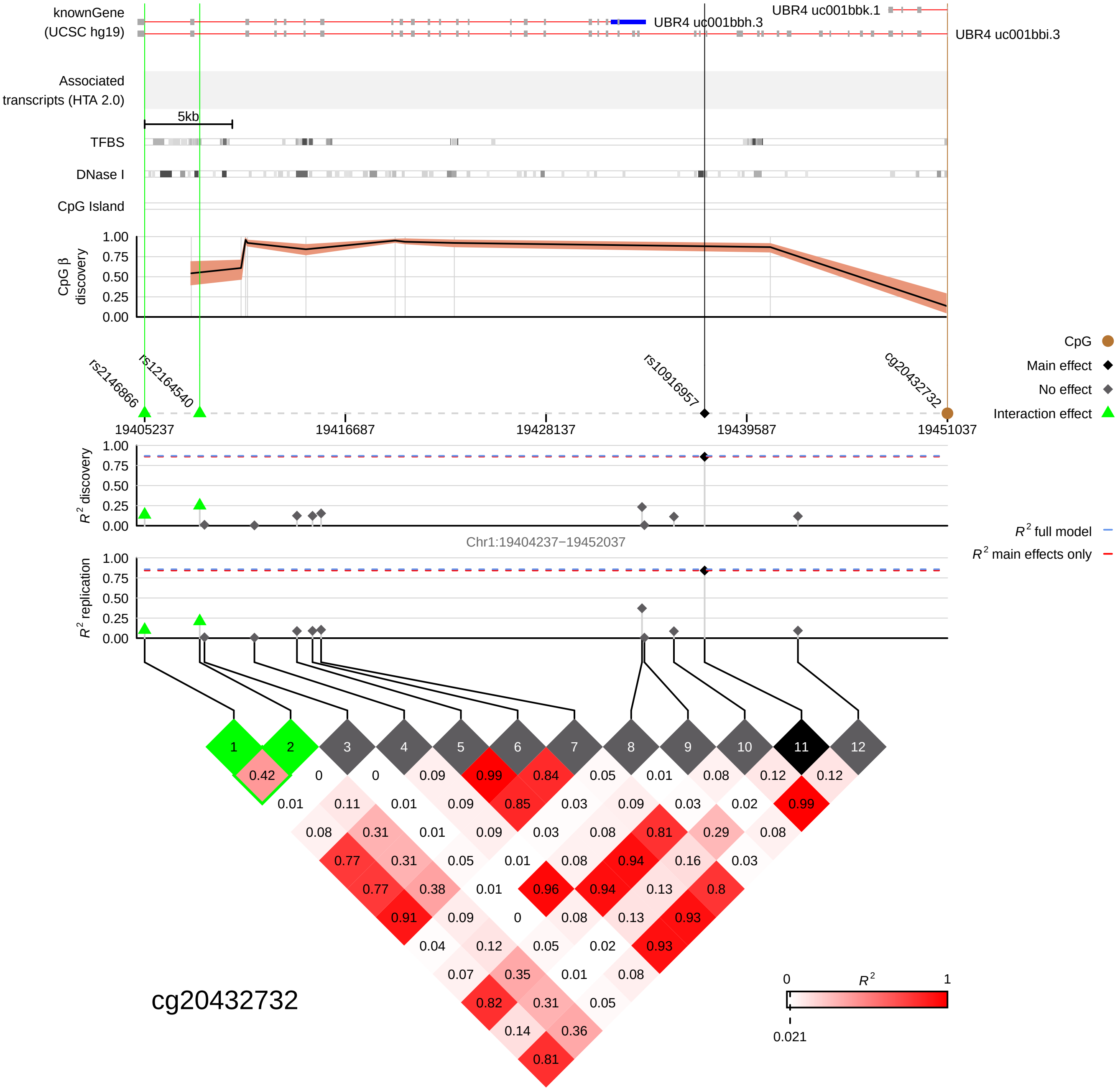
knownGene
(UCSC hg19)
UBR4 uc001bbk.1
UBR4 uc001bbh.3
UBR4 uc001bbi.3
Associated
transcripts (HTA 2.0)
5kb
TFBS
DNase I
CpG Island
CpG β
discovery
1.00
0.75
0.50
0.25
0.00
rs2146866
rs12164540
rs10916957
cg20432732
19405237
19416687
19428137
19439587
19451037
CpG
Main effect
No effect
Interaction effect
R^2 discovery
Chr1:19404237−19452037
R^2 full model
R^2 main effects only
R^2 replication
1
2
3
4
5
6
7
8
9
10
11
12
0
R^2
1
0.021
cg20432732

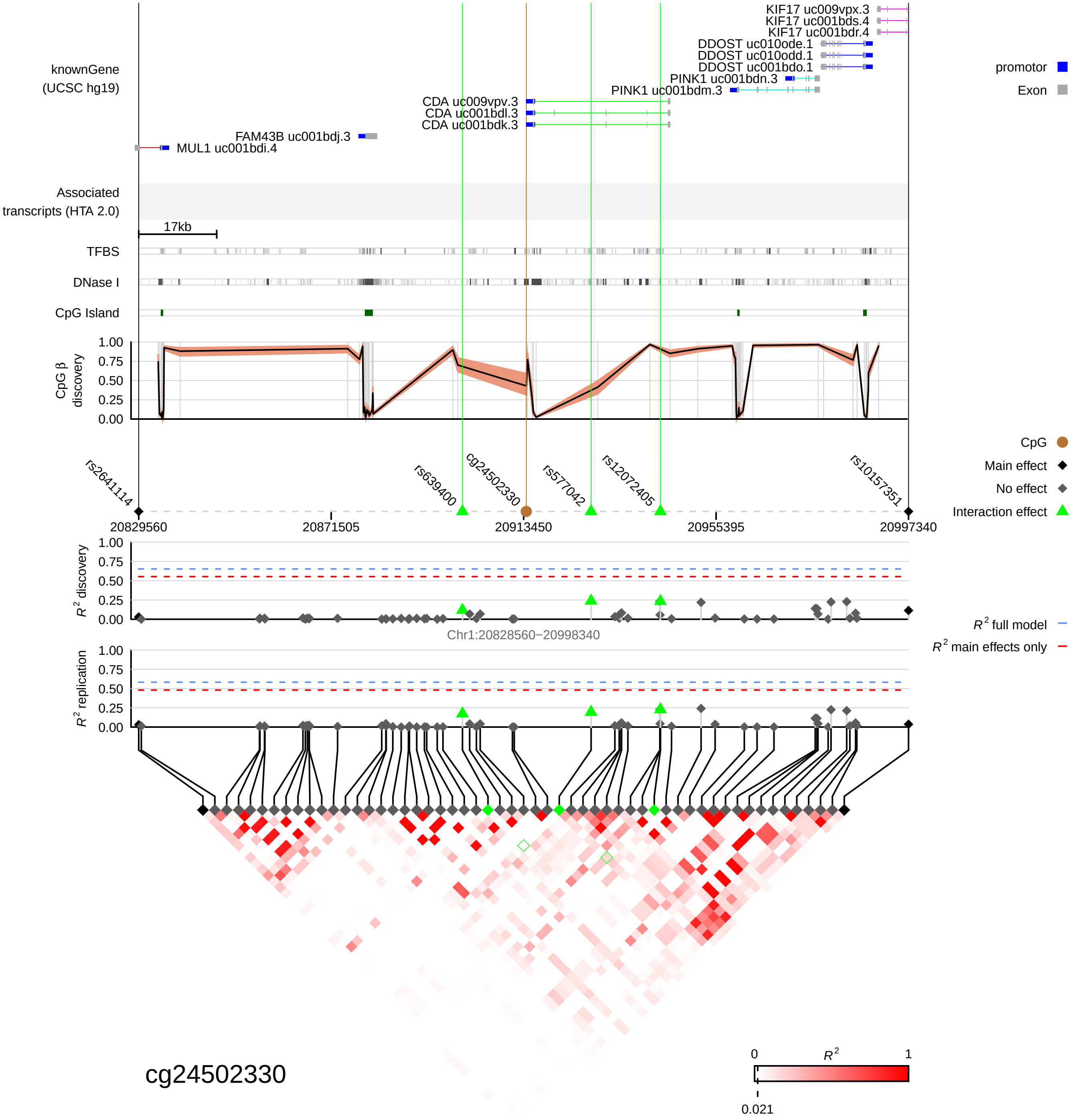

knownGene (UCSC hg19)
KIF17 uc009vpx.3
KIF17 uc001bds.4
KIF17 uc001bdr.4
DDOST uc010ode.1
DDOST uc010odd.1
DDOST uc001bdo.1
PINK1 uc001bdn.3
PINK1 uc001bdm.3
CDA uc009vpv.3
CDA uc001bdl.3
CDA uc001bdk.3
FAM43B uc001bdj.3
MUL1 uc001bdi.4
promotor
Exon
Associated transcripts (HTA 2.0)
17kb
TFBS
DNase I
CpG Island
CpG β discovery
1.00
0.75
0.50
0.25
0.00
rs2641114
rs639400
cg24502330
rs577042
rs12072405
rs10157351
20829560
20871505
20913450
20955395
20997340
CpG
Main effect
No effect
Interaction effect
R^2 discovery
Chr1:20828560−20998340
R^2 full model
R^2 main effects only
R^2 replication
0
R^2
1
0.021
cg24502330

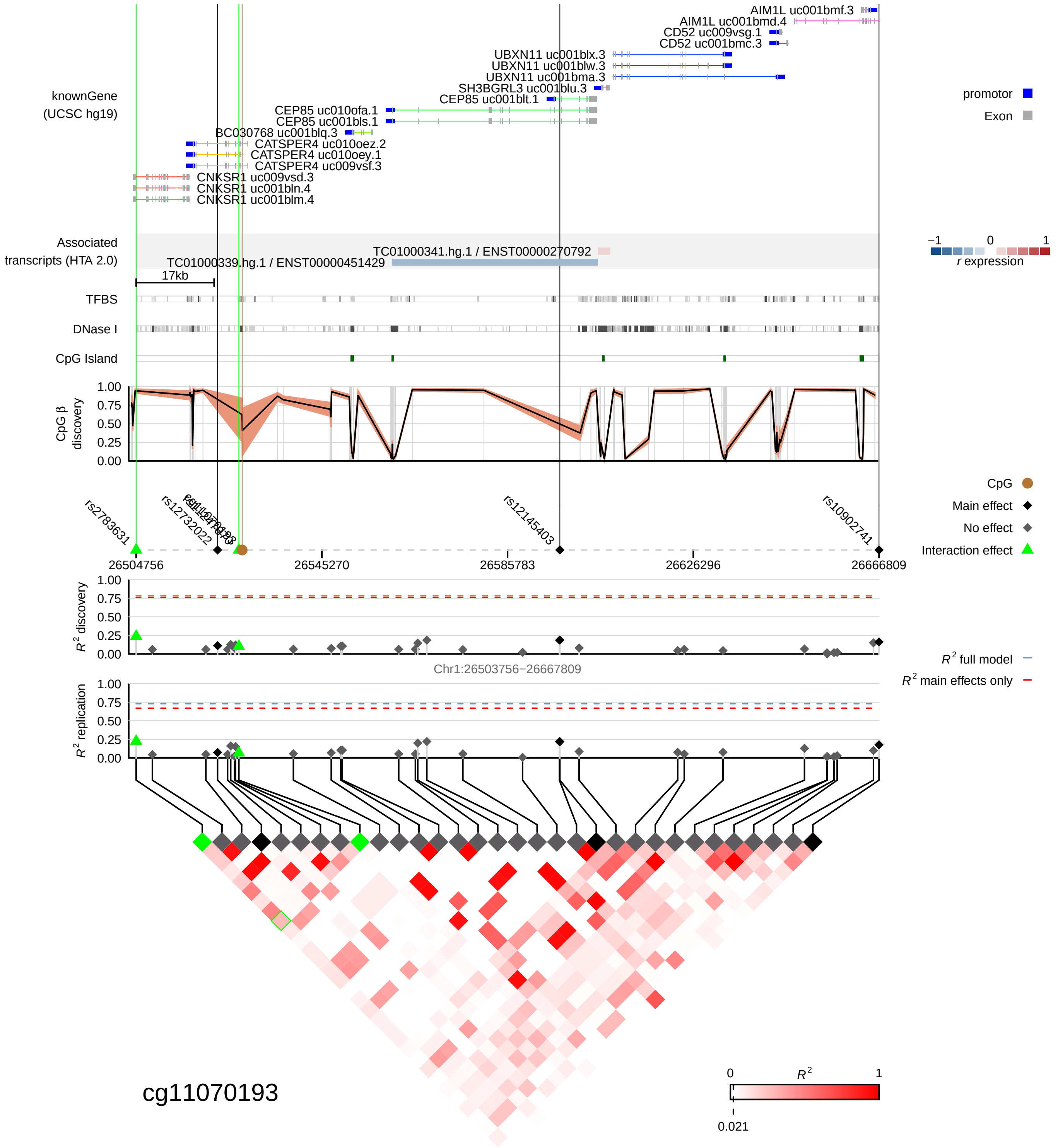
knownGene
(UCSC hg19)
AIM1L uc001bmf.3
AIM1L uc001bmd.4
CD52 uc009vsg.1
CD52 uc001bmc.3
UBXN11 uc001blx.3
UBXN11 uc001blw.3
UBXN11 uc001bma.3
SH3BGRL3 uc001blu.3
CEP85 uc001blt.1
CEP85 uc010ofa.1
CEP85 uc001bls.1
BC030768 uc001blq.3
CATSPER4 uc010oez.2
CATSPER4 uc010oey.1
CATSPER4 uc009vsf.3
CNKSR1 uc009vsd.3
CNKSR1 uc001bln.4
CNKSR1 uc001blm.4
promotor
Exon
Associated
transcripts (HTA 2.0)
TC01000341.hg.1 / ENST00000270792
TC01000339.hg.1 / ENST00000451429
−1
0
1
r expression
17kb
TFBS
DNase I
CpG Island
CpG β
discovery
1.00
0.75
0.50
0.25
0.00
rs2783631
rs12732022
rs12145403
rs10902741
CpG
Main effect
No effect
Interaction effect
26504756
26545270
26585783
26626296
26666809
R² discovery
Chr1:26503756−26667809
R² full model
R² main effects only
R² replication
0
R²
1
0.021
cg11070193

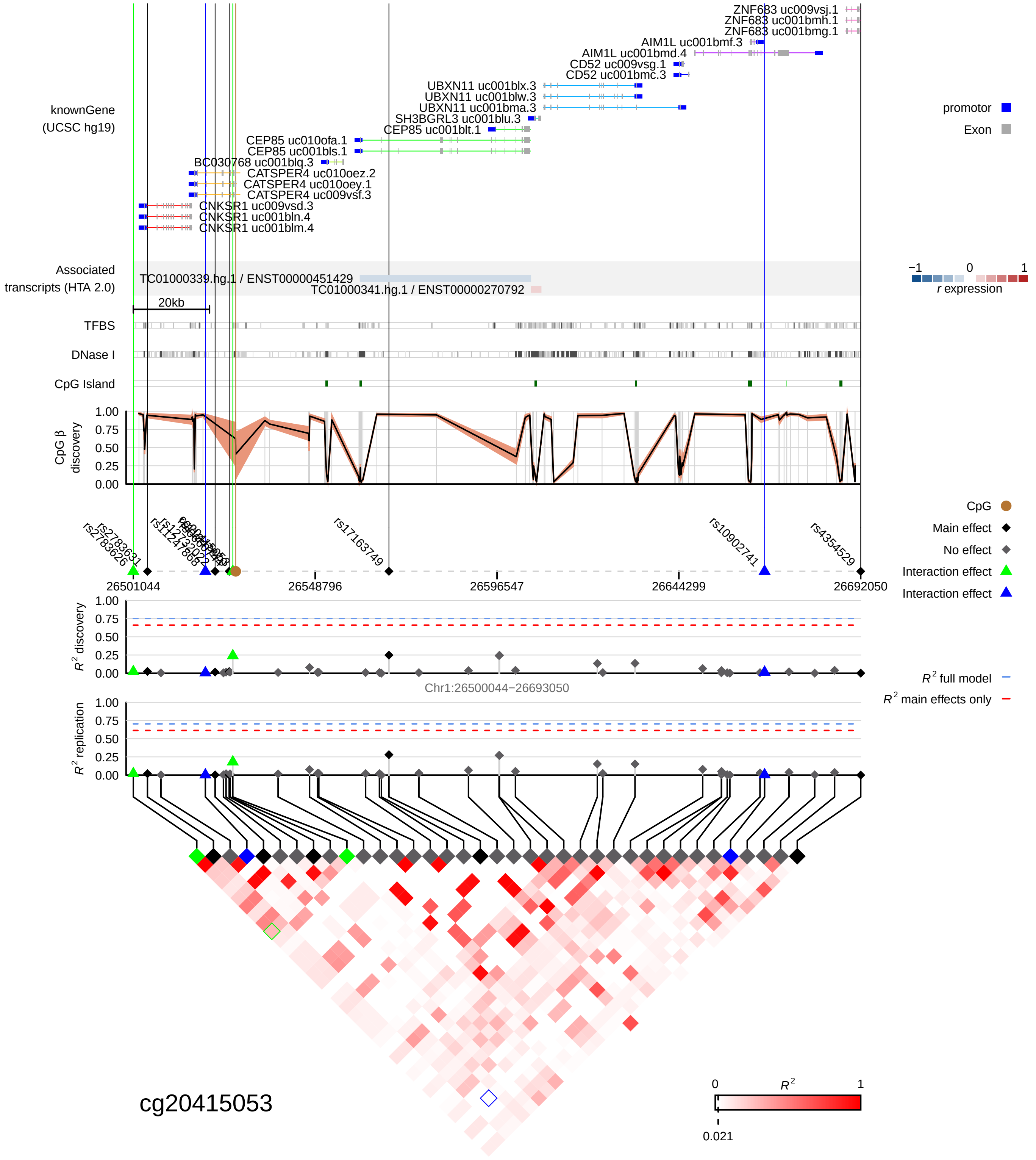

knownGene
(UCSC hg19)
ZNF683 uc009vsj.1
ZNF683 uc001bmh.1
ZNF683 uc001bmg.1
AIM1L uc001bmf.3
AIM1L uc001bmd.4
CD52 uc009vsg.1
CD52 uc001bmc.3
UBXN11 uc001blx.3
UBXN11 uc001blw.3
UBXN11 uc001bma.3
SH3BGRL3 uc001blu.3
CEP85 uc001blt.1
CEP85 uc010ofa.1
CEP85 uc001bls.1
BC030768 uc001blq.3
CATSPER4 uc010oez.2
CATSPER4 uc010oey.1
CATSPER4 uc009vsf.3
CNKSR1 uc009vsd.3
CNKSR1 uc001bln.4
CNKSR1 uc001blm.4
promotor
Exon
Associated
transcripts (HTA 2.0)
TC01000339.hg.1 / ENST00000451429
TC01000341.hg.1 / ENST00000270792
−1
0
1
r expression
20kb
TFBS
DNase I
CpG Island
CpG β
discovery
1.00
0.75
0.50
0.25
0.00
rs2783631
rs2783626
rs17163749
rs10902741
rs4354529
CpG
Main effect
No effect
Interaction effect
Interaction effect
26501044
26548796
26596547
26644299
26692050
R² discovery
Chr1:26500044−26693050
R² full model
R² main effects only
R² replication
0
R²
1
0.021
cg20415053

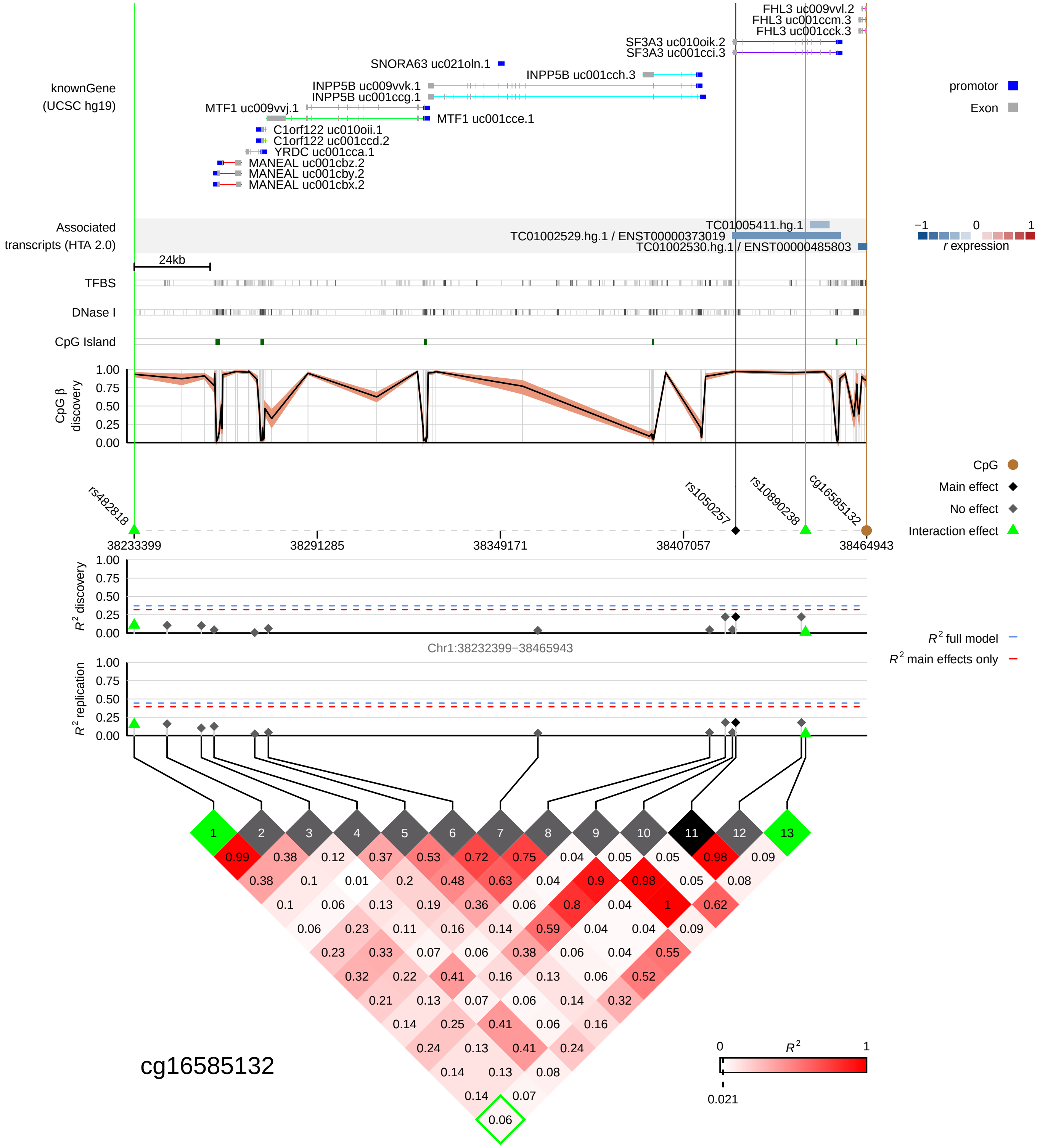
knownGene
(UCSC hg19)
FHL3 uc009vvl.2
FHL3 uc001ccm.3
FHL3 uc001cck.3
SF3A3 uc010oik.2
SF3A3 uc001cci.3
SNORA63 uc021oln.1
INPP5B uc001cch.3
INPP5B uc009vvk.1
INPP5B uc001ccg.1
MTF1 uc009vvj.1
MTF1 uc001cce.1
C1orf122 uc010oii.1
C1orf122 uc001ccd.2
YRDC uc001cca.1
MANEAL uc001cbz.2
MANEAL uc001cby.2
MANEAL uc001cbx.2
promotor
Exon
Associated
transcripts (HTA 2.0)
TC01005411.hg.1
TC01002529.hg.1 / ENST00000373019
TC01002530.hg.1 / ENST00000485803
−1
0
1
r expression
24kb
TFBS
DNase I
CpG Island
CpG β
discovery
1.00
0.75
0.50
0.25
0.00
rs482818
rs1050257
rs10890238
cg16585132
38233399
38291285
38349171
38407057
38464943
CpG
Main effect
No effect
Interaction effect
R2 discovery
Chr1:38232399−38465943
R2 full model
R2 main effects only
R2 replication
1
2
3
4
5
6
7
8
9
10
11
12
13
0.99 0.38 0.12 0.37 0.53 0.72 0.75 0.04 0.05 0.05 0.98 0.09
0.38 0.1 0.01 0.2 0.48 0.63 0.04 0.9 0.98 0.05 0.08
0.1 0.06 0.13 0.19 0.36 0.06 0.8 0.04 1 0.62
0.06 0.23 0.11 0.16 0.14 0.59 0.04 0.04 0.09
0.23 0.33 0.07 0.06 0.38 0.06 0.04 0.55
0.32 0.22 0.41 0.16 0.13 0.06 0.52
0.21 0.13 0.07 0.06 0.14 0.32
0.14 0.25 0.41 0.06 0.16
0.24 0.13 0.41 0.24
0.14 0.13 0.08
0.14 0.07
0.06
0
R2
1
0.021
cg16585132

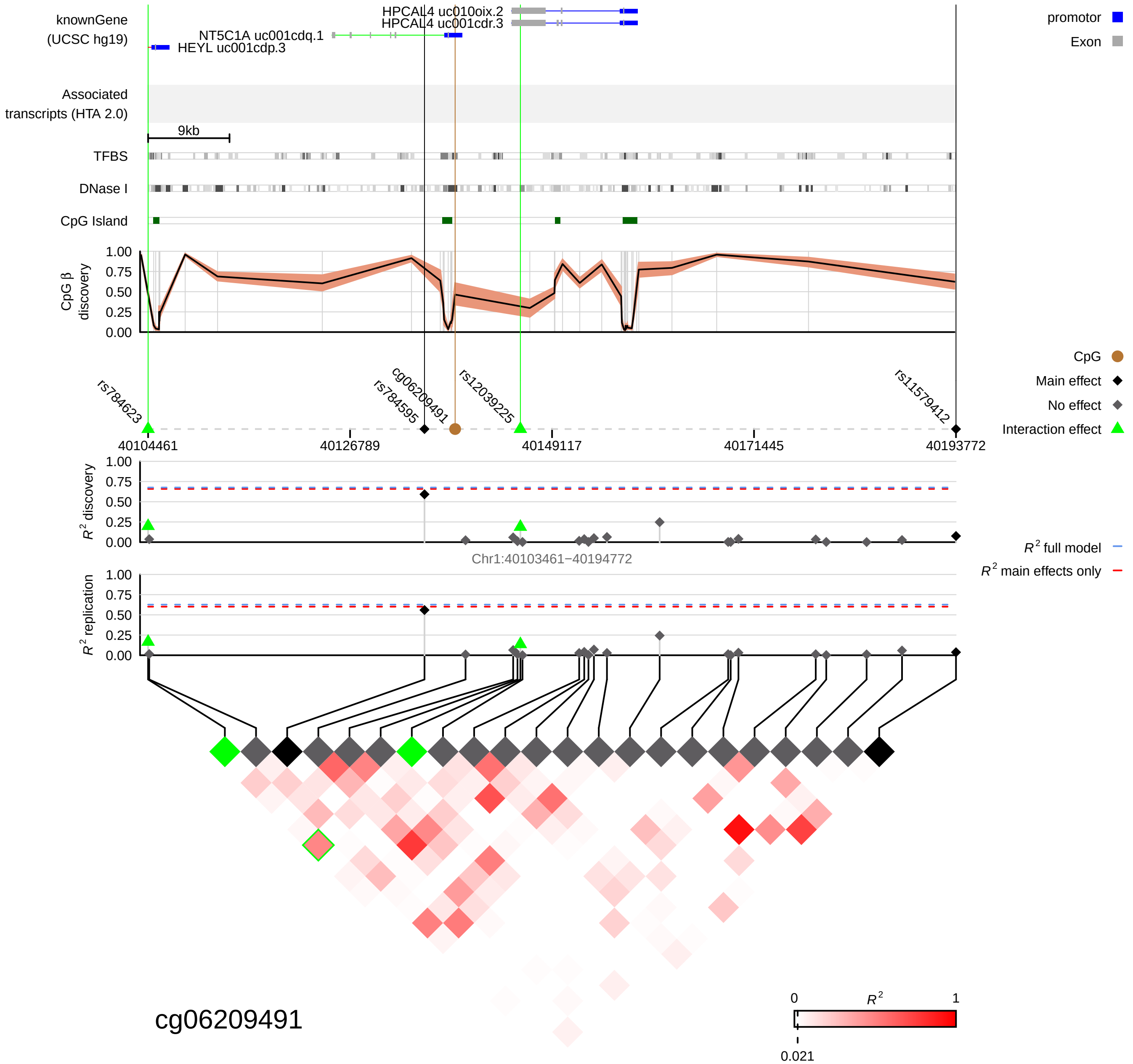
knownGene
(UCSC hg19)
HPCAL4 uc010oix.2
HPCAL4 uc001cdr.3
NT5C1A uc001cdq.1
HEYL uc001cdp.3
promotor
Exon
Associated
transcripts (HTA 2.0)
9kb
TFBS
DNase I
CpG Island
CpG β
discovery
1.00
0.75
0.50
0.25
0.00
rs784623
rs784595
cg06209491
rs12039225
rs11579412
CpG
Main effect
No effect
Interaction effect
40104461
40126789
40149117
40171445
40193772
R^2 discovery
Chr1:40103461−40194772
R^2 full model
R^2 main effects only
R^2 replication
0
R^2
1
0.021
cg06209491

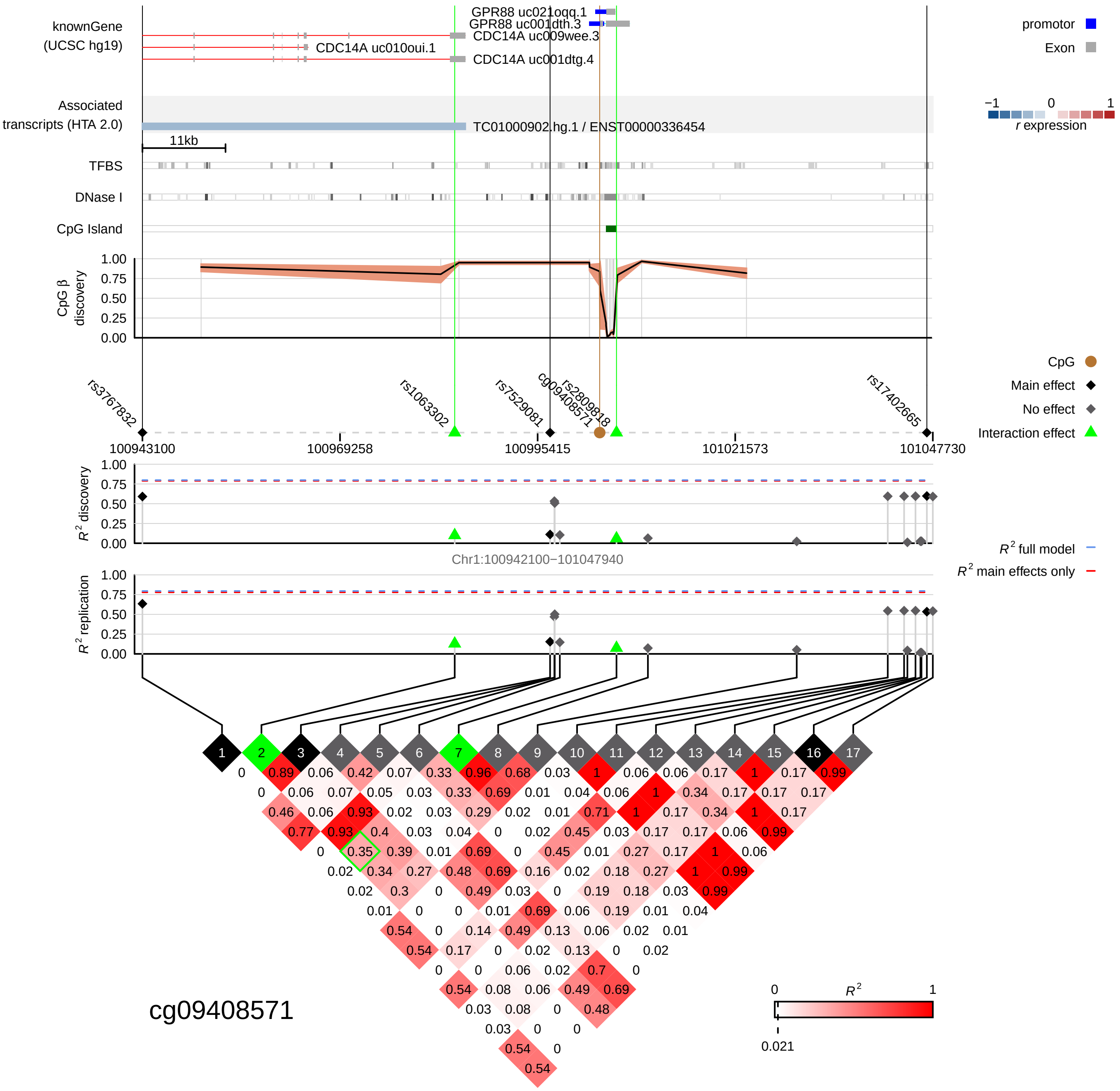
knownGene
(UCSC hg19)
GPR88 uc021oqq.1
GPR88 uc001dth.3
CDC14A uc009wee.3
CDC14A uc010oui.1
CDC14A uc001dtg.4
promotor
Exon
Associated
transcripts (HTA 2.0)
TC01000902.hg.1 / ENST00000336454
−1
0
1
r expression
11kb
TFBS
DNase I
CpG Island
CpG β
discovery
1.00
0.75
0.50
0.25
0.00
rs3767832
rs1063302
rs7529081
cg09408571
rs2809818
rs17402665
CpG
Main effect
No effect
Interaction effect
100943100
100969258
100995415
101021573
101047730
R² discovery
Chr1:100942100−101047940
R² full model
R² main effects only
R² replication
1
2
3
4
5
6
7
8
9
10
11
12
13
14
15
16
17
cg09408571
0
R²
1
0.021

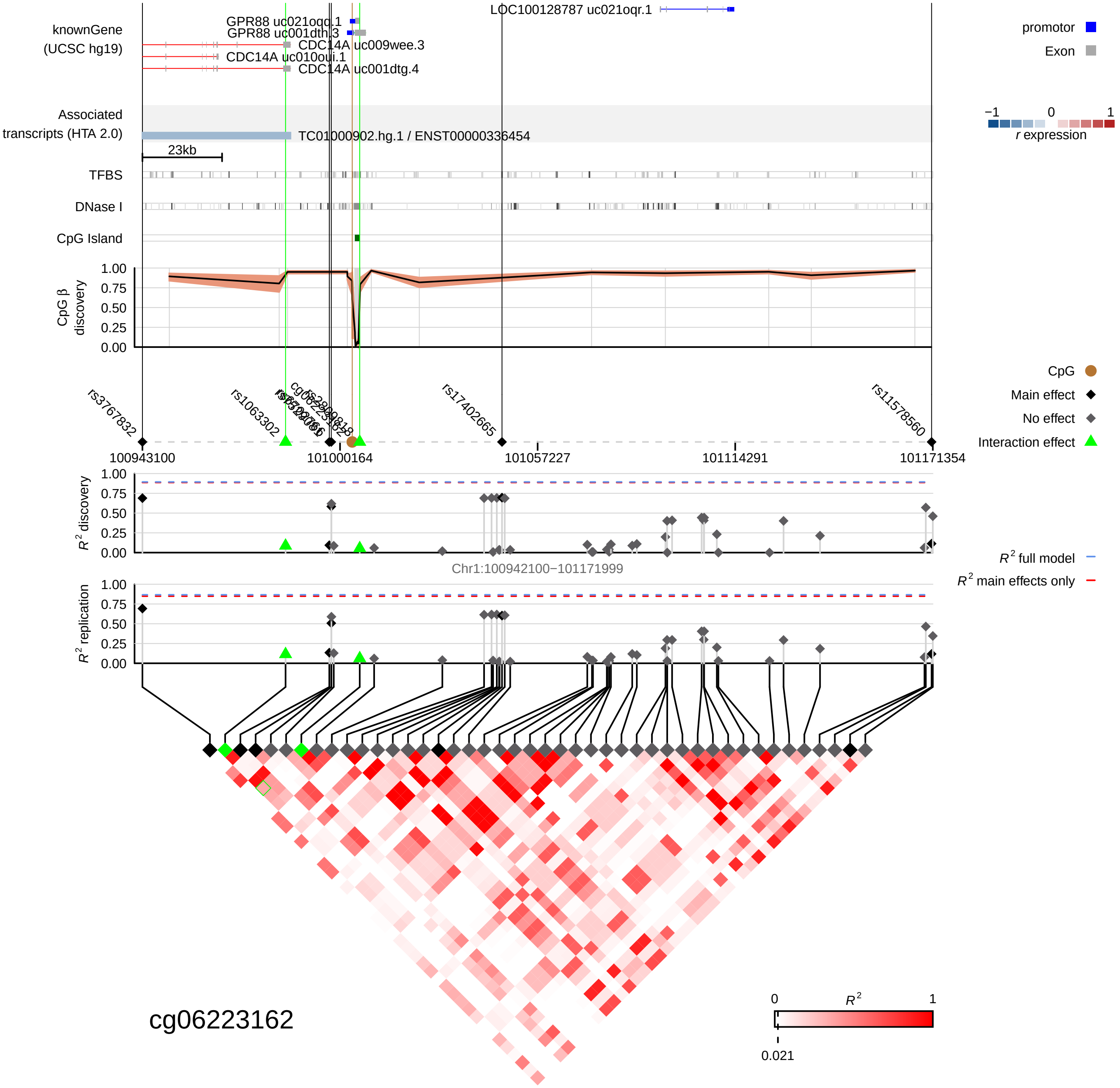

knownGene (UCSC hg19)
LOC100128787 uc021oqr.1
GPR88 uc021oqq.1
GPR88 uc001dth.3
CDC14A uc009wee.3
CDC14A uc010oui.1
CDC14A uc001dtg.4
promotor
Exon
Associated transcripts (HTA 2.0)
TC01000902.hg.1 / ENST00000336454
−1
0
1
r expression
23kb
TFBS
DNase I
CpG Island
CpG β discovery
1.00
0.75
0.50
0.25
0.00
rs3767832
rs1063302
rs17402665
rs11578560
CpG
Main effect
No effect
Interaction effect
100943100
101000164
101057227
101114291
101171354
R2 discovery
Chr1:100942100−101171999
R2 full model
R2 main effects only
R2 replication
0
R2
1
0.021
cg06223162

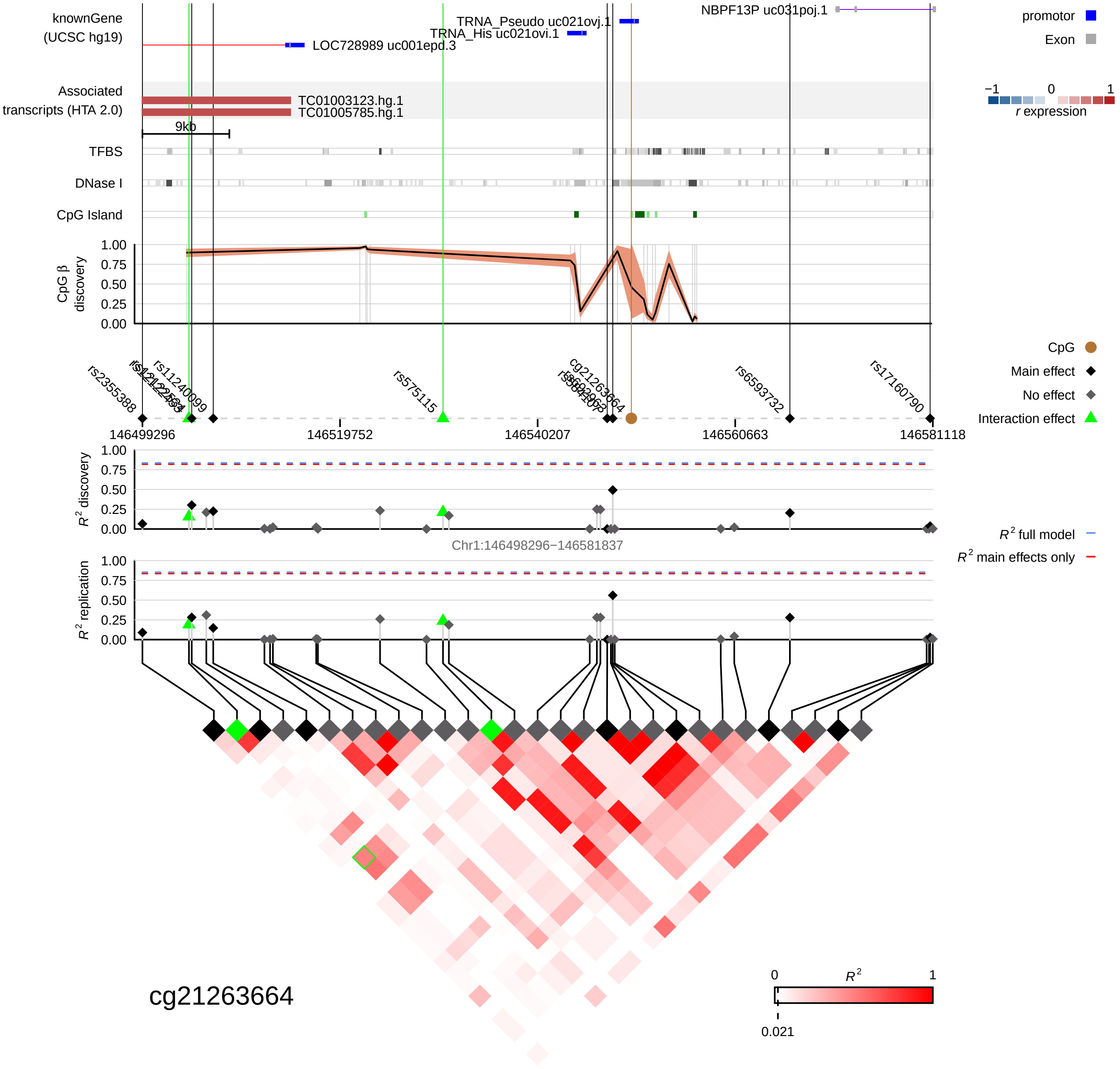
knownGene
(UCSC hg19)
NBPF13P uc031poj.1
TRNA_Pseudo uc021ovj.1
TRNA_His uc021ovi.1
LOC728989 uc001epd.3
promotor
Exon
Associated
transcripts (HTA 2.0)
TC01003123.hg.1
TC01005785.hg.1
−1
0
1
r expression
9kb
TFBS
DNase I
CpG Island
CpG β
discovery
1.00
0.75
0.50
0.25
0.00
rs2355388
rs11240099
rs575115
cg21263664
rs6593732
rs17160790
CpG
Main effect
No effect
Interaction effect
146499296
146519752
146540207
146560663
146581118
R² discovery
Chr1:146498296−146581837
R² full model
R² main effects only
R² replication
0
R²
1
0.021
cg21263664

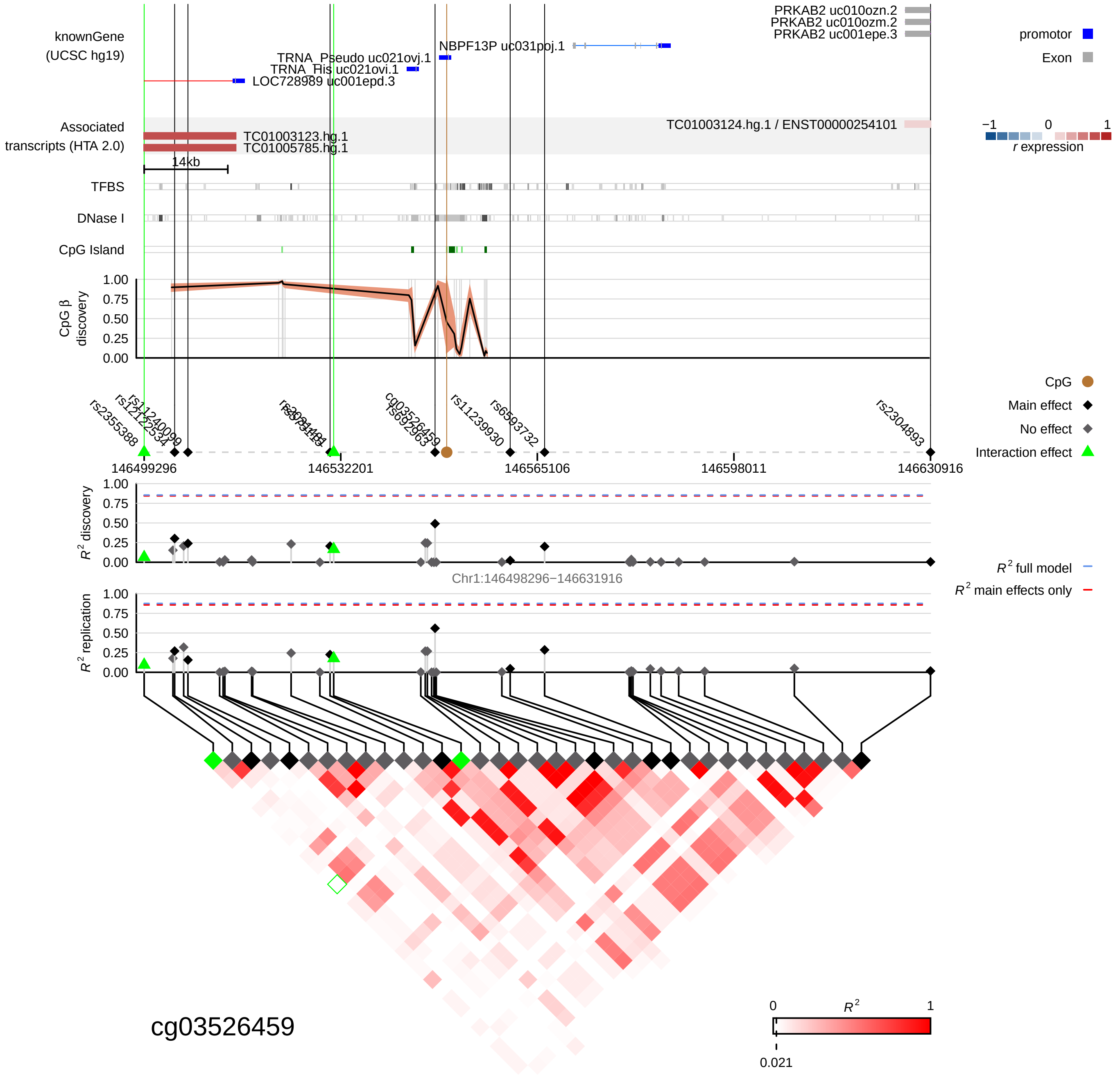
knownGene
(UCSC hg19)
PRKAB2 uc010ozn.2
PRKAB2 uc010ozm.2
PRKAB2 uc001epe.3
NBPF13P uc031poj.1
TRNA_Pseudo uc021ovj.1
TRNA_His uc021ovi.1
LOC728989 uc001epd.3
promotor
Exon
Associated
transcripts (HTA 2.0)
TC01003124.hg.1 / ENST00000254101
TC01003123.hg.1
TC01005785.hg.1
−1
0
1
r expression
14kb
TFBS
DNase I
CpG Island
CpG β
discovery
1.00
0.75
0.50
0.25
0.00
rs2355388
rs11240099
rs11239930
rs6593732
rs2304893
cg03526459
CpG
Main effect
No effect
Interaction effect
146499296
146532201
146565106
146598011
146630916
R^2 discovery
Chr1:146498296−146631916
R^2 full model
R^2 main effects only
R^2 replication
0
R^2
1
0.021
cg03526459

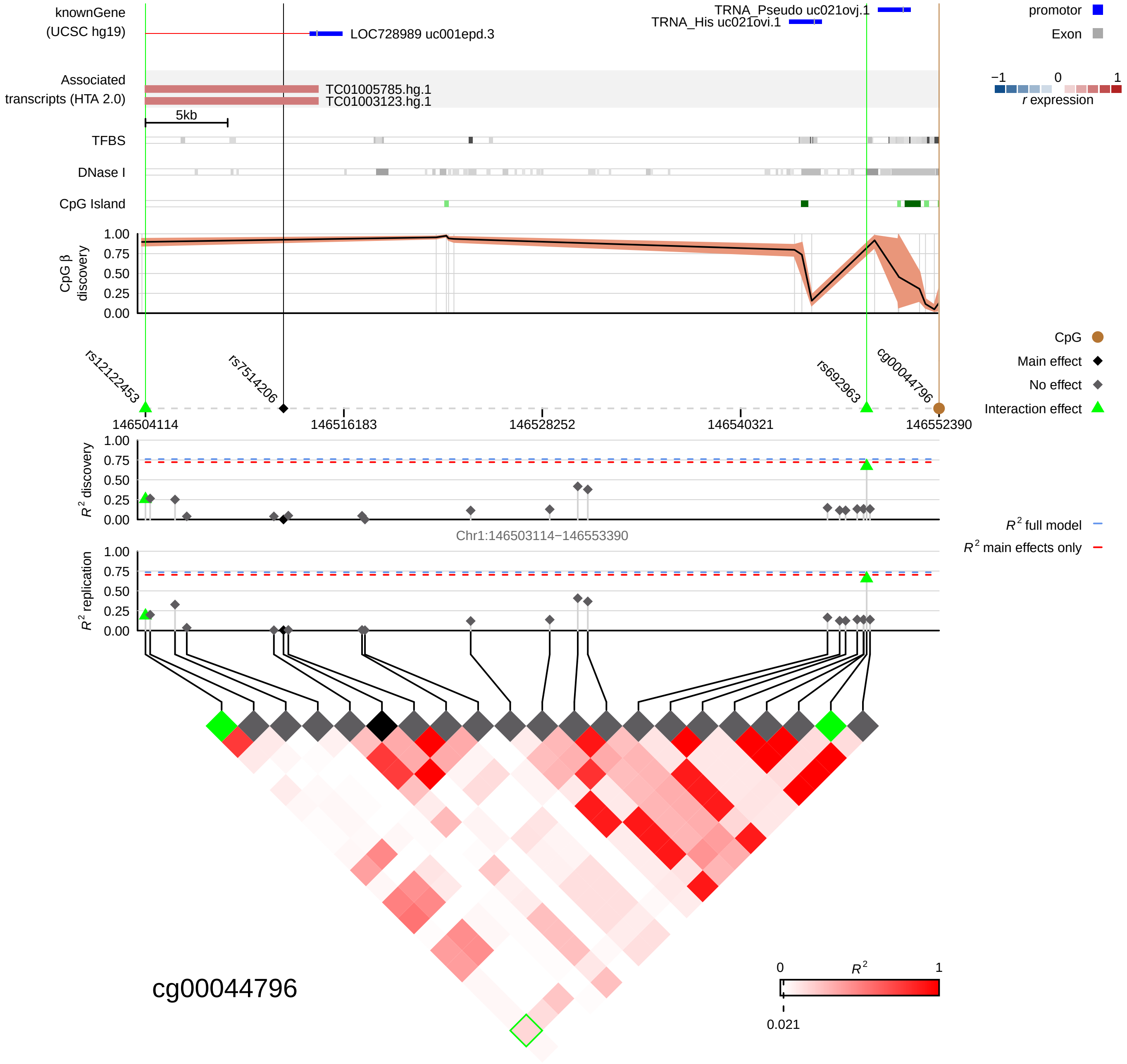

knownGene
(UCSC hg19)
LOC728989 uc001epd.3
TRNA_Pseudo uc021ovj.1
TRNA_His uc021ovi.1
promotor
Exon
Associated
transcripts (HTA 2.0)
TC01005785.hg.1
TC01003123.hg.1
−1
0
1
r expression
5kb
TFBS
DNase I
CpG Island
CpG β
discovery
1.00
0.75
0.50
0.25
0.00
rs12122453
rs7514206
rs692963
cg00044796
146504114
146516183
146528252
146540321
146552390
CpG
Main effect
No effect
Interaction effect
R2 discovery
Chr1:146503114−146553390
R2 full model
R2 main effects only
R2 replication
cg00044796
0
R2
1
0.021

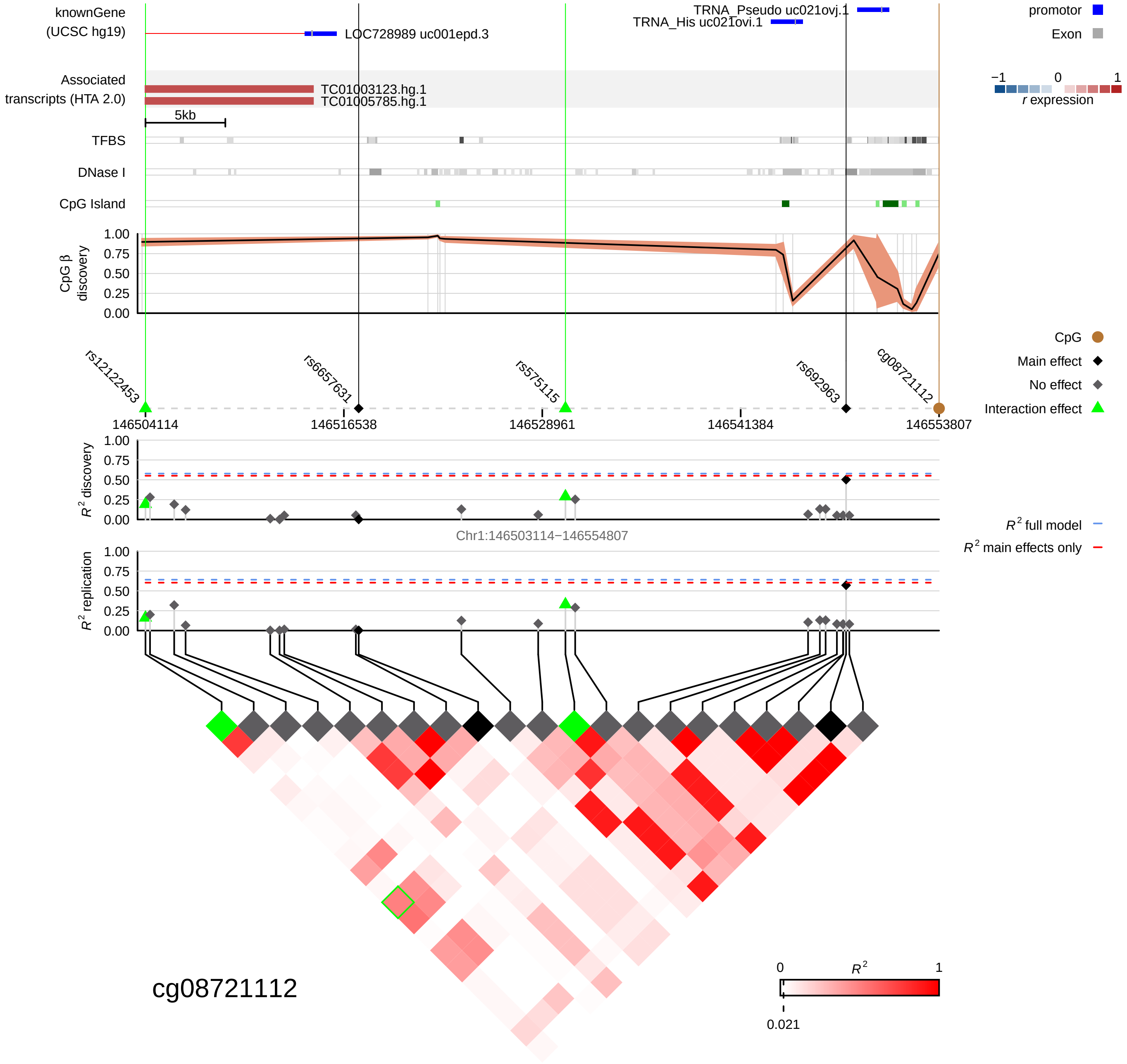
knownGene (UCSC hg19)
LOC728989 uc001epd.3
TRNA_Pseudo uc021ovj.1
TRNA_His uc021ovi.1
promotor
Exon
Associated transcripts (HTA 2.0)
TC01003123.hg.1
TC01005785.hg.1
−1 0 1
r expression
5kb
TFBS
DNase I
CpG Island
CpG β discovery
rs12122453
rs6657631
rs575115
rs692963
cg08721112
CpG
Main effect
No effect
Interaction effect
146504114
146516538
146528961
146541384
146553807
R^2 discovery
Chr1:146503114−146554807
R^2 full model
R^2 main effects only
R^2 replication
R^2
0.021
cg08721112

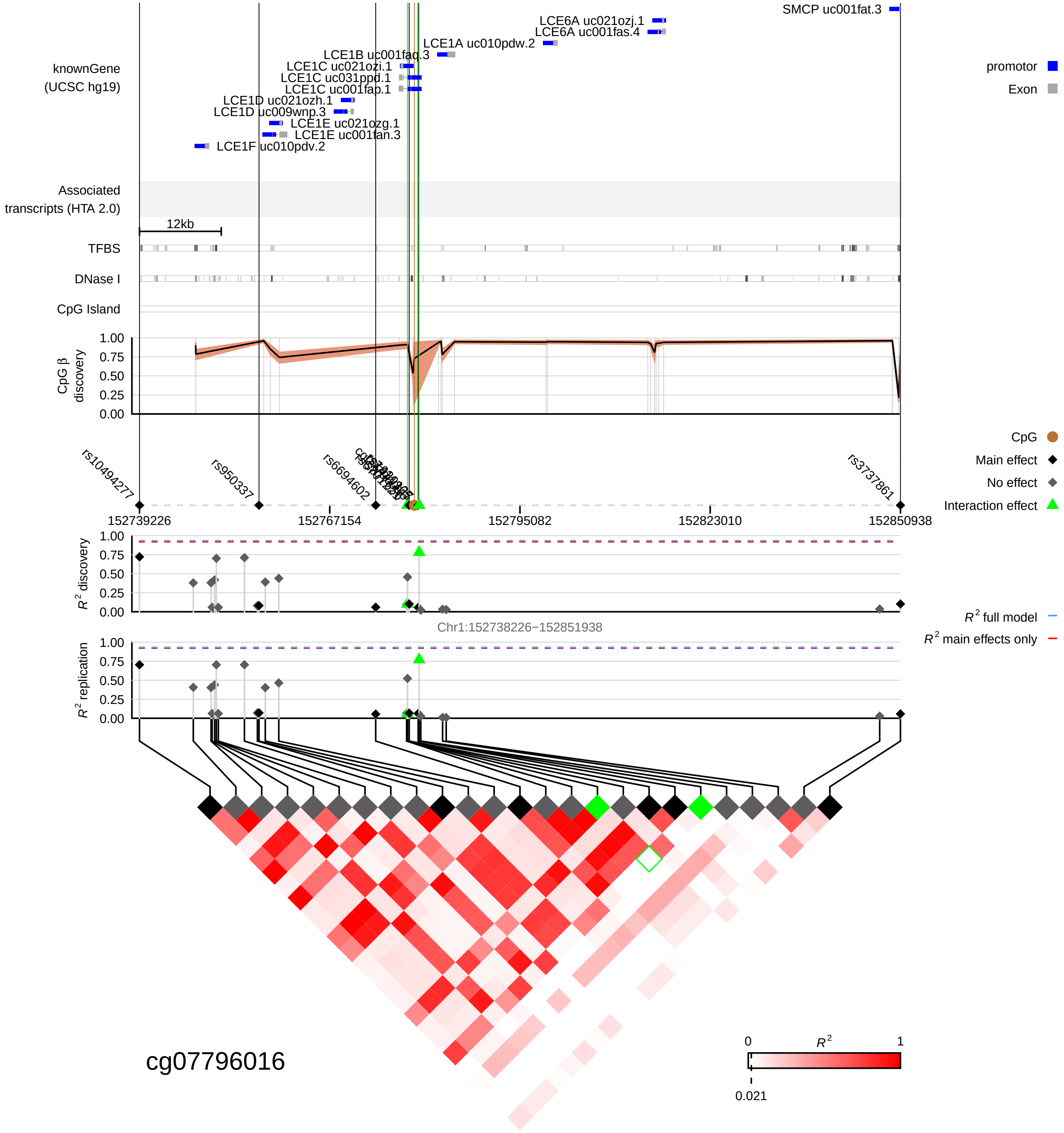

SMCP uc001fat.3
LCE6A uc021ozj.1
LCE6A uc001fas.4
LCE1A uc010pdw.2
LCE1B uc001faq.3
LCE1C uc021ozi.1
LCE1C uc031ppd.1
LCE1C uc001fap.1
LCE1D uc021ozh.1
LCE1D uc009wnp.3
LCE1E uc021ozg.1
LCE1E uc001fan.3
LCE1F uc010pdv.2
knownGene
(UCSC hg19)
promotor
Exon
Associated
transcripts (HTA 2.0)
12kb
TFBS
DNase I
CpG Island
CpG β
discovery
1.00
0.75
0.50
0.25
0.00
CpG
Main effect
No effect
Interaction effect
rs10494277
rs950337
rs6694602
rs3737861
152739226
152767154
152795082
152823010
152850938
R^2 discovery
R^2 full model
R^2 main effects only
Chr1:152738226−152851938
R^2 replication
0
R^2
1
0.021
cg07796016

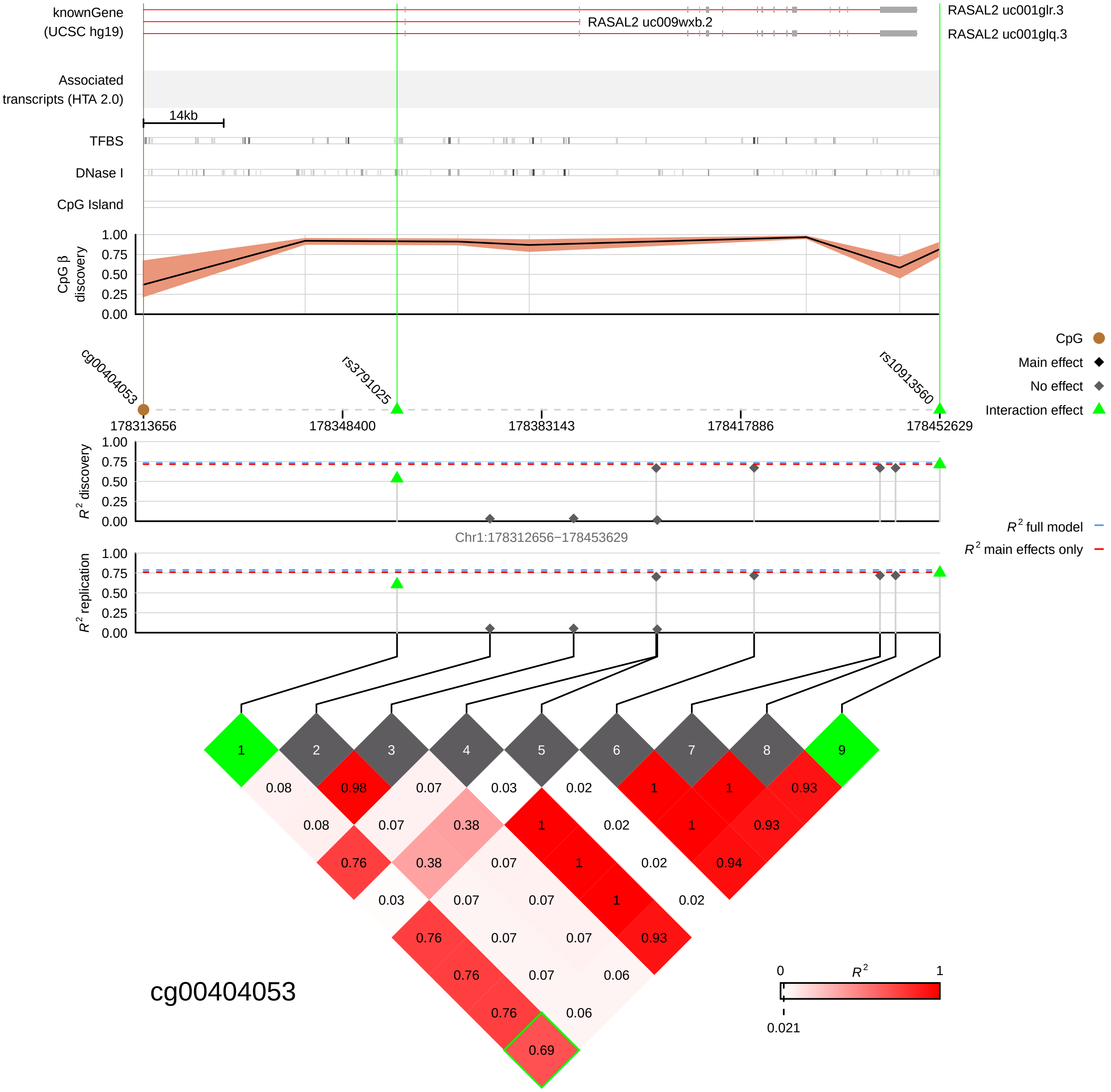
knownGene (UCSC hg19)
RASAL2 uc001glr.3
RASAL2 uc009wxb.2
RASAL2 uc001glq.3
Associated transcripts (HTA 2.0)
14kb
TFBS
DNase I
CpG Island
CpG β discovery
cg00404053
rs3791025
rs10913560
178313656
178348400
178383143
178417886
178452629
CpG
Main effect
No effect
Interaction effect
R^2 discovery
Chr1:178312656−178453629
R^2 full model
R^2 main effects only
R^2 replication
0
R^2
1
0.021
cg00404053

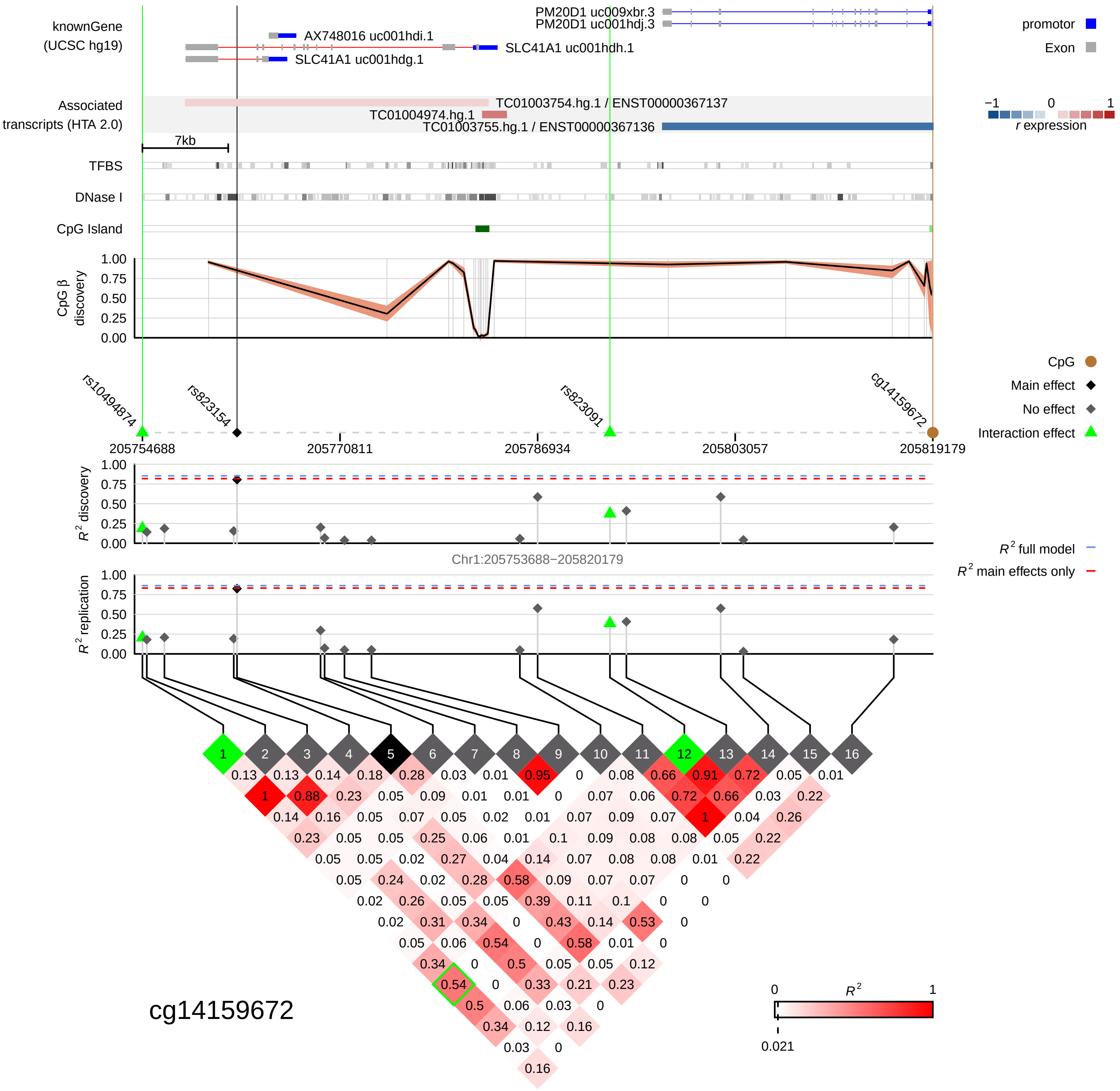

knownGene (UCSC hg19)
PM20D1 uc009xbr.3
PM20D1 uc001hdj.3
AX748016 uc001hdi.1
SLC41A1 uc001hdh.1
SLC41A1 uc001hdg.1
promotor
Exon
Associated transcripts (HTA 2.0)
TC01003754.hg.1 / ENST00000367137
TC01004974.hg.1
TC01003755.hg.1 / ENST00000367136
−1 0 1
r expression
7kb
TFBS
DNase I
CpG Island
CpG β discovery
1.00 0.75 0.50 0.25 0.00
rs10494874
rs823154
rs823091
cg14159672
CpG
Main effect
No effect
Interaction effect
205754688
205770811
205786934
205803057
205819179
R2 discovery
Chr1:205753688−205820179
R2 full model
R2 main effects only
R2 replication
1 2 3 4 5 6 7 8 9 10 11 12 13 14 15 16
0 R2 1
0.021
cg14159672

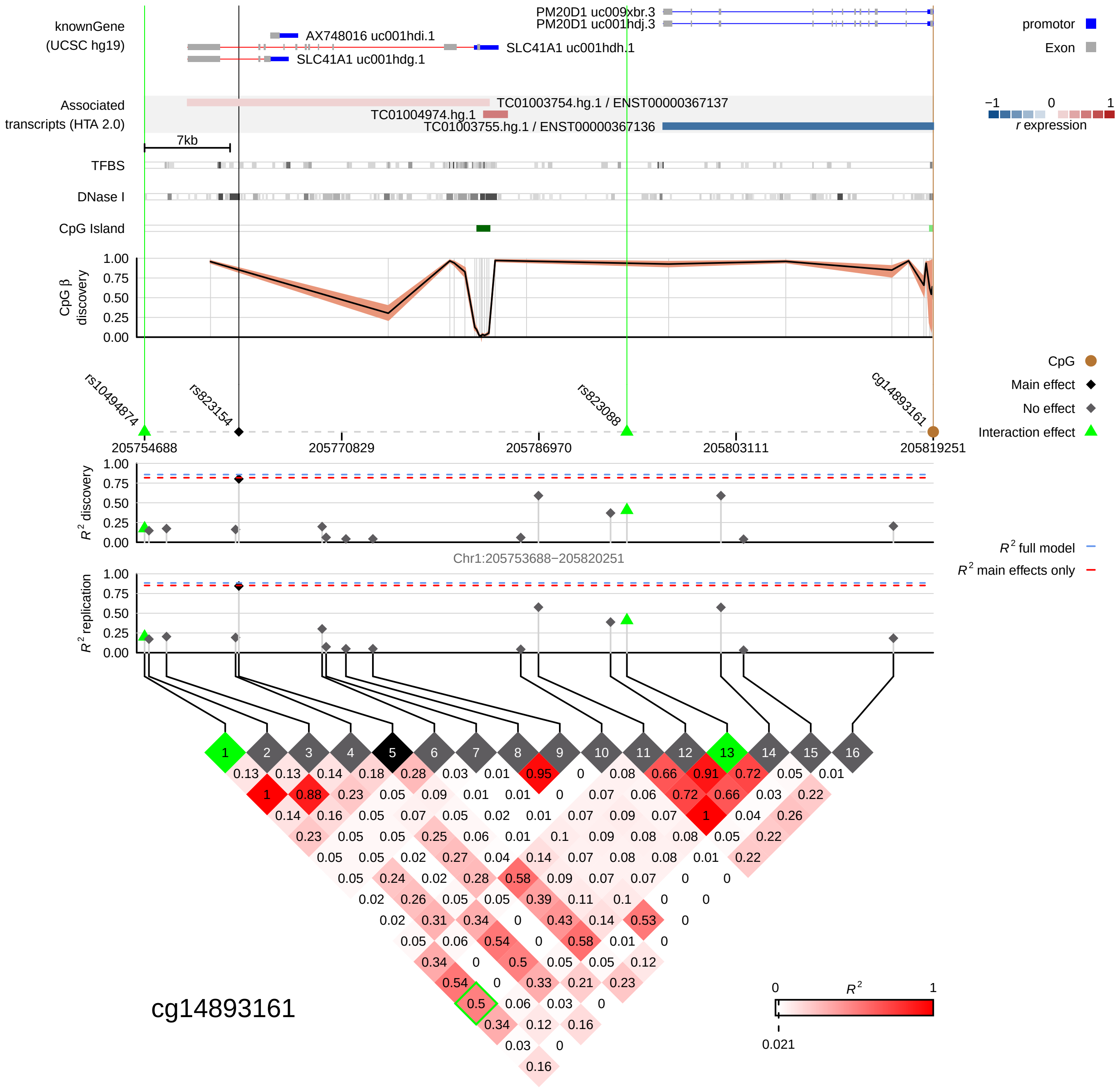
knownGene (UCSC hg19)
PM20D1 uc009xbr.3
PM20D1 uc001hdj.3
AX748016 uc001hdi.1
SLC41A1 uc001hdh.1
SLC41A1 uc001hdg.1
promotor
Exon
Associated transcripts (HTA 2.0)
TC01003754.hg.1 / ENST00000367137
TC01004974.hg.1
TC01003755.hg.1 / ENST00000367136
−1
0
1
r expression
7kb
TFBS
DNase I
CpG Island
CpG β discovery
1.00
0.75
0.50
0.25
0.00
rs10494874
rs823154
rs823088
cg14893161
CpG
Main effect
No effect
Interaction effect
205754688
205770829
205786970
205803111
205819251
R² discovery
Chr1:205753688−205820251
R² full model
R² main effects only
R² replication
1
2
3
4
5
6
7
8
9
10
11
12
13
14
15
16
0
R²
1
0.021
cg14893161

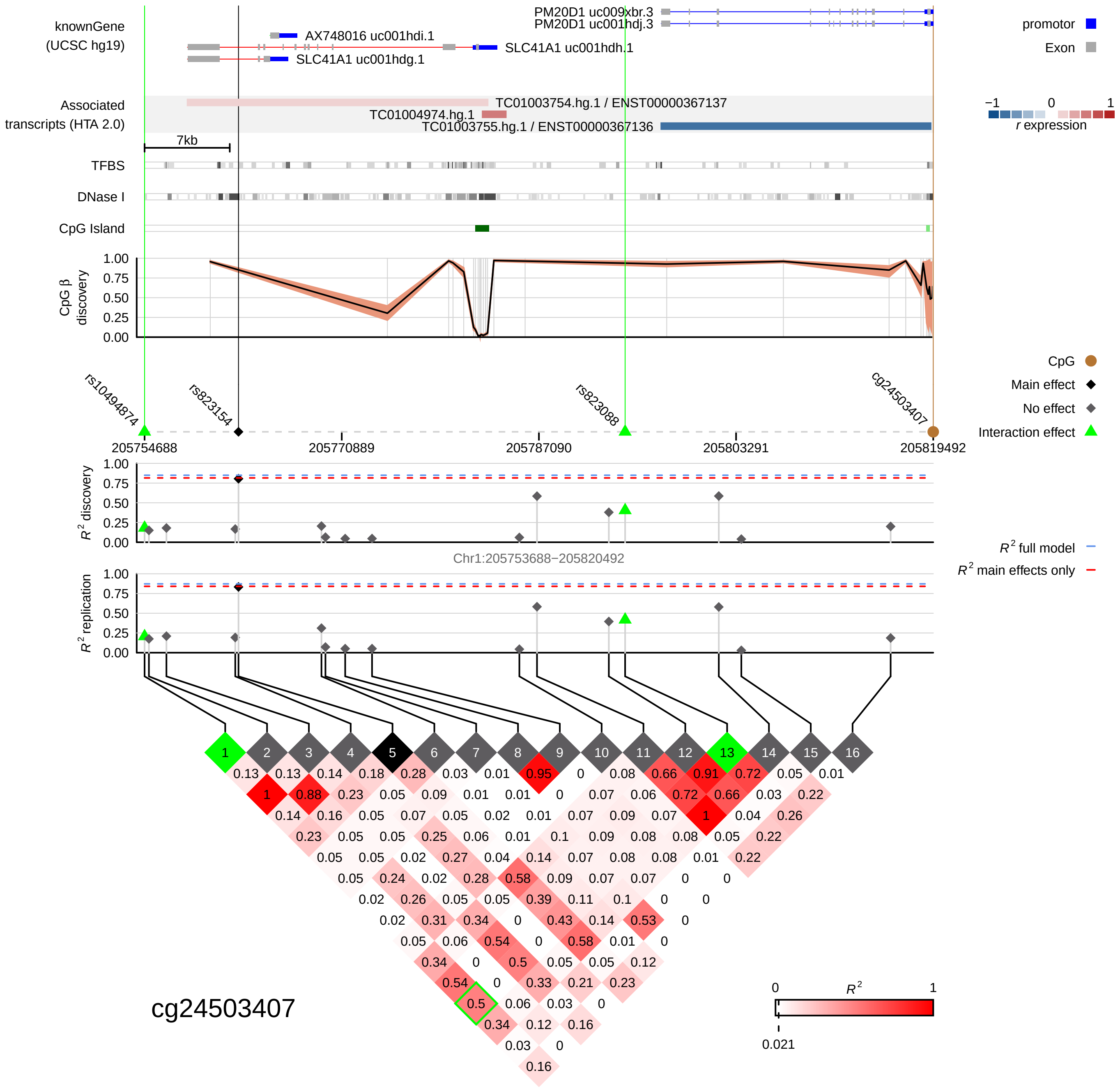

knownGene
(UCSC hg19)
PM20D1 uc009xbr.3
PM20D1 uc001hdj.3
AX748016 uc001hdi.1
SLC41A1 uc001hdh.1
SLC41A1 uc001hdg.1
promotor
Exon
Associated
transcripts (HTA 2.0)
TC01003754.hg.1 / ENST00000367137
TC01004974.hg.1
TC01003755.hg.1 / ENST00000367136
−1
0
1
r expression
7kb
TFBS
DNase I
CpG Island
CpG β
discovery
1.00
0.75
0.50
0.25
0.00
rs10494874
rs823154
rs823088
cg24503407
CpG
Main effect
No effect
Interaction effect
205754688
205770889
205787090
205803291
205819492
R² discovery
Chr1:205753688−205820492
R² full model
R² main effects only
R² replication
1
2
3
4
5
6
7
8
9
10
11
12
13
14
15
16
cg24503407
0
R²
1
0.021

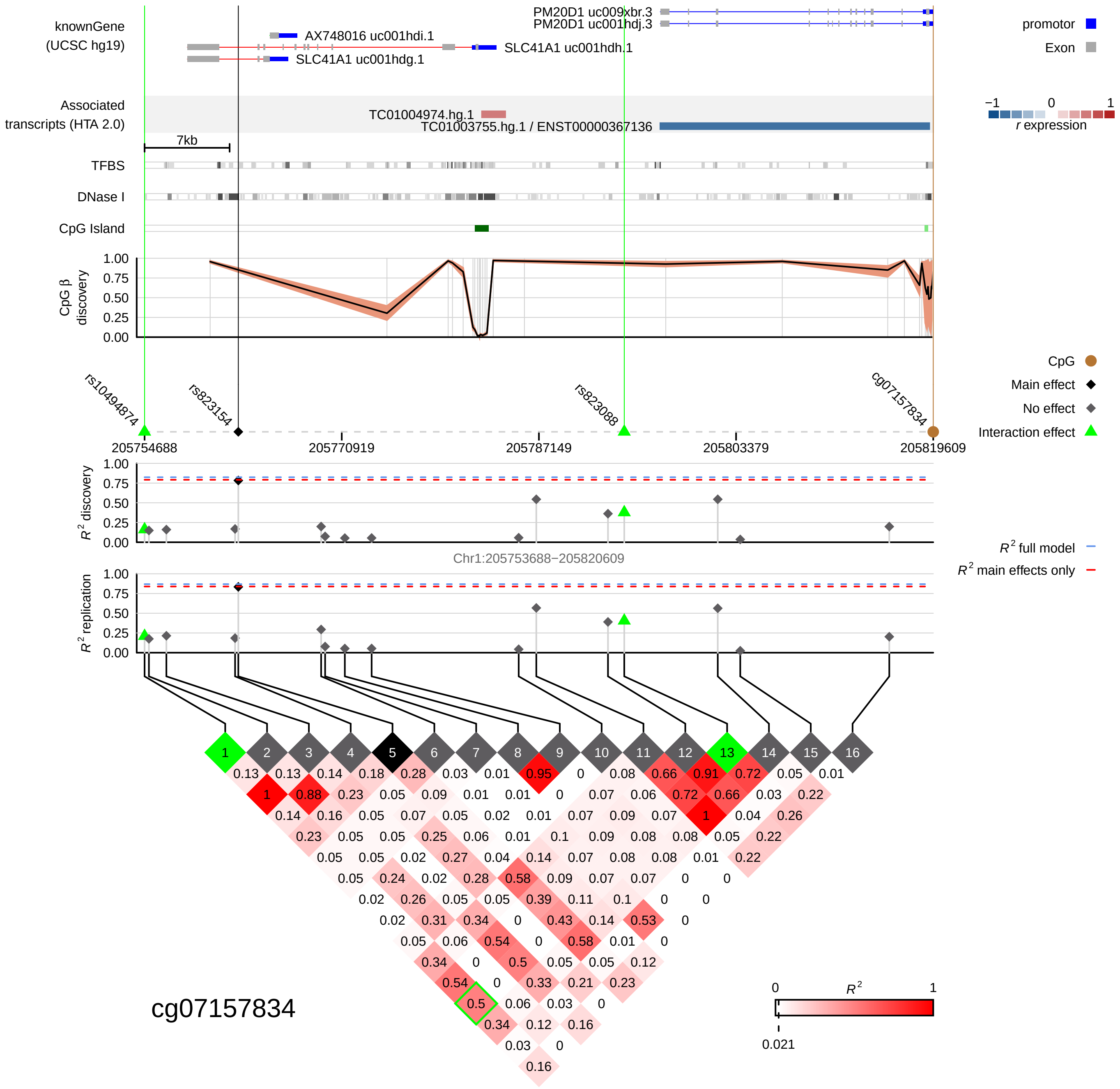

knownGene (UCSC hg19)
PM20D1 uc009xbr.3
PM20D1 uc001hdj.3
AX748016 uc001hdi.1
SLC41A1 uc001hdh.1
SLC41A1 uc001hdg.1
promotor
Exon
Associated transcripts (HTA 2.0)
TC01004974.hg.1
TC01003755.hg.1 / ENST00000367136
r expression
7kb
TFBS
DNase I
CpG Island
CpG β discovery
rs10494874
rs823154
rs823088
cg07157834
205754688
205770919
205787149
205803379
205819609
CpG
Main effect
No effect
Interaction effect
R² discovery
Chr1:205753688−205820609
R² full model
R² main effects only
R² replication
cg07157834
0.021

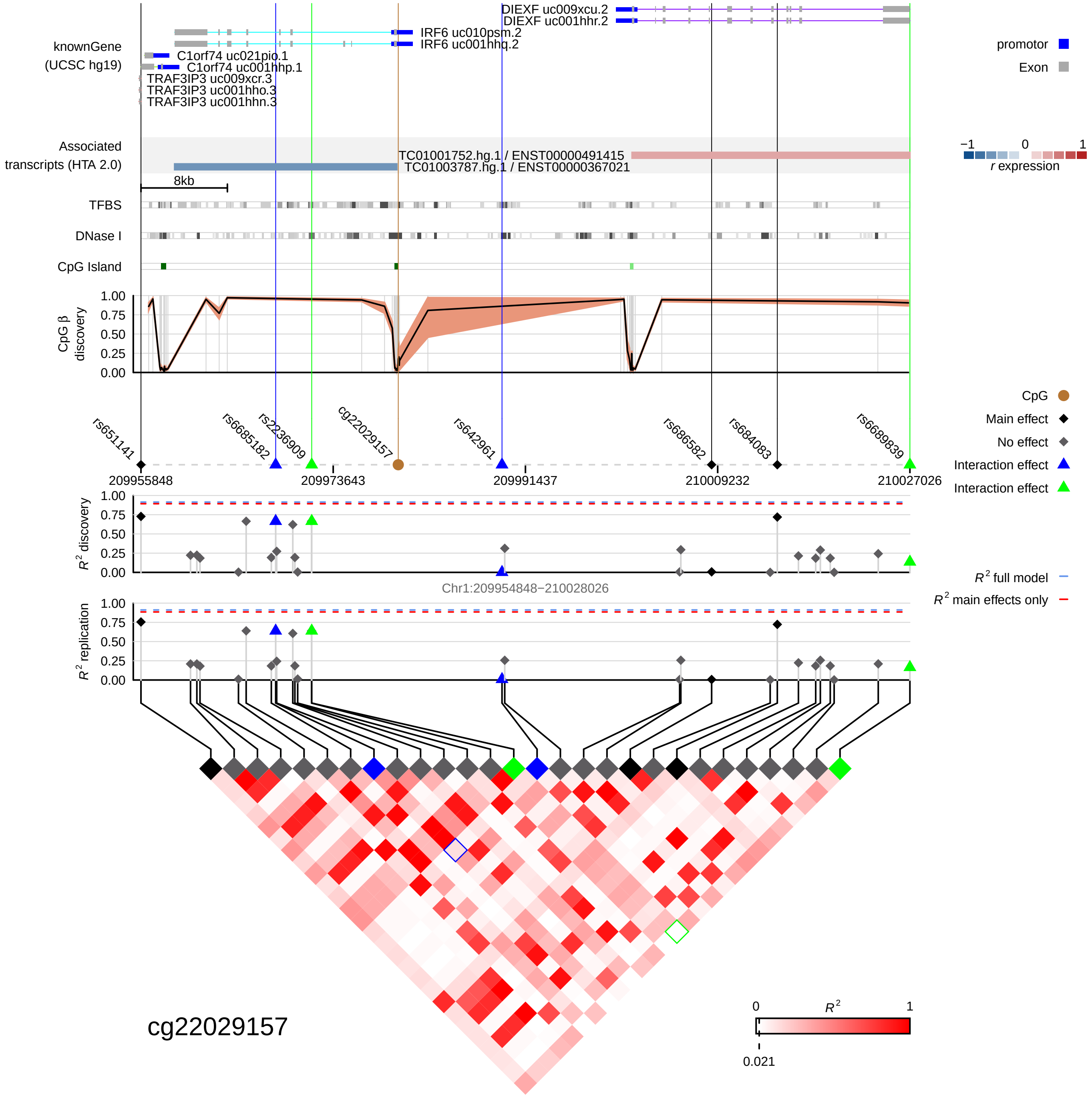

knownGene
(UCSC hg19)
DIEXF uc009xcu.2
DIEXF uc001hhr.2
IRF6 uc010psm.2
IRF6 uc001hhq.2
C1orf74 uc021pio.1
C1orf74 uc001hhp.1
TRAF3IP3 uc009xcr.3
TRAF3IP3 uc001hho.3
TRAF3IP3 uc001hhn.3
promotor
Exon
Associated
transcripts (HTA 2.0)
TC01001752.hg.1 / ENST00000491415
TC01003787.hg.1 / ENST00000367021
r expression
8kb
TFBS
DNase I
CpG Island
CpG β
discovery
rs651141
rs6685182
rs2236909
cg22029157
rs642961
rs686582
rs684083
rs6689839
209955848
209973643
209991437
210009232
210027026
CpG
Main effect
No effect
Interaction effect
Interaction effect
R^2 discovery
Chr1:209954848−210028026
R^2 full model
R^2 main effects only
R^2 replication
cg22029157
R^2
0.021

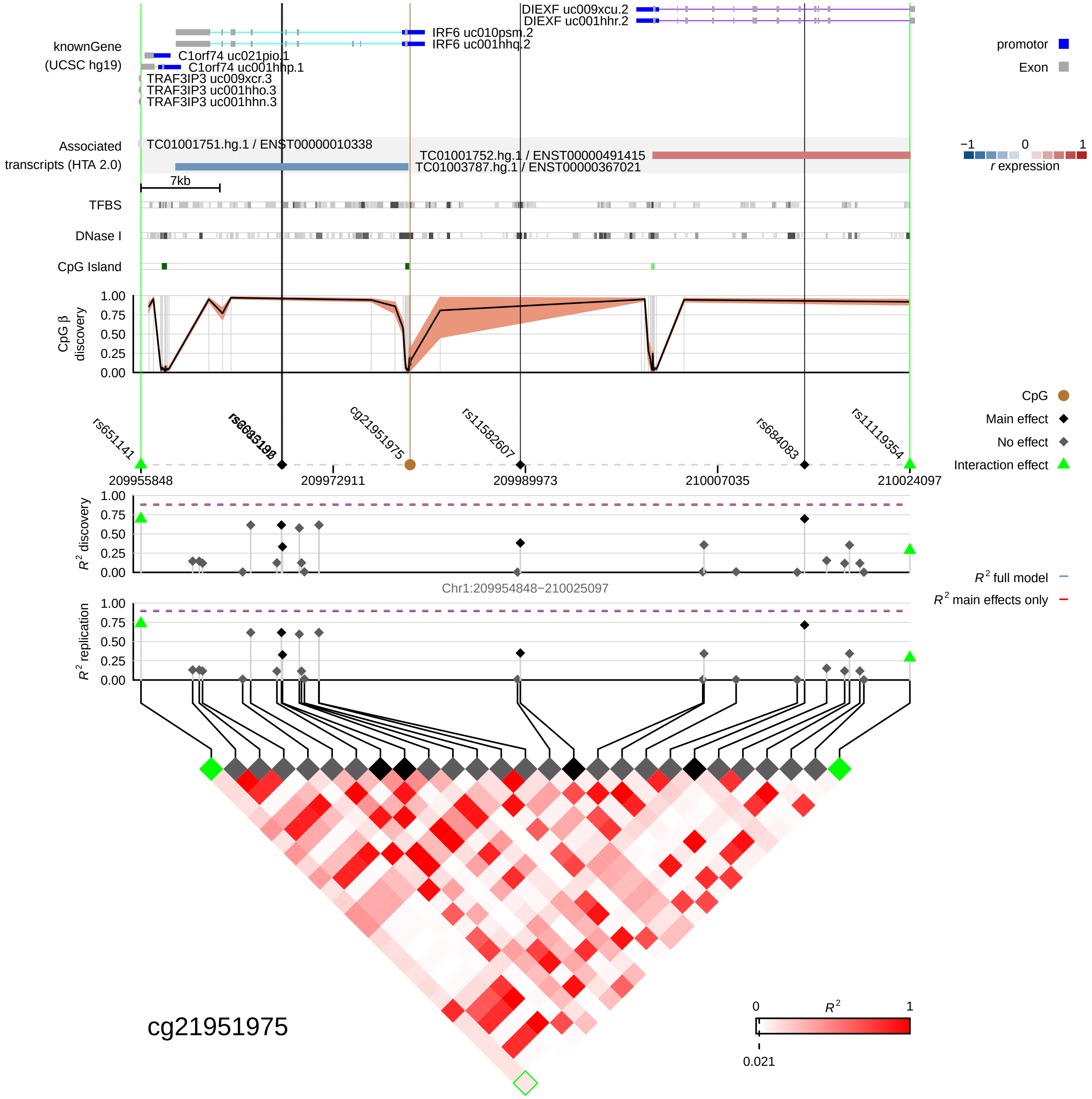

knownGene
(UCSC hg19)
DIEXF uc009xcu.2
DIEXF uc001hhr.2
IRF6 uc010psm.2
IRF6 uc001hhq.2
C1orf74 uc021pio.1
C1orf74 uc001hhp.1
TRAF3IP3 uc009xcr.3
TRAF3IP3 uc001hho.3
TRAF3IP3 uc001hhn.3
promotor
Exon
Associated
transcripts (HTA 2.0)
TC01001751.hg.1 / ENST00000010338
TC01001752.hg.1 / ENST00000491415
TC01003787.hg.1 / ENST00000367021
r expression
7kb
TFBS
DNase I
CpG Island
CpG β
discovery
rs651141
cg21951975
rs11582607
rs684083
rs11119354
209955848
209972911
209989973
210007035
210024097
CpG
Main effect
No effect
Interaction effect
R² discovery
Chr1:209954848−210025097
R² full model
R² main effects only
R² replication
R²
0.021
cg21951975

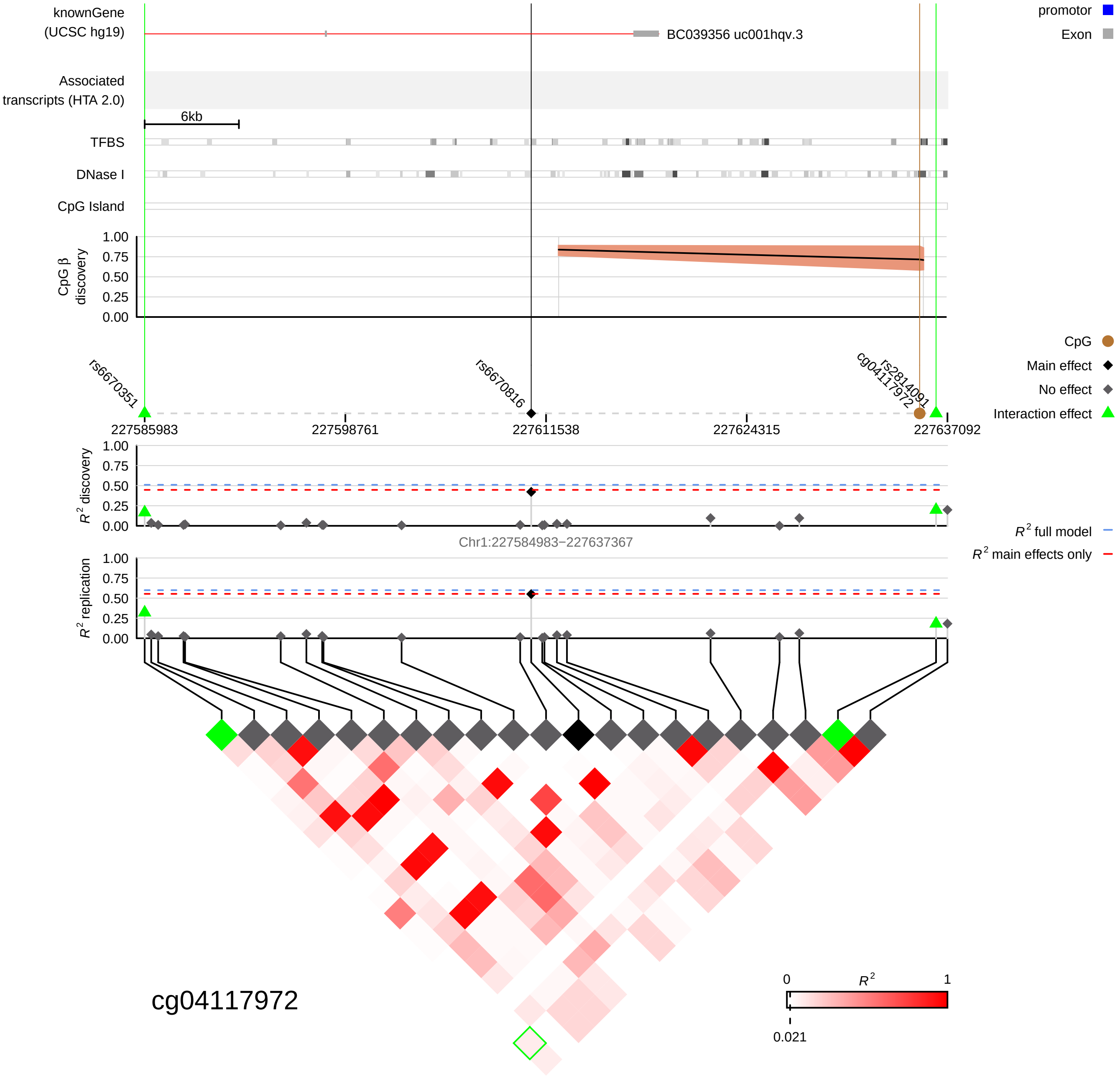

knownGene (UCSC hg19)
BC039356 uc001hqv.3
Associated transcripts (HTA 2.0)
6kb
TFBS
DNase I
CpG Island
CpG β discovery
rs6670351
rs6670816
cg04117972
rs2814091
227585983
227598761
227611538
227624315
227637092
R^2 discovery
Chr1:227584983−227637367
R^2 replication
promotor
Exon
CpG
Main effect
No effect
Interaction effect
R^2 full model
R^2 main effects only
R^2
0.021
cg04117972

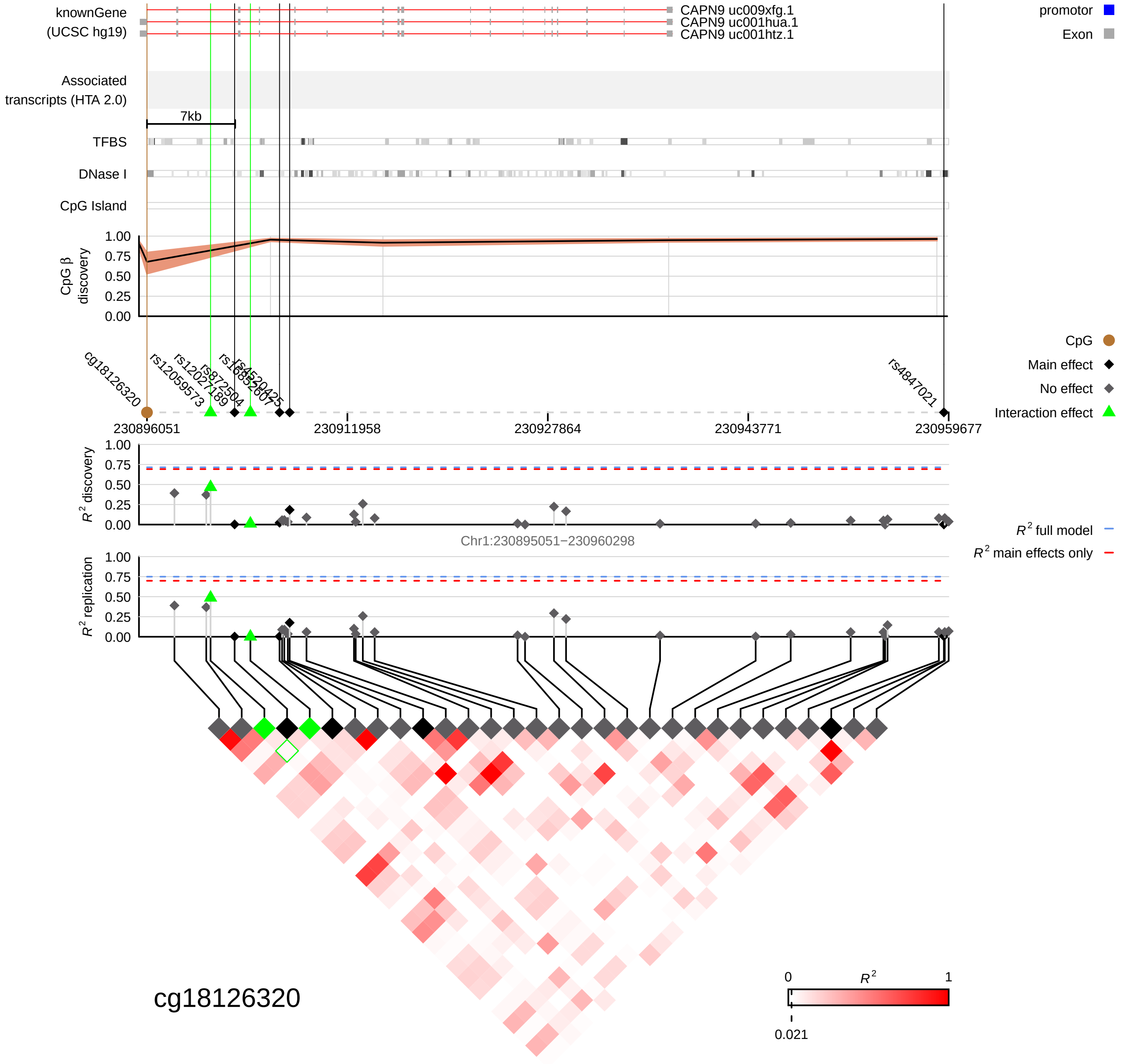

knownGene
(UCSC hg19)
CAPN9 uc009xfg.1
CAPN9 uc001hua.1
CAPN9 uc001htz.1
promotor
Exon
Associated
transcripts (HTA 2.0)
7kb
TFBS
DNase I
CpG Island
CpG β
discovery
1.00
0.75
0.50
0.25
0.00
cg18126320
rs12059573
rs4847021
CpG
Main effect
No effect
Interaction effect
230896051
230911958
230927864
230943771
230959677
R^2 discovery
Chr1:230895051−230960298
R^2 full model
R^2 main effects only
R^2 replication
cg18126320
0
R^2
1
0.021

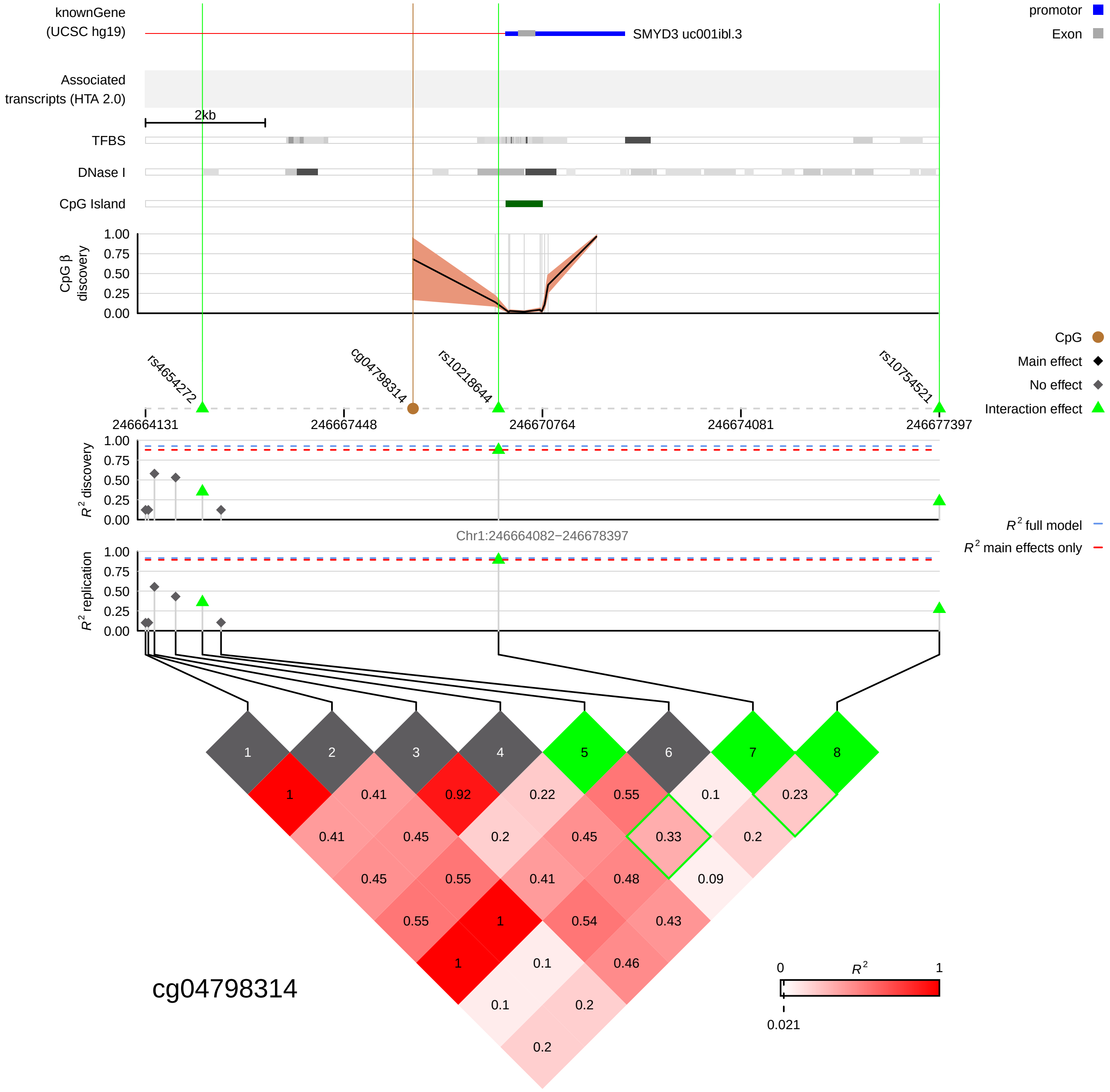

knownGene
(UCSC hg19)
SMYD3 uc001ibl.3
promotor
Exon
Associated
transcripts (HTA 2.0)
2kb
TFBS
DNase I
CpG Island
CpG β
discovery
1.00
0.75
0.50
0.25
0.00
rs4654272
cg04798314
rs10218644
rs10754521
CpG
Main effect
No effect
Interaction effect
246664131
246667448
246670764
246674081
246677397
R² discovery
Chr1:246664082−246678397
R² full model
R² main effects only
R² replication
1
2
3
4
5
6
7
8
1
0.41
0.92
0.22
0.55
0.1
0.23
0.41
0.45
0.2
0.45
0.33
0.2
0.45
0.55
0.41
0.48
0.09
0.55
1
0.54
0.43
1
0.1
0.46
0.1
0.2
0.2
cg04798314
0
R²
1
0.021

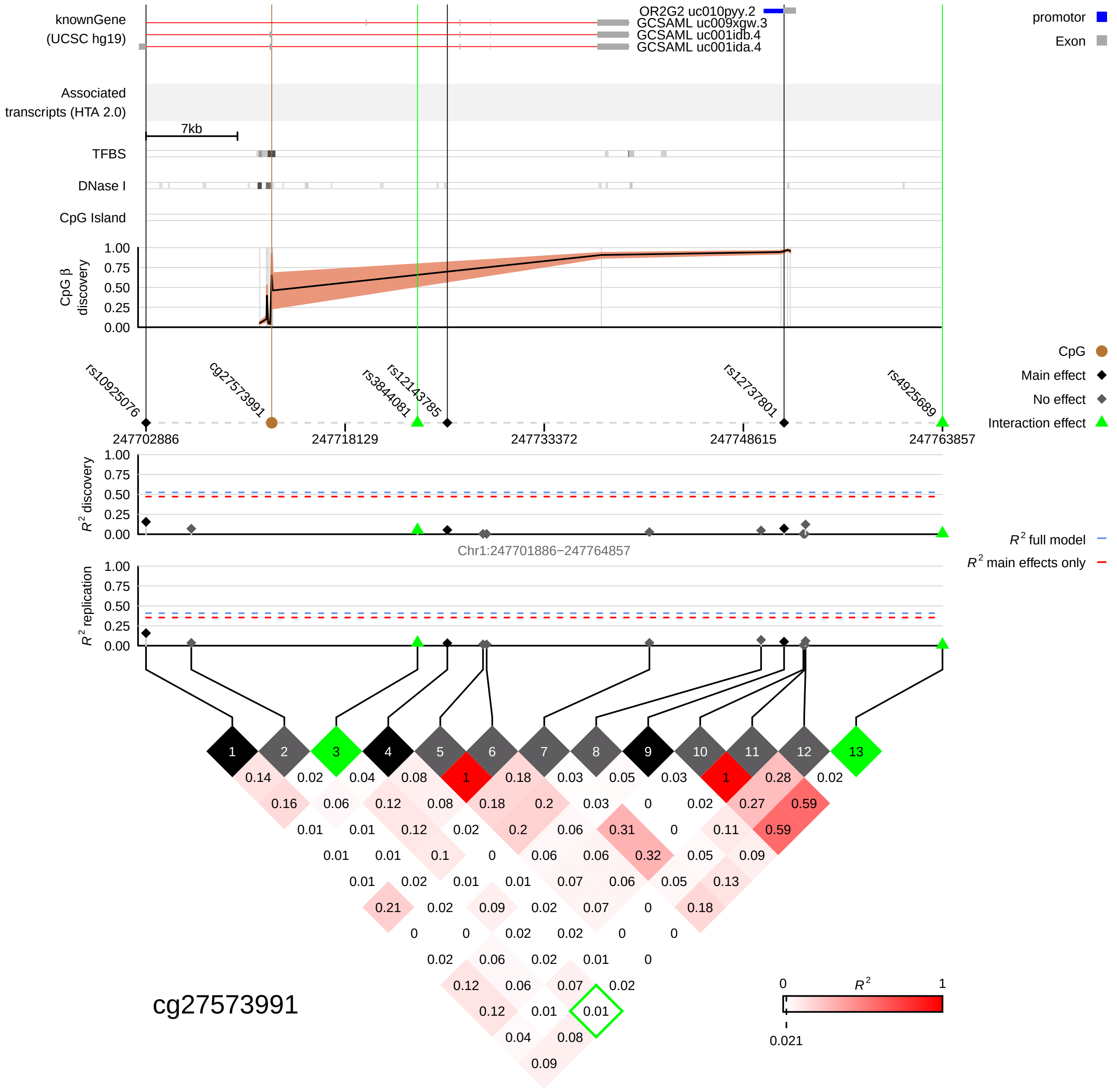

knownGene (UCSC hg19)
OR2G2 uc010pyy.2
GCSAML uc009xgw.3
GCSAML uc001idb.4
GCSAML uc001ida.4
promotor
Exon
Associated transcripts (HTA 2.0)
7kb
TFBS
DNase I
CpG Island
CpG β discovery
rs10925076
cg27573991
rs3844081
rs12143785
rs12737801
rs4925689
CpG
Main effect
No effect
Interaction effect
247702886
247718129
247733372
247748615
247763857
R^2 discovery
Chr1:247701886−247764857
R^2 full model
R^2 main effects only
R^2 replication
cg27573991
0.021

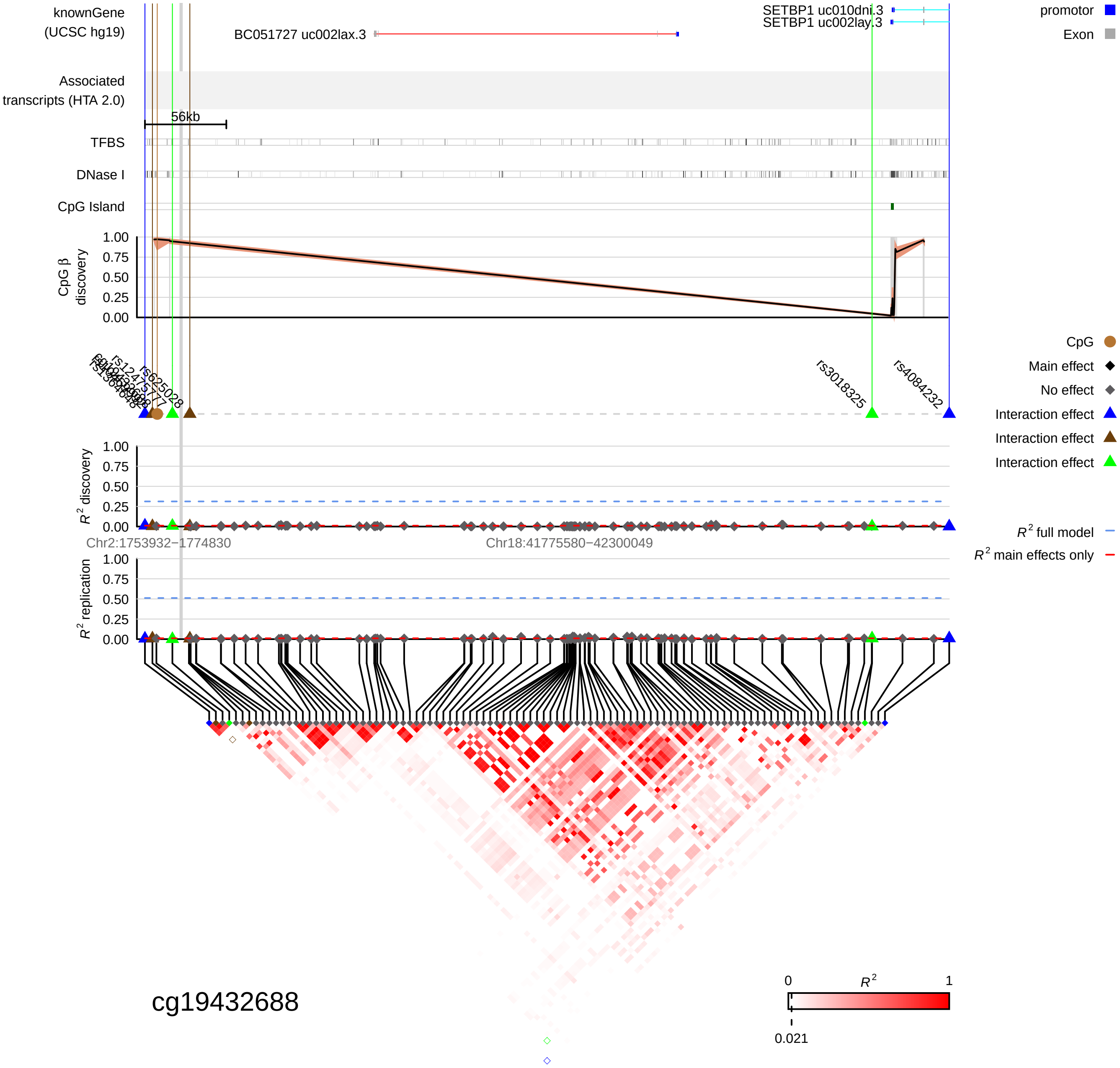
knownGene (UCSC hg19)
BC051727 uc002lax.3
SETBP1 uc010dni.3
SETBP1 uc002lay.3
promotor
Exon
Associated transcripts (HTA 2.0)
56kb
TFBS
DNase I
CpG Island
CpG β discovery
1.00
0.75
0.50
0.25
0.00
rs625028
rs3018325
rs4084232
CpG
Main effect
No effect
Interaction effect
Interaction effect
Interaction effect
R² discovery
R² full model
R² main effects only
Chr2:1753932−1774830
Chr18:41775580−42300049
R² replication
cg19432688
0
R²
1
0.021

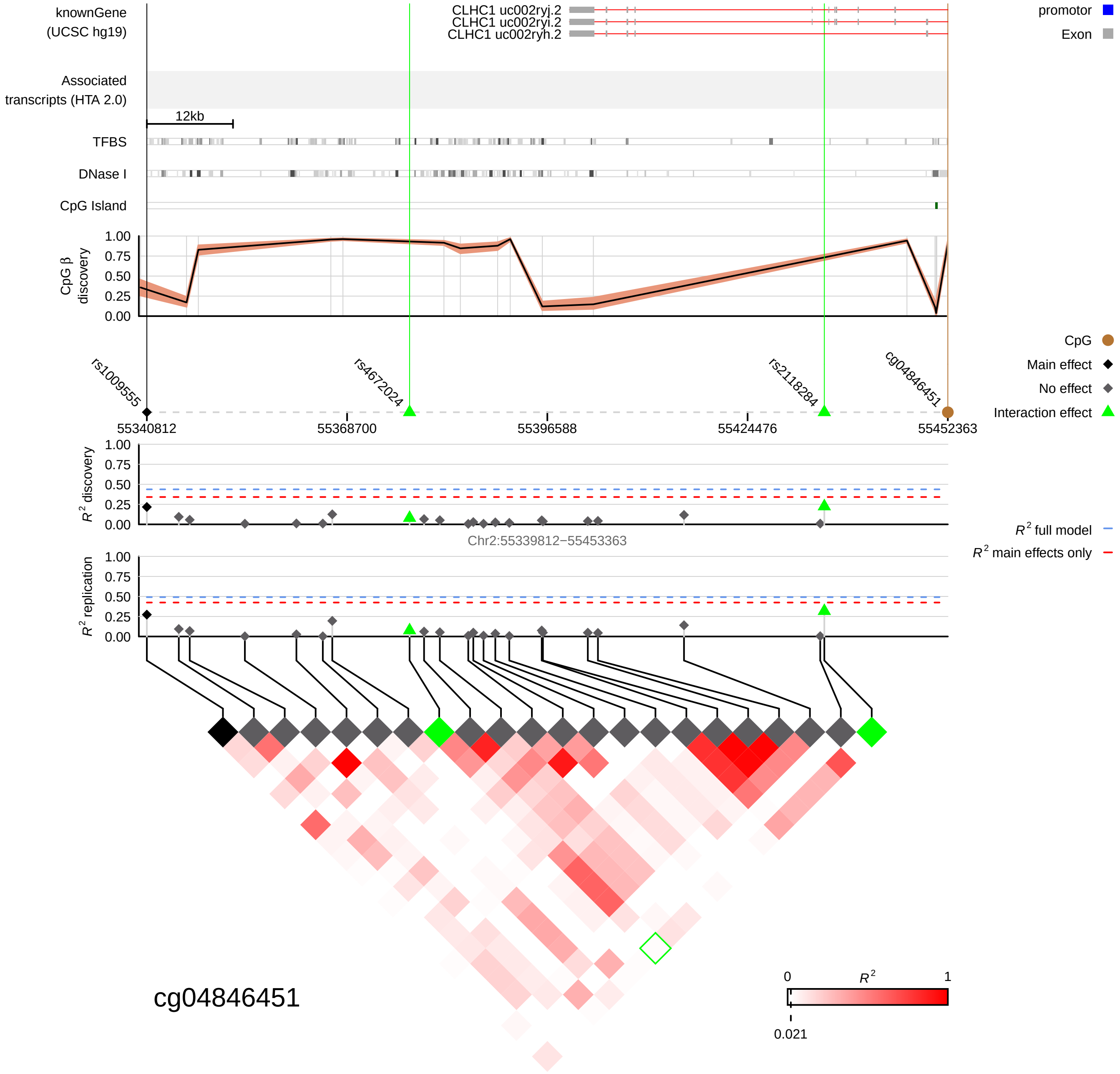
knownGene (UCSC hg19)
CLHC1 uc002ryj.2
CLHC1 uc002ryi.2
CLHC1 uc002ryh.2
promotor
Exon
Associated transcripts (HTA 2.0)
12kb
TFBS
DNase I
CpG Island
CpG β discovery
1.00
0.75
0.50
0.25
0.00
CpG
Main effect
No effect
Interaction effect
rs1009555
rs4672024
rs2118284
cg04846451
55340812
55368700
55396588
55424476
55452363
R2 discovery
Chr2:55339812−55453363
R2 full model
R2 main effects only
R2 replication
0
R2
1
0.021
cg04846451

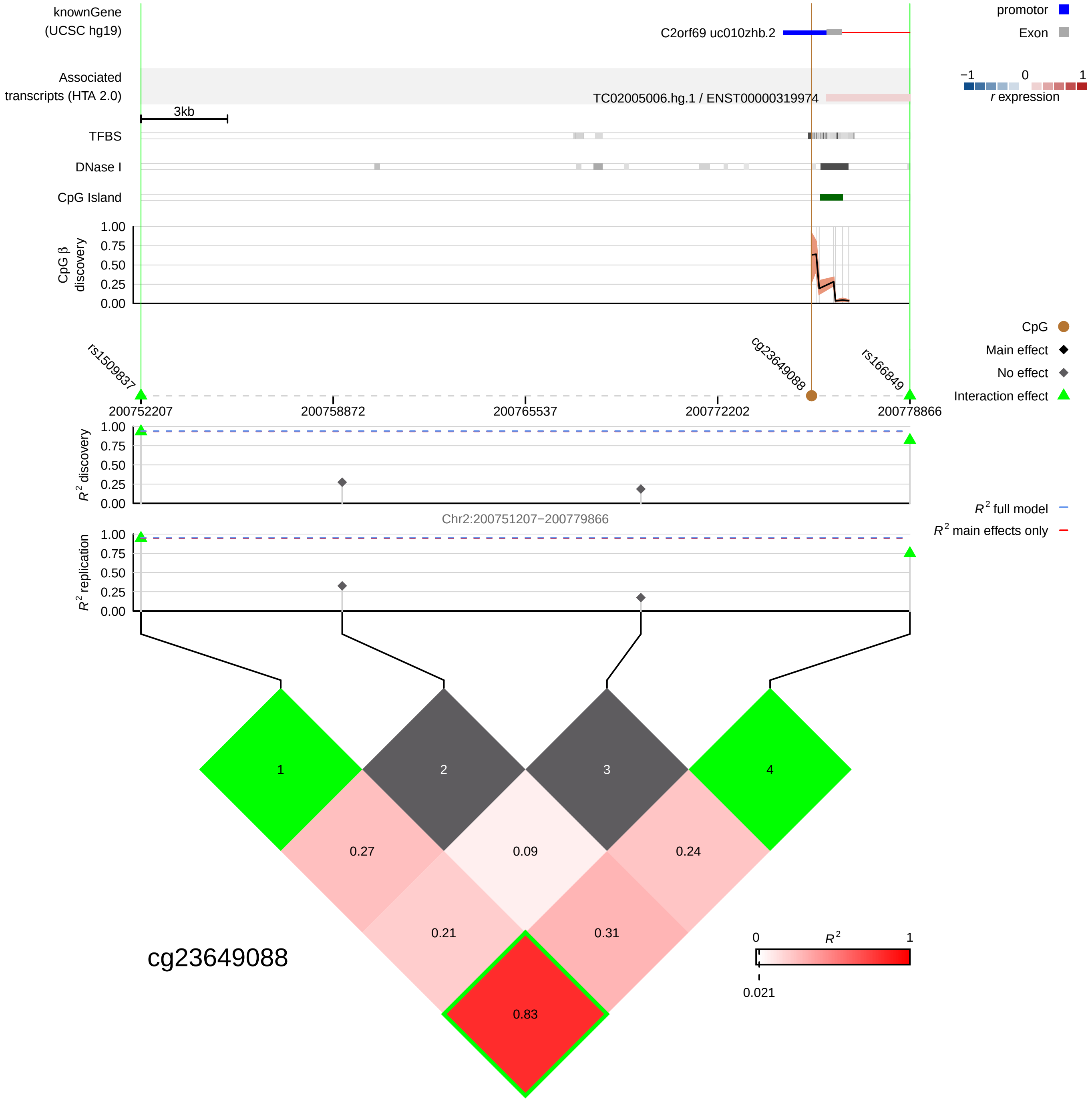
knownGene (UCSC hg19)
C2orf69 uc010zhb.2
promotor
Exon
Associated transcripts (HTA 2.0)
TC02005006.hg.1 / ENST00000319974
−1
0
1
r expression
3kb
TFBS
DNase I
CpG Island
CpG β discovery
1.00
0.75
0.50
0.25
0.00
rs1509837
cg23649088
rs166849
CpG
Main effect
No effect
Interaction effect
200752207
200758872
200765537
200772202
200778866
R2 discovery
Chr2:200751207−200779866
R2 full model
R2 main effects only
R2 replication
1
2
3
4
0.27
0.09
0.24
0.21
0.31
0.83
cg23649088
0
R2
1
0.021

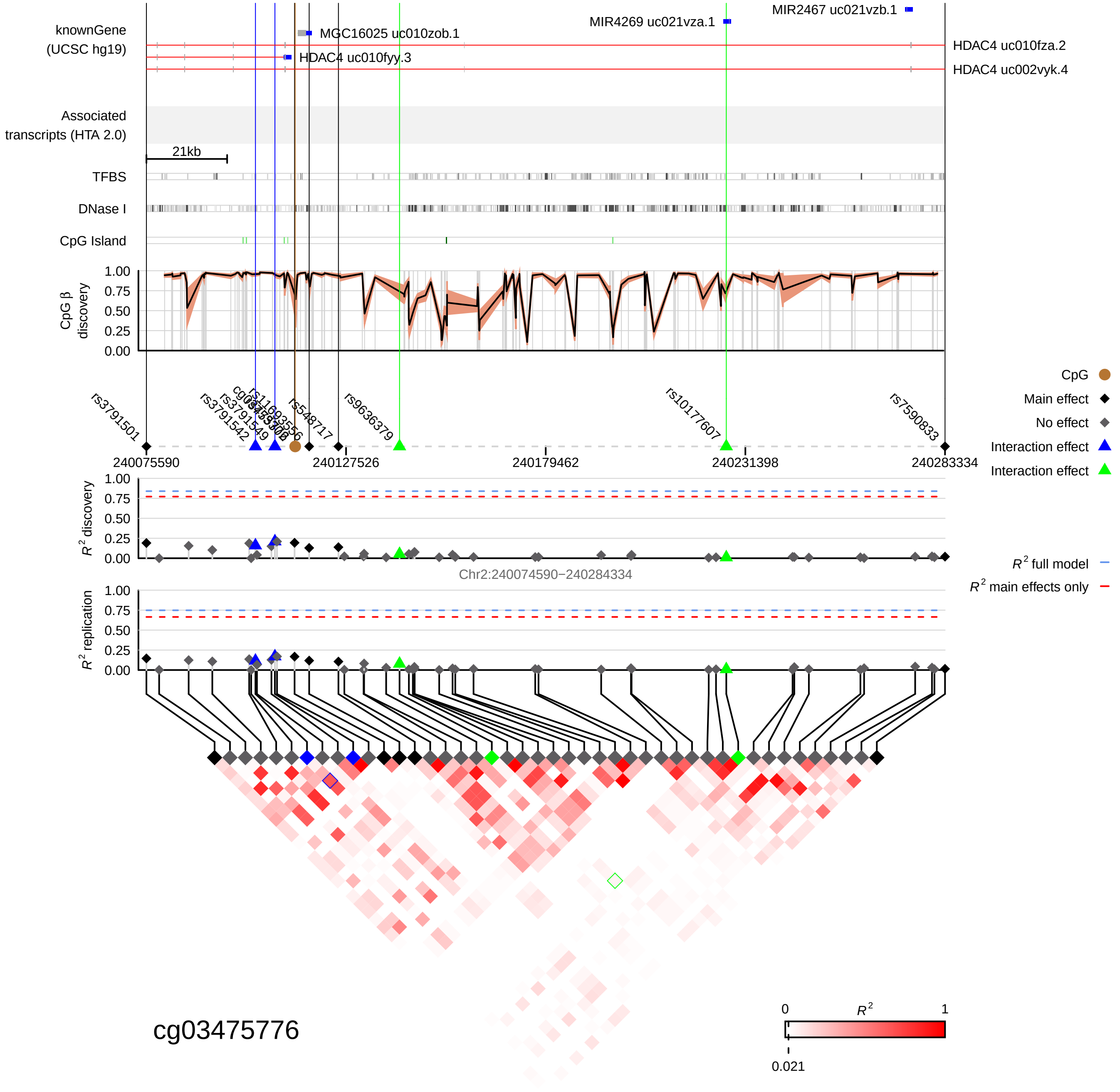
knownGene
(UCSC hg19)
MGC16025 uc010zob.1
HDAC4 uc010fyy.3
MIR4269 uc021vza.1
MIR2467 uc021vzb.1
HDAC4 uc010fza.2
HDAC4 uc002vyk.4
Associated
transcripts (HTA 2.0)
21kb
TFBS
DNase I
CpG Island
CpG β
discovery
1.00
0.75
0.50
0.25
0.00
rs3791501
rs548717
rs9636379
rs10177607
rs7590833
240075590
240127526
240179462
240231398
240283334
CpG
Main effect
No effect
Interaction effect
Interaction effect
R^2 discovery
Chr2:240074590−240284334
R^2 full model
R^2 main effects only
R^2 replication
0
R^2
1
0.021
cg03475776

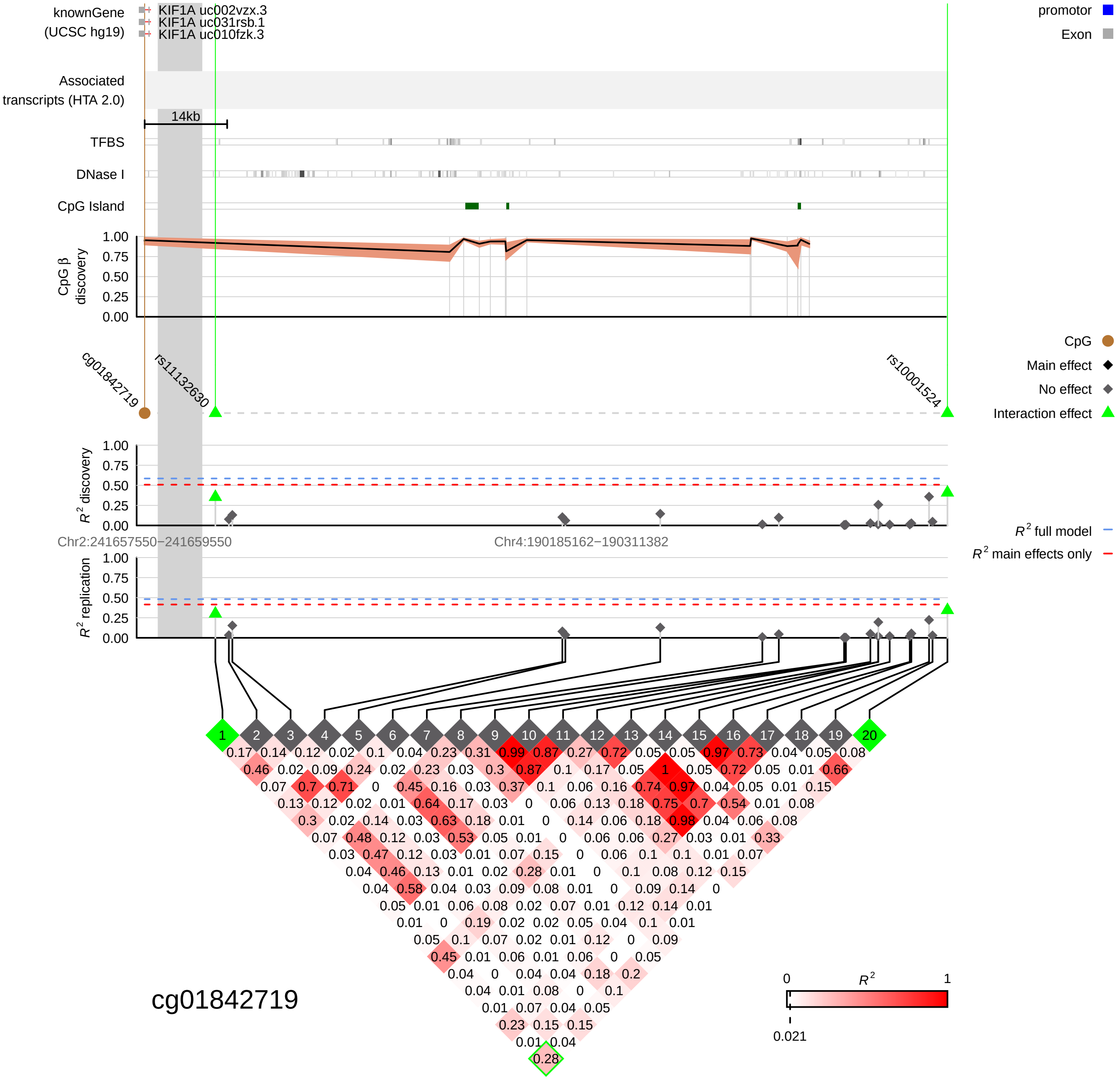
knownGene
(UCSC hg19)
KIF1A uc002vzx.3
KIF1A uc031rsb.1
KIF1A uc010fzk.3
Associated
transcripts (HTA 2.0)
14kb
TFBS
DNase I
CpG Island
CpG β discovery
cg01842719
rs11132630
rs10001524
R^2 discovery
Chr2:241657550−241659550
Chr4:190185162−190311382
R^2 replication
promotor
Exon
CpG
Main effect
No effect
Interaction effect
R^2 full model
R^2 main effects only
R^2
0.021
cg01842719

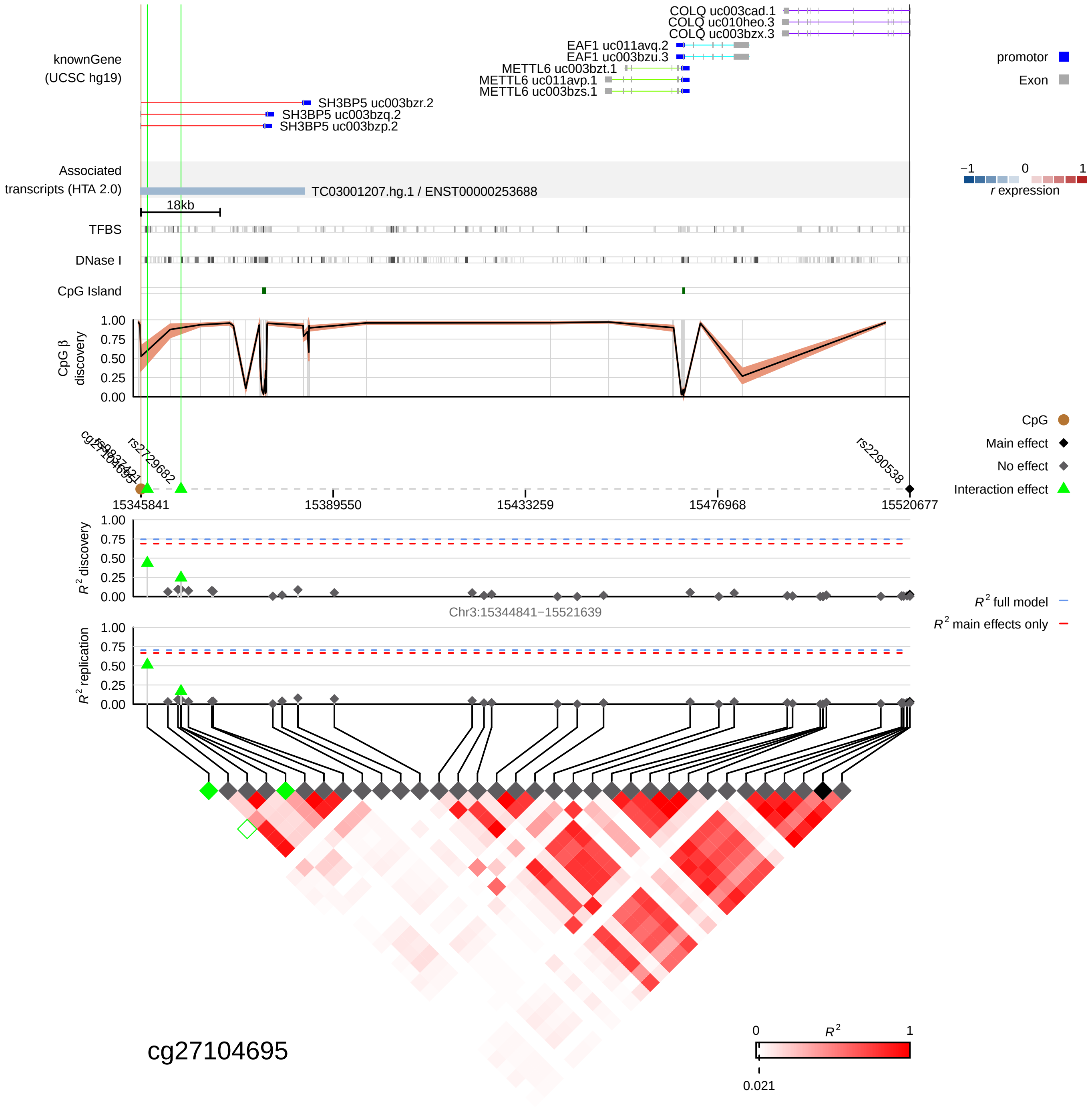

knownGene
(UCSC hg19)
COLQ uc003cad.1
COLQ uc010heo.3
COLQ uc003bzx.3
EAF1 uc011avq.2
EAF1 uc003bzu.3
METTL6 uc003bzt.1
METTL6 uc011avp.1
METTL6 uc003bzs.1
SH3BP5 uc003bzr.2
SH3BP5 uc003bzq.2
SH3BP5 uc003bzp.2
promotor
Exon
Associated
transcripts (HTA 2.0)
TC03001207.hg.1 / ENST00000253688
−1
0
1
r expression
18kb
TFBS
DNase I
CpG Island
CpG β
discovery
1.00
0.75
0.50
0.25
0.00
rs2729682
rs2290538
CpG
Main effect
No effect
Interaction effect
15345841
15389550
15433259
15476968
15520677
R^2 discovery
Chr3:15344841−15521639
R^2 full model
R^2 main effects only
R^2 replication
0
R^2
1
0.021
cg27104695

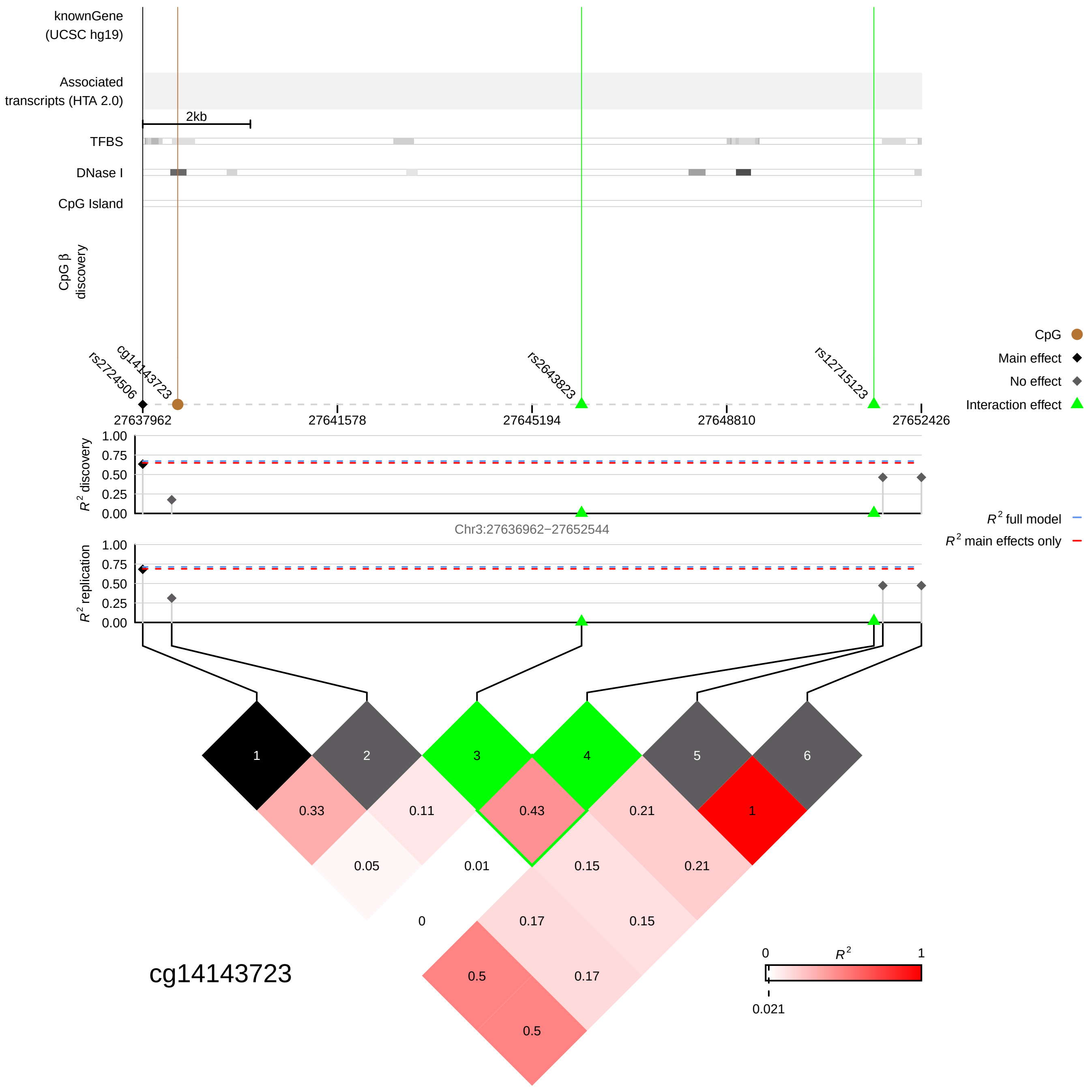
knownGene (UCSC hg19)
Associated transcripts (HTA 2.0)
2kb
TFBS
DNase I
CpG Island
CpG β discovery
rs2724506
cg14143723
rs2643823
rs12715123
27637962
27641578
27645194
27648810
27652426
CpG
Main effect
No effect
Interaction effect
R^2 discovery
Chr3:27636962−27652544
R^2 replication
R^2 full model
R^2 main effects only
1
2
3
4
5
6
0.33
0.11
0.43
0.21
1
0.05
0.01
0.15
0.21
0
0.17
0.15
0.5
0.17
0.5
cg14143723
0
R^2
1
0.021

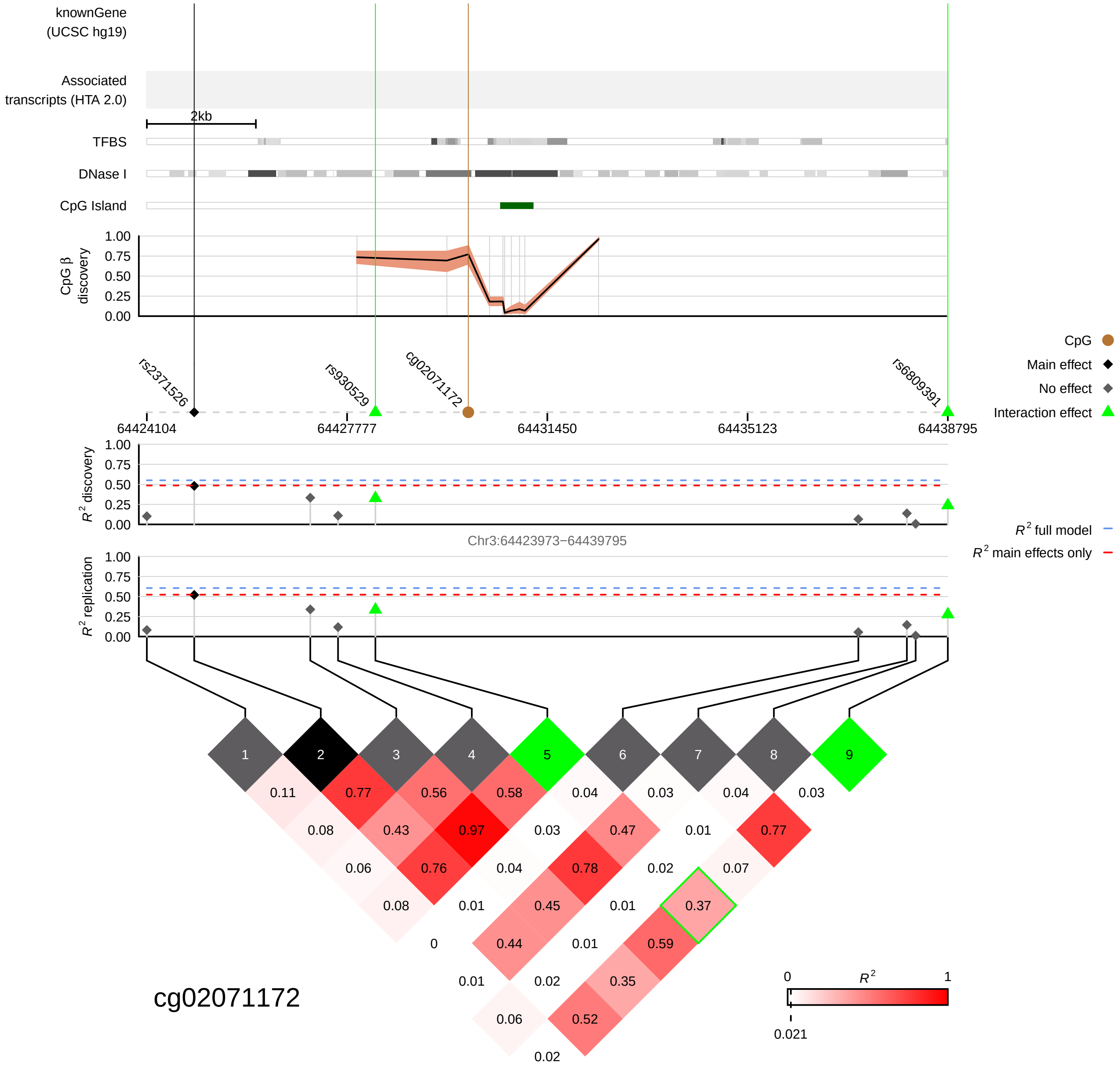
knownGene (UCSC hg19)
Associated transcripts (HTA 2.0)
2kb
TFBS
DNase I
CpG Island
CpG β discovery
rs2371526
rs930529
cg02071172
rs6809391
64424104
64427777
64431450
64435123
64438795
CpG
Main effect
No effect
Interaction effect
R² discovery
Chr3:64423973−64439795
R² full model
R² main effects only
R² replication
R²
0.021
cg02071172

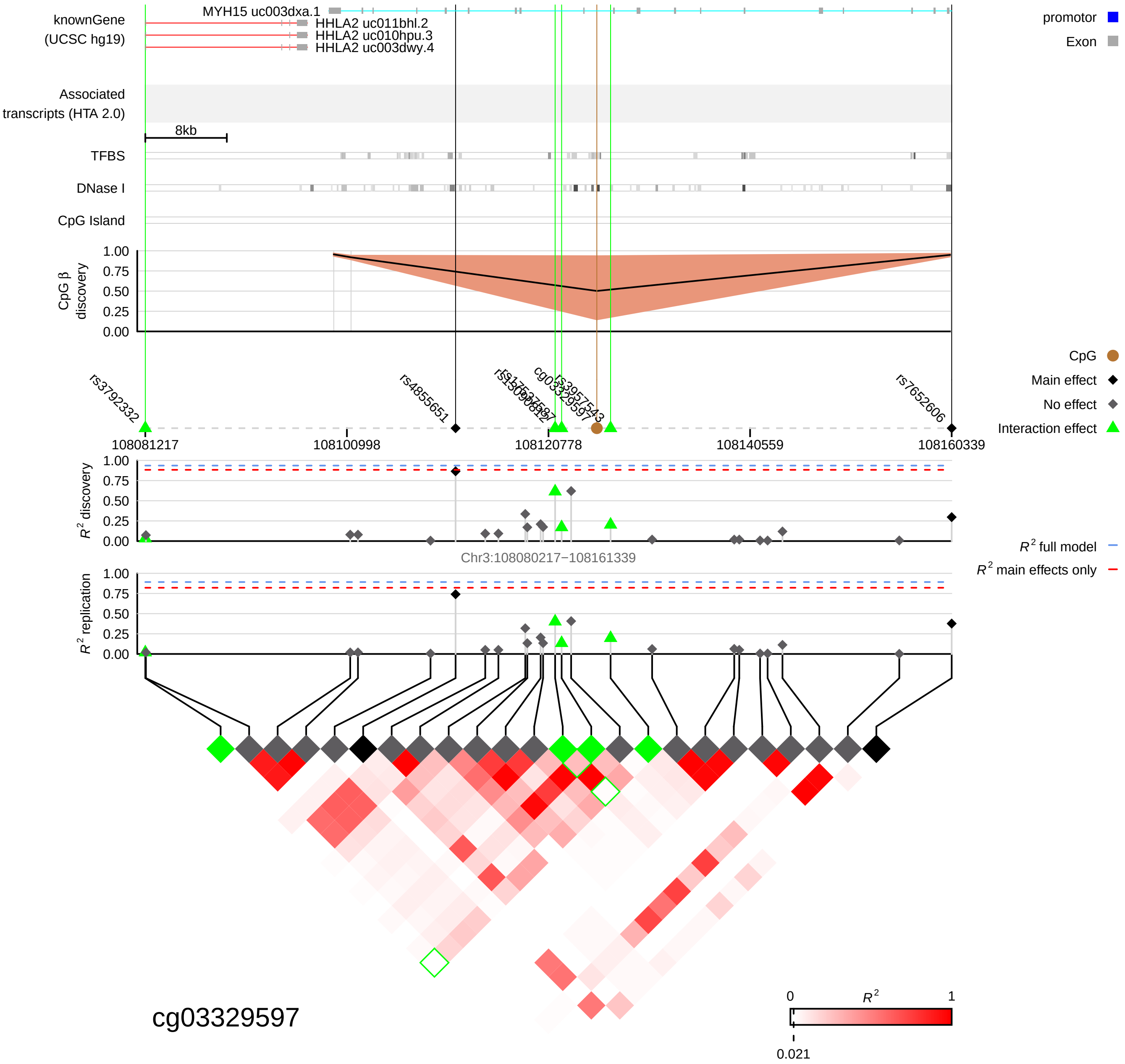
knownGene
(UCSC hg19)
MYH15 uc003dxa.1
HHLA2 uc011bhl.2
HHLA2 uc010hpu.3
HHLA2 uc003dwy.4
promotor
Exon
Associated
transcripts (HTA 2.0)
8kb
TFBS
DNase I
CpG Island
CpG β
discovery
1.00
0.75
0.50
0.25
0.00
rs3792332
rs4855651
rs7652606
CpG
Main effect
No effect
Interaction effect
108081217
108100998
108120778
108140559
108160339
R^2 discovery
R^2 full model
R^2 main effects only
Chr3:108080217−108161339
R^2 replication
cg03329597
0
R^2
1
0.021

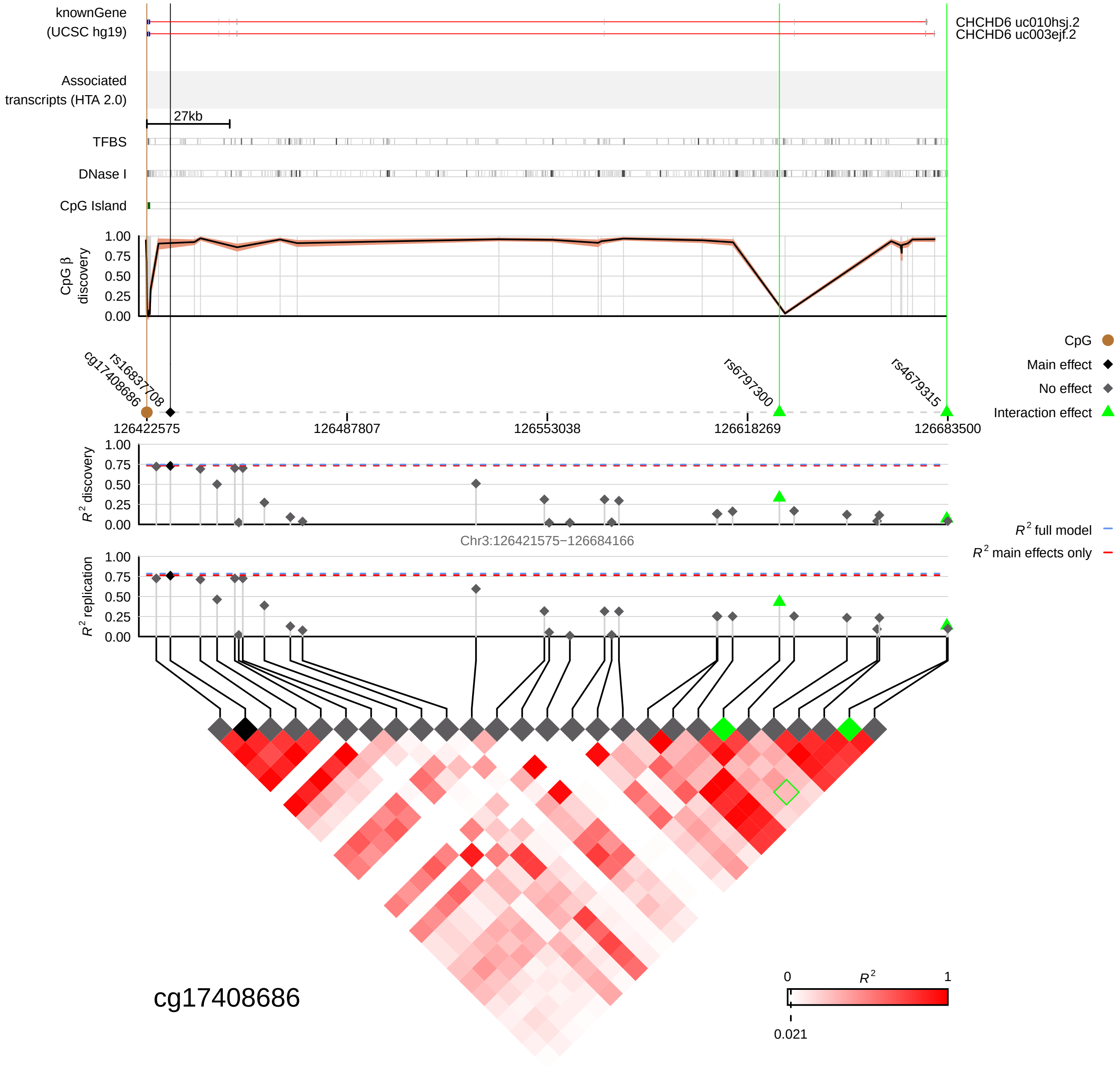

knownGene (UCSC hg19)
CHCHD6 uc010hsj.2
CHCHD6 uc003ejf.2
Associated transcripts (HTA 2.0)
27kb
TFBS
DNase I
CpG Island
CpG β discovery
1.00
0.75
0.50
0.25
0.00
cg17408686
rs16837708
rs6797300
rs4679315
CpG
Main effect
No effect
Interaction effect
126422575
126487807
126553038
126618269
126683500
R2 discovery
Chr3:126421575−126684166
R2 full model
R2 main effects only
R2 replication
cg17408686
0
R2
1
0.021

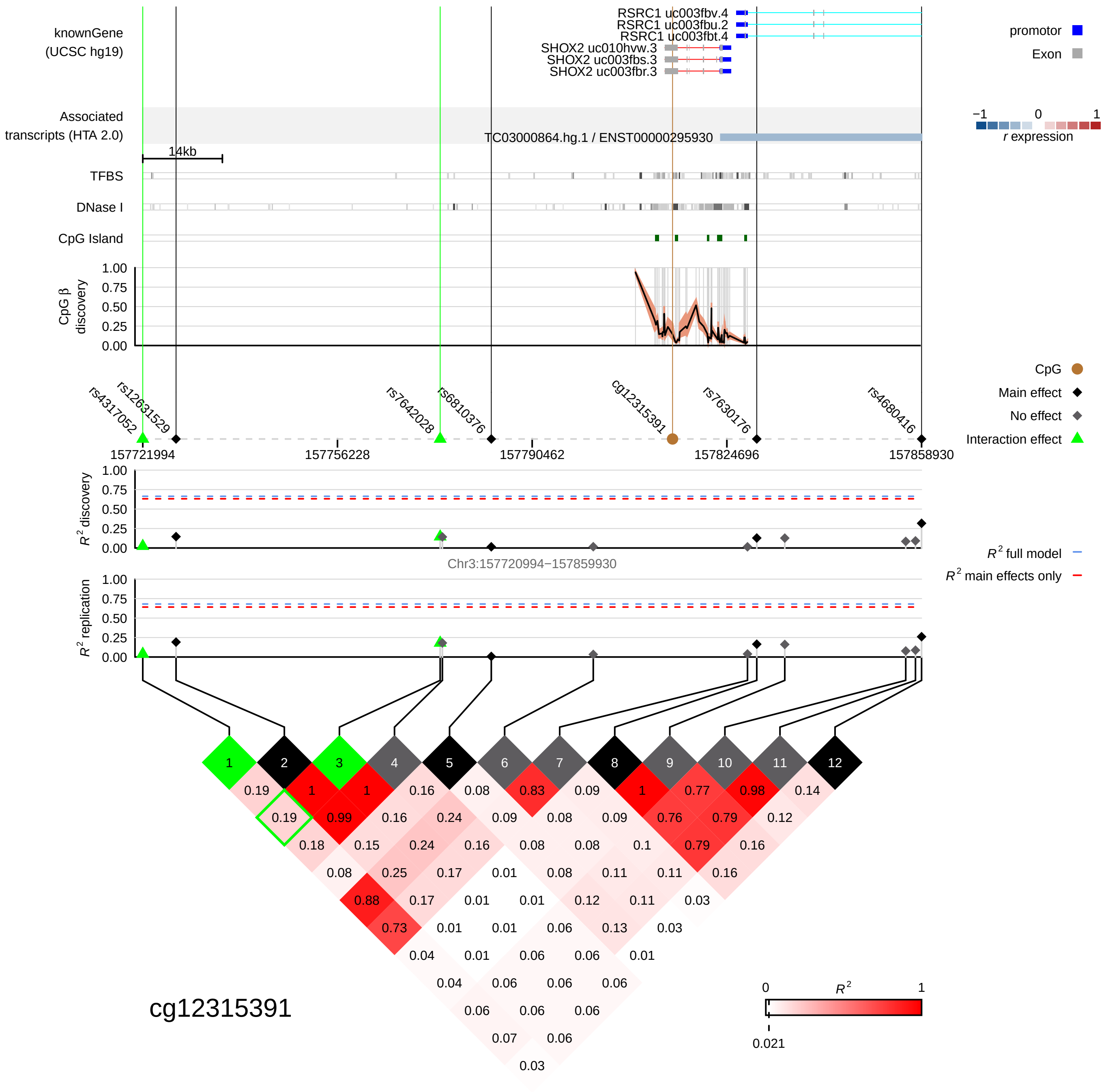

knownGene (UCSC hg19)
RSRC1 uc003fbv.4
RSRC1 uc003fbu.2
RSRC1 uc003fbt.4
SHOX2 uc010hvw.3
SHOX2 uc003fbs.3
SHOX2 uc003fbr.3
promotor
Exon
Associated transcripts (HTA 2.0)
TC03000864.hg.1 / ENST00000295930
r expression
14kb
TFBS
DNase I
CpG Island
CpG β discovery
rs4317052
rs12631529
rs7642028
rs6810376
cg12315391
rs7630176
rs4680416
CpG
Main effect
No effect
Interaction effect
157721994
157756228
157790462
157824696
157858930
R2 discovery
Chr3:157720994−157859930
R2 full model
R2 main effects only
R2 replication
cg12315391
0.021

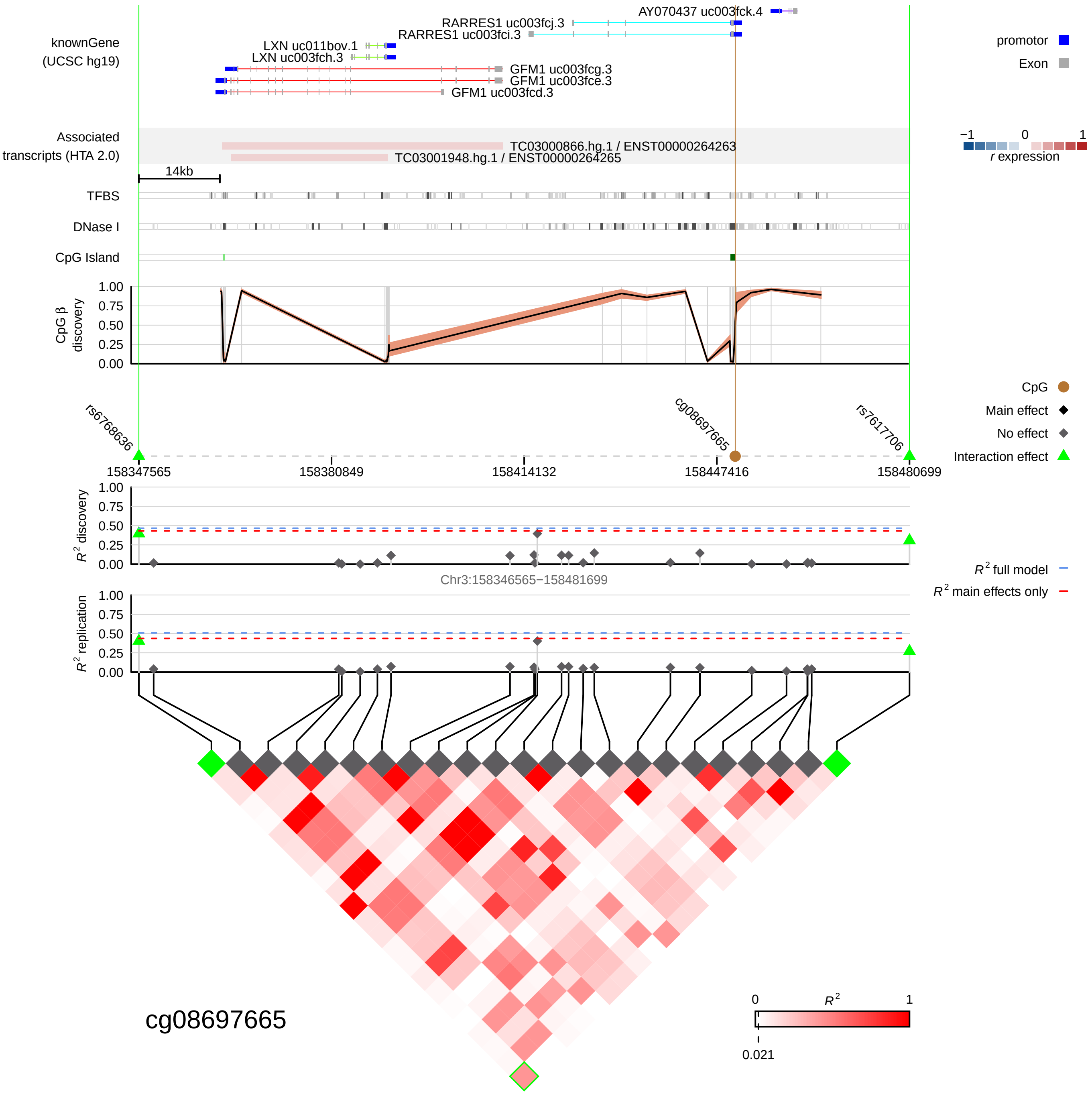
knownGene
(UCSC hg19)
AY070437 uc003fck.4
RARRES1 uc003fcj.3
RARRES1 uc003fci.3
LXN uc011bov.1
LXN uc003fch.3
GFM1 uc003fcg.3
GFM1 uc003fce.3
GFM1 uc003fcd.3
promotor
Exon
Associated
transcripts (HTA 2.0)
TC03000866.hg.1 / ENST00000264263
TC03001948.hg.1 / ENST00000264265
−1
0
1
r expression
14kb
TFBS
DNase I
CpG Island
CpG β
discovery
1.00
0.75
0.50
0.25
0.00
rs6768636
cg08697665
rs7617706
158347565
158380849
158414132
158447416
158480699
CpG
Main effect
No effect
Interaction effect
R2 discovery
R2 full model
R2 main effects only
Chr3:158346565−158481699
R2 replication
0
R2
1
0.021
cg08697665

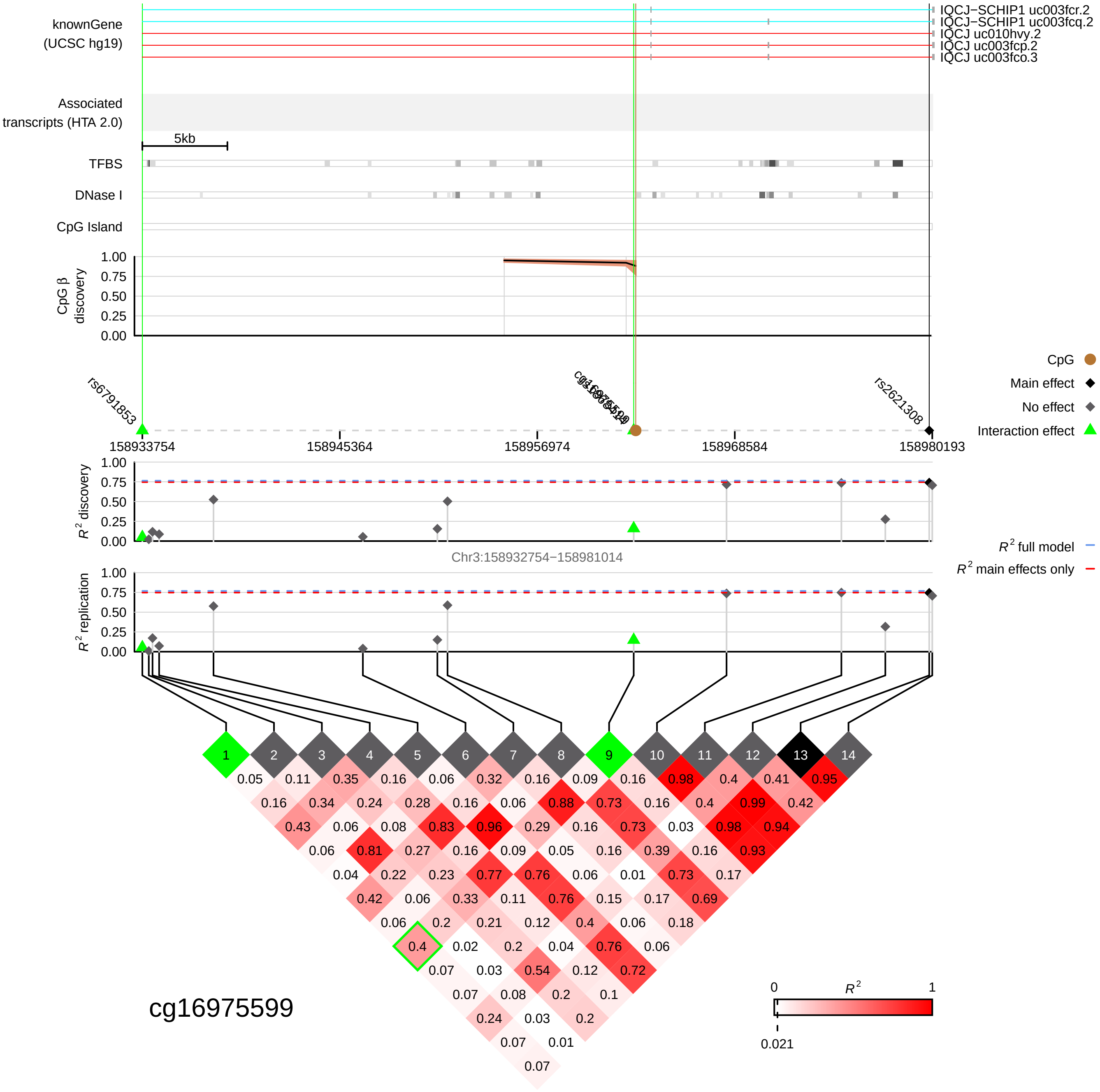
knownGene
(UCSC hg19)
IQCJ−SCHIP1 uc003fcr.2
IQCJ−SCHIP1 uc003fcq.2
IQCJ uc010hvy.2
IQCJ uc003fcp.2
IQCJ uc003fco.3
Associated
transcripts (HTA 2.0)
5kb
TFBS
DNase I
CpG Island
CpG β
discovery
1.00
0.75
0.50
0.25
0.00
rs6791853
rs2621308
CpG
Main effect
No effect
Interaction effect
158933754
158945364
158956974
158968584
158980193
R^2 discovery
Chr3:158932754−158981014
R^2 full model
R^2 main effects only
R^2 replication
1
2
3
4
5
6
7
8
9
10
11
12
13
14
0
R^2
1
0.021
cg16975599

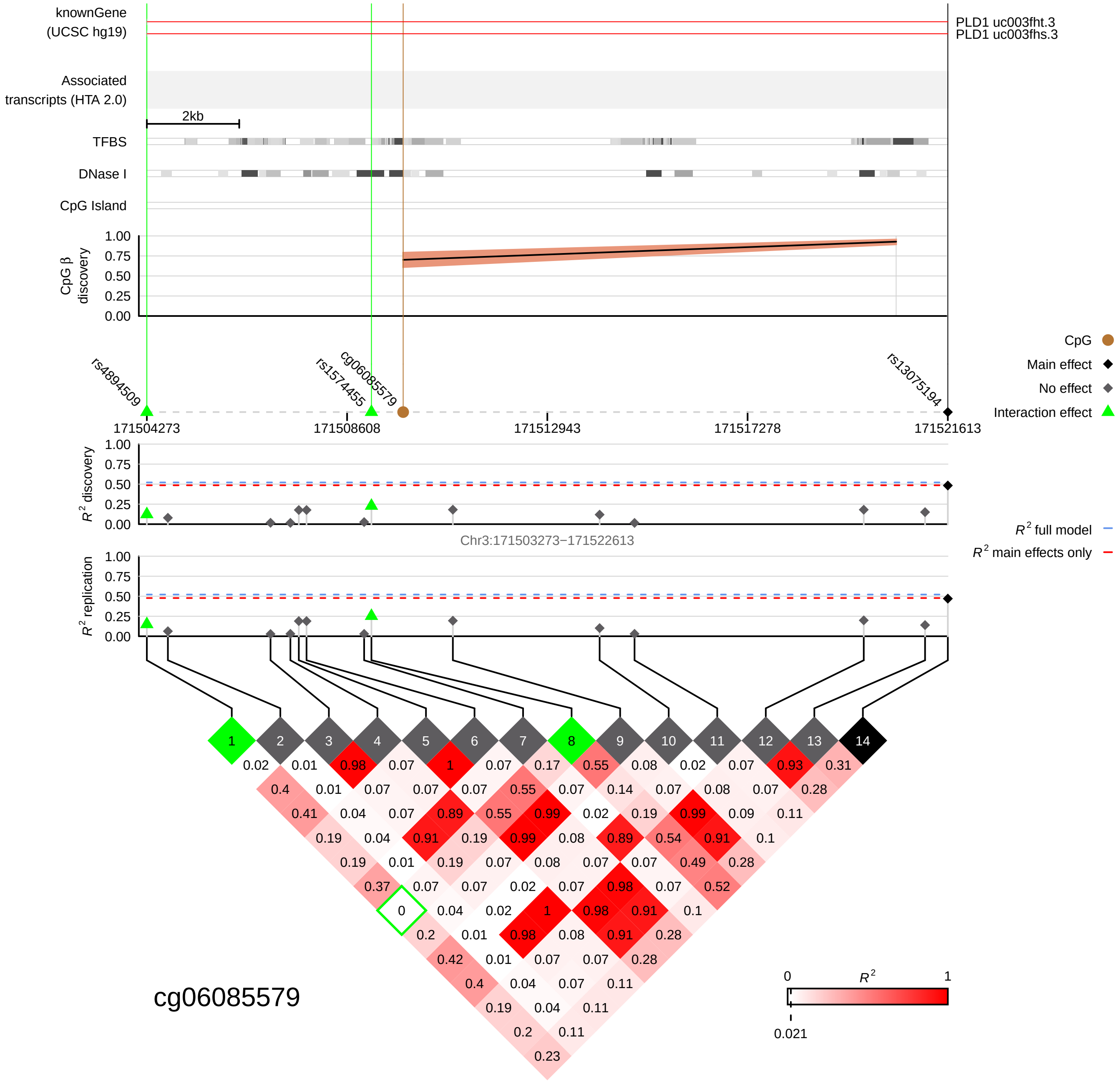

knownGene (UCSC hg19)
PLD1 uc003fht.3
PLD1 uc003fhs.3
Associated transcripts (HTA 2.0)
2kb
TFBS
DNase I
CpG Island
CpG β discovery
rs4894509
rs1574455
cg06085579
rs13075194
171504273
171508608
171512943
171517278
171521613
CpG
Main effect
No effect
Interaction effect
R^2 discovery
Chr3:171503273−171522613
R^2 replication
R^2 full model
R^2 main effects only
R^2
0.021
cg06085579

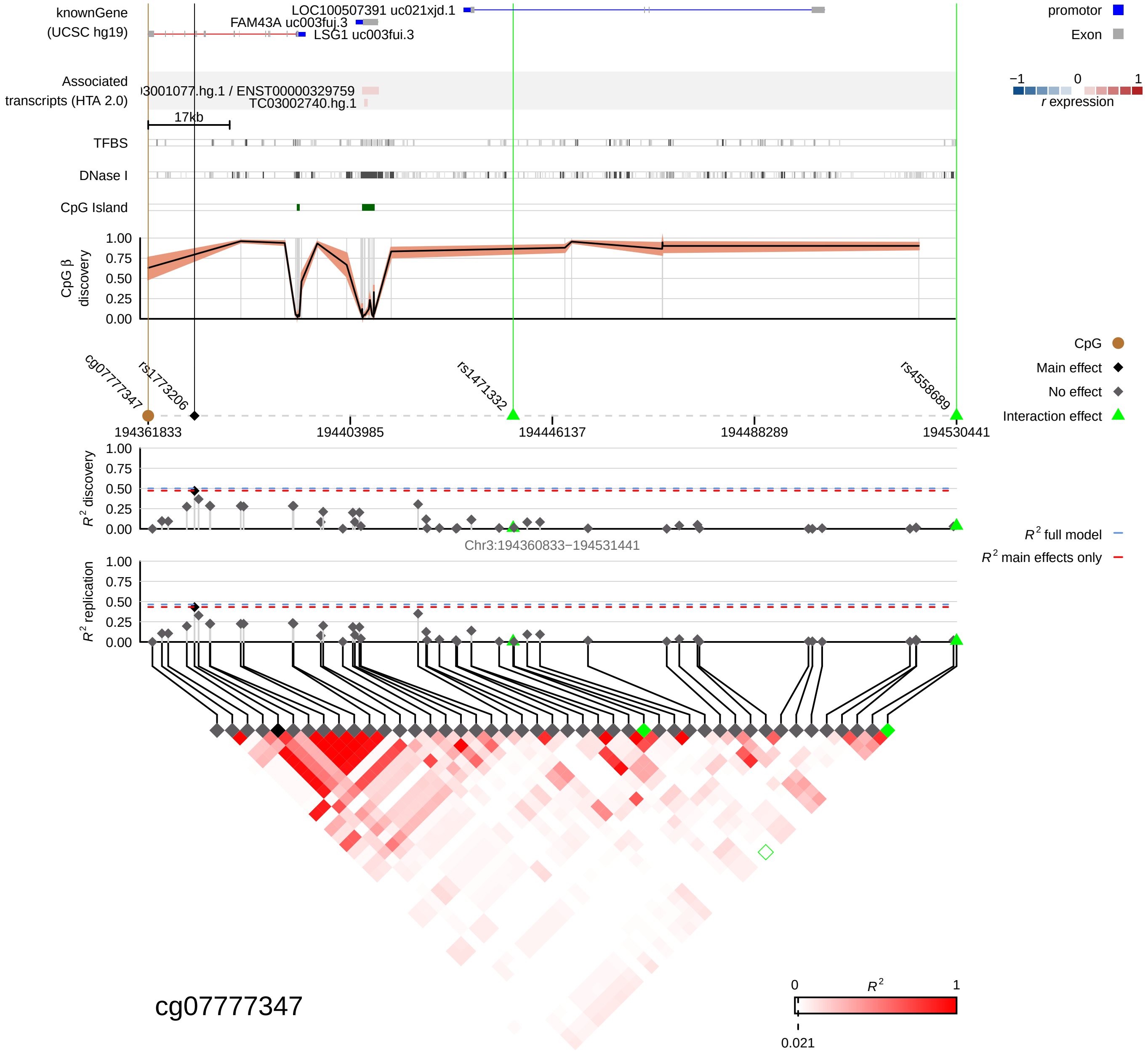
knownGene (UCSC hg19)
LOC100507391 uc021xjd.1
FAM43A uc003fuj.3
LSG1 uc003fui.3
promotor
Exon
Associated transcripts (HTA 2.0)
TC03002740.hg.1
r expression
17kb
TFBS
DNase I
CpG Island
CpG β discovery
cg07777347
rs1773206
rs1471332
rs4558689
194361833
194403985
194446137
194488289
194530441
CpG
Main effect
No effect
Interaction effect
R² discovery
Chr3:194360833−194531441
R² full model
R² main effects only
R² replication
R²
0.021
cg07777347

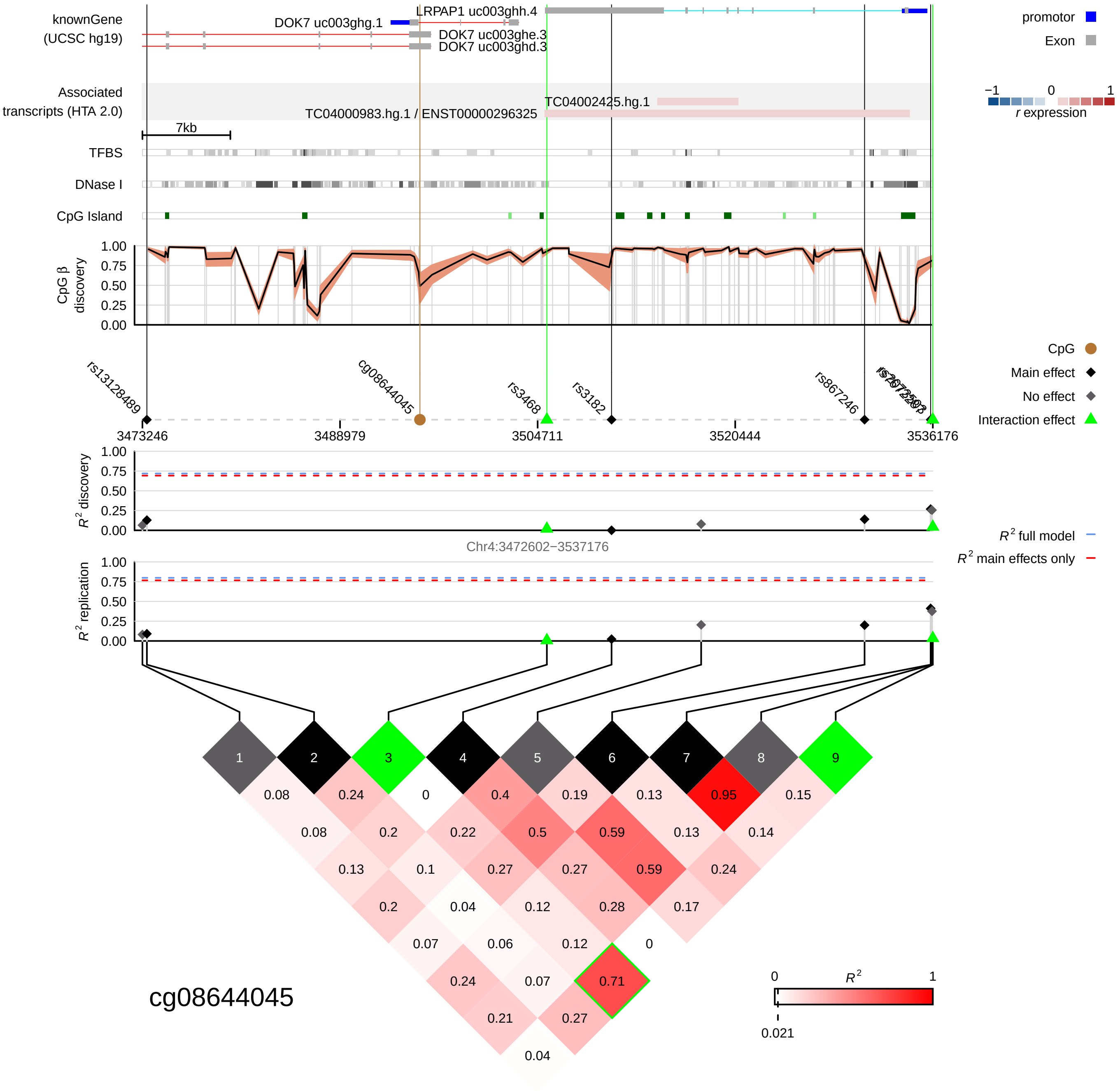
knownGene
(UCSC hg19)
DOK7 uc003ghg.1
LRPAP1 uc003ghh.4
DOK7 uc003ghe.3
DOK7 uc003ghd.3
promotor
Exon
Associated
transcripts (HTA 2.0)
TC04002425.hg.1
TC04000983.hg.1 / ENST00000296325
−1
0
1
r expression
7kb
TFBS
DNase I
CpG Island
CpG β
discovery
1.00
0.75
0.50
0.25
0.00
rs13128489
cg08644045
rs3468
rs3182
rs867246
CpG
Main effect
No effect
Interaction effect
3473246
3488979
3504711
3520444
3536176
R^2 discovery
Chr4:3472602−3537176
R^2 full model
R^2 main effects only
R^2 replication
1
2
3
4
5
6
7
8
9
0.08
0.24
0
0.4
0.19
0.13
0.95
0.15
0.08
0.2
0.22
0.5
0.59
0.13
0.14
0.13
0.1
0.27
0.27
0.59
0.24
0.2
0.04
0.12
0.28
0.17
0.07
0.06
0.12
0
0.24
0.07
0.71
0.21
0.27
0.04
cg08644045
0
R^2
1
0.021

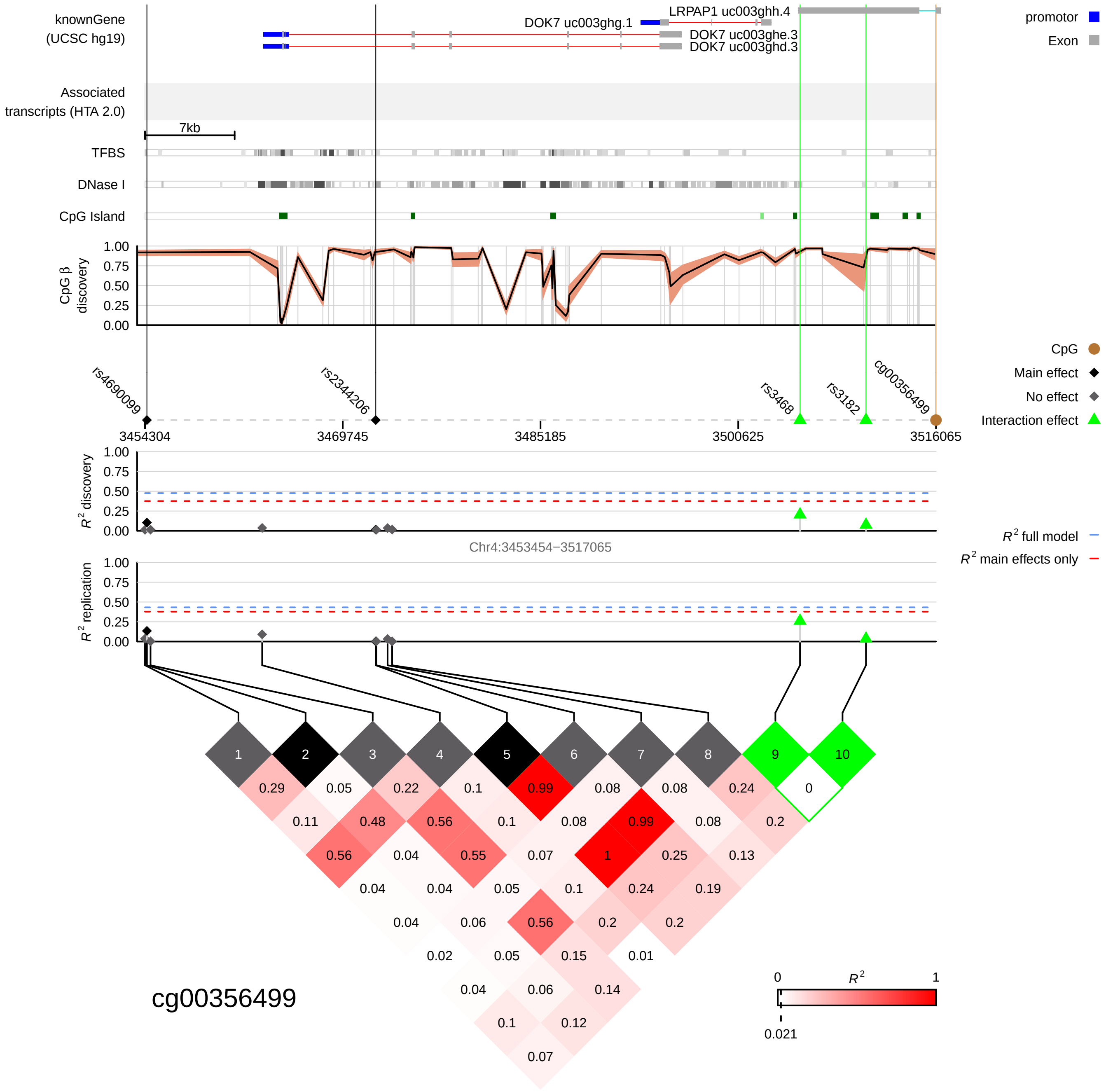

knownGene (UCSC hg19)
LRPAP1 uc003ghh.4
DOK7 uc003ghg.1
DOK7 uc003ghe.3
DOK7 uc003ghd.3
promotor
Exon
Associated transcripts (HTA 2.0)
7kb
TFBS
DNase I
CpG Island
CpG β discovery
1.00
0.75
0.50
0.25
0.00
rs4690099
rs2344206
rs3468
rs3182
cg00356499
CpG
Main effect
No effect
Interaction effect
3454304
3469745
3485185
3500625
3516065
R^2 discovery
Chr4:3453454−3517065
R^2 full model
R^2 main effects only
R^2 replication
1
2
3
4
5
6
7
8
9
10
0.29
0.05
0.22
0.1
0.99
0.08
0.08
0.24
0
0.11
0.48
0.56
0.1
0.08
0.99
0.08
0.2
0.56
0.04
0.55
0.07
1
0.25
0.13
0.04
0.04
0.05
0.1
0.24
0.19
0.04
0.06
0.56
0.2
0.2
0.02
0.05
0.15
0.01
0.04
0.06
0.14
0.1
0.12
0.07
0
R^2
1
0.021

cg00356499

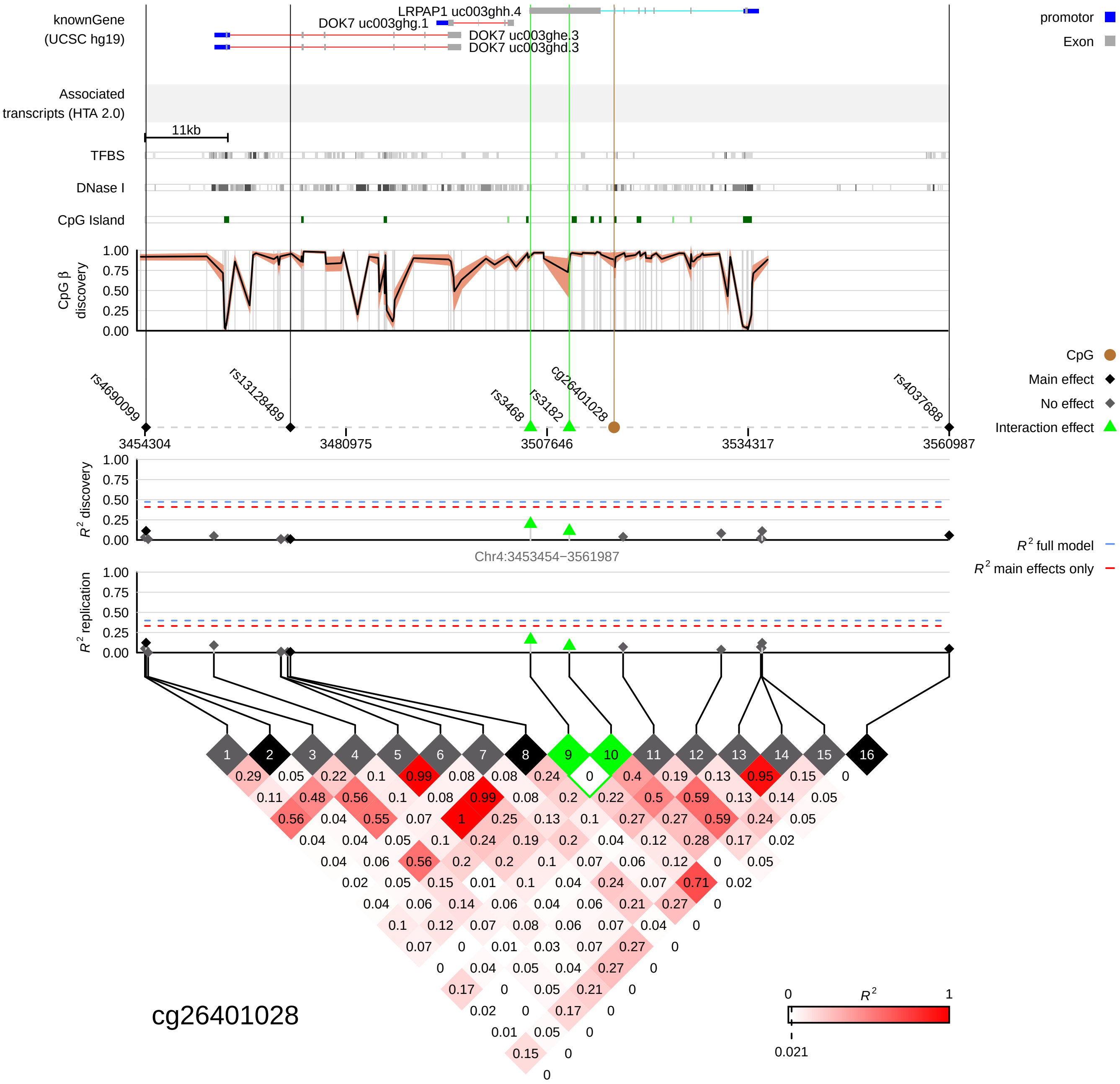
knownGene
(UCSC hg19)
LRPAP1 uc003ghh.4
DOK7 uc003ghg.1
DOK7 uc003ghe.3
DOK7 uc003ghd.3
promotor
Exon
Associated
transcripts (HTA 2.0)
11kb
TFBS
DNase I
CpG Island
CpG β discovery
1.00
0.75
0.50
0.25
0.00
rs4690099
rs13128489
rs3468
rs3182
cg26401028
rs4037688
3454304
3480975
3507646
3534317
3560987
CpG
Main effect
No effect
Interaction effect
R² discovery
Chr4:3453454−3561987
R² full model
R² main effects only
R² replication
1
2
3
4
5
6
7
8
9
10
11
12
13
14
15
16
0
R²
1
0.021
cg26401028

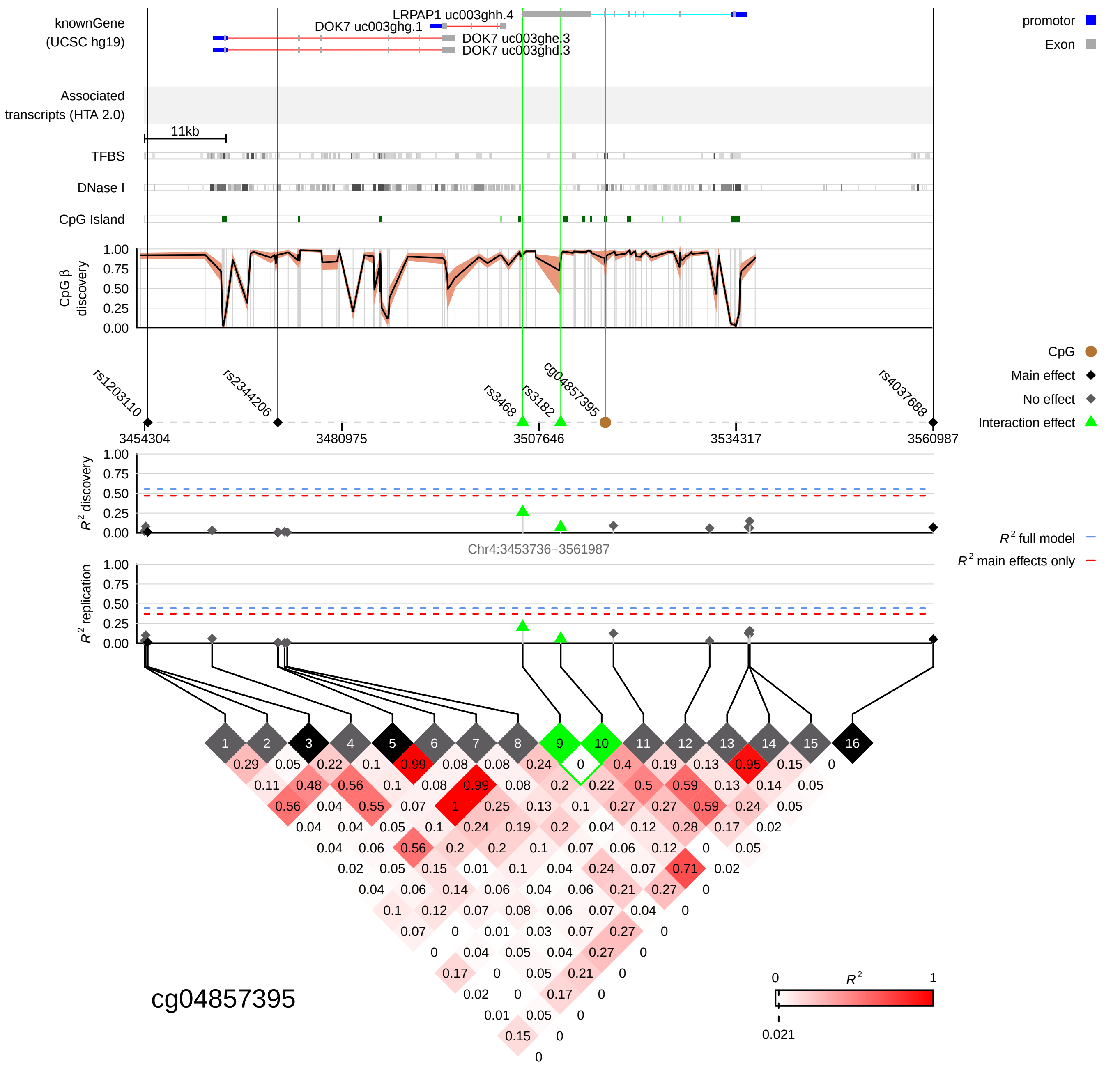
knownGene
(UCSC hg19)
LRPAP1 uc003ghh.4
DOK7 uc003ghg.1
DOK7 uc003ghe.3
DOK7 uc003ghd.3
promotor
Exon
Associated
transcripts (HTA 2.0)
11kb
TFBS
DNase I
CpG Island
CpG β
discovery
1.00
0.75
0.50
0.25
0.00
rs1203110
rs2344206
rs3468
rs3182
cg04857395
rs4037688
CpG
Main effect
No effect
Interaction effect
3454304
3480975
3507646
3534317
3560987
R2 discovery
Chr4:3453736−3561987
R2 full model
R2 main effects only
R2 replication
cg04857395
0
R2
1
0.021

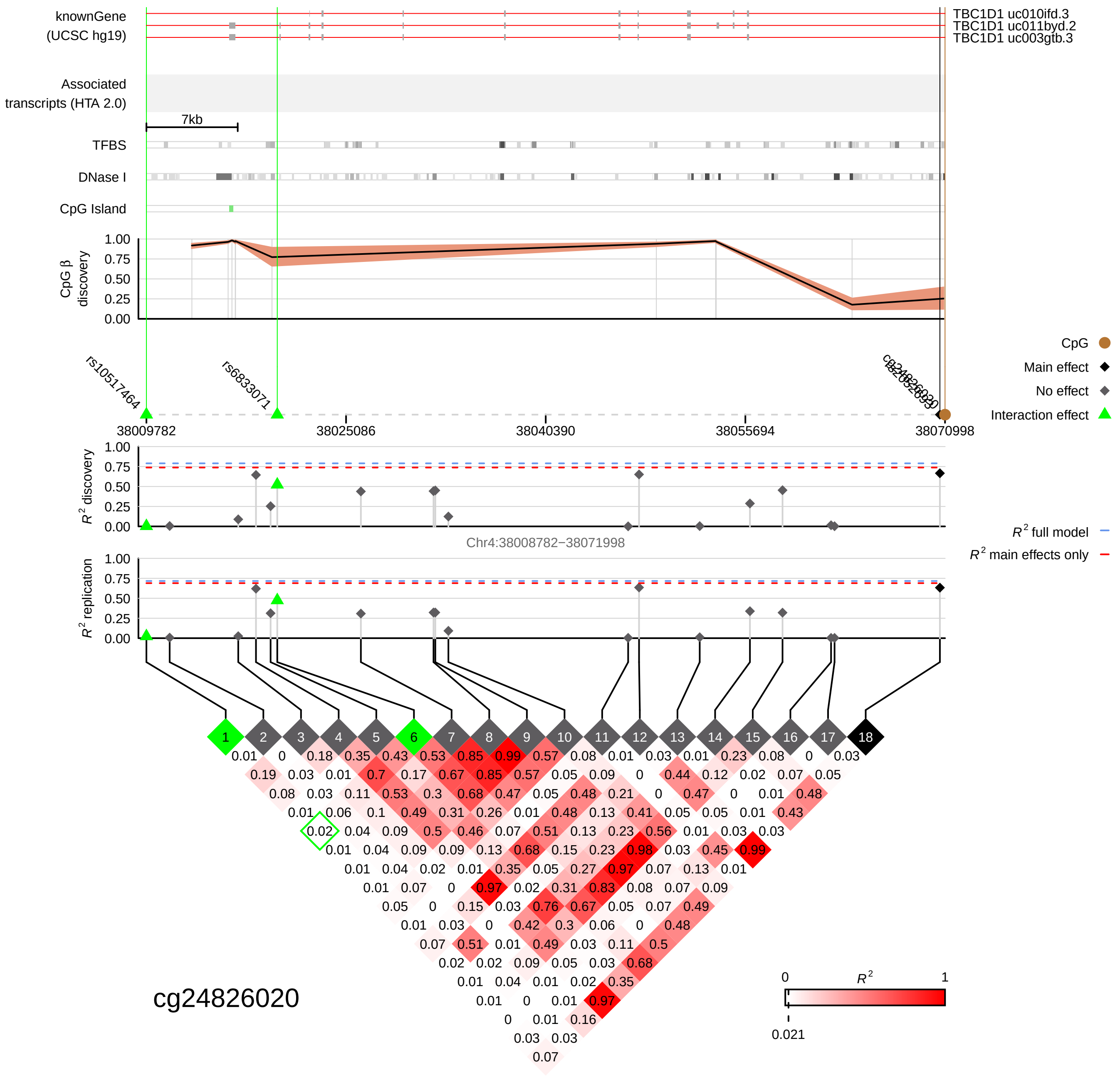

knownGene
(UCSC hg19)
TBC1D1 uc010ifd.3
TBC1D1 uc011byd.2
TBC1D1 uc003gtb.3
Associated
transcripts (HTA 2.0)
7kb
TFBS
DNase I
CpG Island
CpG β
discovery
rs10517464
rs6833071
38009782
38025086
38040390
38055694
38070998
CpG
Main effect
No effect
Interaction effect
R^2 discovery
Chr4:38008782−38071998
R^2 full model
R^2 main effects only
R^2 replication
R^2
0.021
cg24826020

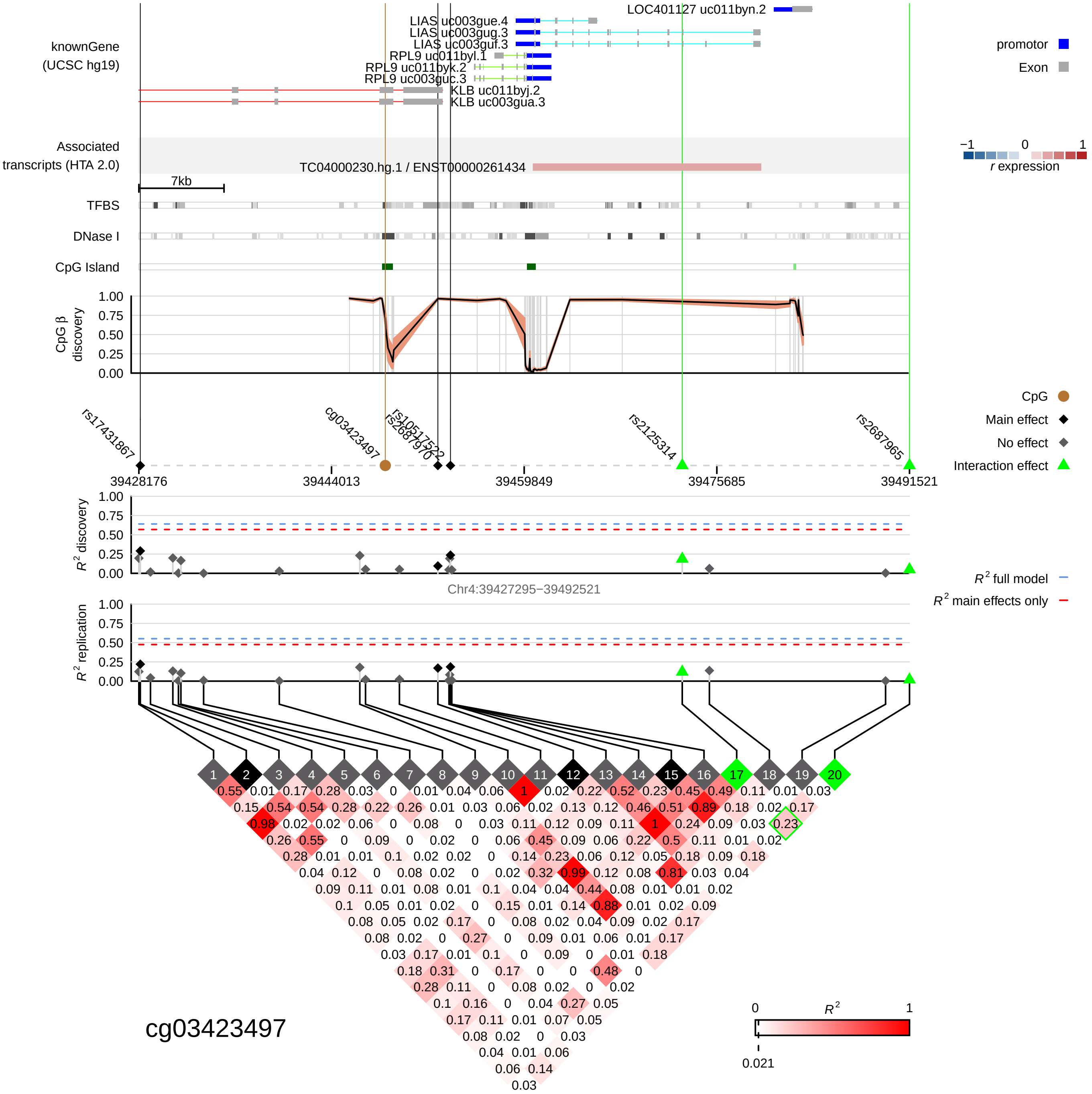

knownGene (UCSC hg19)
LOC401127 uc011byn.2
LIAS uc003gue.4
LIAS uc003gug.3
LIAS uc003guf.3
RPL9 uc011byl.1
RPL9 uc011byk.2
RPL9 uc003guc.3
KLB uc011byj.2
KLB uc003gua.3
promotor
Exon
Associated transcripts (HTA 2.0)
TC04000230.hg.1 / ENST00000261434
r expression
7kb
TFBS
DNase I
CpG Island
CpG β discovery
rs17431867
cg03423497
rs10517522
rs2687970
rs2125314
rs2687965
39428176
39444013
39459849
39475685
39491521
CpG
Main effect
No effect
Interaction effect
R² discovery
Chr4:39427295−39492521
R² full model
R² main effects only
R² replication
R²
0.021
cg03423497

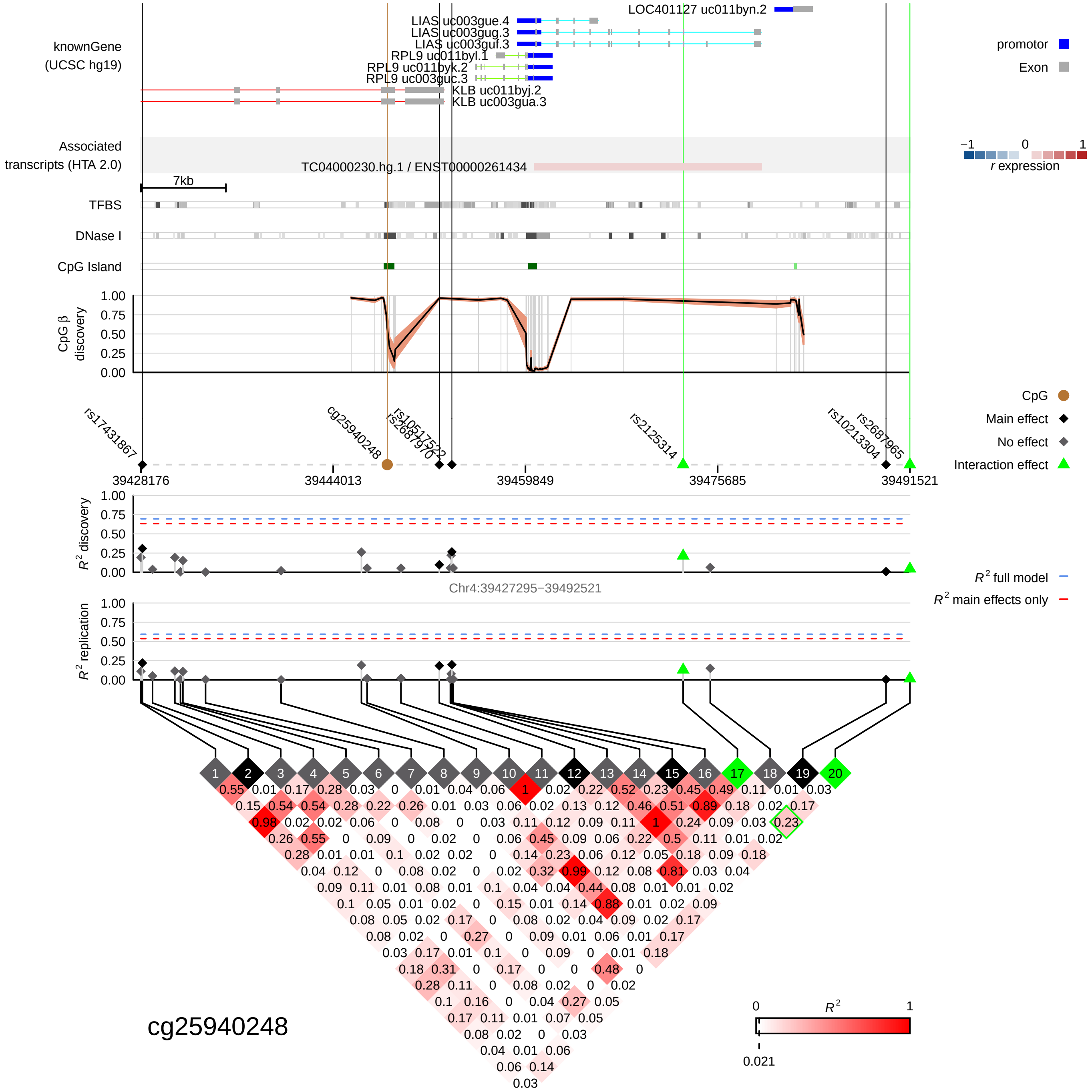

knownGene (UCSC hg19)
LOC401127 uc011byn.2
LIAS uc003gue.4
LIAS uc003gug.3
LIAS uc003guf.3
RPL9 uc011byl.1
RPL9 uc011byk.2
RPL9 uc003guc.3
KLB uc011byj.2
KLB uc003gua.3
promotor
Exon
Associated transcripts (HTA 2.0)
TC04000230.hg.1 / ENST00000261434
−1
0
1
r expression
7kb
TFBS
DNase I
CpG Island
CpG β discovery
1.00
0.75
0.50
0.25
0.00
rs17431867
cg25940248
rs10517522
rs2687970
rs2125314
rs10213304
rs2687965
CpG
Main effect
No effect
Interaction effect
39428176
39444013
39459849
39475685
39491521
R² discovery
Chr4:39427295−39492521
R² full model
R² main effects only
R² replication
R²
0
1
0.021
cg25940248

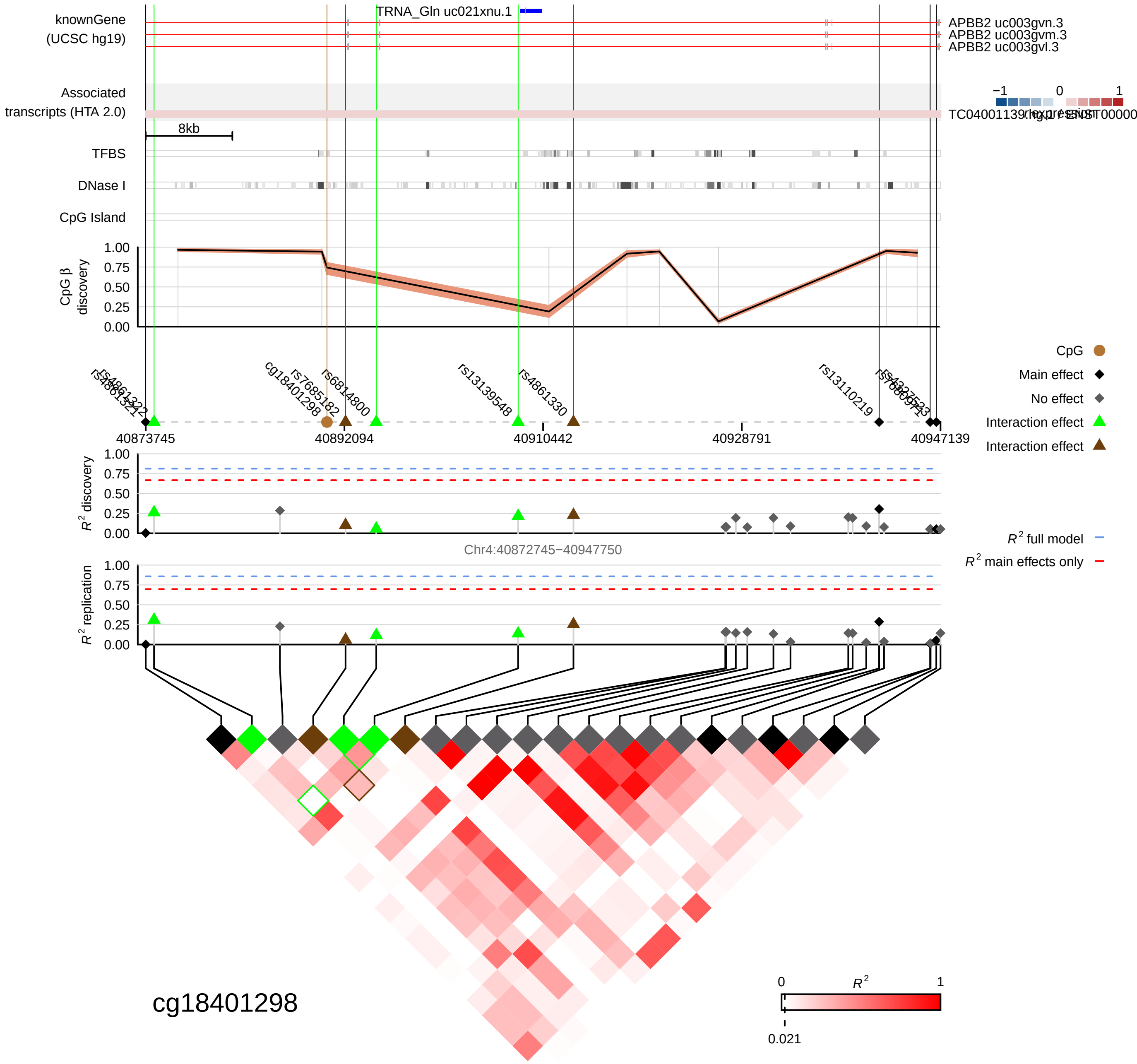

knownGene
(UCSC hg19)
TRNA_Gln uc021xnu.1
APBB2 uc003gvn.3
APBB2 uc003gvm.3
APBB2 uc003gvl.3
Associated
transcripts (HTA 2.0)
8kb
TFBS
DNase I
CpG Island
CpG β
discovery
rs13139548
rs4861330
rs13110219
40873745
40892094
40910442
40928791
40947139
CpG
Main effect
No effect
Interaction effect
Interaction effect
R^2 discovery
Chr4:40872745−40947750
R^2 replication
R^2 full model
R^2 main effects only
cg18401298
R^2
0.021

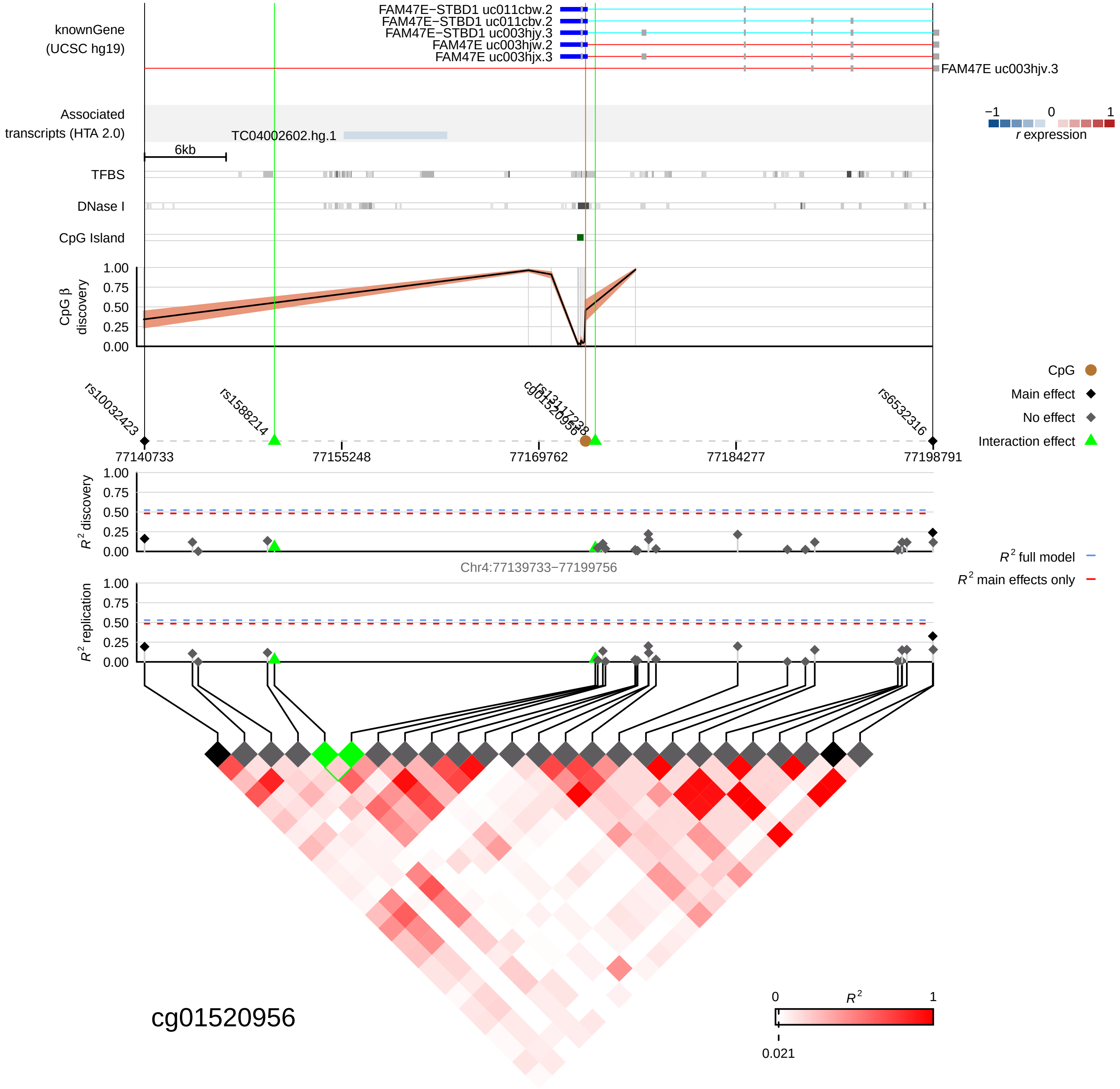

knownGene
(UCSC hg19)
FAM47E−STBD1 uc011cbw.2
FAM47E−STBD1 uc011cbv.2
FAM47E−STBD1 uc003hjy.3
FAM47E uc003hjw.2
FAM47E uc003hjx.3
FAM47E uc003hjv.3
Associated
transcripts (HTA 2.0)
TC04002602.hg.1
6kb
TFBS
DNase I
CpG Island
−1
0
1
r expression
CpG β
discovery
1.00
0.75
0.50
0.25
0.00
rs10032423
rs1588214
rs6532316
77140733
77155248
77169762
77184277
77198791
CpG
Main effect
No effect
Interaction effect
R² discovery
Chr4:77139733−77199756
R² full model
R² main effects only
R² replication
0
R²
1
0.021
cg01520956

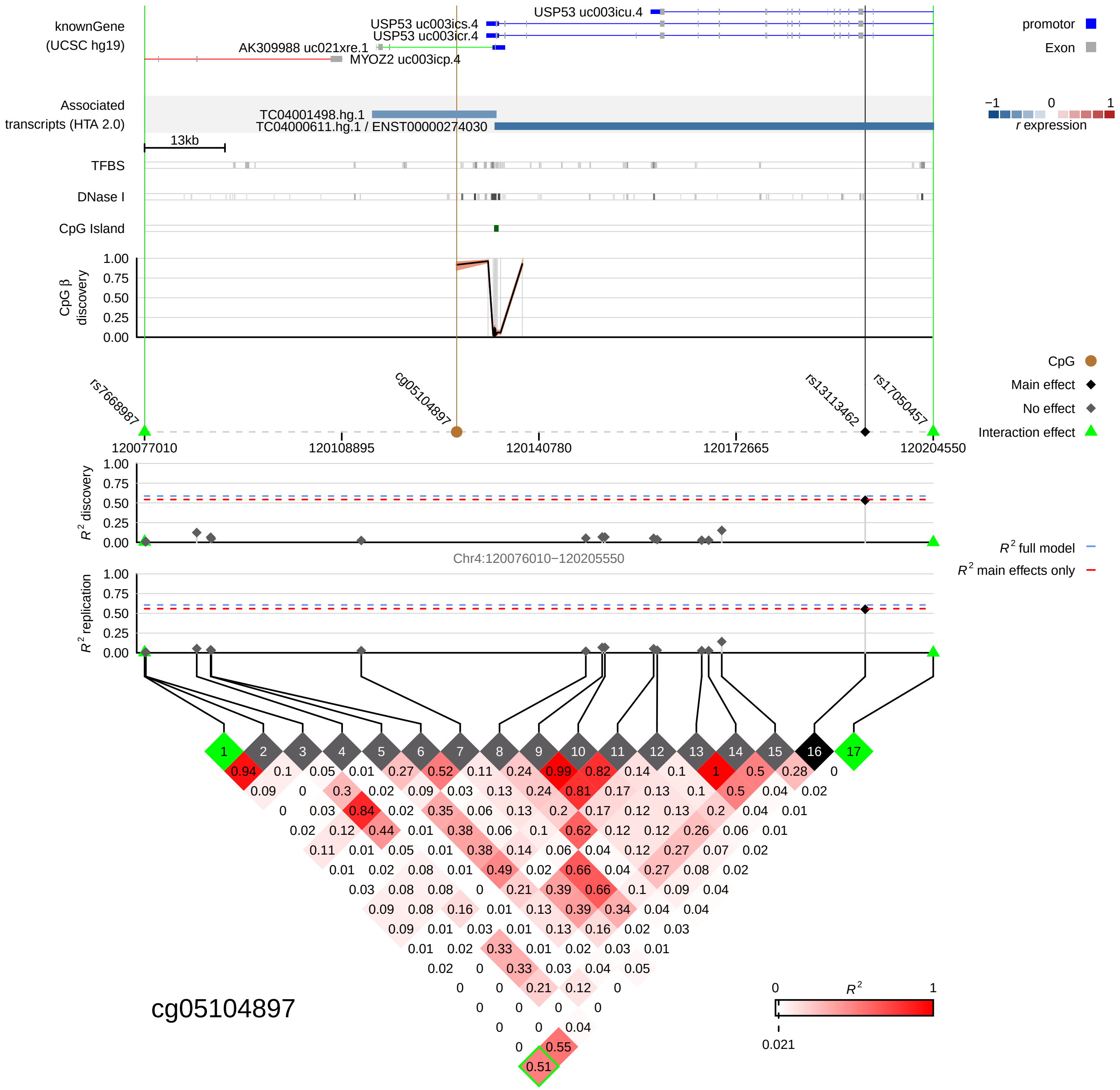

knownGene
(UCSC hg19)
USP53 uc003icu.4
USP53 uc003ics.4
USP53 uc003icr.4
AK309988 uc021xre.1
MYOZ2 uc003icp.4
promotor
Exon
Associated
transcripts (HTA 2.0)
TC04001498.hg.1
TC04000611.hg.1 / ENST00000274030
−1
0
1
r expression
13kb
TFBS
DNase I
CpG Island
CpG β
discovery
rs7668987
cg05104897
rs13113462
rs17050457
CpG
Main effect
No effect
Interaction effect
120077010
120108895
120140780
120172665
120204550
R² discovery
R² full model
R² main effects only
Chr4:120076010−120205550
R² replication
cg05104897
R²
0.021

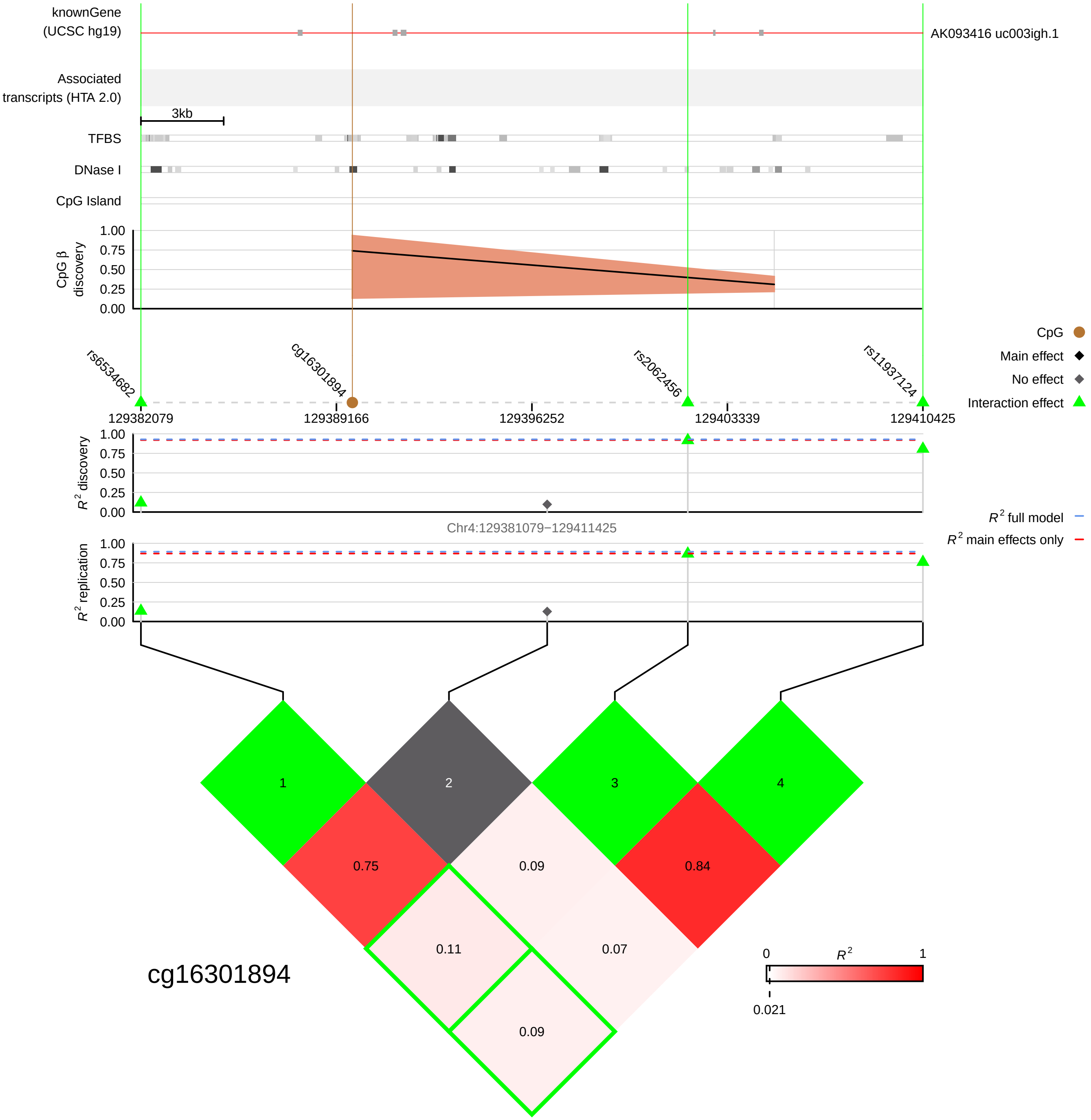

knownGene (UCSC hg19)
AK093416 uc003igh.1
Associated transcripts (HTA 2.0)
3kb
TFBS
DNase I
CpG Island
CpG β discovery
1.00
0.75
0.50
0.25
0.00
rs6534682
cg16301894
rs2062456
rs11937124
CpG
Main effect
No effect
Interaction effect
129382079
129389166
129396252
129403339
129410425
R^2 discovery
Chr4:129381079−129411425
R^2 full model
R^2 main effects only
R^2 replication
1
2
3
4
0.75
0.09
0.84
0.11
0.07
0.09
cg16301894
0
R^2
1
0.021

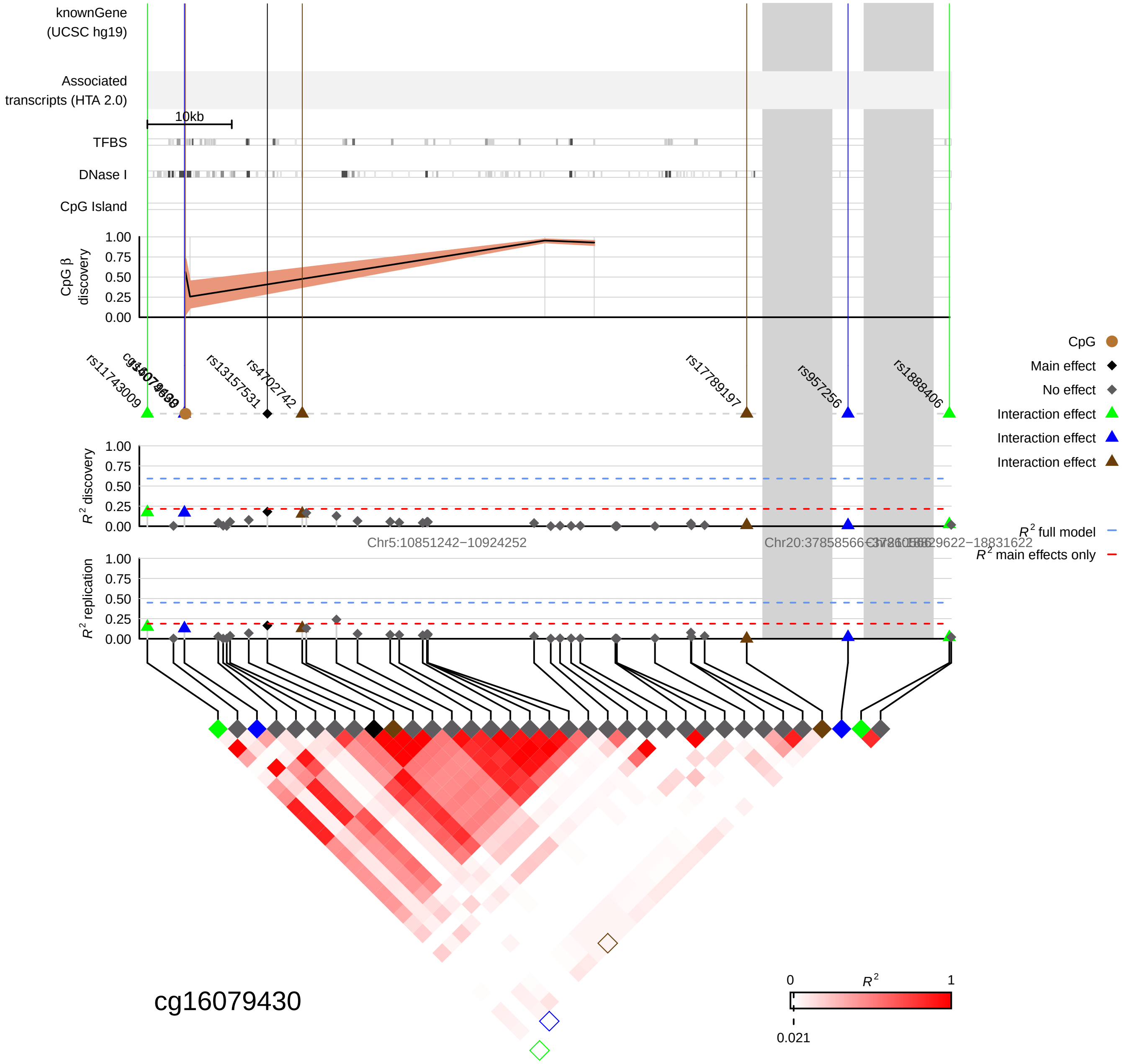
knownGene
(UCSC hg19)
Associated
transcripts (HTA 2.0)
10kb
TFBS
DNase I
CpG Island
CpG β
discovery
1.00
0.75
0.50
0.25
0.00
rs11743009
rs13157531
rs4702742
rs17789197
rs957256
rs1888406
CpG
Main effect
No effect
Interaction effect
Interaction effect
Interaction effect
R^2 discovery
Chr5:10851242−10924252
R^2 full model
R^2 main effects only
R^2 replication
0
R^2
1
0.021
cg16079430

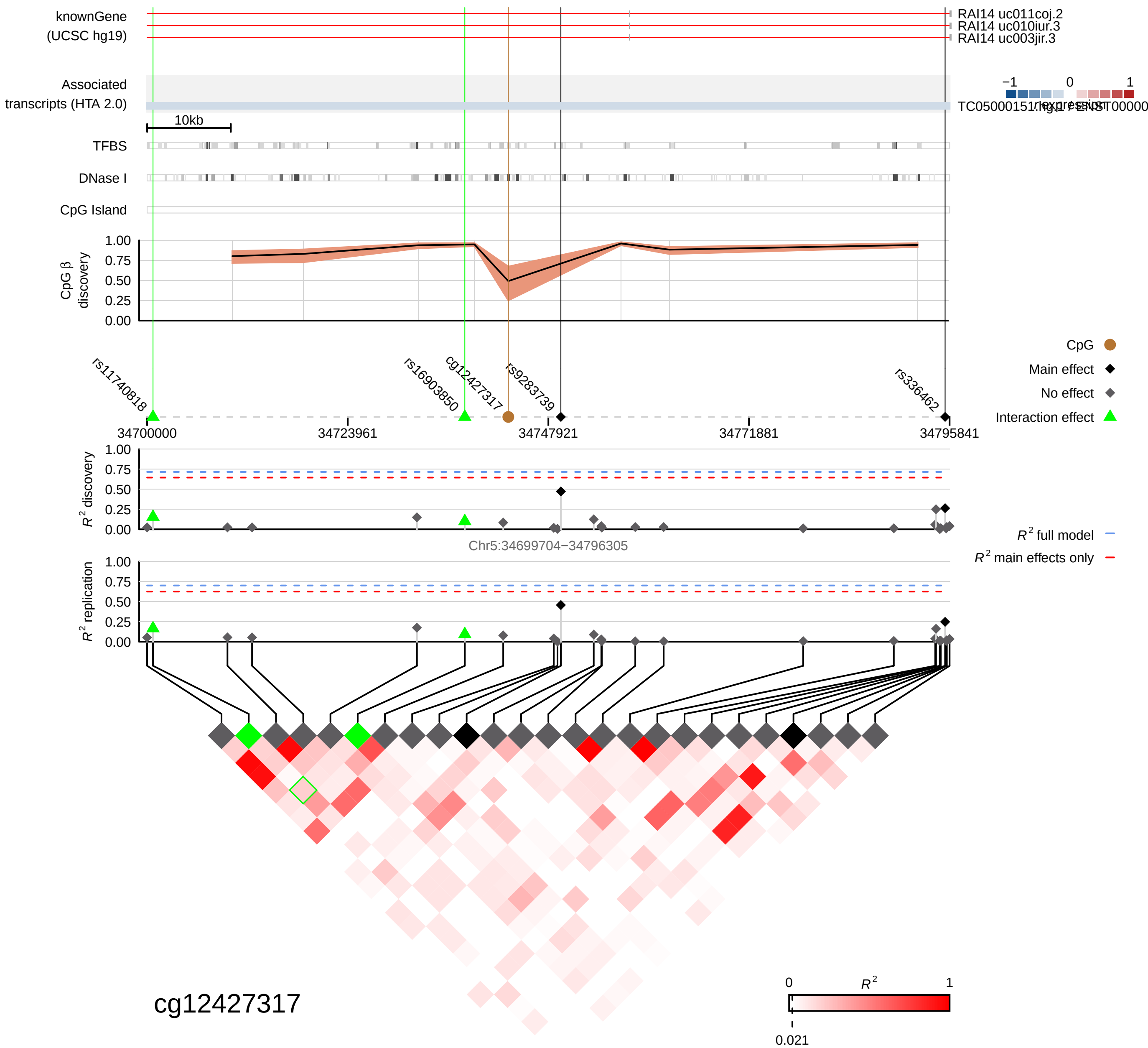

knownGene
(UCSC hg19)
RAI14 uc011coj.2
RAI14 uc010iur.3
RAI14 uc003jir.3
Associated
transcripts (HTA 2.0)
−1
0
1
10kb
TFBS
DNase I
CpG Island
CpG β
discovery
1.00
0.75
0.50
0.25
0.00
rs11740818
rs16903850
cg12427317
rs9283739
rs336462
CpG
Main effect
No effect
Interaction effect
34700000
34723961
34747921
34771881
34795841
R^2 discovery
Chr5:34699704−34796305
R^2 full model
R^2 main effects only
R^2 replication
0
R^2
1
0.021
cg12427317

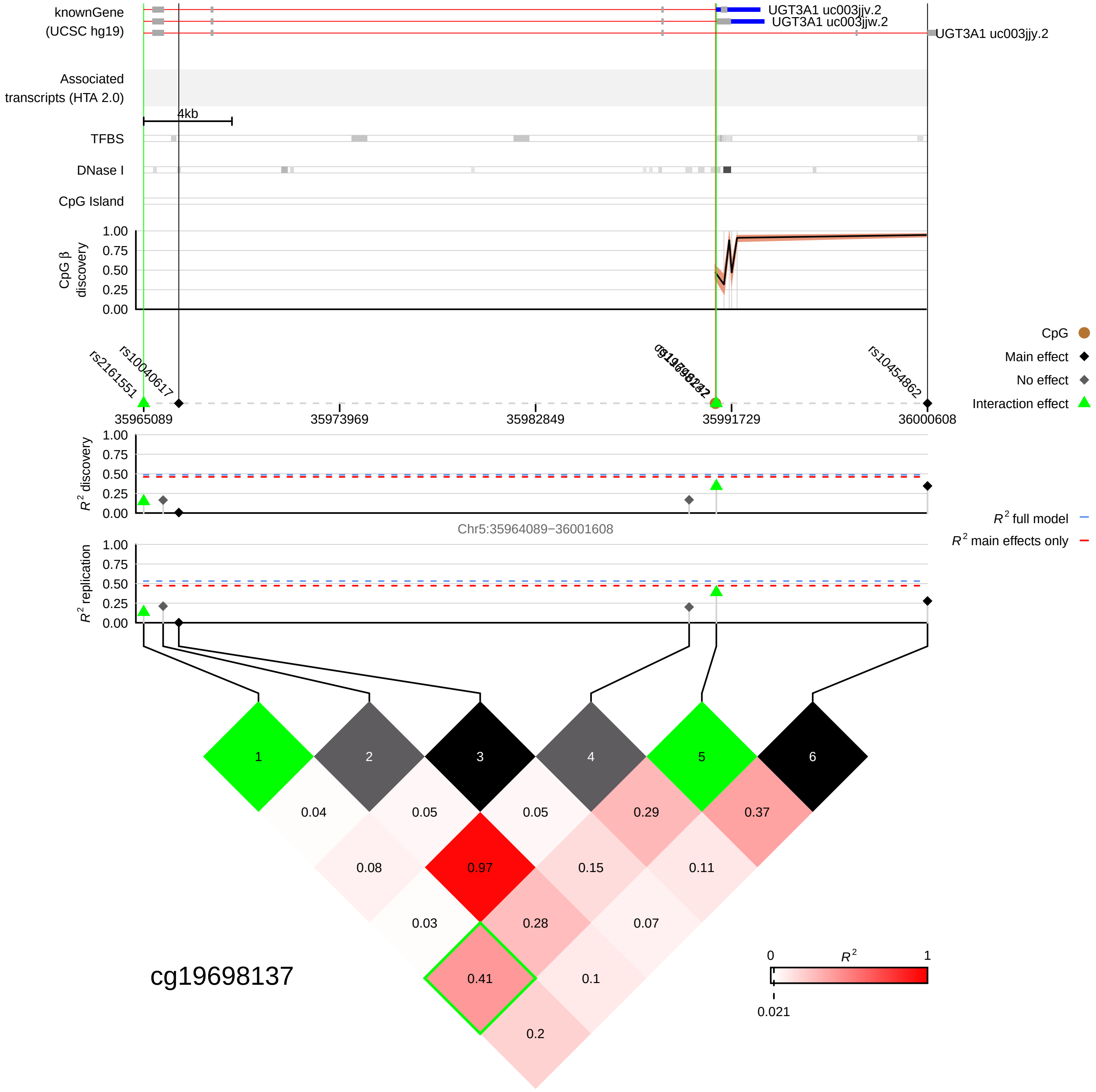

knownGene
(UCSC hg19)
UGT3A1 uc003jjv.2
UGT3A1 uc003jjw.2
UGT3A1 uc003jjy.2
Associated
transcripts (HTA 2.0)
4kb
TFBS
DNase I
CpG Island
CpG β
discovery
1.00
0.75
0.50
0.25
0.00
CpG
Main effect
No effect
Interaction effect
rs2161551
rs10040617
rs10454862
35965089
35973969
35982849
35991729
36000608
R² discovery
R² full model
R² main effects only
Chr5:35964089−36001608
R² replication
1
2
3
4
5
6
0.04
0.05
0.05
0.29
0.37
0.08
0.97
0.15
0.11
0.03
0.28
0.07
0.41
0.1
0.2
0
R²
1
0.021
cg19698137

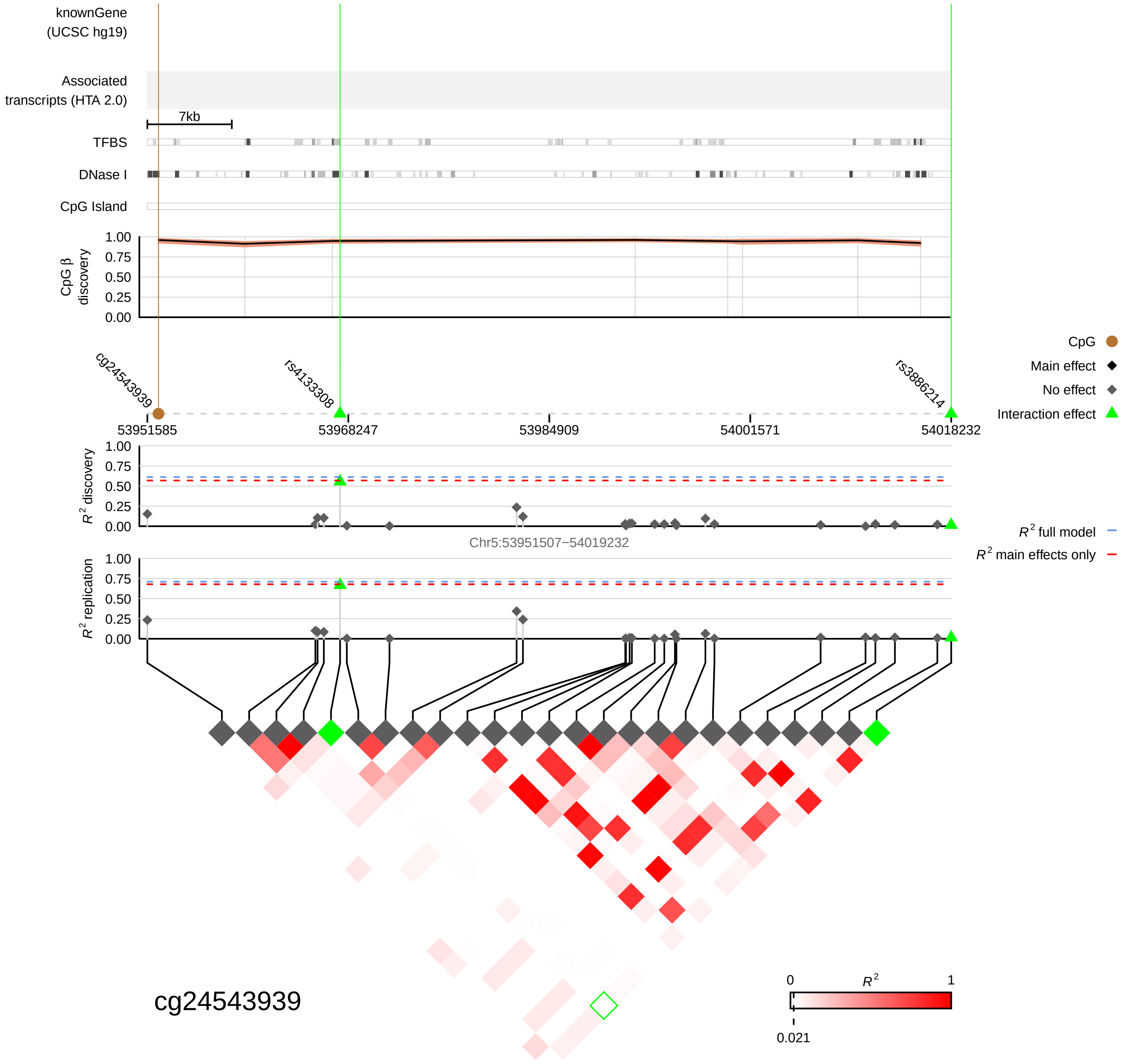

knownGene (UCSC hg19)
Associated transcripts (HTA 2.0)
7kb
TFBS
DNase I
CpG Island
CpG β discovery
cg24543939
rs4133308
rs3886214
53951585
53968247
53984909
54001571
54018232
CpG
Main effect
No effect
Interaction effect
R^2 discovery
Chr5:53951507−54019232
R^2 full model
R^2 main effects only
R^2 replication
0
R^2
1
0.021
cg24543939

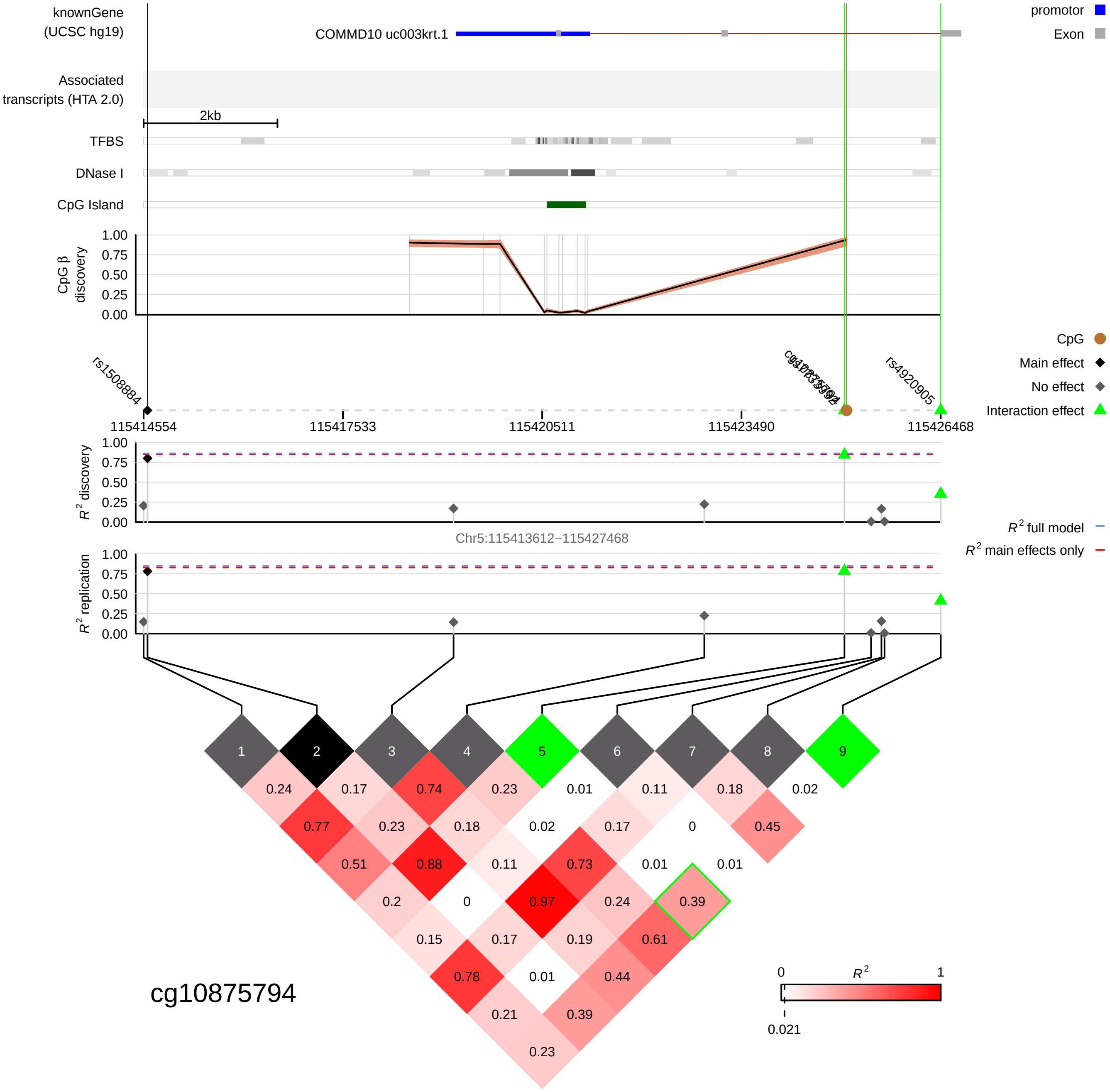
knownGene
(UCSC hg19)
COMMD10 uc003krt.1
promotor
Exon
Associated
transcripts (HTA 2.0)
2kb
TFBS
DNase I
CpG Island
CpG β
discovery
1.00
0.75
0.50
0.25
0.00
CpG
Main effect
No effect
Interaction effect
rs1508884
rs4920905
115414554
115417533
115420511
115423490
115426468
R^2 discovery
Chr5:115413612−115427468
R^2 full model
R^2 main effects only
R^2 replication
1
2
3
4
5
6
7
8
9
0.24
0.17
0.74
0.23
0.01
0.11
0.18
0.02
0.77
0.23
0.18
0.02
0.17
0
0.45
0.51
0.88
0.11
0.73
0.01
0.01
0.2
0
0.97
0.24
0.39
0.15
0.17
0.19
0.61
0.78
0.01
0.44
0.21
0.39
0.23
cg10875794
0
R^2
1
0.021

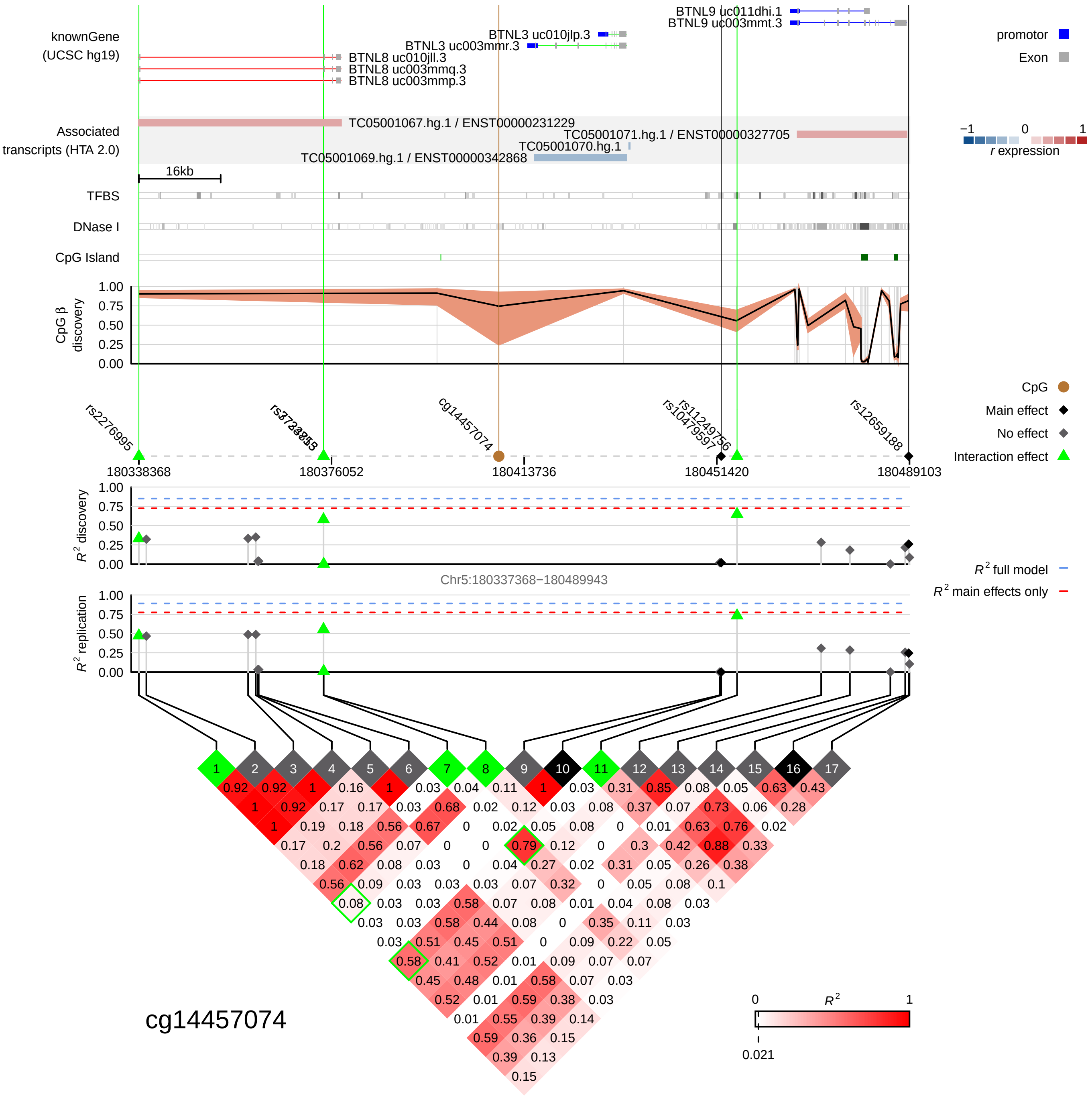

knownGene (UCSC hg19)
BTNL9 uc011dhi.1
BTNL9 uc003mmt.3
BTNL3 uc010jlp.3
BTNL3 uc003mmr.3
BTNL8 uc010jll.3
BTNL8 uc003mmq.3
BTNL8 uc003mmp.3
promotor
Exon
Associated transcripts (HTA 2.0)
TC05001067.hg.1 / ENST00000231229
TC05001071.hg.1 / ENST00000327705
TC05001070.hg.1
TC05001069.hg.1 / ENST00000342868
r expression
16kb
TFBS
DNase I
CpG Island
CpG β discovery
rs2276995
cg14457074
rs12659188
CpG
Main effect
No effect
Interaction effect
180338368
180376052
180413736
180451420
180489103
R^2 discovery
Chr5:180337368−180489943
R^2 full model
R^2 main effects only
R^2 replication
R^2
0.021
cg14457074

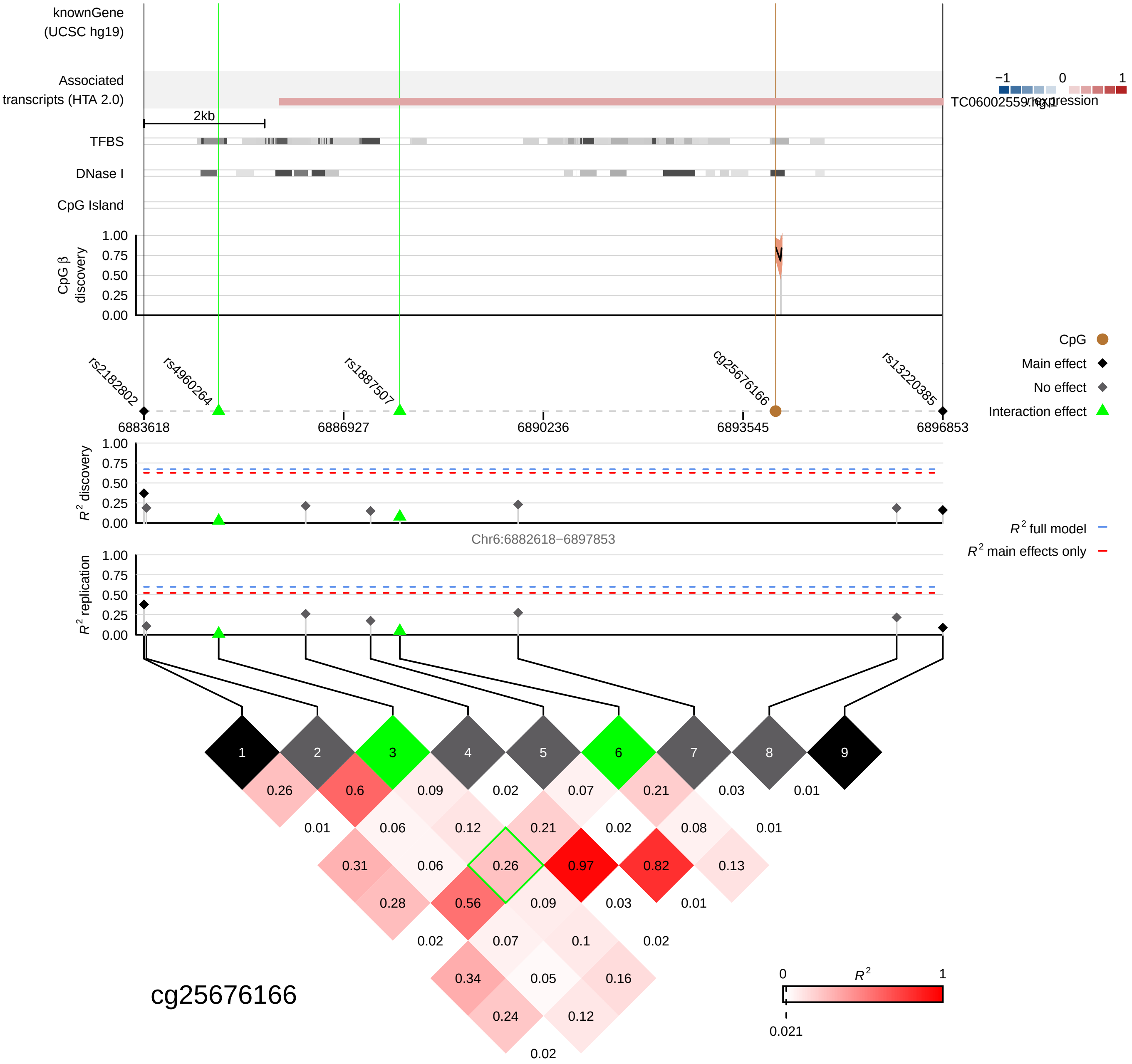

knownGene (UCSC hg19)
Associated transcripts (HTA 2.0)
TC06002559.hg.1
expression
−1
0
1
2kb
TFBS
DNase I
CpG Island
CpG β discovery
1.00
0.75
0.50
0.25
0.00
rs2182802
rs4960264
rs1887507
cg25676166
rs13220385
6883618
6886927
6890236
6893545
6896853
CpG
Main effect
No effect
Interaction effect
R² discovery
Chr6:6882618−6897853
R² full model
R² main effects only
R² replication
1
2
3
4
5
6
7
8
9
0.26
0.6
0.09
0.02
0.07
0.21
0.03
0.01
0.01
0.06
0.12
0.21
0.02
0.08
0.01
0.31
0.06
0.26
0.97
0.82
0.13
0.28
0.56
0.09
0.03
0.01
0.02
0.07
0.1
0.02
0.34
0.05
0.16
0.24
0.12
0.02
cg25676166
0.021

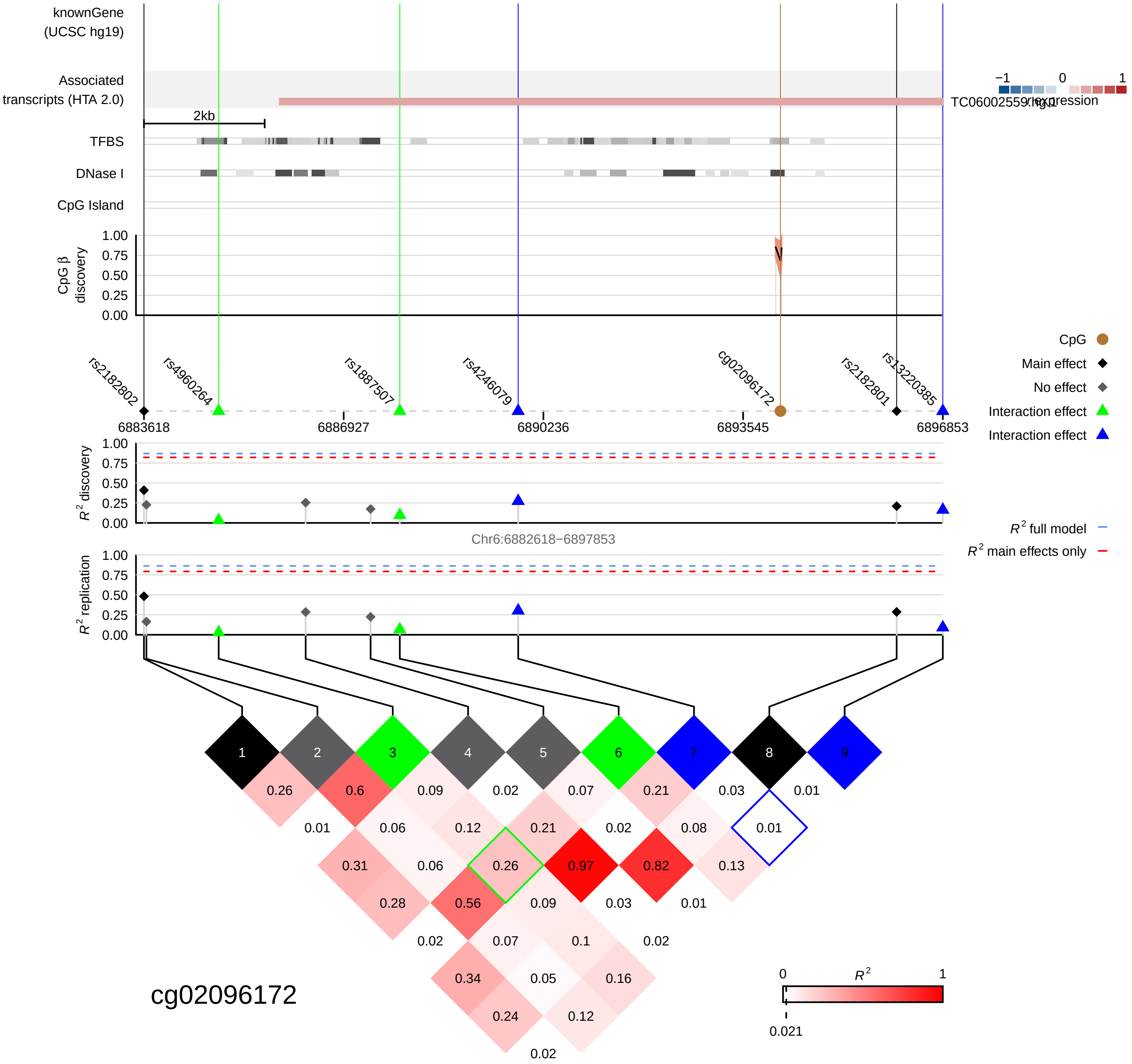
knownGene
(UCSC hg19)
Associated
transcripts (HTA 2.0)
TFBS
DNase I
CpG Island
CpG β
discovery
2kb
TC06002559.hg.1
−1
0
1
1.00
0.75
0.50
0.25
0.00
rs2182802
rs4960264
rs1887507
rs4246079
cg02096172
rs2182801
rs13220385
6883618
6886927
6890236
6893545
6896853
CpG
Main effect
No effect
Interaction effect
Interaction effect
R² discovery
Chr6:6882618−6897853
R² full model
R² main effects only
R² replication
1
2
3
4
5
6
7
8
9
0.26
0.6
0.09
0.02
0.07
0.21
0.03
0.01
0.01
0.06
0.12
0.21
0.02
0.08
0.01
0.31
0.06
0.26
0.97
0.82
0.13
0.28
0.56
0.09
0.03
0.01
0.02
0.07
0.1
0.02
0.34
0.05
0.16
0.24
0.12
0.02
cg02096172
0
R²
1
0.021

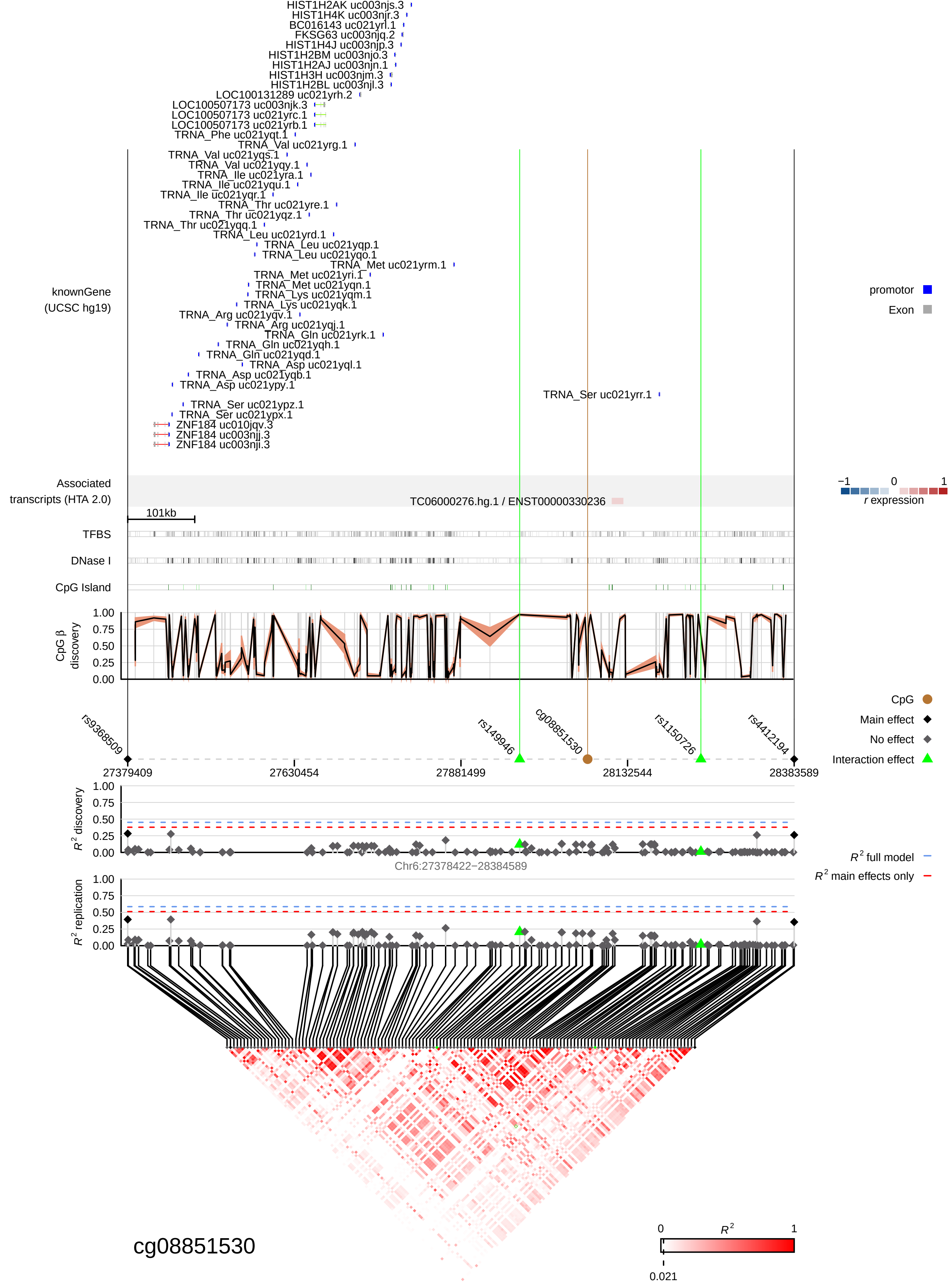
HIST1H2AK uc003njs.3
HIST1H4K uc003njr.3
BC016143 uc021yrl.1
FKSG63 uc003njq.2
HIST1H4J uc003njp.3
HIST1H2BM uc003njo.3
HIST1H2AJ uc003njn.1
HIST1H3H uc003njm.3
HIST1H2BL uc003njl.3
LOC100131289 uc021yrh.2
LOC100507173 uc003njk.3
LOC100507173 uc021yrc.1
LOC100507173 uc021yrb.1
TRNA_Phe uc021yqt.1
TRNA_Val uc021yrg.1
TRNA_Val uc021yqs.1
TRNA_Val uc021yqy.1
TRNA_Ile uc021yra.1
TRNA_Ile uc021yqu.1
TRNA_Ile uc021yqr.1
TRNA_Thr uc021yre.1
TRNA_Thr uc021yqz.1
TRNA_Thr uc021yqq.1
TRNA_Leu uc021yrd.1
TRNA_Leu uc021yqp.1
TRNA_Leu uc021yqo.1
TRNA_Met uc021yrm.1
TRNA_Met uc021yri.1
TRNA_Met uc021yqn.1
TRNA_Lys uc021yqm.1
TRNA_Lys uc021yqk.1
TRNA_Arg uc021yqv.1
TRNA_Arg uc021yqj.1
TRNA_Gln uc021yrk.1
TRNA_Gln uc021yqh.1
TRNA_Gln uc021yqd.1
TRNA_Asp uc021yql.1
TRNA_Asp uc021yqb.1
TRNA_Asp uc021ypy.1
TRNA_Ser uc021yrr.1
TRNA_Ser uc021ypz.1
TRNA_Ser uc021ypx.1
ZNF184 uc010jqv.3
ZNF184 uc003njj.3
ZNF184 uc003nji.3
knownGene (UCSC hg19)
promotor
Exon
Associated transcripts (HTA 2.0)
TC06000276.hg.1 / ENST00000330236
−1
0
1
r expression
101kb
TFBS
DNase I
CpG Island
CpG β discovery
1.00
0.75
0.50
0.25
0.00
rs9368509
rs149946
cg08851530
rs1150726
rs4412194
CpG
Main effect
No effect
Interaction effect
27379409
27630454
27881499
28132544
28383589
R^2 discovery
1.00
0.75
0.50
0.25
0.00
Chr6:27378422−28384589
R^2 full model
R^2 main effects only
R^2 replication
1.00
0.75
0.50
0.25
0.00
cg08851530
0
R^2
1
0.021

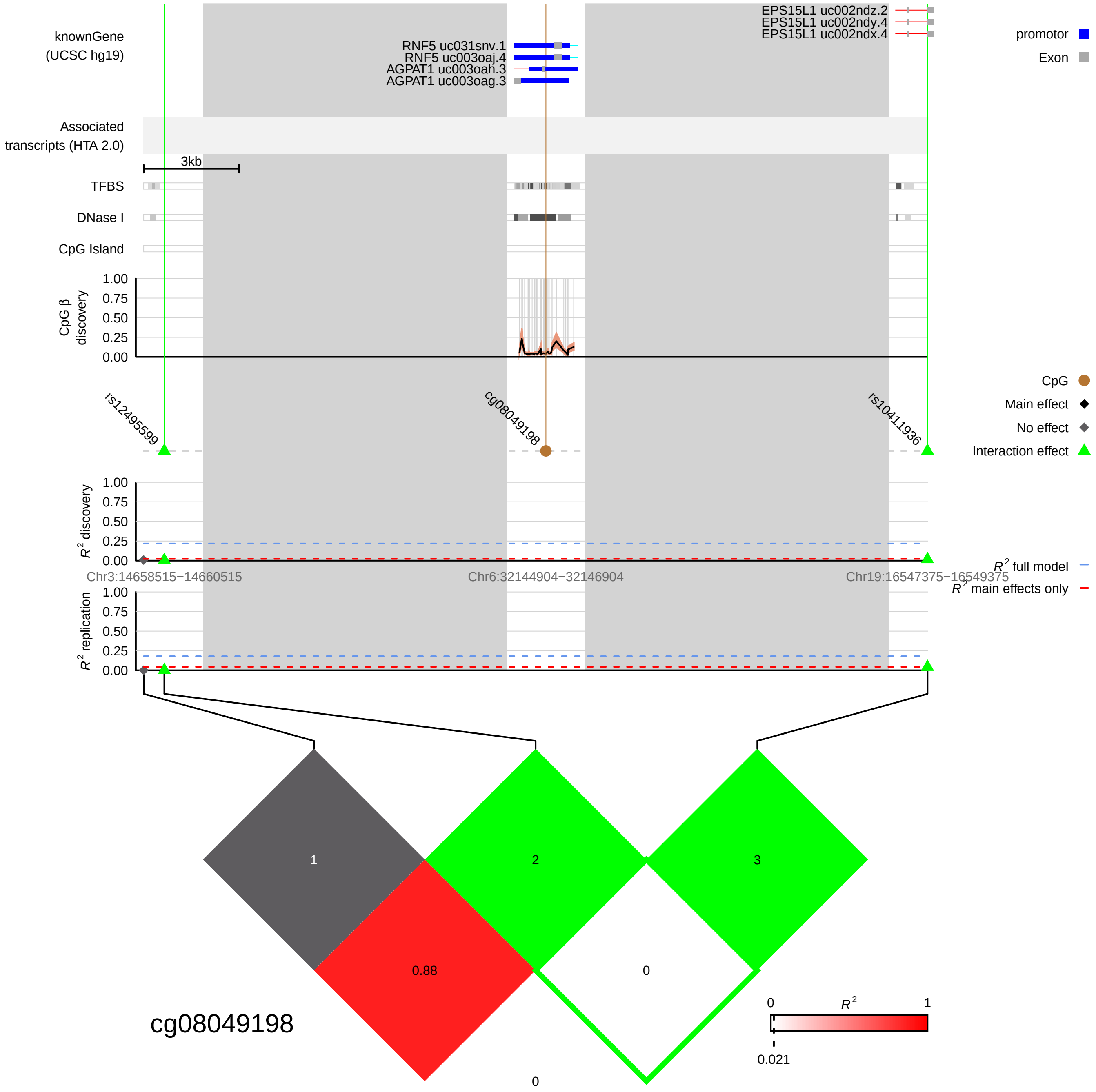

knownGene
(UCSC hg19)
EPS15L1 uc002ndz.2
EPS15L1 uc002ndy.4
EPS15L1 uc002ndx.4
RNF5 uc031snv.1
RNF5 uc003oaj.4
AGPAT1 uc003oah.3
AGPAT1 uc003oag.3
promotor
Exon
Associated
transcripts (HTA 2.0)
3kb
TFBS
DNase I
CpG Island
CpG β
discovery
1.00
0.75
0.50
0.25
0.00
rs12495599
cg08049198
rs10411936
CpG
Main effect
No effect
Interaction effect
R^2 discovery
R^2 full model
R^2 main effects only
Chr3:14658515−14660515
Chr6:32144904−32146904
Chr19:16547375−16549375
R^2 replication
1
2
3
0.88
0
0
0
R^2
1
0.021
cg08049198

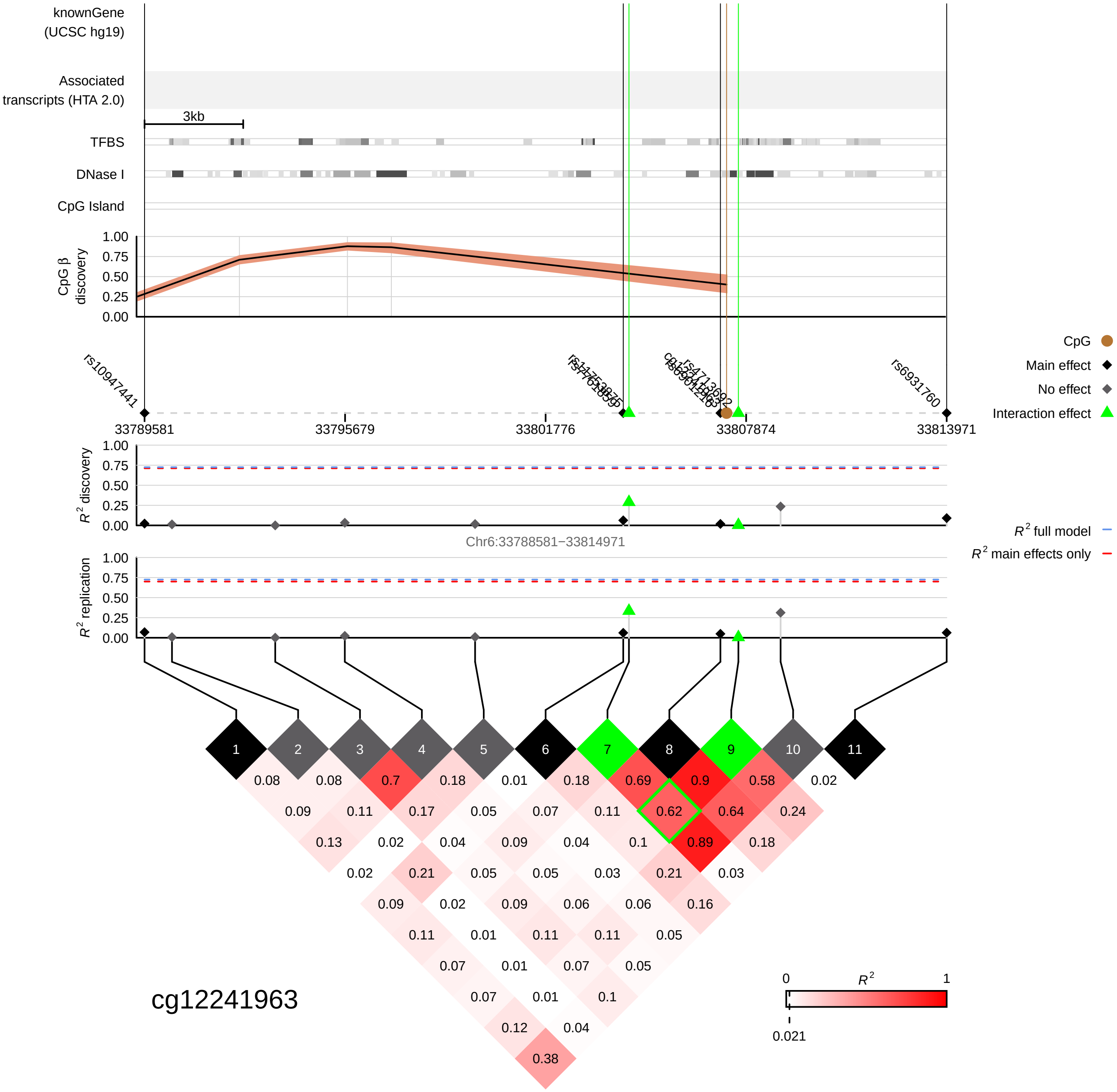

knownGene (UCSC hg19)
Associated transcripts (HTA 2.0)
3kb
TFBS
DNase I
CpG Island
CpG β discovery
1.00
0.75
0.50
0.25
0.00
rs10947441
rs6931760
33789581
33795679
33801776
33807874
33813971
CpG
Main effect
No effect
Interaction effect
R^2 discovery
Chr6:33788581−33814971
R^2 replication
R^2 full model
R^2 main effects only
1 2 3 4 5 6 7 8 9 10 11
0.08 0.08 0.7 0.18 0.01 0.18 0.69 0.9 0.58 0.02
0.09 0.11 0.17 0.05 0.07 0.11 0.62 0.64 0.24
0.13 0.02 0.04 0.09 0.04 0.1 0.89 0.18
0.02 0.21 0.05 0.05 0.03 0.21 0.03
0.09 0.02 0.09 0.06 0.06 0.16
0.11 0.01 0.11 0.11 0.05
0.07 0.01 0.07 0.05
0.07 0.01 0.1
0.12 0.04
0.38
cg12241963
0
R^2
1
0.021

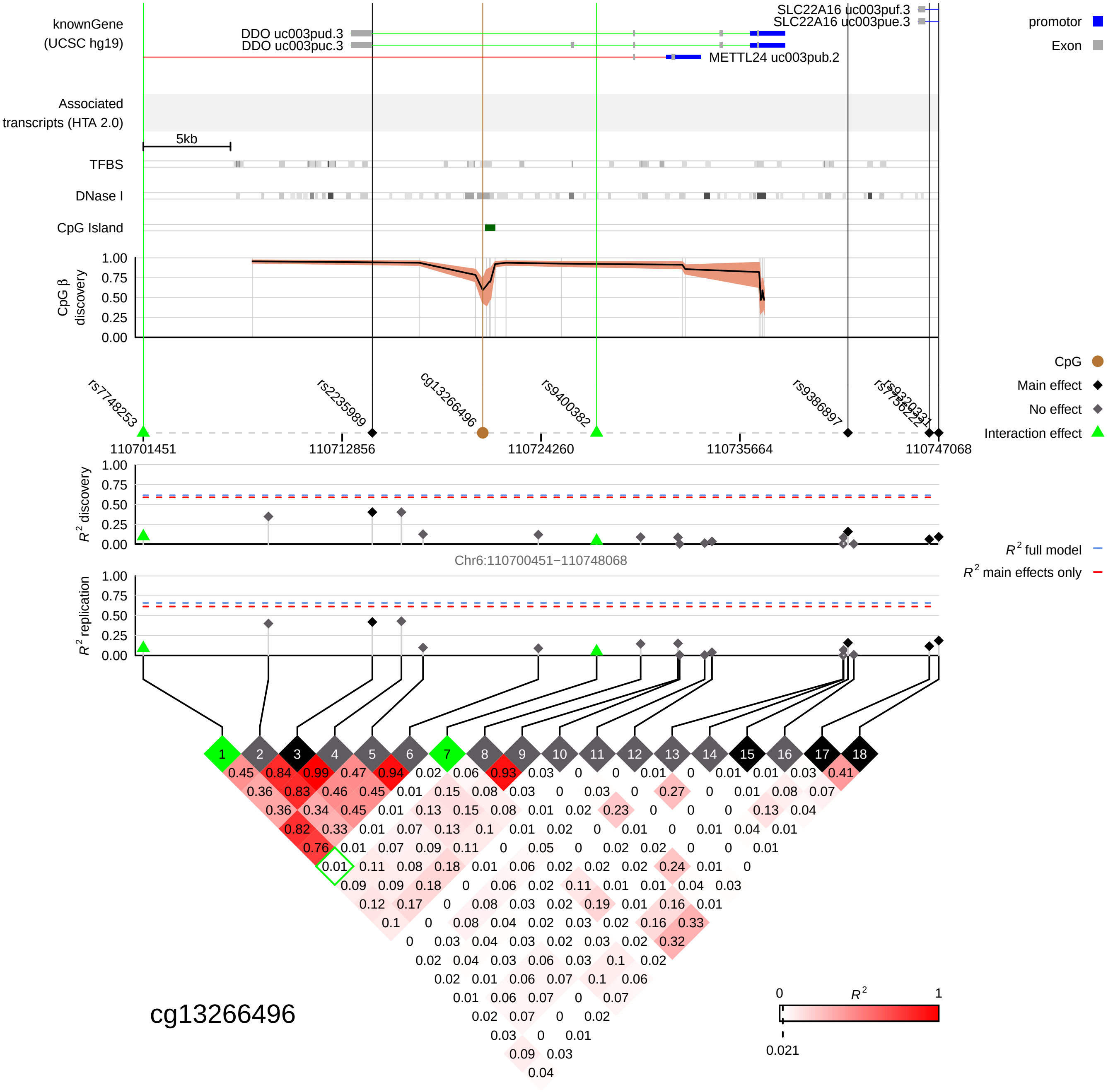
knownGene
(UCSC hg19)
SLC22A16 uc003puf.3
SLC22A16 uc003pue.3
DDO uc003pud.3
DDO uc003puc.3
METTL24 uc003pub.2
promotor
Exon
Associated
transcripts (HTA 2.0)
5kb
TFBS
DNase I
CpG Island
CpG β discovery
1.00
0.75
0.50
0.25
0.00
rs7748253
rs2235989
cg13266496
rs9400382
rs9386897
CpG
Main effect
No effect
Interaction effect
110701451
110712856
110724260
110735664
110747068
R² discovery
Chr6:110700451−110748068
R² full model
R² main effects only
R² replication
1 2 3 4 5 6 7 8 9 10 11 12 13 14 15 16 17 18
cg13266496
0
R²
1
0.021

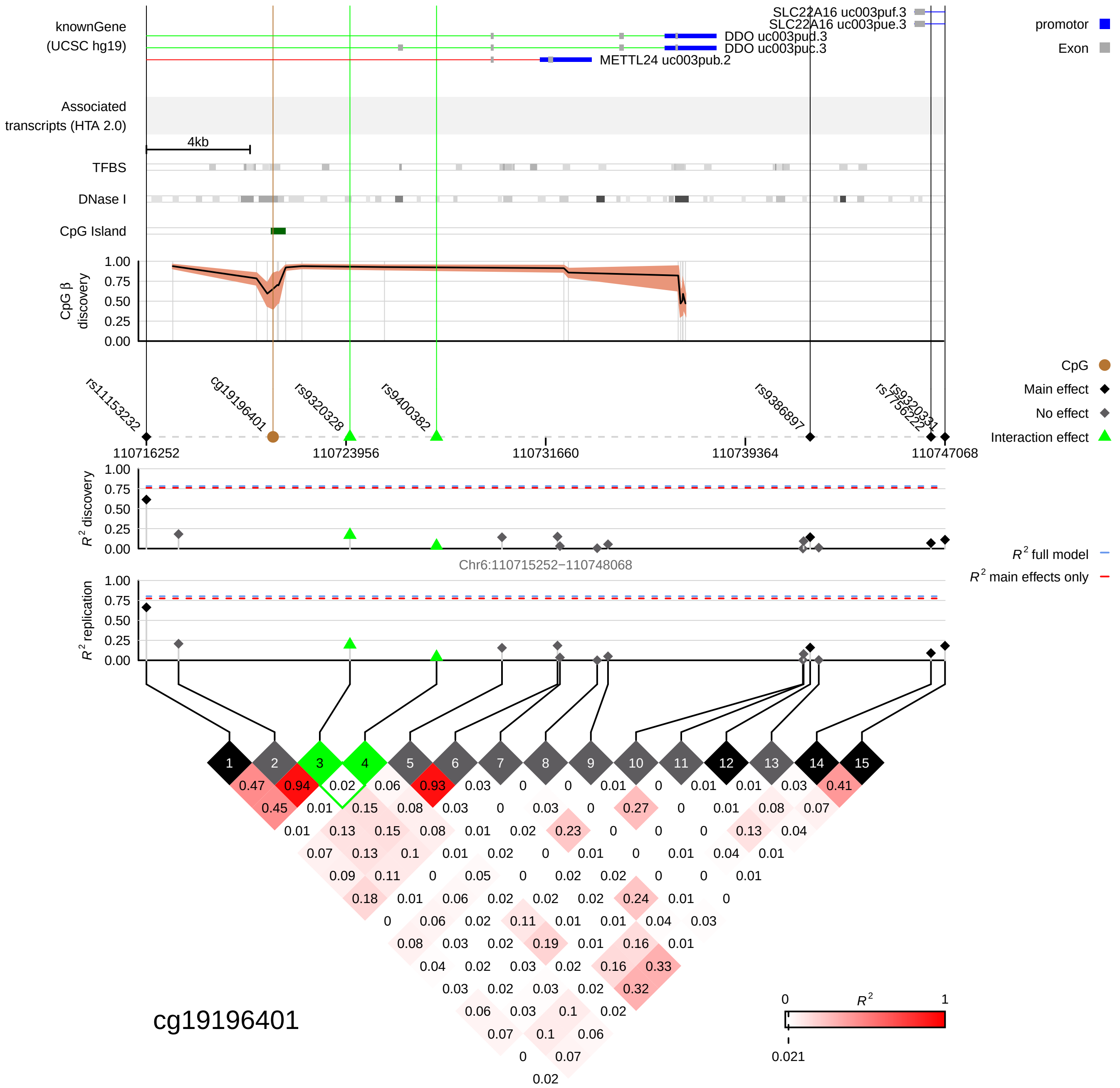

knownGene (UCSC hg19)
SLC22A16 uc003puf.3
SLC22A16 uc003pue.3
DDO uc003pud.3
DDO uc003puc.3
METTL24 uc003pub.2
promotor
Exon
Associated transcripts (HTA 2.0)
4kb
TFBS
DNase I
CpG Island
CpG β discovery
rs11153232
cg19196401
rs9320328
rs9400382
rs9386897
rs7756222
rs9320331
CpG
Main effect
No effect
Interaction effect
110716252
110723956
110731660
110739364
110747068
R^2 discovery
Chr6:110715252−110748068
R^2 full model
R^2 main effects only
R^2 replication
0
R^2
1
0.021
cg19196401

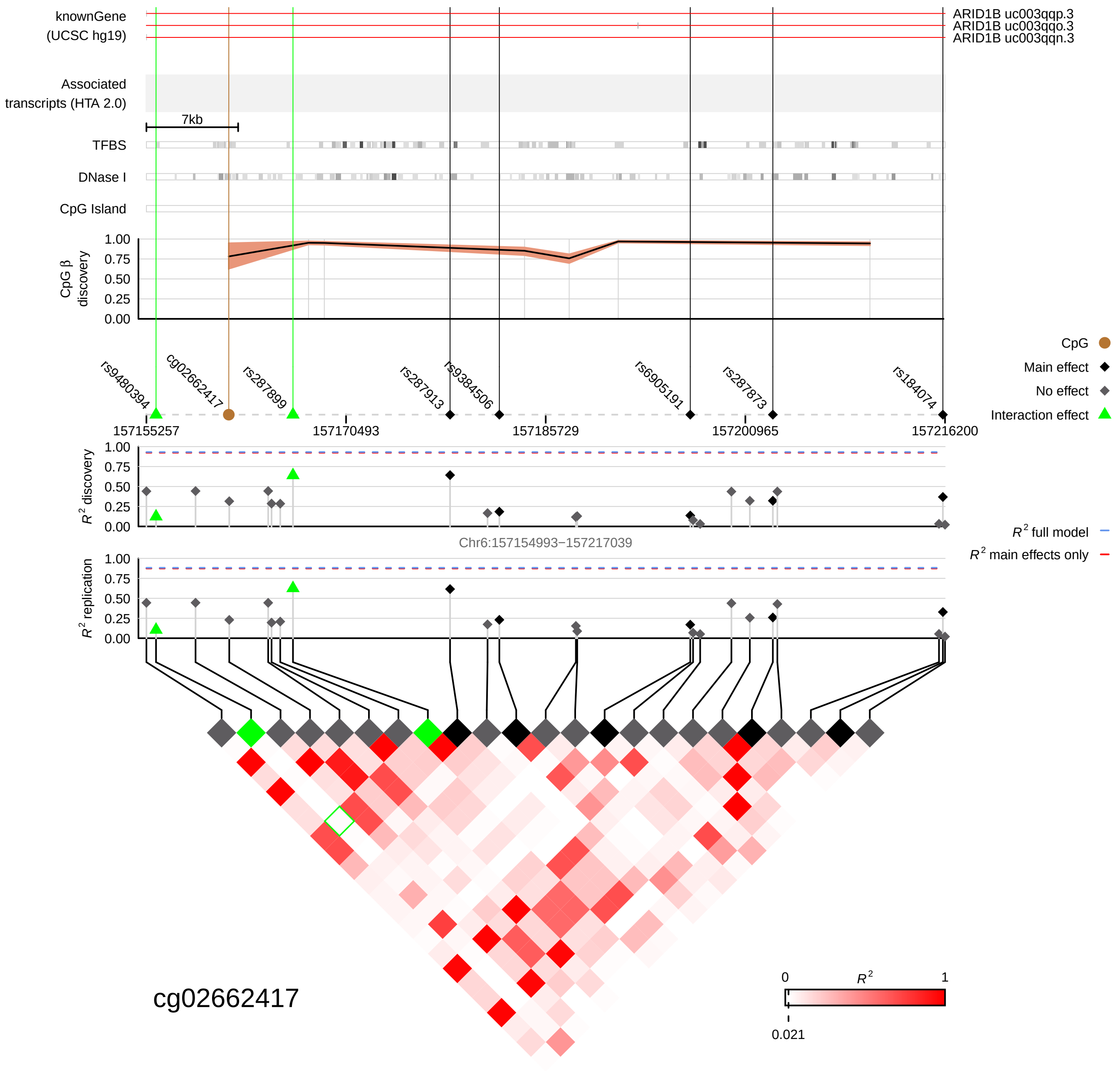

knownGene
(UCSC hg19)
ARID1B uc003qqp.3
ARID1B uc003qqo.3
ARID1B uc003qqn.3
Associated
transcripts (HTA 2.0)
7kb
TFBS
DNase I
CpG Island
CpG β
discovery
1.00
0.75
0.50
0.25
0.00
rs9480394
cg02662417
rs287899
rs287913
rs9384506
rs6905191
rs287873
rs184074
157155257
157170493
157185729
157200965
157216200
CpG
Main effect
No effect
Interaction effect
R2 discovery
Chr6:157154993−157217039
R2 full model
R2 main effects only
R2 replication
cg02662417
0
R2
1
0.021

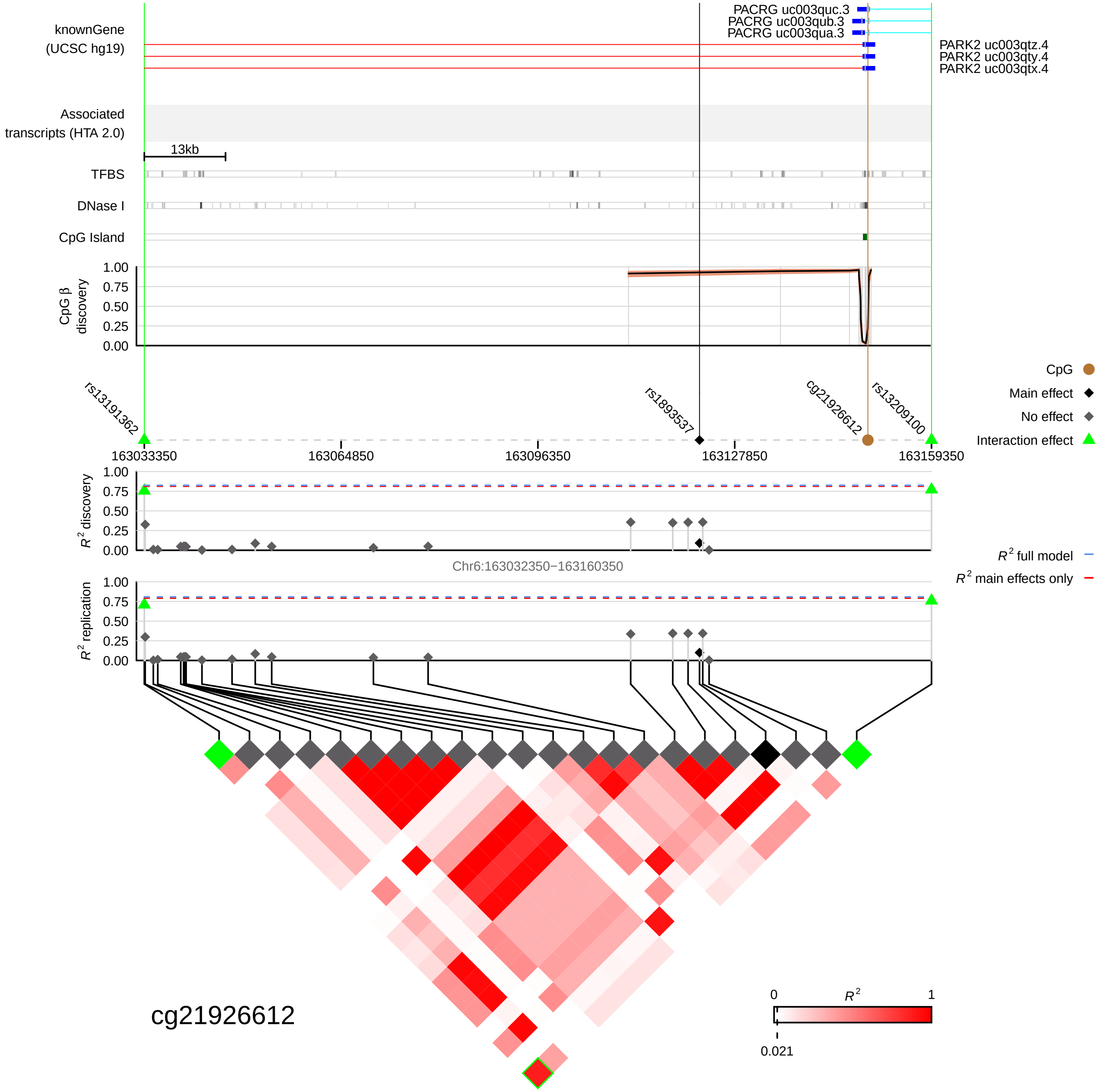

knownGene
(UCSC hg19)
PACRG uc003quc.3
PACRG uc003qub.3
PACRG uc003qua.3
PARK2 uc003qtz.4
PARK2 uc003qty.4
PARK2 uc003qtx.4
Associated
transcripts (HTA 2.0)
13kb
TFBS
DNase I
CpG Island
CpG β
discovery
1.00
0.75
0.50
0.25
0.00
rs13191362
rs1893537
cg21926612
rs13209100
CpG
Main effect
No effect
Interaction effect
163033350
163064850
163096350
163127850
163159350
R² discovery
Chr6:163032350−163160350
R² full model
R² main effects only
R² replication
cg21926612
0
R²
1
0.021

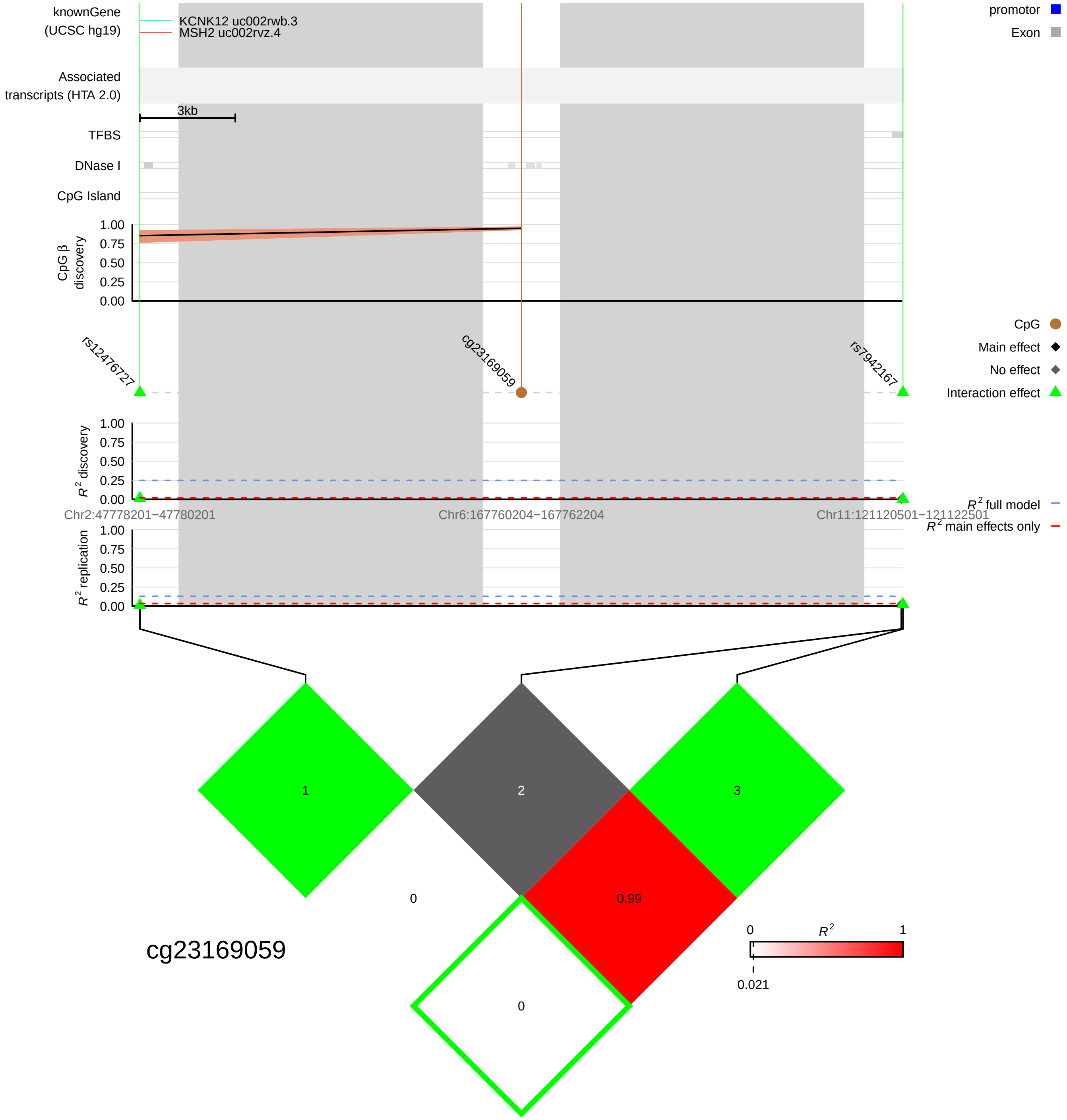

knownGene (UCSC hg19)
KCNK12 uc002rwb.3
MSH2 uc002rvz.4
Associated transcripts (HTA 2.0)
3kb
TFBS
DNase I
CpG Island
CpG β discovery
1.00
0.75
0.50
0.25
0.00
rs12476727
cg23169059
rs7942167
R^2 discovery
R^2 replication
Chr2:47778201−47780201
Chr6:167760204−167762204
Chr11:121120501−121122501
promotor
Exon
CpG
Main effect
No effect
Interaction effect
R^2 full model
R^2 main effects only
1
2
3
0
0.99
0
R^2
0
1
0.021
cg23169059

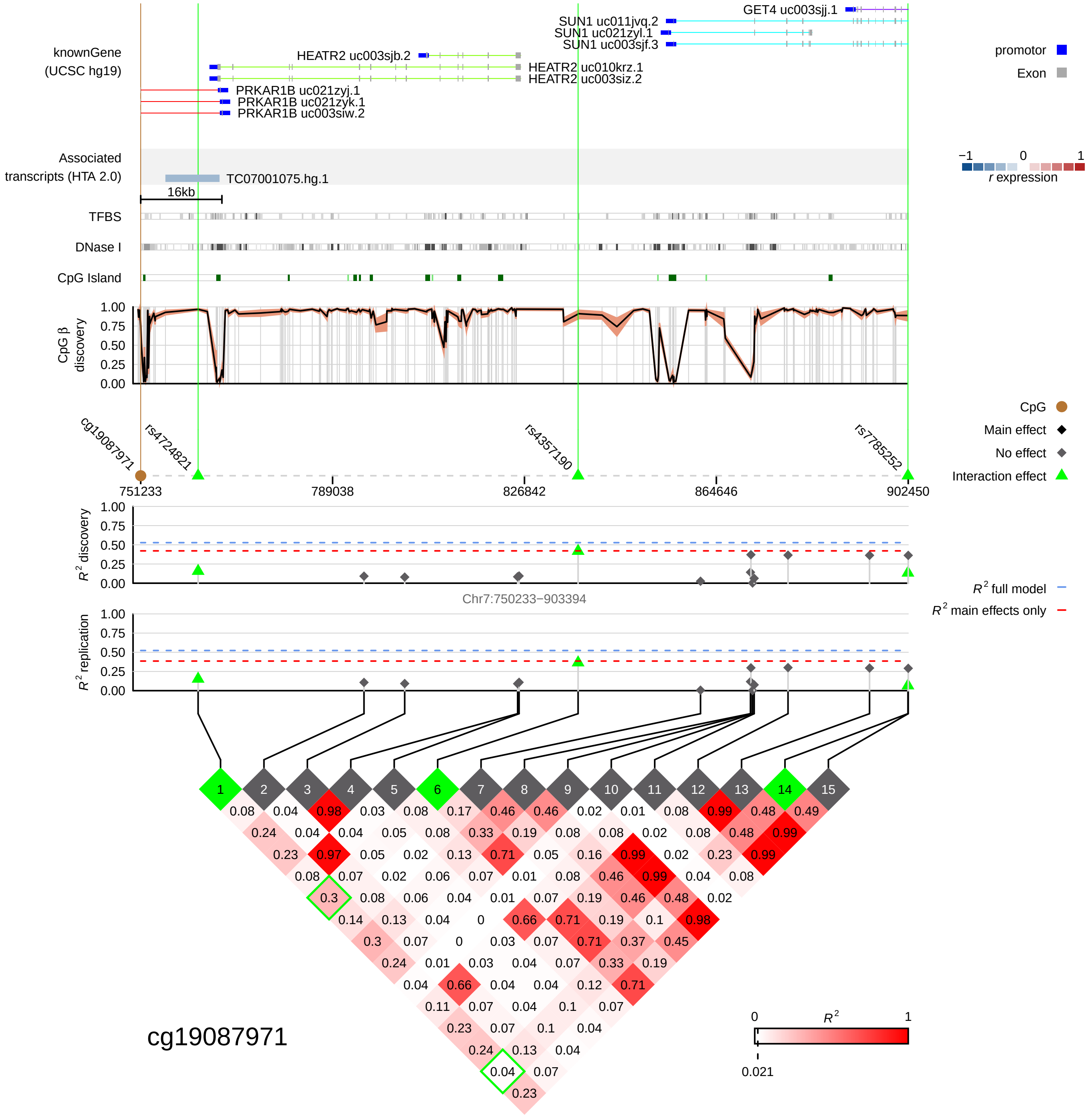
knownGene (UCSC hg19)
GET4 uc003sjj.1
SUN1 uc011jvq.2
SUN1 uc021zyl.1
SUN1 uc003sjf.3
HEATR2 uc003sjb.2
HEATR2 uc010krz.1
HEATR2 uc003siz.2
PRKAR1B uc021zyj.1
PRKAR1B uc021zyk.1
PRKAR1B uc003siw.2
promotor
Exon
Associated transcripts (HTA 2.0)
TC07001075.hg.1
16kb
r expression
TFBS
DNase I
CpG Island
CpG β discovery
cg19087971
rs4724821
rs4357190
rs7785252
751233
789038
826842
864646
902450
CpG
Main effect
No effect
Interaction effect
R^2 discovery
Chr7:750233−903394
R^2 full model
R^2 main effects only
R^2 replication
R^2
0.021
cg19087971

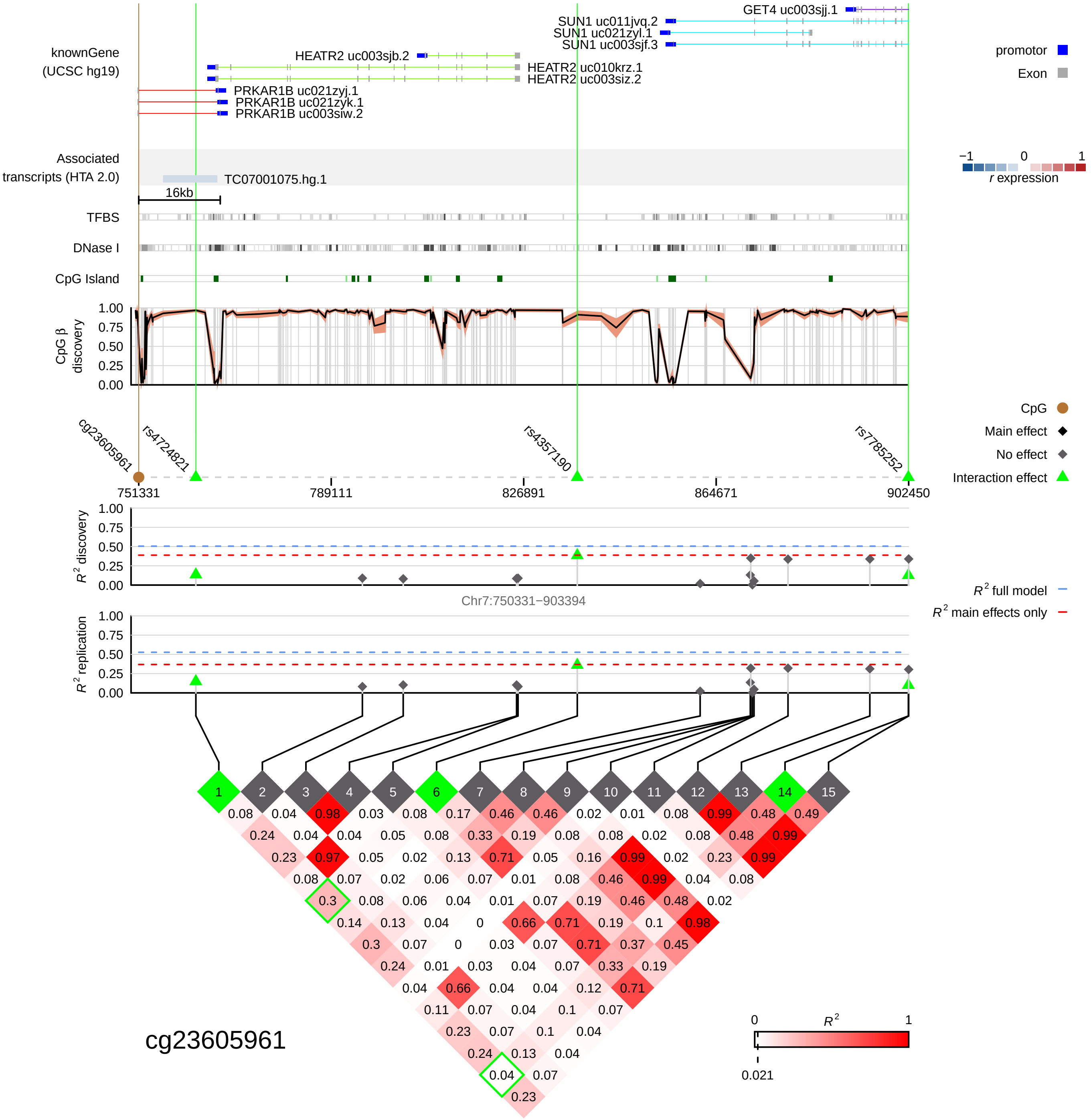
knownGene
(UCSC hg19)
GET4 uc003sjj.1
SUN1 uc011jvq.2
SUN1 uc021zyl.1
SUN1 uc003sjf.3
HEATR2 uc003sjb.2
HEATR2 uc010krz.1
HEATR2 uc003siz.2
PRKAR1B uc021zyj.1
PRKAR1B uc021zyk.1
PRKAR1B uc003siw.2
promotor
Exon
Associated
transcripts (HTA 2.0)
TC07001075.hg.1
16kb
−1
0
1
r expression
TFBS
DNase I
CpG Island
CpG β
discovery
cg23605961
rs4724821
rs4357190
rs7785252
751331
789111
826891
864671
902450
CpG
Main effect
No effect
Interaction effect
R² discovery
Chr7:750331−903394
R² full model
R² main effects only
R² replication
0
R²
1
0.021
cg23605961

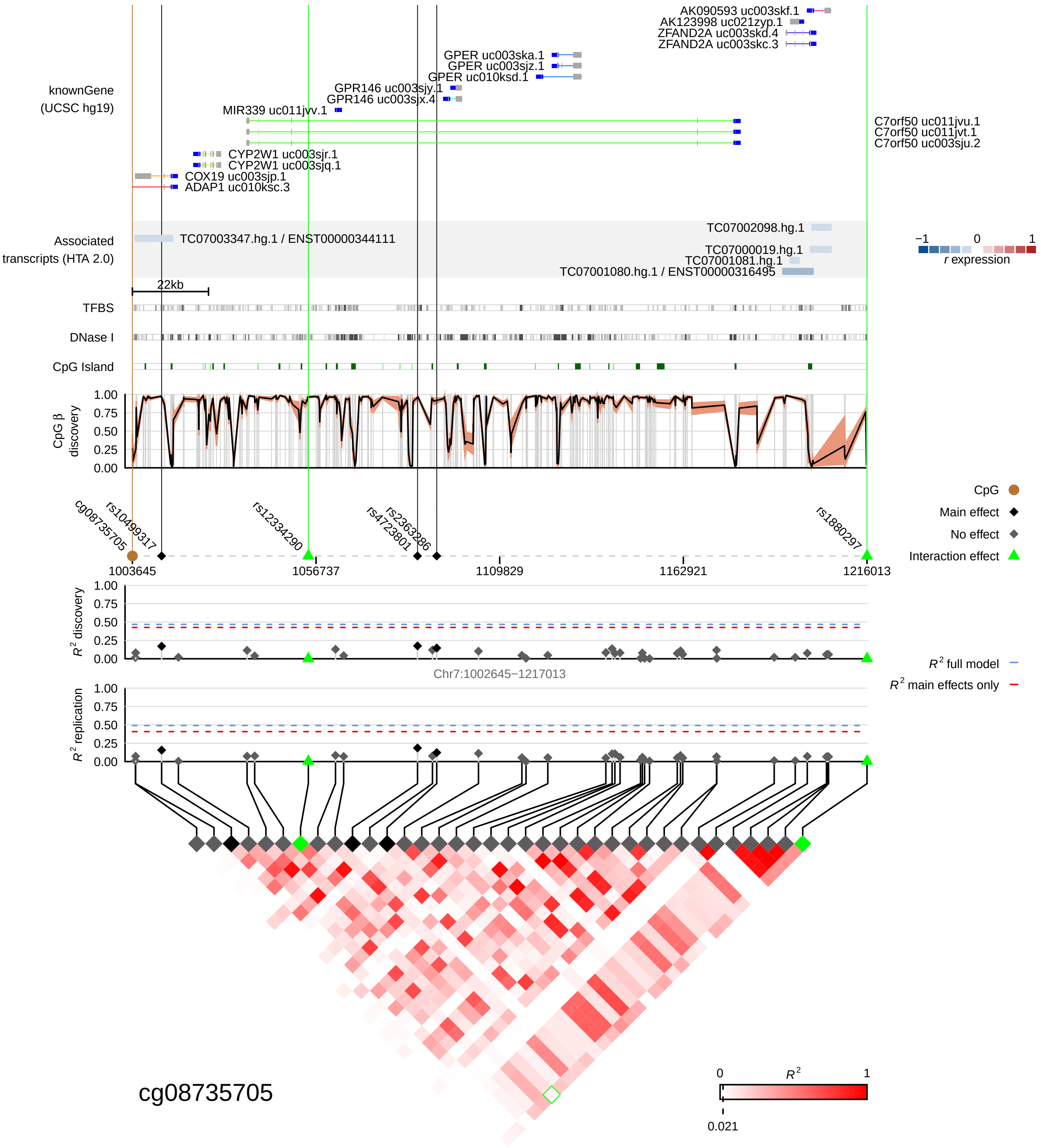
knownGene
(UCSC hg19)
AK090593 uc003skf.1
AK123998 uc021zyp.1
ZFAND2A uc003skd.4
ZFAND2A uc003skc.3
GPER uc003ska.1
GPER uc003sjz.1
GPER uc010ksd.1
GPR146 uc003sjy.1
GPR146 uc003sjx.4
MIR339 uc011jvv.1
C7orf50 uc011jvu.1
C7orf50 uc011jvt.1
C7orf50 uc003sju.2
CYP2W1 uc003sjr.1
CYP2W1 uc003sjq.1
COX19 uc003sjp.1
ADAP1 uc010ksc.3
Associated
transcripts (HTA 2.0)
TC07003347.hg.1 / ENST00000344111
TC07002098.hg.1
TC07000019.hg.1
TC07001081.hg.1
TC07001080.hg.1 / ENST00000316495
−1
0
1
r expression
22kb
TFBS
DNase I
CpG Island
CpG β
discovery
1.00
0.75
0.50
0.25
0.00
cg08735705
rs10499317
rs12334290
rs4723801
rs2363286
rs1880297
CpG
Main effect
No effect
Interaction effect
1003645
1056737
1109829
1162921
1216013
R2 discovery
Chr7:1002645−1217013
R2 full model
R2 main effects only
R2 replication
0
R2
1
0.021
cg08735705

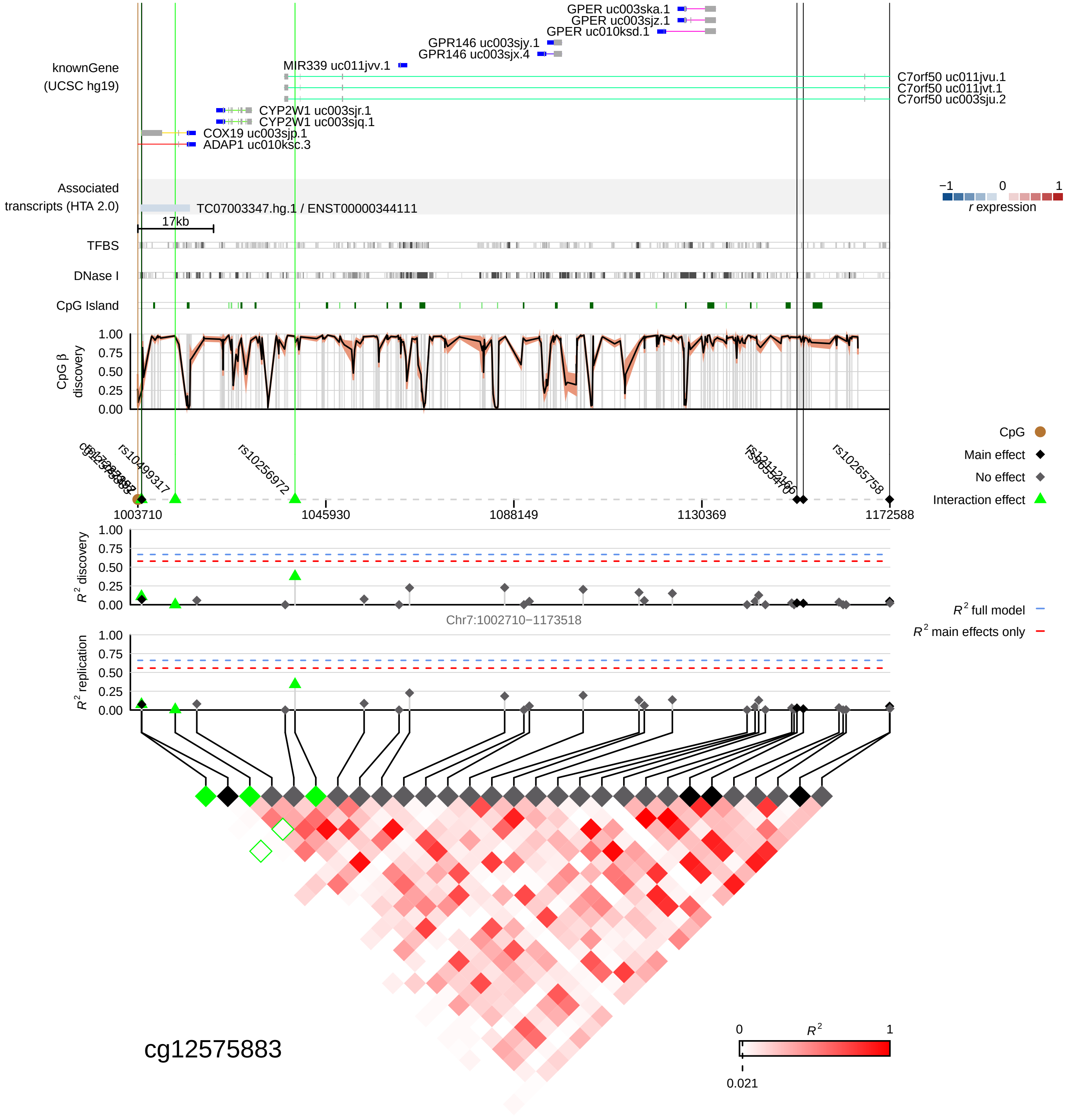

knownGene (UCSC hg19)
GPER uc003ska.1
GPER uc003sjz.1
GPER uc010ksd.1
GPR146 uc003sjy.1
GPR146 uc003sjx.4
MIR339 uc011jvv.1
C7orf50 uc011jvu.1
C7orf50 uc011jvt.1
C7orf50 uc003sju.2
CYP2W1 uc003sjr.1
CYP2W1 uc003sjq.1
COX19 uc003sjp.1
ADAP1 uc010ksc.3
Associated transcripts (HTA 2.0)
TC07003347.hg.1 / ENST00000344111
17kb
−1 0 1
r expression
TFBS
DNase I
CpG Island
CpG β discovery
1.00 0.75 0.50 0.25 0.00
rs10499317
rs10256972
rs10265758
CpG
Main effect
No effect
Interaction effect
1003710
1045930
1088149
1130369
1172588
R^2 discovery
Chr7:1002710−1173518
R^2 full model
R^2 main effects only
R^2 replication
cg12575883
0
R^2
1
0.021

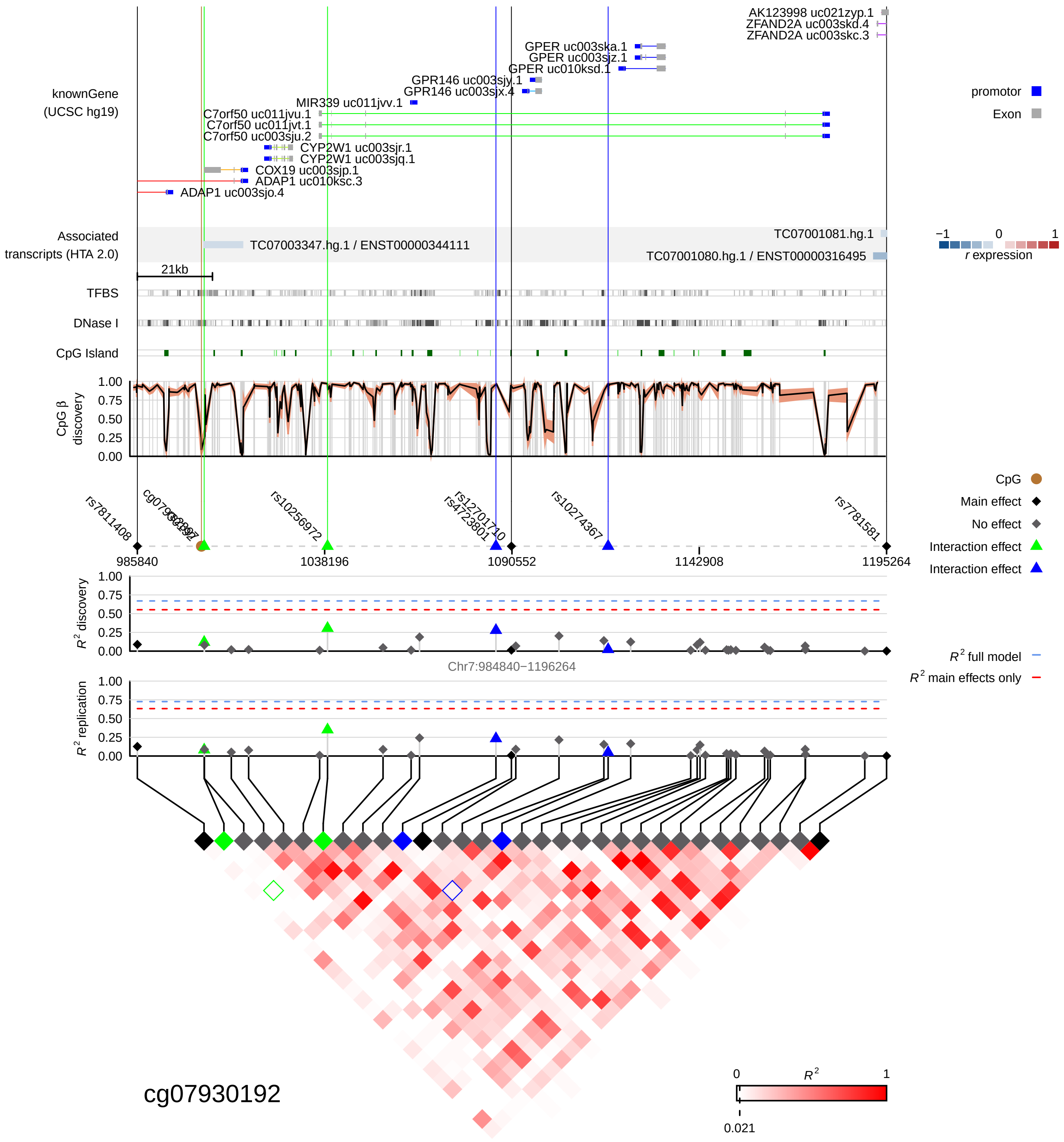

knownGene (UCSC hg19)
AK123998 uc021zyp.1
ZFAND2A uc003skd.4
ZFAND2A uc003skc.3
GPER uc003ska.1
GPER uc003sjz.1
GPER uc010ksd.1
GPR146 uc003sjy.1
GPR146 uc003sjx.4
MIR339 uc011jvv.1
C7orf50 uc011jvu.1
C7orf50 uc011jvt.1
C7orf50 uc003sju.2
CYP2W1 uc003sjr.1
CYP2W1 uc003sjq.1
COX19 uc003sjp.1
ADAP1 uc010ksc.3
ADAP1 uc003sjo.4
promotor
Exon
Associated transcripts (HTA 2.0)
TC07001081.hg.1
TC07003347.hg.1 / ENST00000344111
TC07001080.hg.1 / ENST00000316495
−1 0 1
r expression
21kb
TFBS
DNase I
CpG Island
CpG β discovery
1.00 0.75 0.50 0.25 0.00
rs7811408
cg07930192
rs10256972
rs12701740
rs4723801
rs10274367
rs7781581
985840
1038196
1090552
1142908
1195264
CpG
Main effect
No effect
Interaction effect
Interaction effect
R2 discovery
Chr7:984840−1196264
R2 replication
R2 full model
R2 main effects only
0 R2 1
0.021
cg07930192

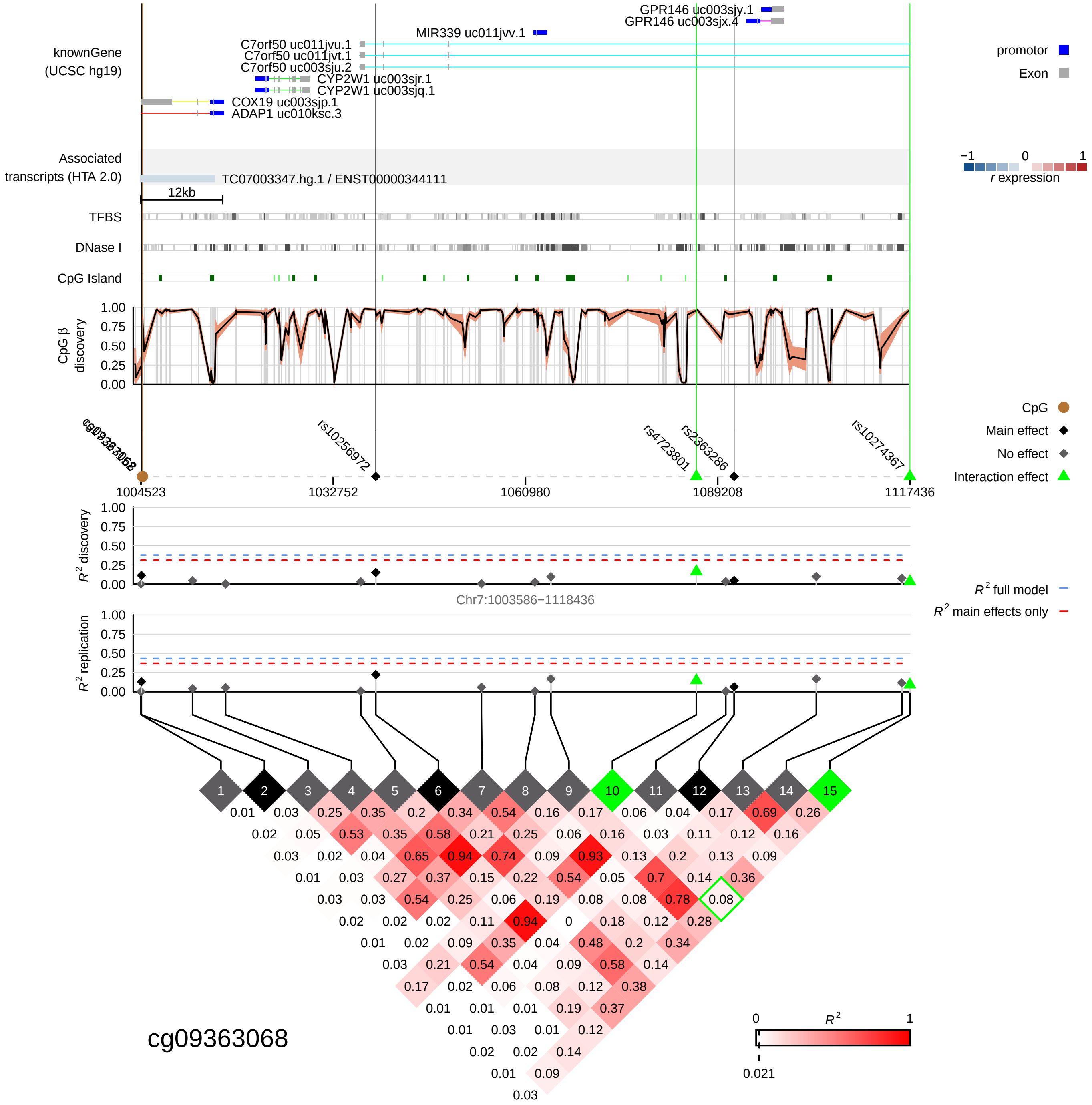

knownGene
(UCSC hg19)
GPR146 uc003sjy.1
GPR146 uc003sjx.4
MIR339 uc011jvv.1
C7orf50 uc011jvu.1
C7orf50 uc011jvt.1
C7orf50 uc003sju.2
CYP2W1 uc003sjr.1
CYP2W1 uc003sjq.1
COX19 uc003sjp.1
ADAP1 uc010ksc.3
promotor
Exon
Associated
transcripts (HTA 2.0)
TC07003347.hg.1 / ENST00000344111
−1 0 1
r expression
12kb
TFBS
DNase I
CpG Island
CpG β discovery
1.00
0.75
0.50
0.25
0.00
rs10256972
rs4723801
rs2363286
rs10274367
CpG
Main effect
No effect
Interaction effect
1004523
1032752
1060980
1089208
1117436
R^2 discovery
Chr7:1003586−1118436
R^2 full model
R^2 main effects only
R^2 replication
1 2 3 4 5 6 7 8 9 10 11 12 13 14 15
0.01 0.03 0.25 0.35 0.2 0.34 0.54 0.16 0.17 0.06 0.04 0.17 0.69 0.26
0.02 0.05 0.53 0.35 0.58 0.21 0.25 0.06 0.16 0.03 0.11 0.12 0.16
0.03 0.02 0.04 0.65 0.94 0.74 0.09 0.93 0.13 0.2 0.13 0.09
0.01 0.03 0.27 0.37 0.15 0.22 0.54 0.05 0.7 0.14 0.36
0.03 0.03 0.54 0.25 0.06 0.19 0.08 0.08 0.78 0.08
0.02 0.02 0.02 0.11 0.94 0 0.18 0.12 0.28
0.01 0.02 0.09 0.35 0.04 0.48 0.2 0.34
0.03 0.21 0.54 0.04 0.09 0.58 0.14
0.17 0.02 0.06 0.08 0.12 0.38
0.01 0.01 0.01 0.19 0.37
0.01 0.03 0.01 0.12
0.02 0.02 0.14
0.01 0.09
0.03
cg09363068
0 R^2 1
0.021

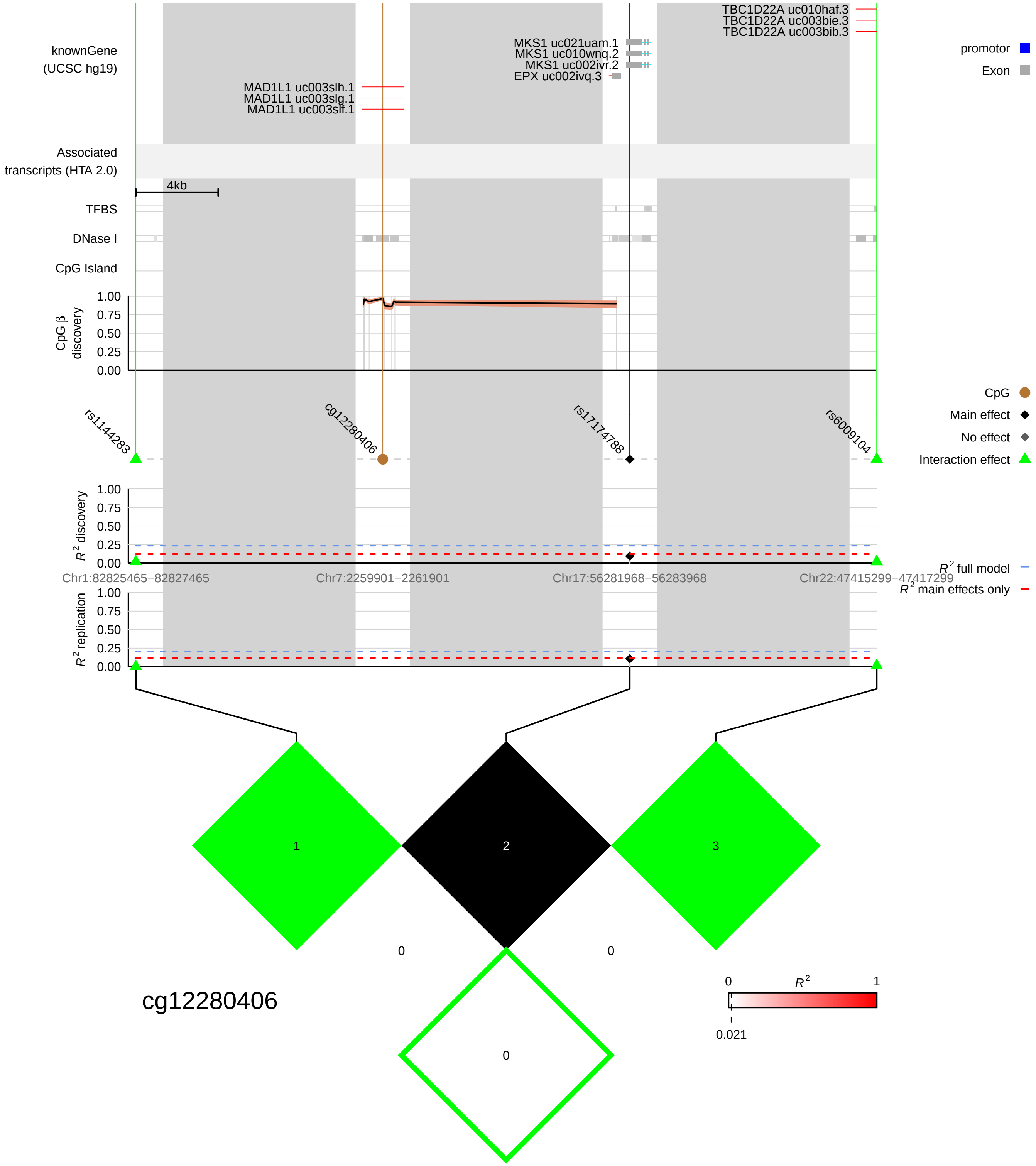

knownGene
(UCSC hg19)
TBC1D22A uc010haf.3
TBC1D22A uc003bie.3
TBC1D22A uc003bib.3
MKS1 uc021uam.1
MKS1 uc010wnq.2
MKS1 uc002ivr.2
EPX uc002ivq.3
MAD1L1 uc003slh.1
MAD1L1 uc003slg.1
MAD1L1 uc003slf.1
promotor
Exon
Associated
transcripts (HTA 2.0)
4kb
TFBS
DNase I
CpG Island
CpG β discovery
1.00
0.75
0.50
0.25
0.00
rs1144283
cg12280406
rs17174788
rs6009104
CpG
Main effect
No effect
Interaction effect
R² discovery
R² full model
R² main effects only
Chr1:82825465−82827465
Chr7:2259901−2261901
Chr17:56281968−56283968
Chr22:47415299−47417299
R² replication
1
2
3
0
0
0
cg12280406
0
R²
1
0.021

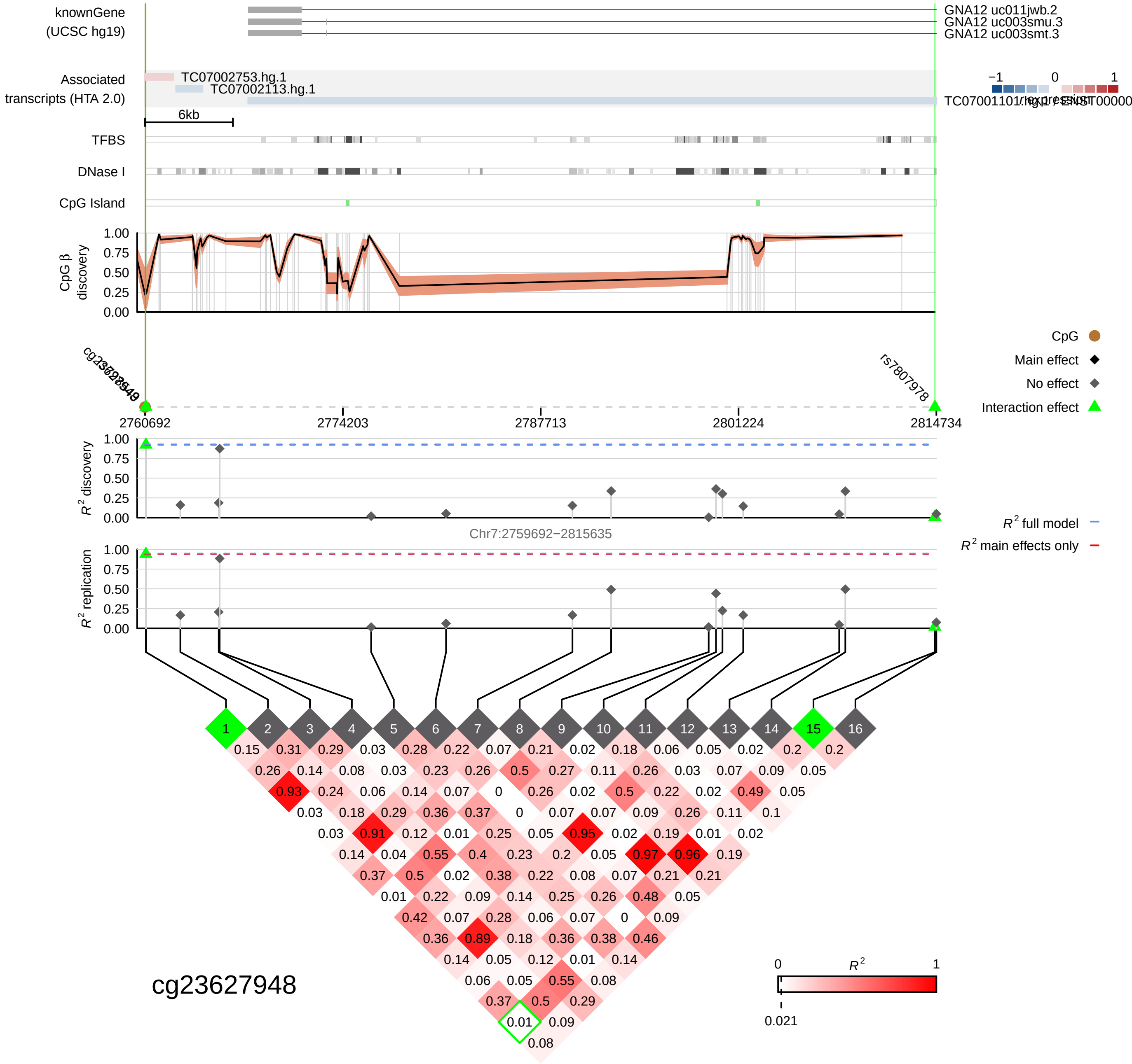
knownGene (UCSC hg19)
GNA12 uc011jwb.2
GNA12 uc003smu.3
GNA12 uc003smt.3
Associated transcripts (HTA 2.0)
TC07002753.hg.1
TC07002113.hg.1
6kb
TFBS
DNase I
CpG Island
CpG β discovery
CpG
Main effect
No effect
Interaction effect
rs7807978
2760692
2774203
2787713
2801224
2814734
R^2 discovery
Chr7:2759692−2815635
R^2 full model
R^2 main effects only
R^2 replication
R^2
0.021
cg23627948

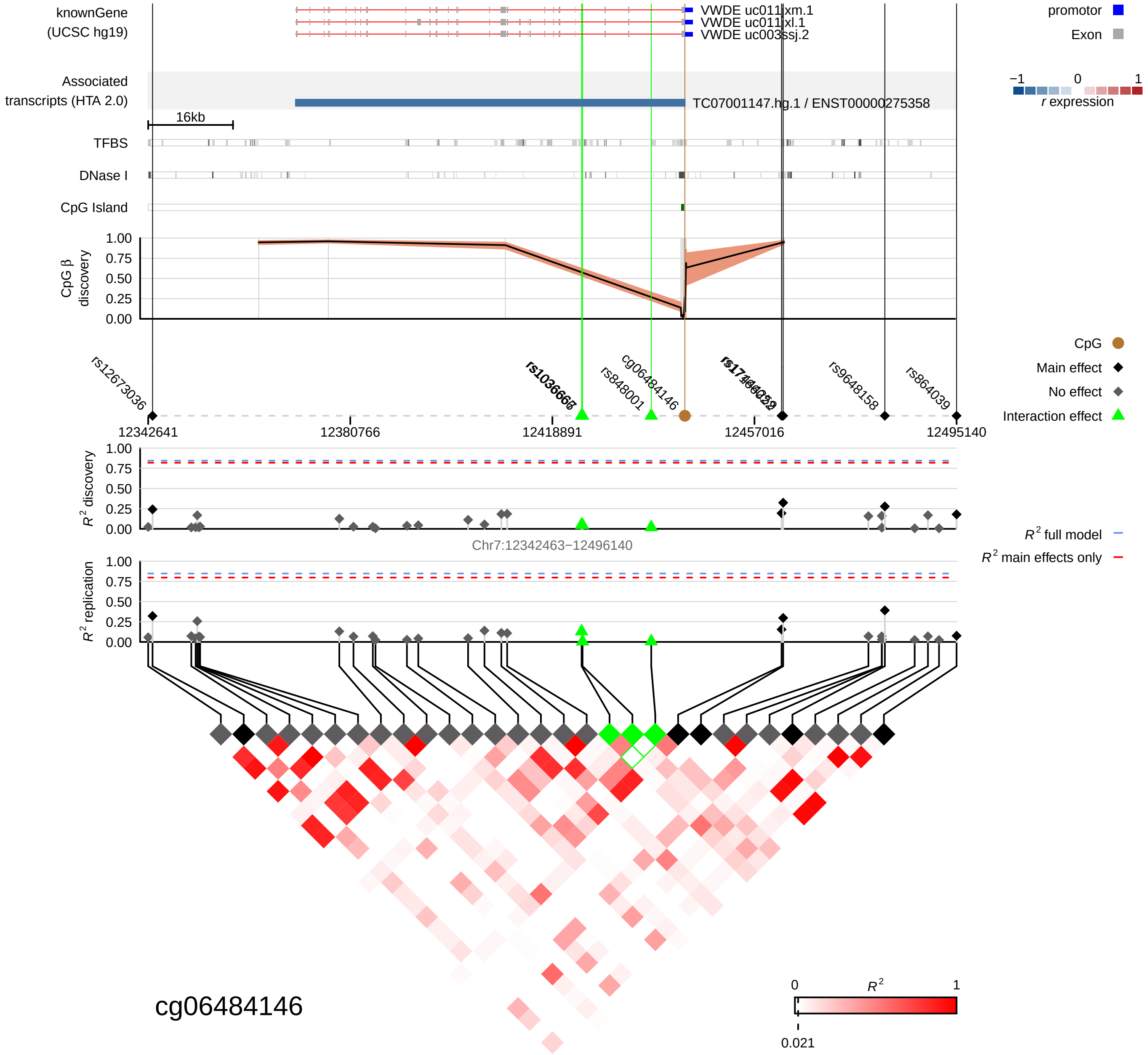
knownGene
(UCSC hg19)
VWDE uc011kxm.1
VWDE uc011kxl.1
VWDE uc003ssj.2
promotor
Exon
Associated
transcripts (HTA 2.0)
TC07001147.hg.1 / ENST00000275358
−1
0
1
r expression
16kb
TFBS
DNase I
CpG Island
CpG β
discovery
1.00
0.75
0.50
0.25
0.00
rs12673036
rs848001
cg06484146
rs9648158
rs864039
CpG
Main effect
No effect
Interaction effect
12342641
12380766
12418891
12457016
12495140
R² discovery
Chr7:12342463−12496140
R² full model
R² main effects only
R² replication
0
R²
1
0.021
cg06484146

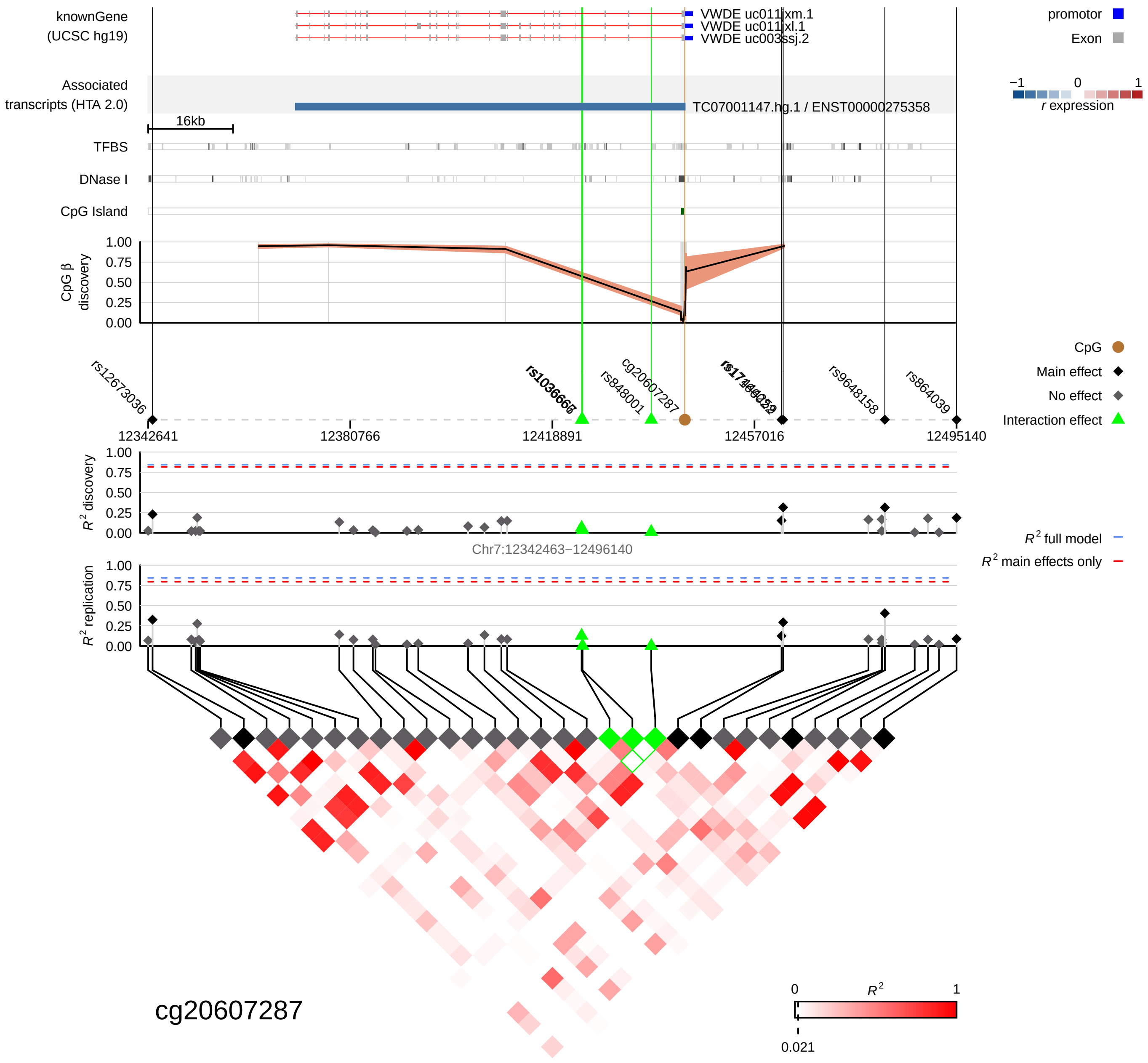

knownGene
(UCSC hg19)
VWDE uc011xm.1
VWDE uc011xl.1
VWDE uc003ssj.2
promotor
Exon
Associated
transcripts (HTA 2.0)
TC07001147.hg.1 / ENST00000275358
−1
0
1
r expression
16kb
TFBS
DNase I
CpG Island
CpG β discovery
1.00
0.75
0.50
0.25
0.00
rs12673036
rs848001
cg20607287
rs9648158
rs864039
12342641
12380766
12418891
12457016
12495140
CpG
Main effect
No effect
Interaction effect
R² discovery
Chr7:12342463−12496140
R² full model
R² main effects only
R² replication
0
R²
1
0.021
cg20607287

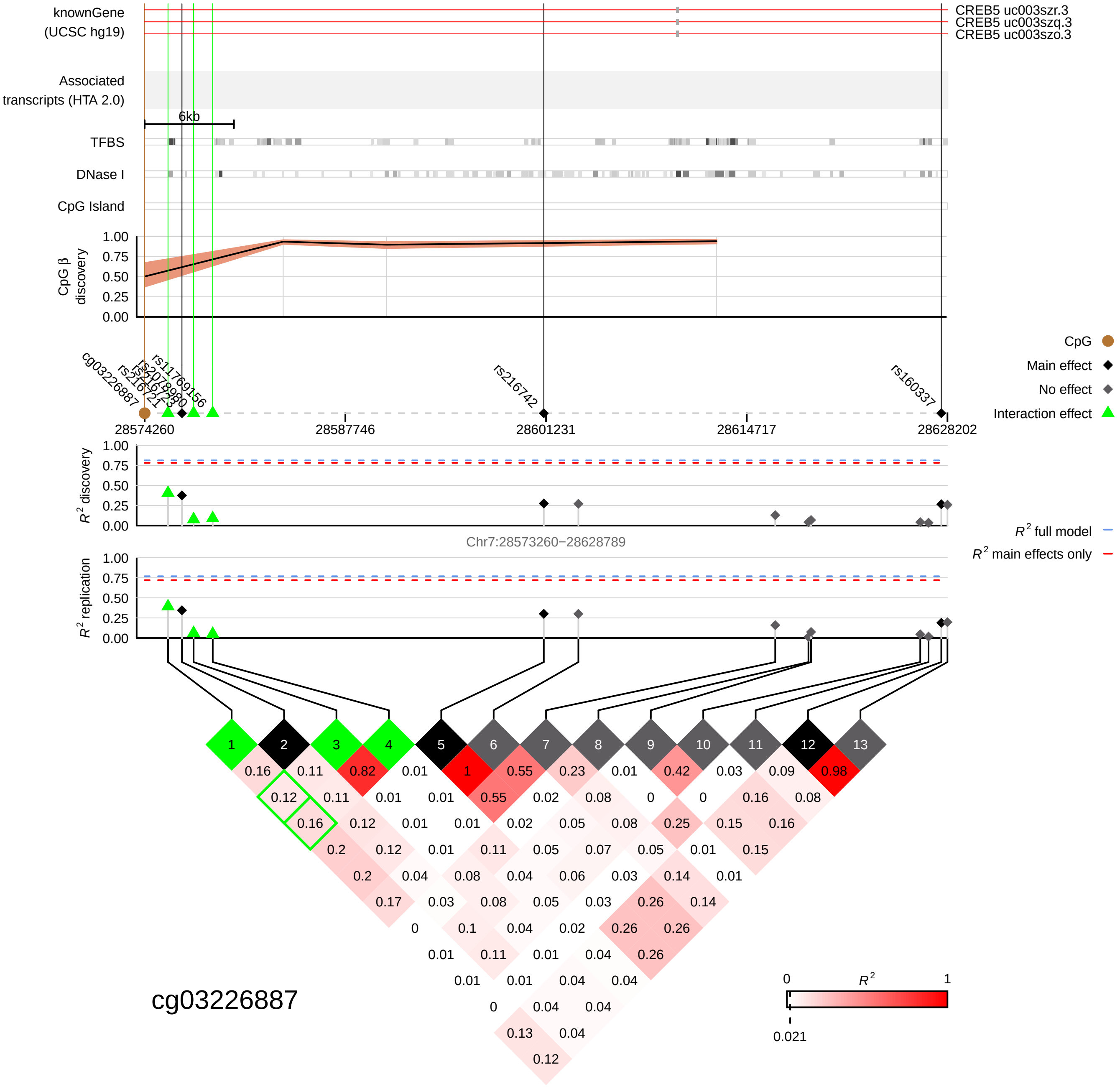

knownGene (UCSC hg19)
CREB5 uc003szr.3
CREB5 uc003szq.3
CREB5 uc003szo.3
Associated transcripts (HTA 2.0)
6kb
TFBS
DNase I
CpG Island
CpG β discovery
cg03226887
rs11769156
rs216742
rs160337
28574260
28587746
28601231
28614717
28628202
CpG
Main effect
No effect
Interaction effect
R^2 discovery
Chr7:28573260−28628789
R^2 full model
R^2 main effects only
R^2 replication
R^2
0.021
cg03226887

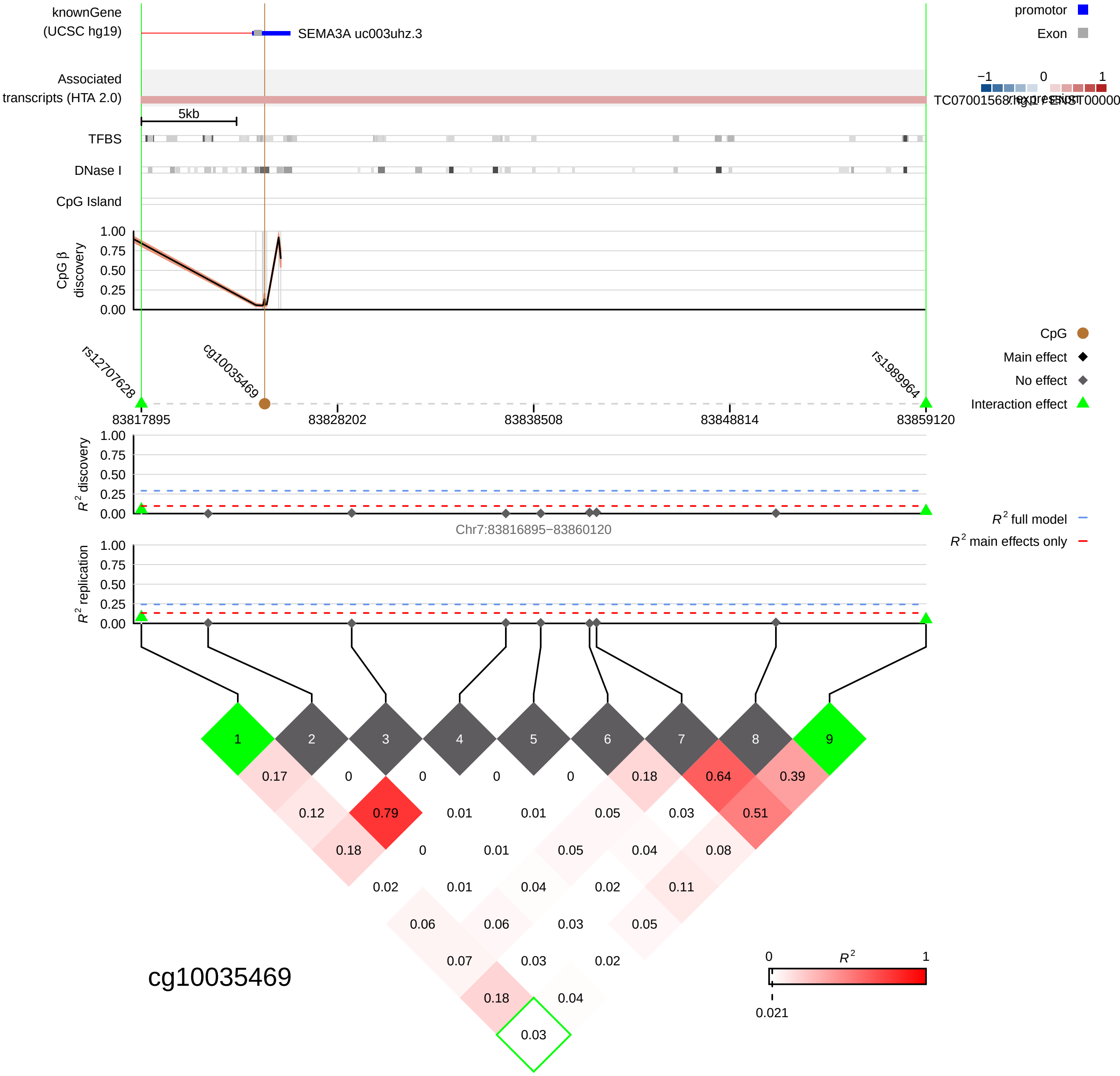
knownGene (UCSC hg19)
SEMA3A uc003uhz.3
promotor
Exon
Associated transcripts (HTA 2.0)
5kb
TFBS
DNase I
CpG Island
CpG β discovery
rs12707628
cg10035469
rs1989964
CpG
Main effect
No effect
Interaction effect
83817895
83828202
83838508
83848814
83859120
R^2 discovery
R^2 replication
Chr7:83816895−83860120
R^2 full model
R^2 main effects only
cg10035469
0.021

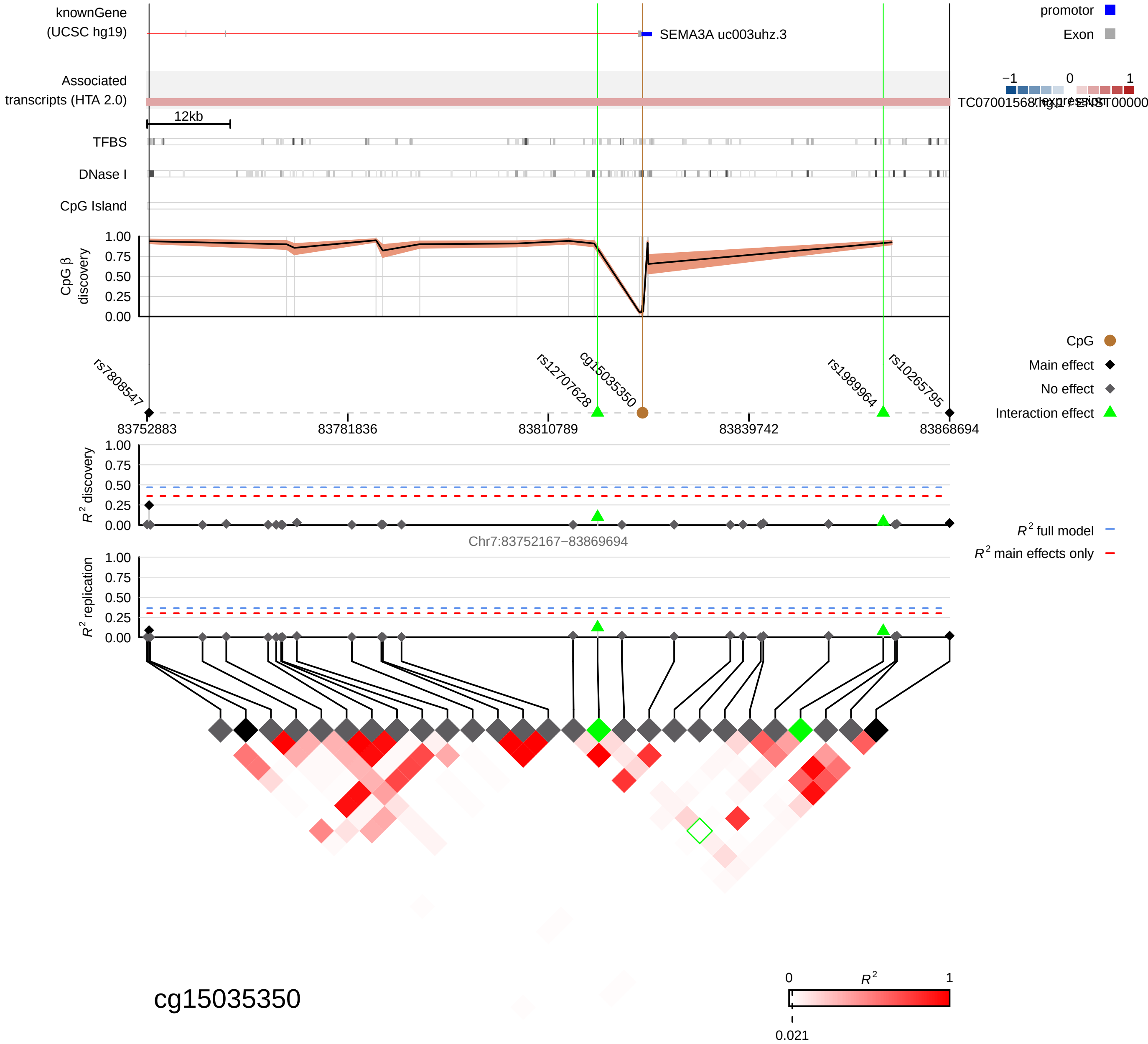

knownGene (UCSC hg19)
SEMA3A uc003uhz.3
promotor
Exon
Associated transcripts (HTA 2.0)
TC07001568.hg.1 / ENST00000
expression
−1
0
1
12kb
TFBS
DNase I
CpG Island
CpG β discovery
1.00
0.75
0.50
0.25
0.00
rs7808547
rs12707628
cg15035350
rs1989964
rs10265795
CpG
Main effect
No effect
Interaction effect
83752883
83781836
83810789
83839742
83868694
R^2 discovery
Chr7:83752167−83869694
R^2 full model
R^2 main effects only
R^2 replication
cg15035350
0
R^2
1
0.021

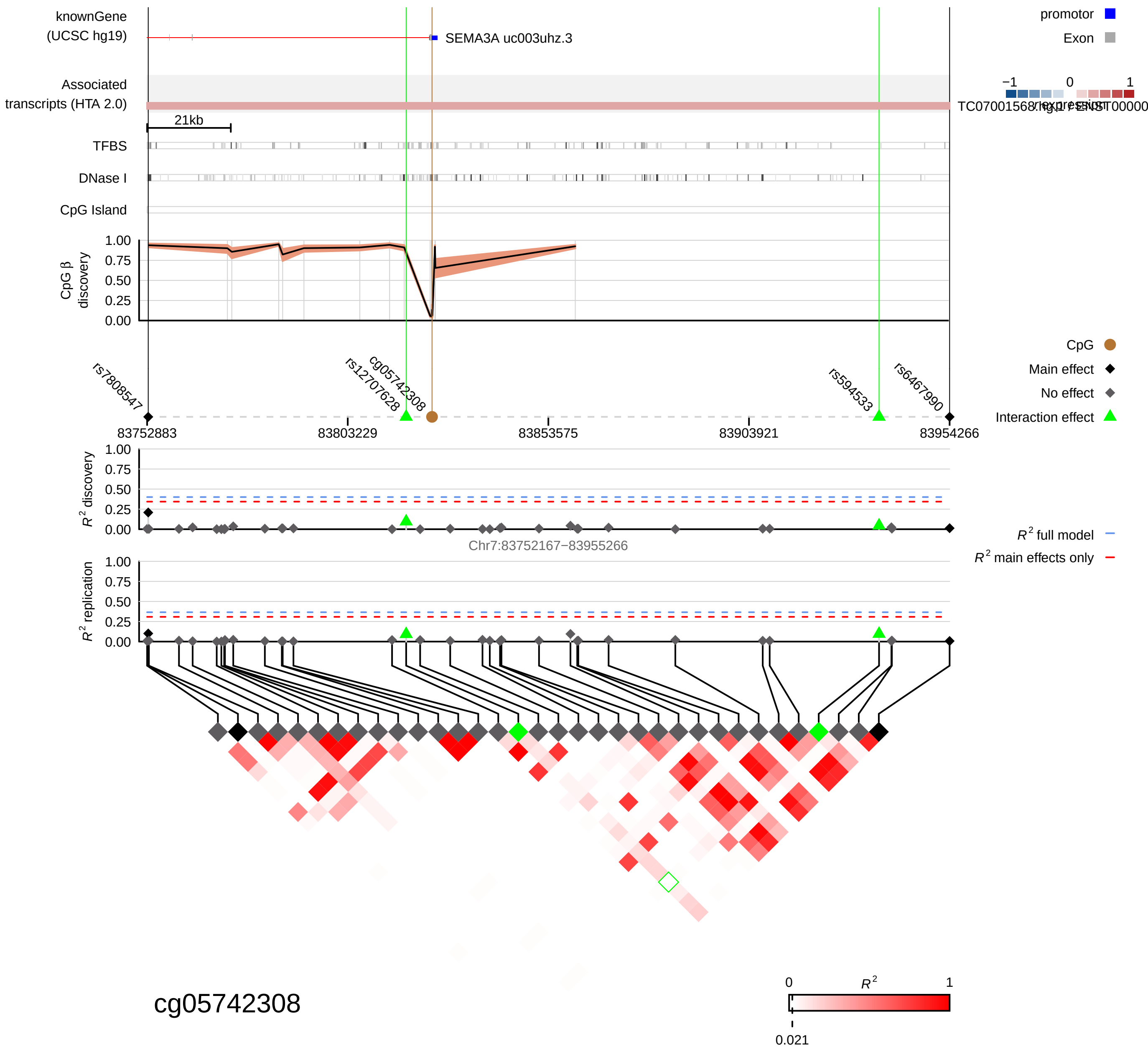

knownGene
(UCSC hg19)
SEMA3A uc003uhz.3
promotor
Exon
Associated
transcripts (HTA 2.0)
TC07001568.hg.1 / ENST00000
expression
−1
0
1
21kb
TFBS
DNase I
CpG Island
CpG β
discovery
1.00
0.75
0.50
0.25
0.00
rs7808547
rs12707628
cg05742308
rs594533
rs6467990
CpG
Main effect
No effect
Interaction effect
83752883
83803229
83853575
83903921
83954266
R^2 discovery
Chr7:83752167−83955266
R^2 full model
R^2 main effects only
R^2 replication
0
R^2
1
0.021
cg05742308

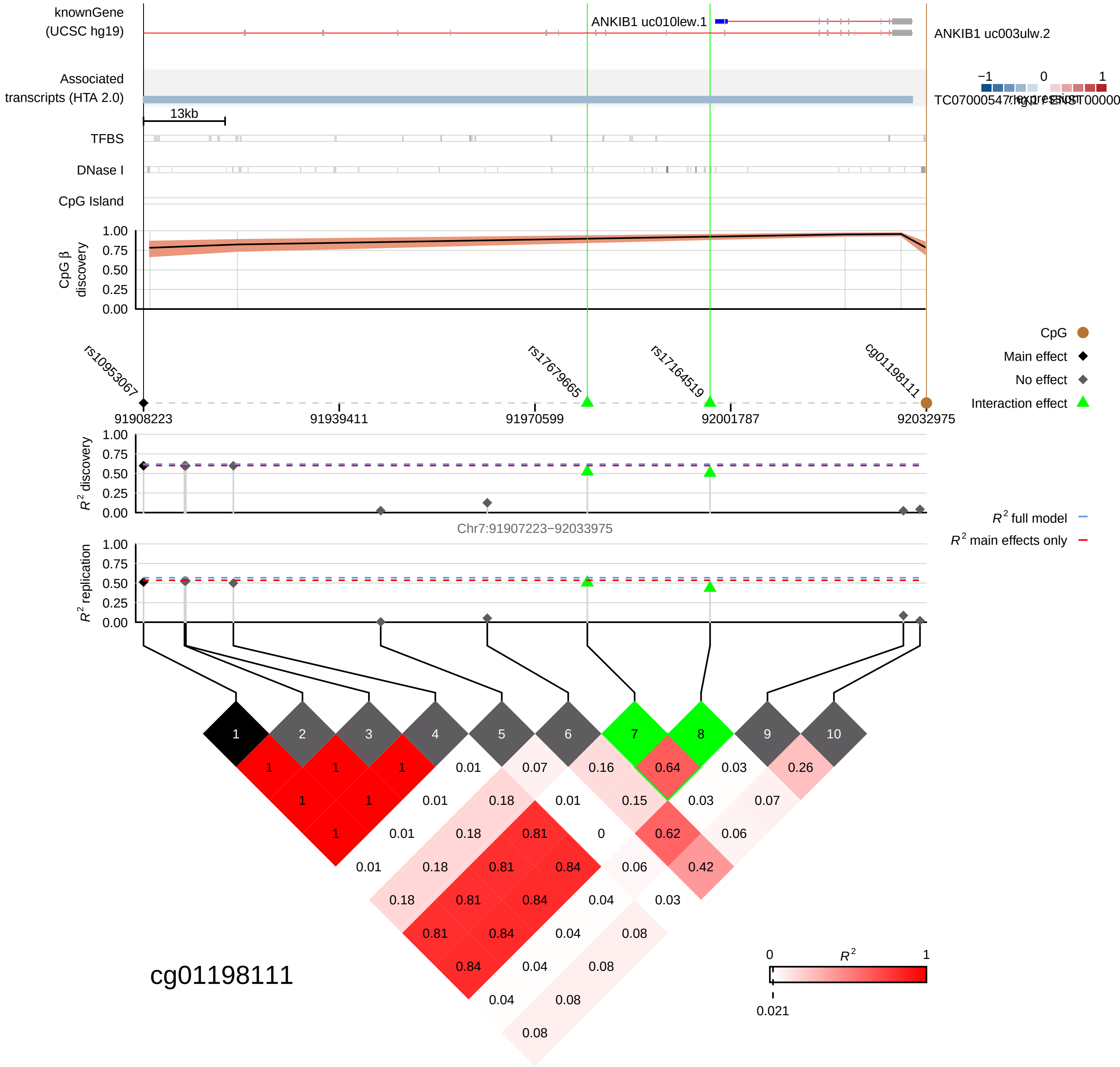
knownGene
(UCSC hg19)
ANKIB1 uc010lew.1
ANKIB1 uc003ulw.2
Associated
transcripts (HTA 2.0)
−1
0
1
13kb
TFBS
DNase I
CpG Island
CpG β
discovery
1.00
0.75
0.50
0.25
0.00
rs10953067
rs17679665
rs17164519
cg01198111
91908223
91939411
91970599
92001787
92032975
CpG
Main effect
No effect
Interaction effect
R² discovery
Chr7:91907223−92033975
R² full model
R² main effects only
R² replication
1
2
3
4
5
6
7
8
9
10
0
R²
1
0.021
cg01198111

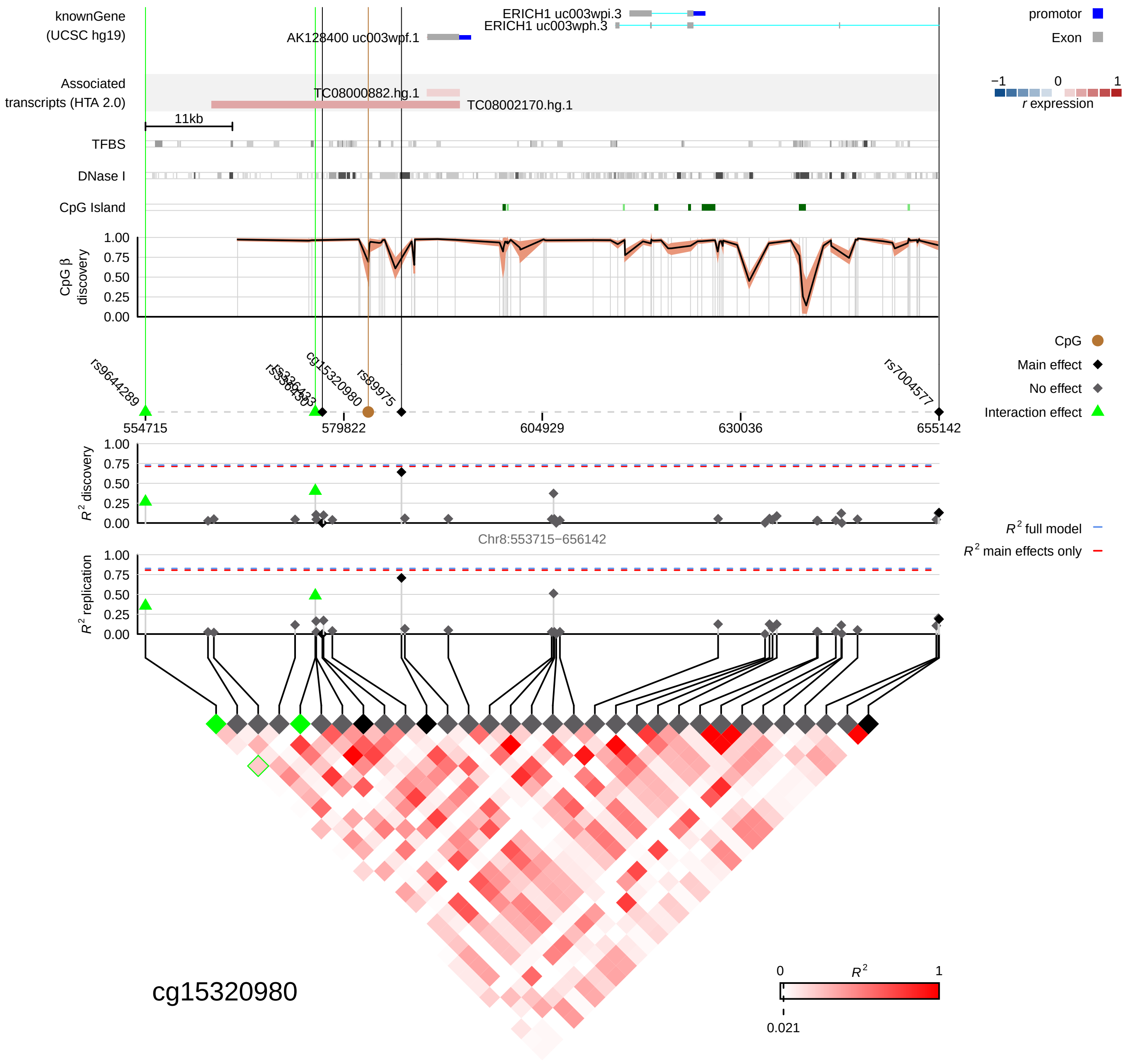

knownGene (UCSC hg19)
ERICH1 uc003wpi.3
ERICH1 uc003wph.3
AK128400 uc003wpf.1
promotor
Exon
Associated transcripts (HTA 2.0)
TC08000882.hg.1
TC08002170.hg.1
−1
0
1
r expression
11kb
TFBS
DNase I
CpG Island
CpG β discovery
1.00
0.75
0.50
0.25
0.00
rs9644289
cg15320980
rs89975
rs7004577
554715
579822
604929
630036
655142
CpG
Main effect
No effect
Interaction effect
R2 discovery
Chr8:553715−656142
R2 full model
R2 main effects only
R2 replication
cg15320980
0
R2
1
0.021

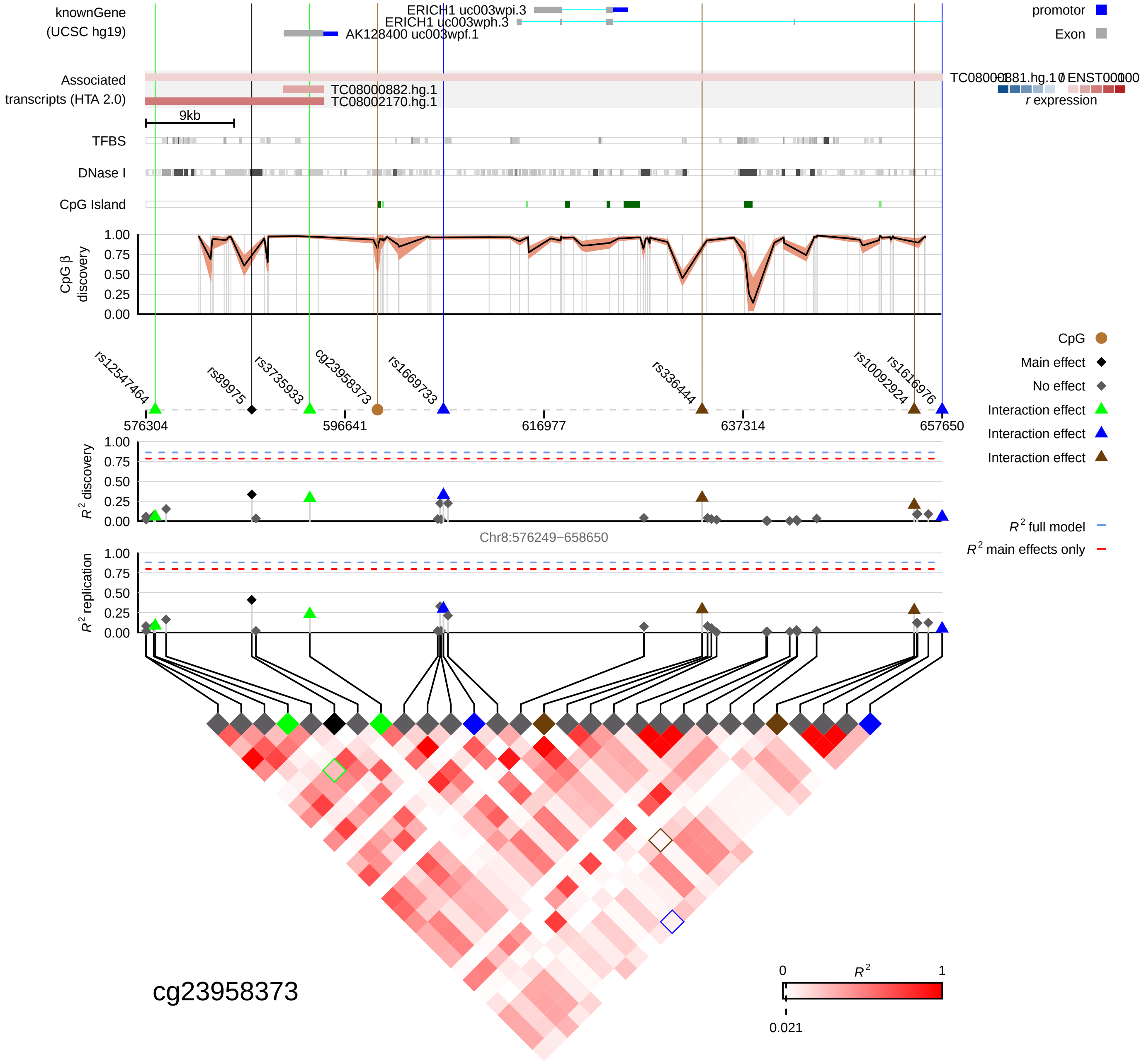
knownGene
(UCSC hg19)
ERICH1 uc003wpi.3
ERICH1 uc003wph.3
AK128400 uc003wpf.1
promotor
Exon
Associated
transcripts (HTA 2.0)
TC08000882.hg.1
TC08002170.hg.1
r expression
9kb
TFBS
DNase I
CpG Island
CpG β
discovery
rs12547464
rs89975
rs3735933
cg23958373
rs1669733
rs336444
rs10092924
rs1616976
576304
596641
616977
637314
657650
CpG
Main effect
No effect
Interaction effect
Interaction effect
Interaction effect
R^2 discovery
Chr8:576249−658650
R^2 full model
R^2 main effects only
R^2 replication
cg23958373
R^2
0
1
0.021

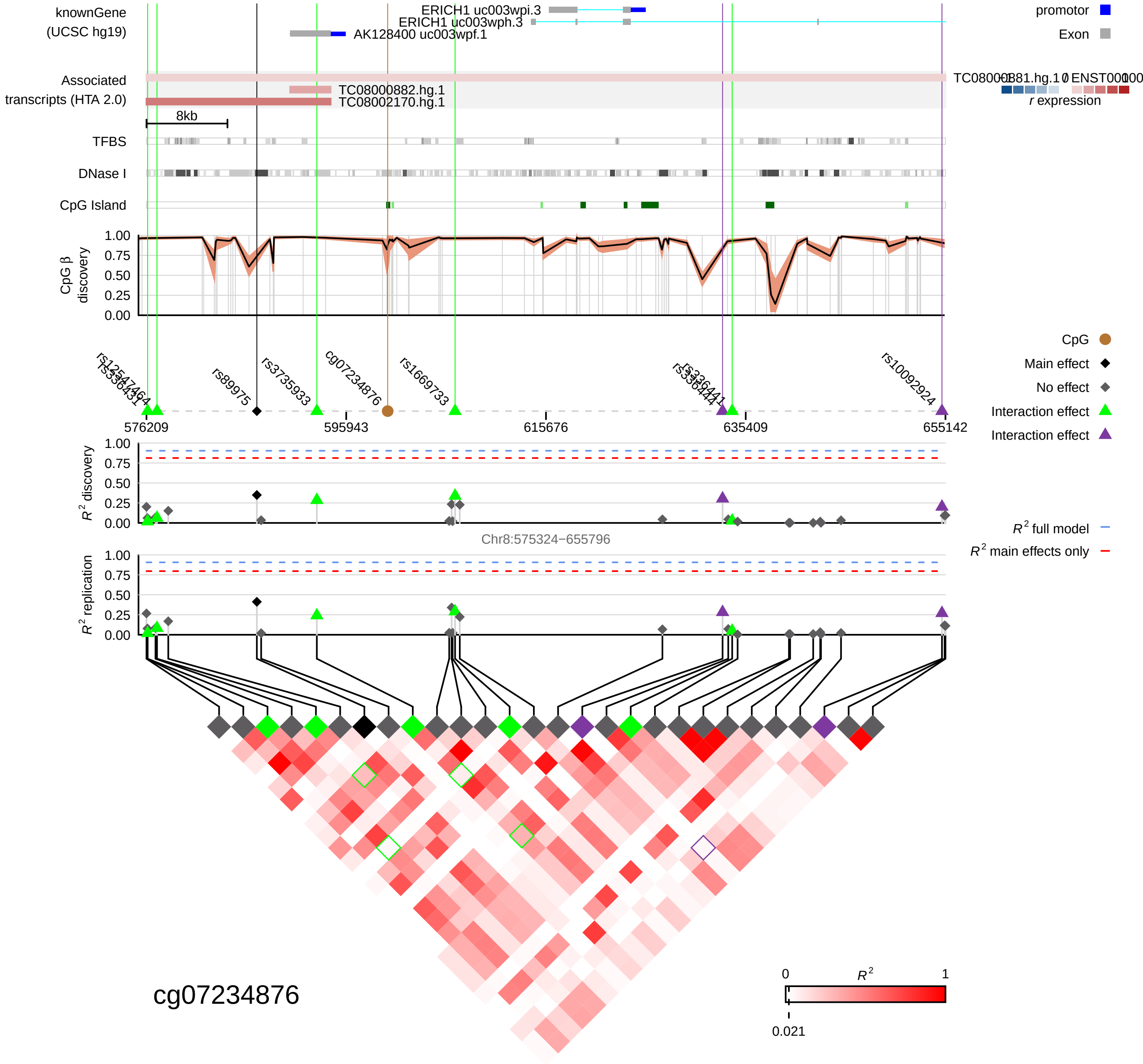
knownGene
(UCSC hg19)
ERICH1 uc003wpi.3
ERICH1 uc003wph.3
AK128400 uc003wpf.1
promotor
Exon
Associated
transcripts (HTA 2.0)
TC08000882.hg.1
TC08002170.hg.1
r expression
8kb
TFBS
DNase I
CpG Island
CpG β discovery
1.00
0.75
0.50
0.25
0.00
rs12547464
rs336431
rs89975
rs3735933
cg07234876
rs1669733
rs336441
rs336444
rs10092924
576209
595943
615676
635409
655142
CpG
Main effect
No effect
Interaction effect
Interaction effect
R² discovery
Chr8:575324−655796
R² replication
R² full model
R² main effects only
0
R²
1
0.021
cg07234876

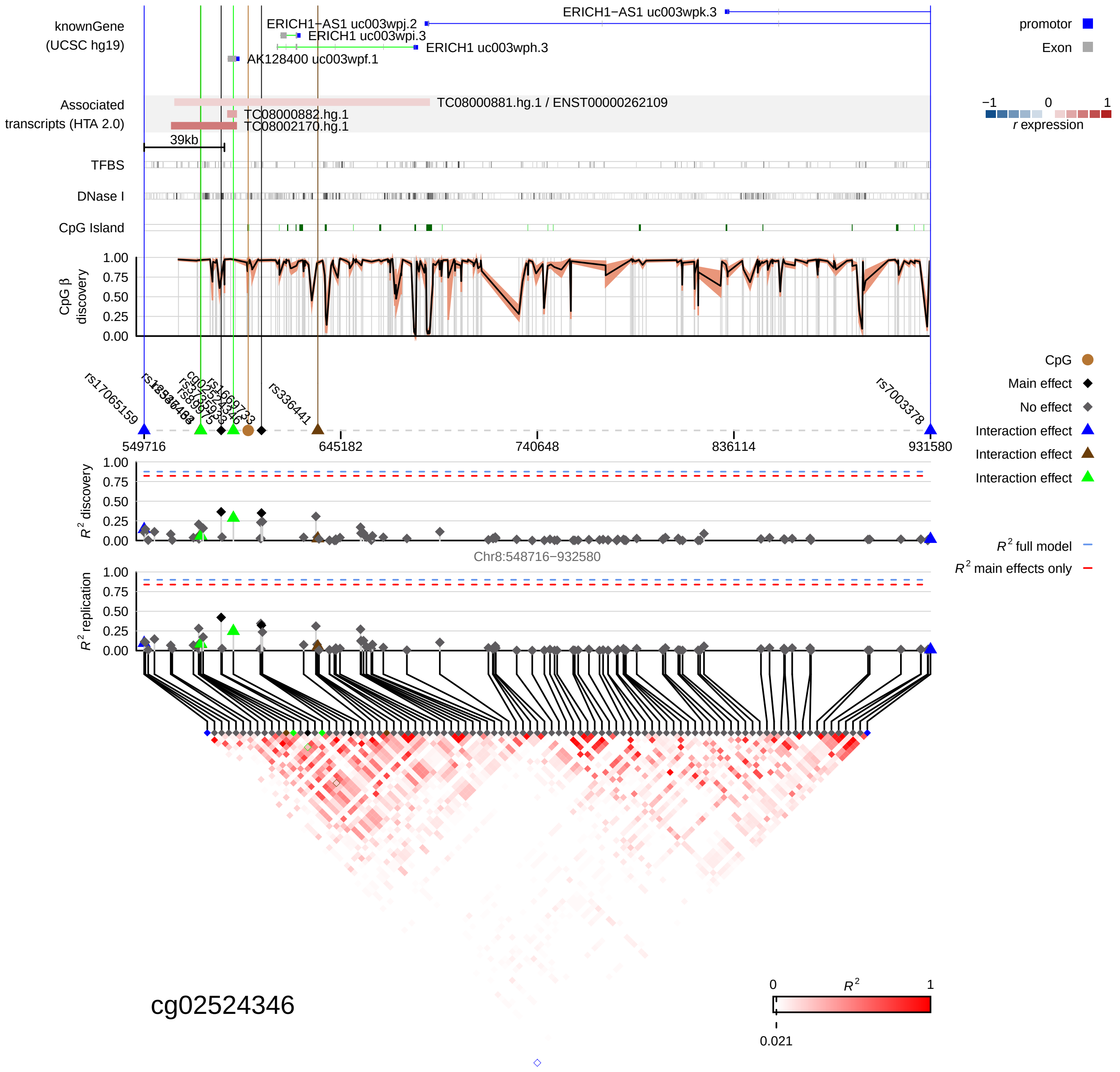
knownGene
(UCSC hg19)
ERICH1−AS1 uc003wpk.3
ERICH1−AS1 uc003wpj.2
ERICH1 uc003wpi.3
ERICH1 uc003wph.3
AK128400 uc003wpf.1
promotor
Exon
Associated
transcripts (HTA 2.0)
TC08000881.hg.1 / ENST00000262109
TC08000882.hg.1
TC08002170.hg.1
−1
0
1
r expression
39kb
TFBS
DNase I
CpG Island
CpG β
discovery
1.00
0.75
0.50
0.25
0.00
rs17065159
rs336441
rs7003378
549716
645182
740648
836114
931580
CpG
Main effect
No effect
Interaction effect
Interaction effect
Interaction effect
R² discovery
Chr8:548716−932580
R² full model
R² main effects only
R² replication
cg02524346
0
R²
1
0.021

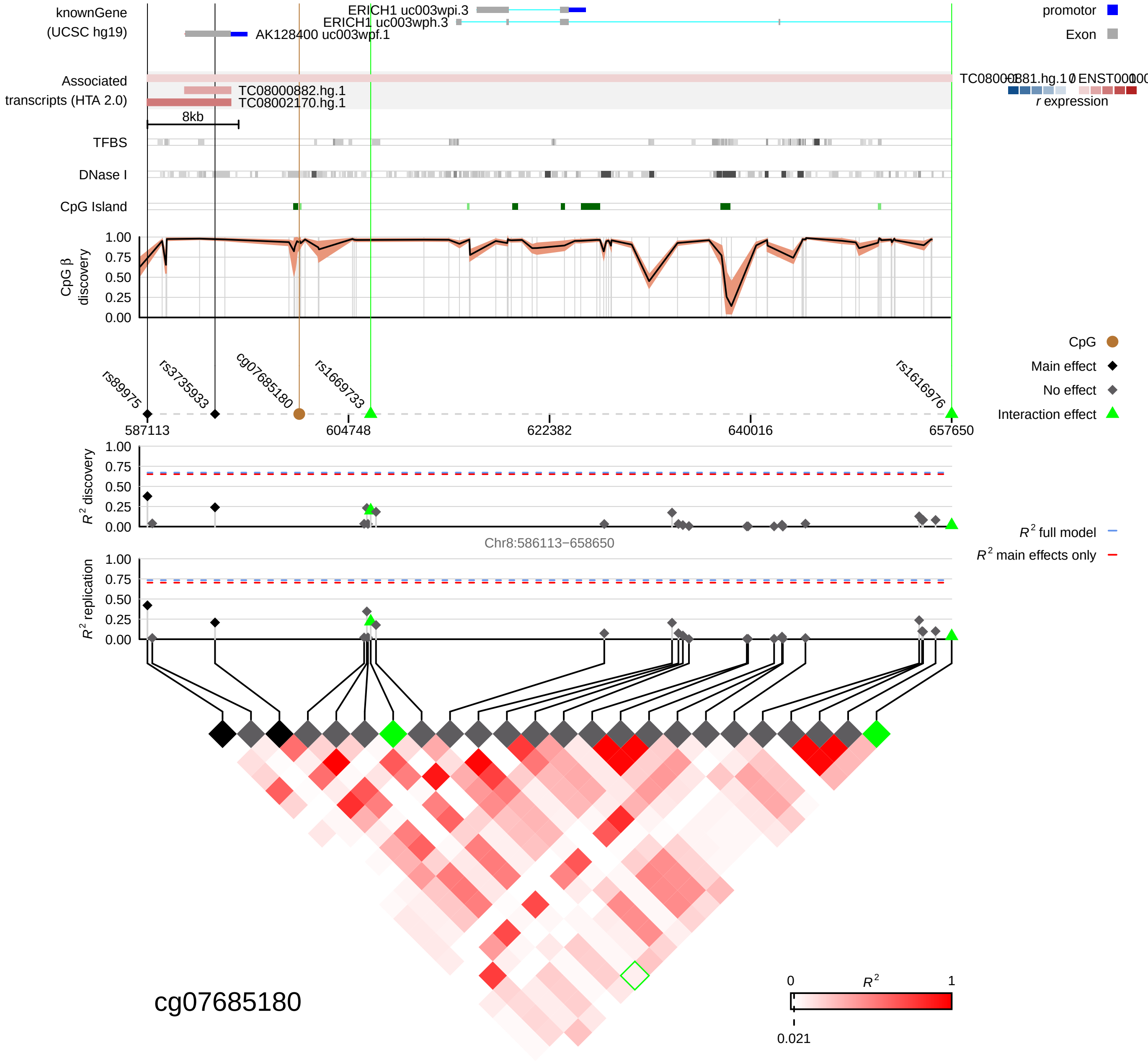
knownGene
(UCSC hg19)
ERICH1 uc003wpi.3
ERICH1 uc003wph.3
AK128400 uc003wpf.1
promotor
Exon
Associated
transcripts (HTA 2.0)
TC08000882.hg.1
TC08002170.hg.1
r expression
8kb
TFBS
DNase I
CpG Island
CpG β
discovery
1.00
0.75
0.50
0.25
0.00
rs899975
rs3735933
cg07685180
rs1669733
rs1616976
CpG
Main effect
No effect
Interaction effect
587113
604748
622382
640016
657650
R^2 discovery
Chr8:586113−658650
R^2 full model
R^2 main effects only
R^2 replication
cg07685180
0
R^2
1
0.021

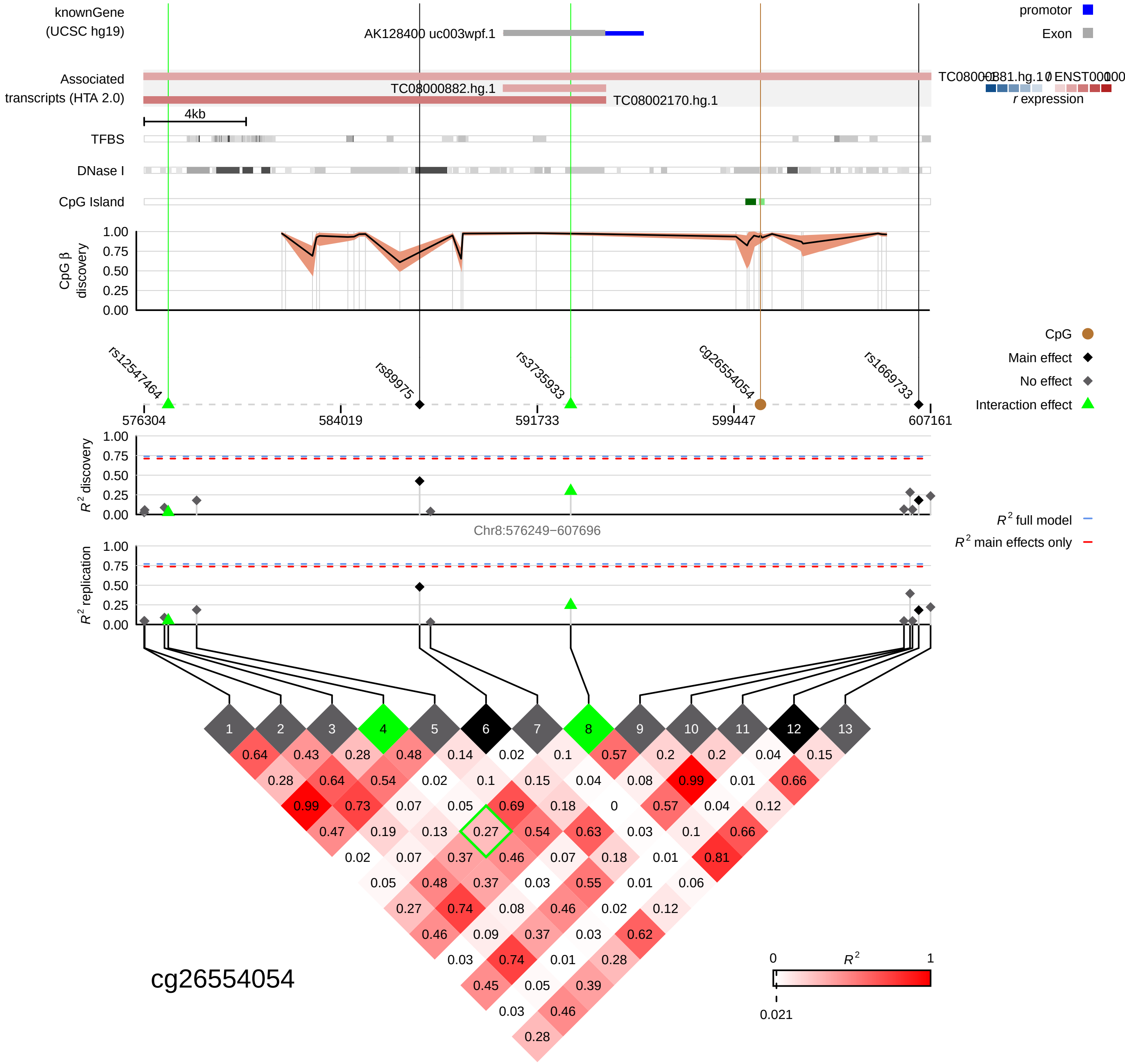

knownGene (UCSC hg19)
AK128400 uc003wpf.1
promotor
Exon
Associated transcripts (HTA 2.0)
TC08000882.hg.1
TC08002170.hg.1
r expression
4kb
TFBS
DNase I
CpG Island
CpG β discovery
rs12547464
rs89975
rs3735933
cg26554054
rs1669733
576304
584019
591733
599447
607161
CpG
Main effect
No effect
Interaction effect
R² discovery
Chr8:576249−607696
R² full model
R² main effects only
R² replication
cg26554054
0.021

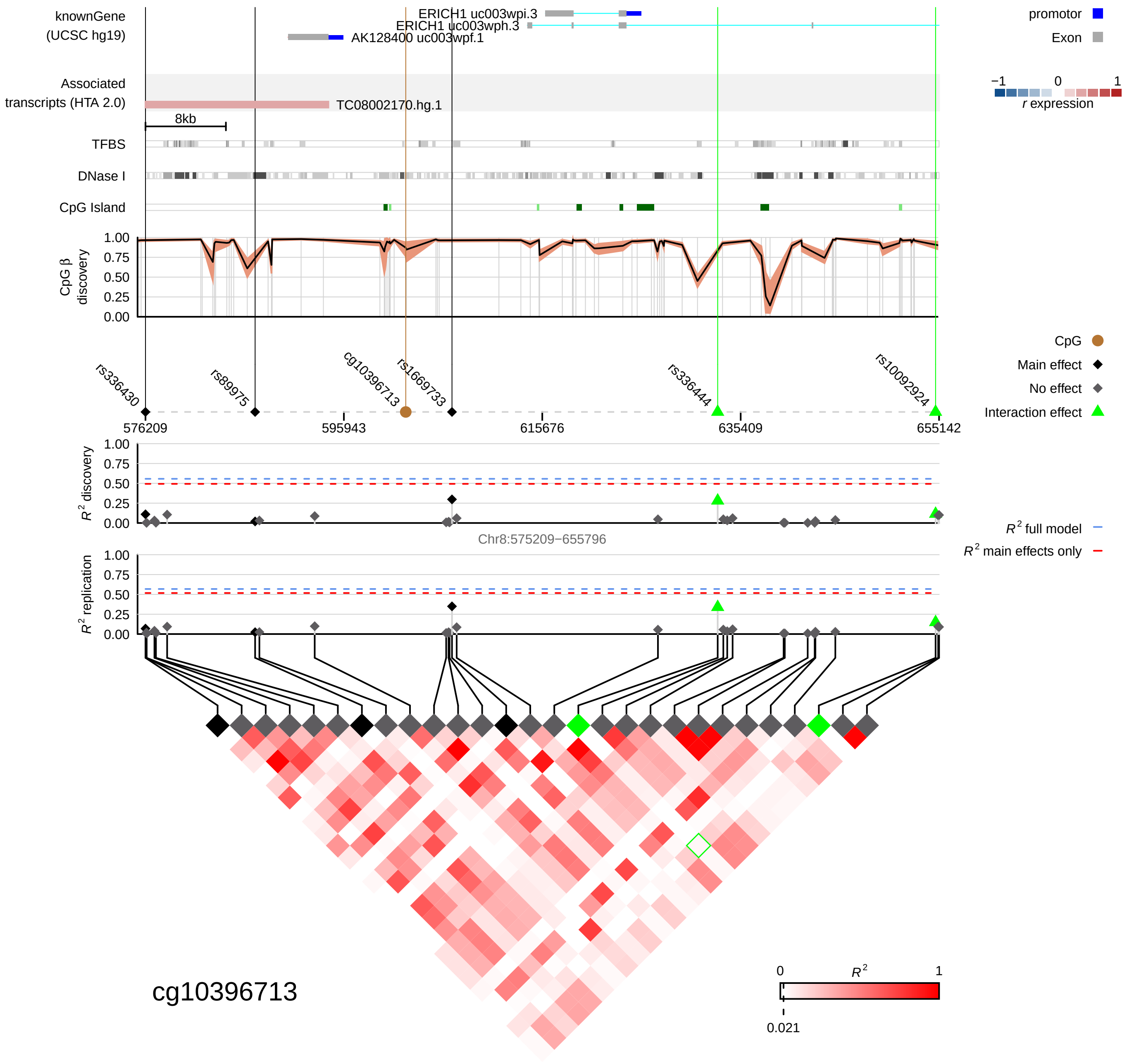

knownGene (UCSC hg19)
ERICH1 uc003wpi.3
ERICH1 uc003wph.3
AK128400 uc003wpf.1
promotor
Exon
Associated transcripts (HTA 2.0)
TC08002170.hg.1
−1 0 1
r expression
8kb
TFBS
DNase I
CpG Island
CpG β discovery
1.00
0.75
0.50
0.25
0.00
rs336430
rs89975
cg10396713
rs1669733
rs336444
rs10092924
576209
595943
615676
635409
655142
CpG
Main effect
No effect
Interaction effect
R² discovery
Chr8:575209−655796
R² full model
R² main effects only
R² replication
cg10396713
0 R² 1
0.021

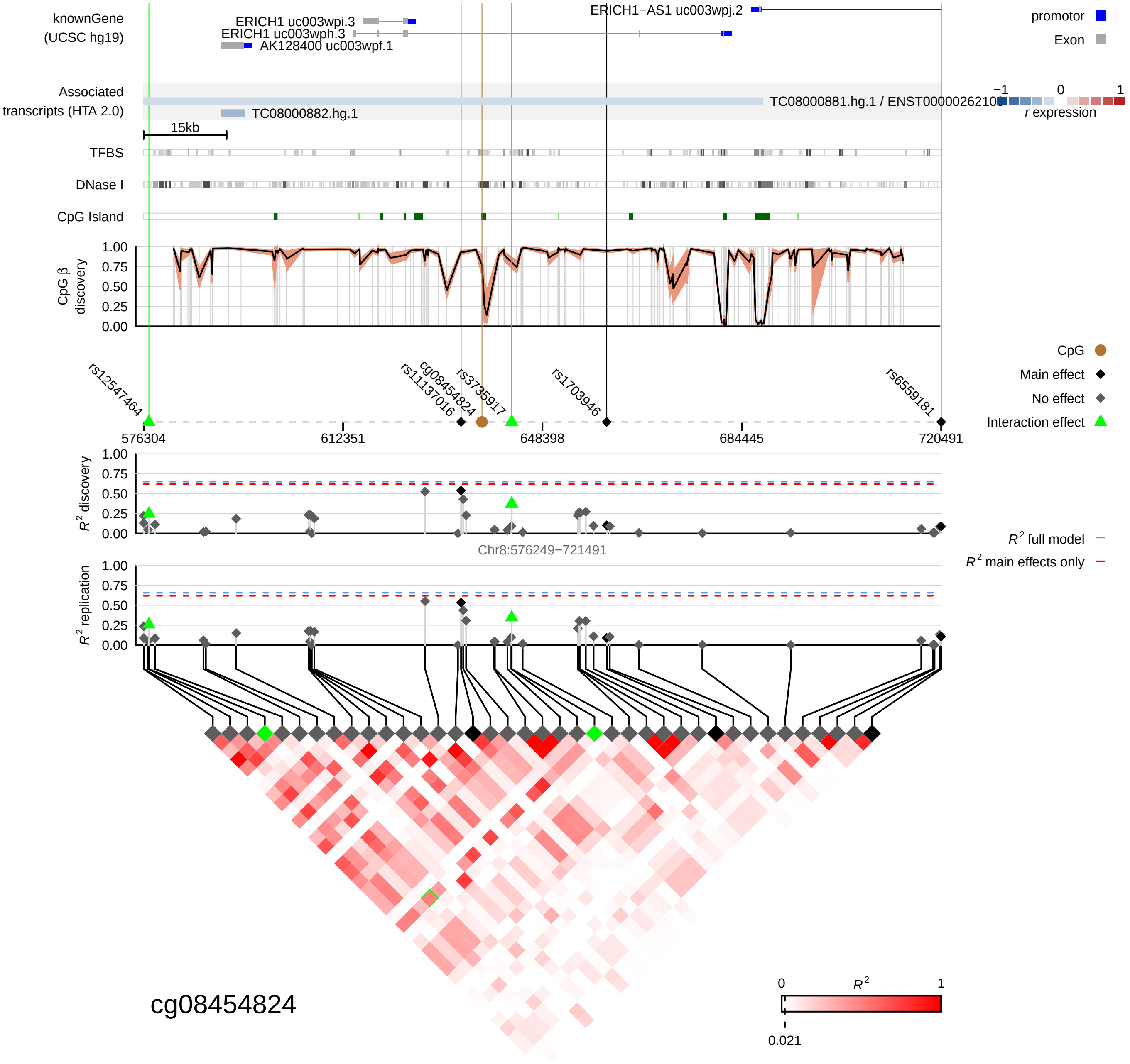

knownGene (UCSC hg19)
ERICH1 uc003wpi.3
ERICH1 uc003wph.3
AK128400 uc003wpf.1
ERICH1−AS1 uc003wpj.2
promotor
Exon
Associated transcripts (HTA 2.0)
TC08000881.hg.1 / ENST00000262109
TC08000882.hg.1
r expression
15kb
TFBS
DNase I
CpG Island
CpG β discovery
rs12547464
rs11137016
cg08454824
rs3735917
rs1703946
rs6559181
576304
612351
648398
684445
720491
CpG
Main effect
No effect
Interaction effect
R² discovery
Chr8:576249−721491
R² full model
R² main effects only
R² replication
0.021

cg08454824

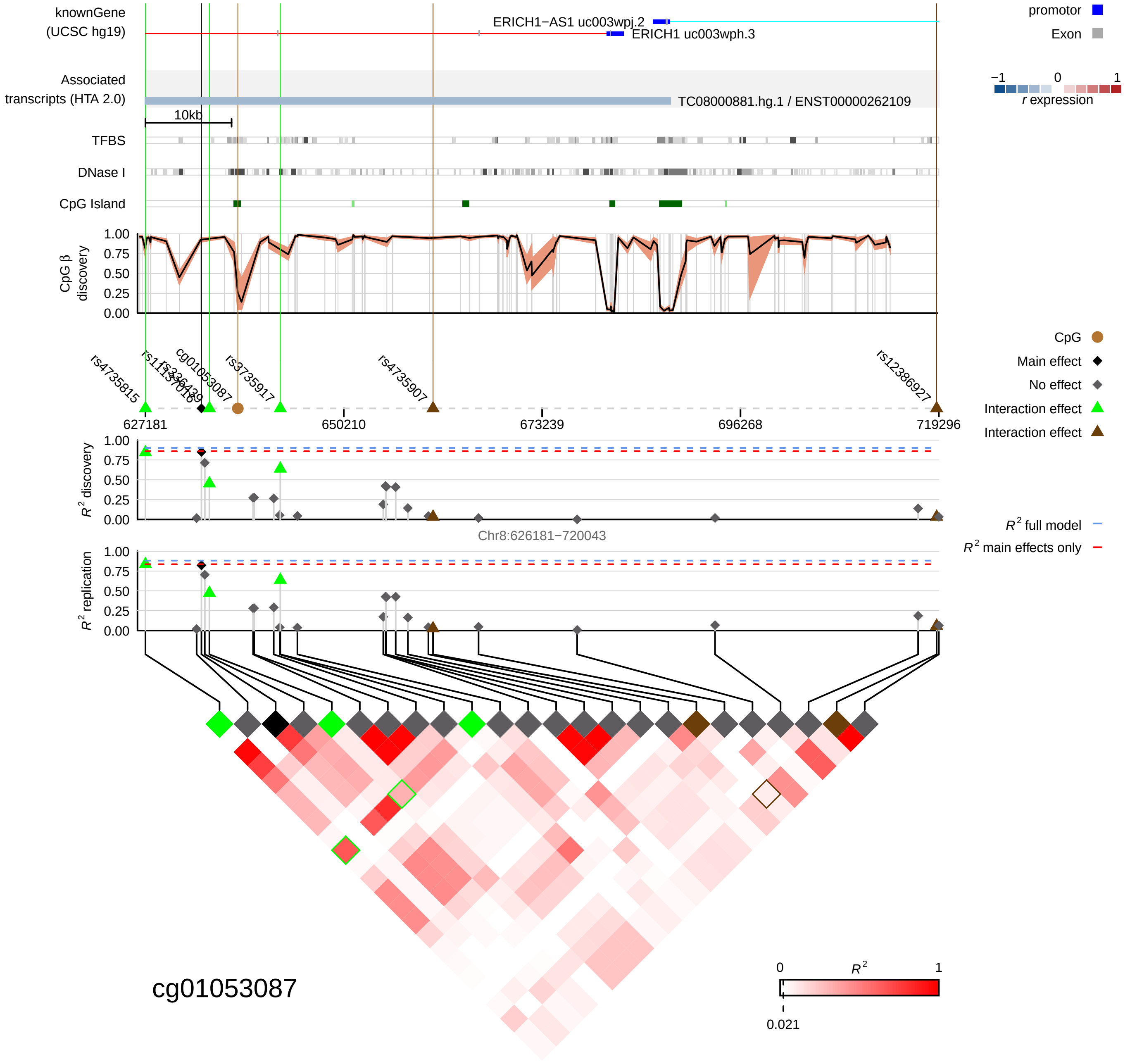

knownGene (UCSC hg19)
ERICH1−AS1 uc003wpj.2
ERICH1 uc003wph.3
promotor
Exon
Associated transcripts (HTA 2.0)
TC08000881.hg.1 / ENST00000262109
−1
0
1
r expression
10kb
TFBS
DNase I
CpG Island
CpG β discovery
1.00
0.75
0.50
0.25
0.00
rs4735815
cg01053087
rs3735917
rs4735907
rs12386927
627181
650210
673239
696268
719296
CpG
Main effect
No effect
Interaction effect
Interaction effect
R² discovery
Chr8:626181−720043
R² full model
R² main effects only
R² replication
cg01053087
0
R²
1
0.021

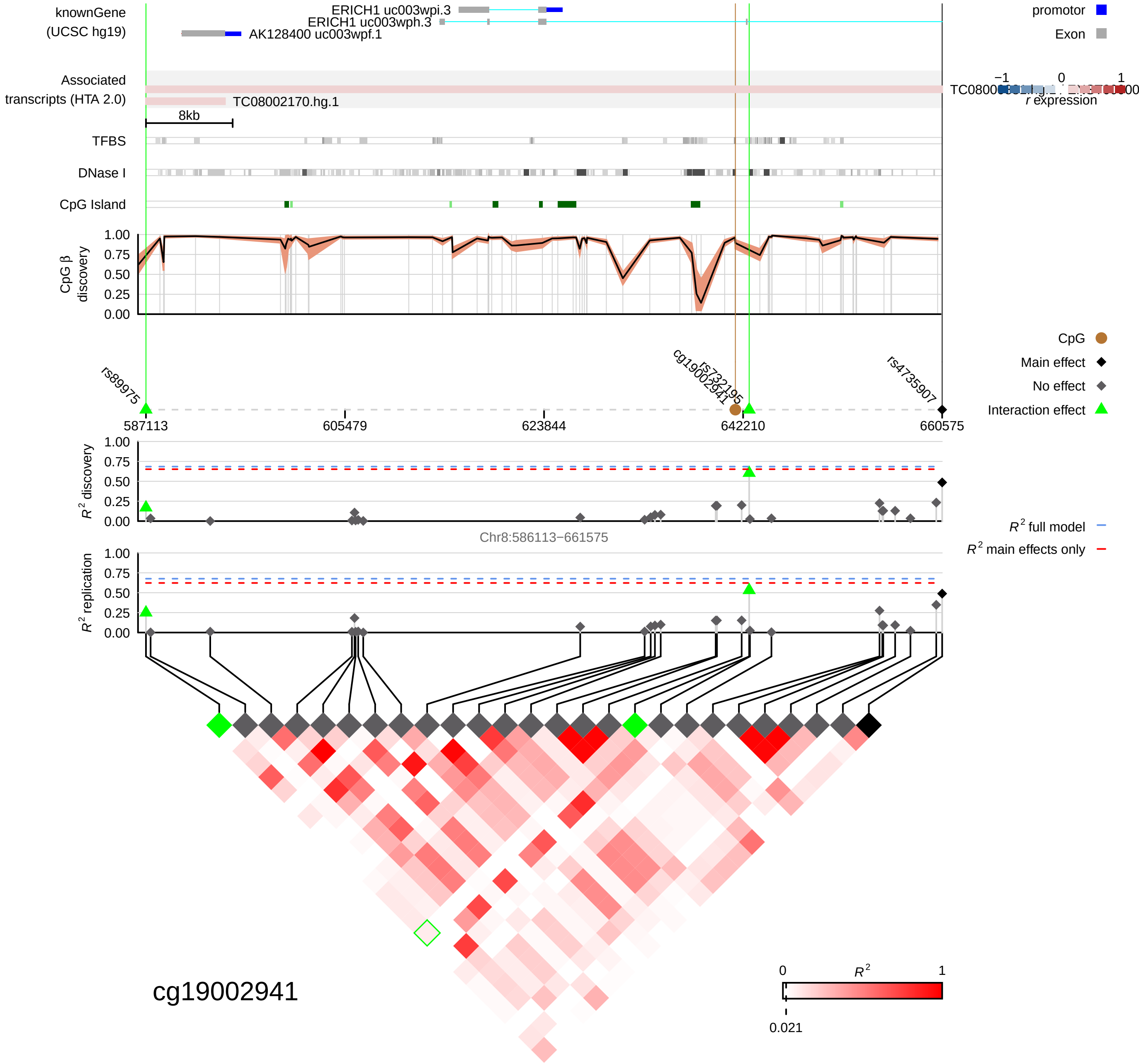

knownGene
(UCSC hg19)
ERICH1 uc003wpi.3
ERICH1 uc003wph.3
AK128400 uc003wpf.1
promotor
Exon
Associated
transcripts (HTA 2.0)
TC08002170.hg.1
8kb
TFBS
DNase I
CpG Island
CpG β
discovery
1.00
0.75
0.50
0.25
0.00
rs89975
cg19002941
rs732195
rs4735907
587113
605479
623844
642210
660575
CpG
Main effect
No effect
Interaction effect
R² discovery
Chr8:586113−661575
R² replication
R² full model
R² main effects only
r expression
−1
0
1
R²
0
1
0.021
cg19002941

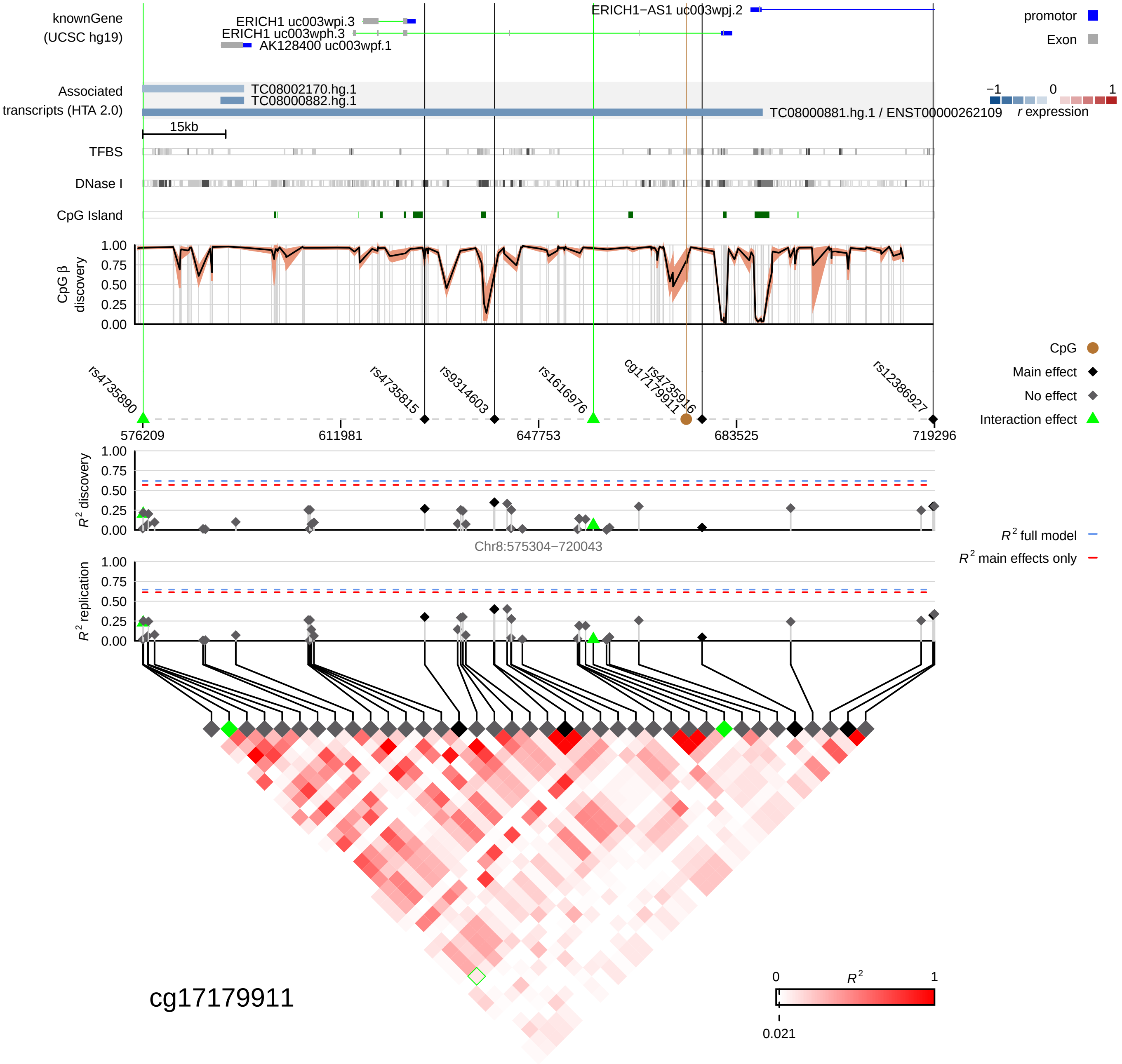
knownGene
(UCSC hg19)
ERICH1 uc003wpi.3
ERICH1 uc003wph.3
AK128400 uc003wpf.1
ERICH1−AS1 uc003wpj.2
promotor
Exon
Associated
transcripts (HTA 2.0)
TC08002170.hg.1
TC08000882.hg.1
TC08000881.hg.1 / ENST00000262109
−1
0
1
r expression
15kb
TFBS
DNase I
CpG Island
CpG β discovery
1.00
0.75
0.50
0.25
0.00
rs4735890
rs4735815
rs9314603
rs1616976
cg17179911
rs4735916
rs12386927
CpG
Main effect
No effect
Interaction effect
576209
611981
647753
683525
719296
R2 discovery
Chr8:575304−720043
R2 full model
R2 main effects only
R2 replication
0
R2
1
0.021
cg17179911

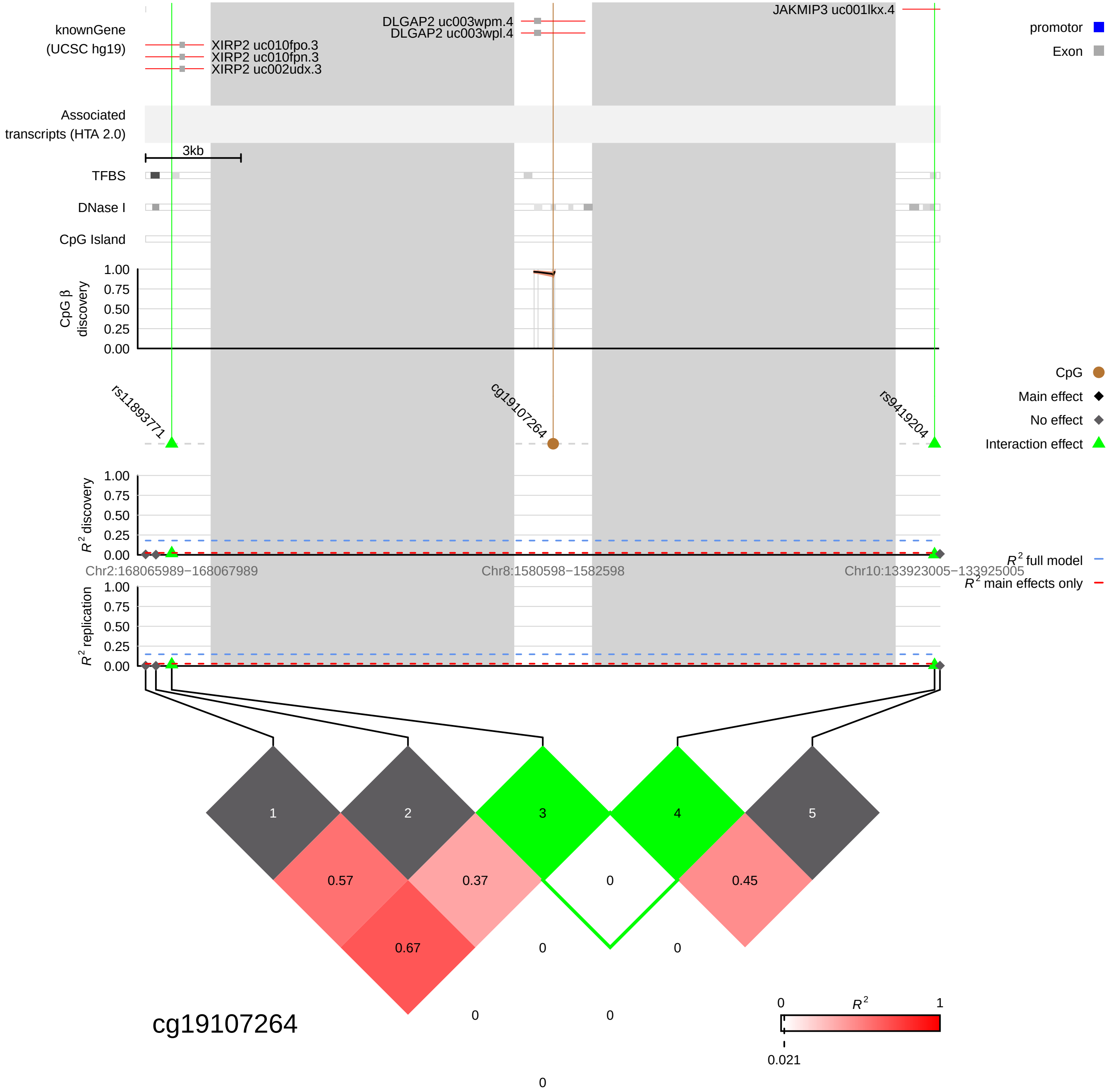
knownGene
(UCSC hg19)
XIRP2 uc010fpo.3
XIRP2 uc010fpn.3
XIRP2 uc002udx.3
DLGAP2 uc003wpm.4
DLGAP2 uc003wpl.4
JAKMIP3 uc001lkx.4
promotor
Exon
Associated
transcripts (HTA 2.0)
3kb
TFBS
DNase I
CpG Island
CpG β
discovery
1.00
0.75
0.50
0.25
0.00
rs11893771
cg19107264
rs9419204
CpG
Main effect
No effect
Interaction effect
R2 discovery
R2 full model
R2 main effects only
Chr2:168065989−168067989
Chr8:1580598−1582598
Chr10:133923005−133925005
R2 replication
1
2
3
4
5
0.57
0.37
0
0.45
0.67
0
0
0
0
0
R2
0
1
0.021
cg19107264

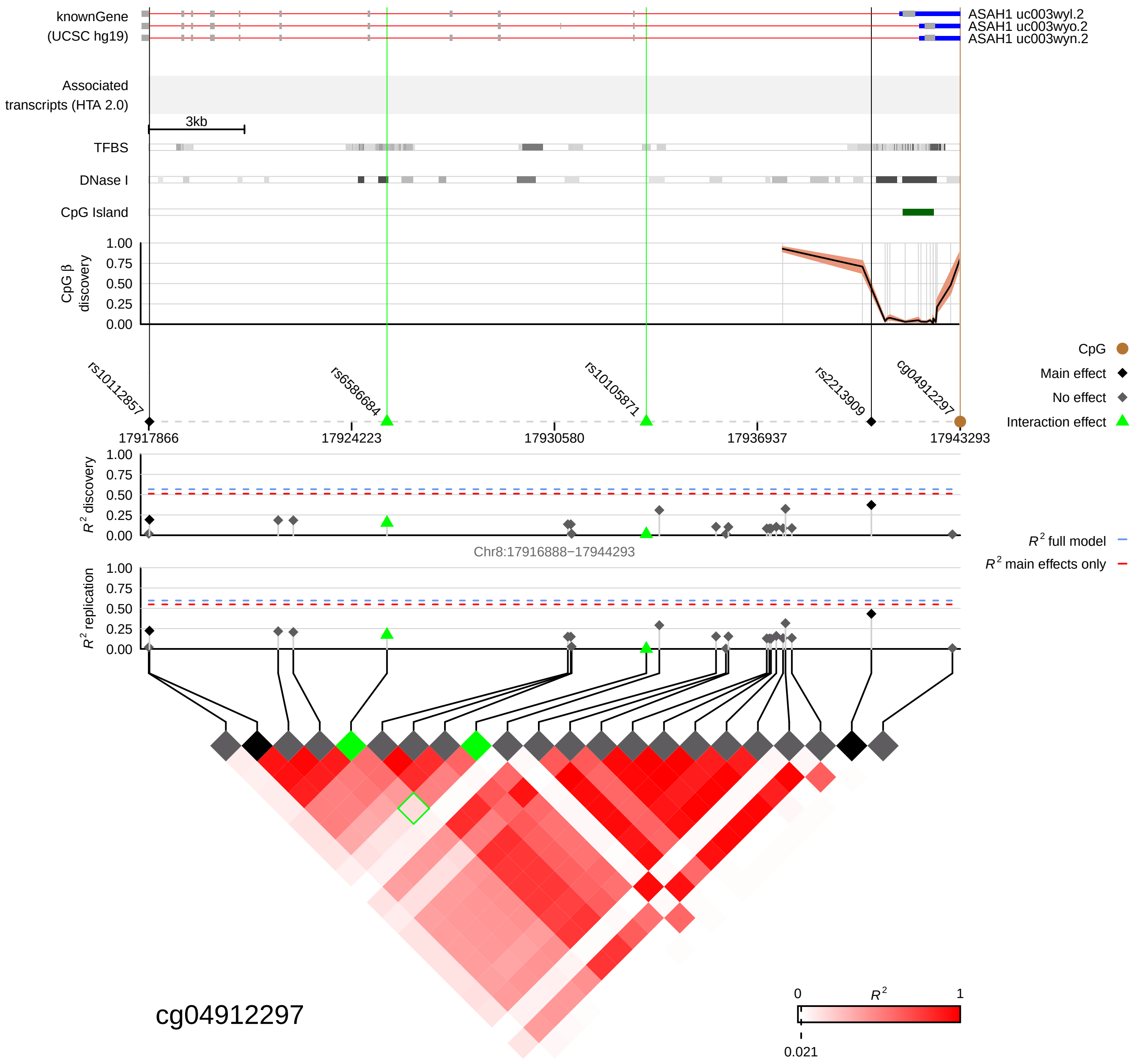

knownGene
(UCSC hg19)
ASAH1 uc003wyl.2
ASAH1 uc003wyo.2
ASAH1 uc003wyn.2
Associated transcripts (HTA 2.0)
3kb
TFBS
DNase I
CpG Island
CpG β discovery
1.00
0.75
0.50
0.25
0.00
rs10112857
rs6586684
rs10105871
rs2213909
cg04912297
CpG
Main effect
No effect
Interaction effect
17917866
17924223
17930580
17936937
17943293
R^2 discovery
Chr8:17916888−17944293
R^2 full model
R^2 main effects only
R^2 replication
cg04912297
0
R^2
1
0.021

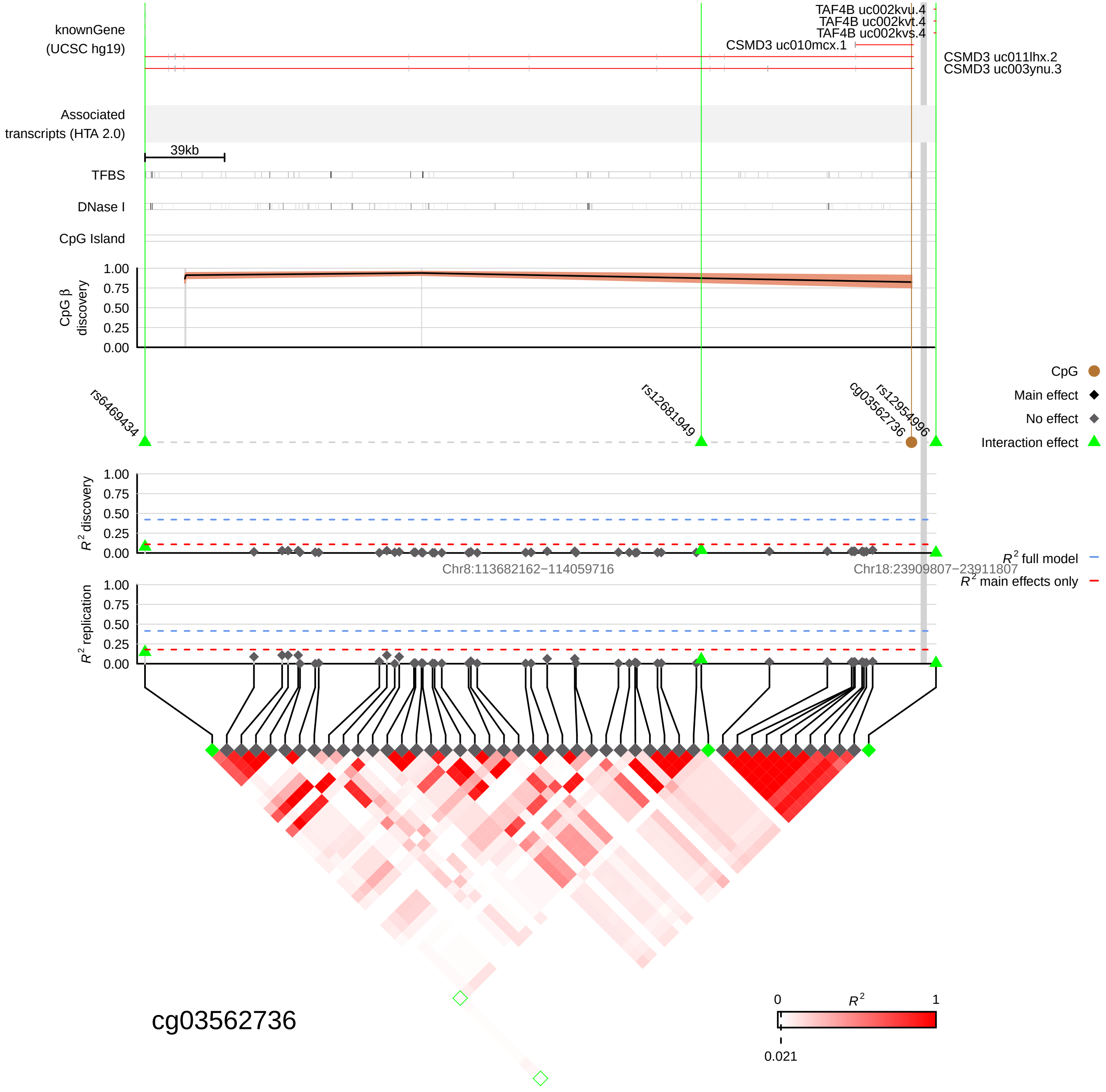

knownGene
(UCSC hg19)
TAF4B uc002kvu.4
TAF4B uc002kvt.4
TAF4B uc002kvs.4
CSMD3 uc010mcx.1
CSMD3 uc011lhx.2
CSMD3 uc003ynu.3
Associated
transcripts (HTA 2.0)
39kb
TFBS
DNase I
CpG Island
CpG β
discovery
rs6469434
rs12681949
cg03562736
rs12954996
CpG
Main effect
No effect
Interaction effect
R² discovery
Chr8:113682162−114059716
Chr18:23909807−23911807
R² full model
R² main effects only
R² replication
0
R²
1
0.021
cg03562736

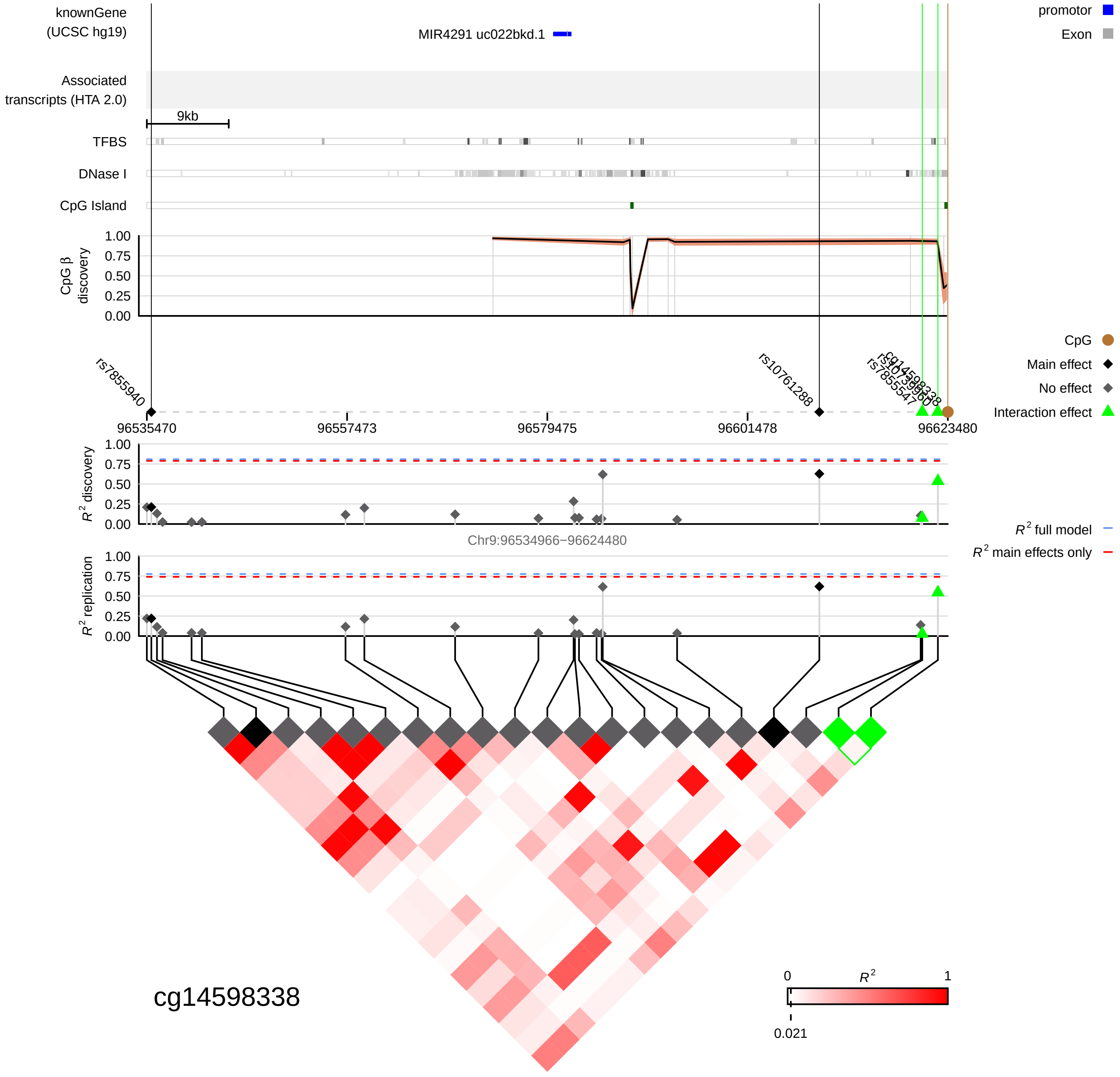
knownGene
(UCSC hg19)
MIR4291 uc022bkd.1
promotor
Exon
Associated
transcripts (HTA 2.0)
9kb
TFBS
DNase I
CpG Island
CpG β
discovery
1.00
0.75
0.50
0.25
0.00
rs7855940
rs10761288
cg14598338
rs10739960
rs7855547
CpG
Main effect
No effect
Interaction effect
96535470
96557473
96579475
96601478
96623480
R^2 discovery
Chr9:96534966−96624480
R^2 replication
R^2 full model
R^2 main effects only
0
R^2
1
0.021
cg14598338

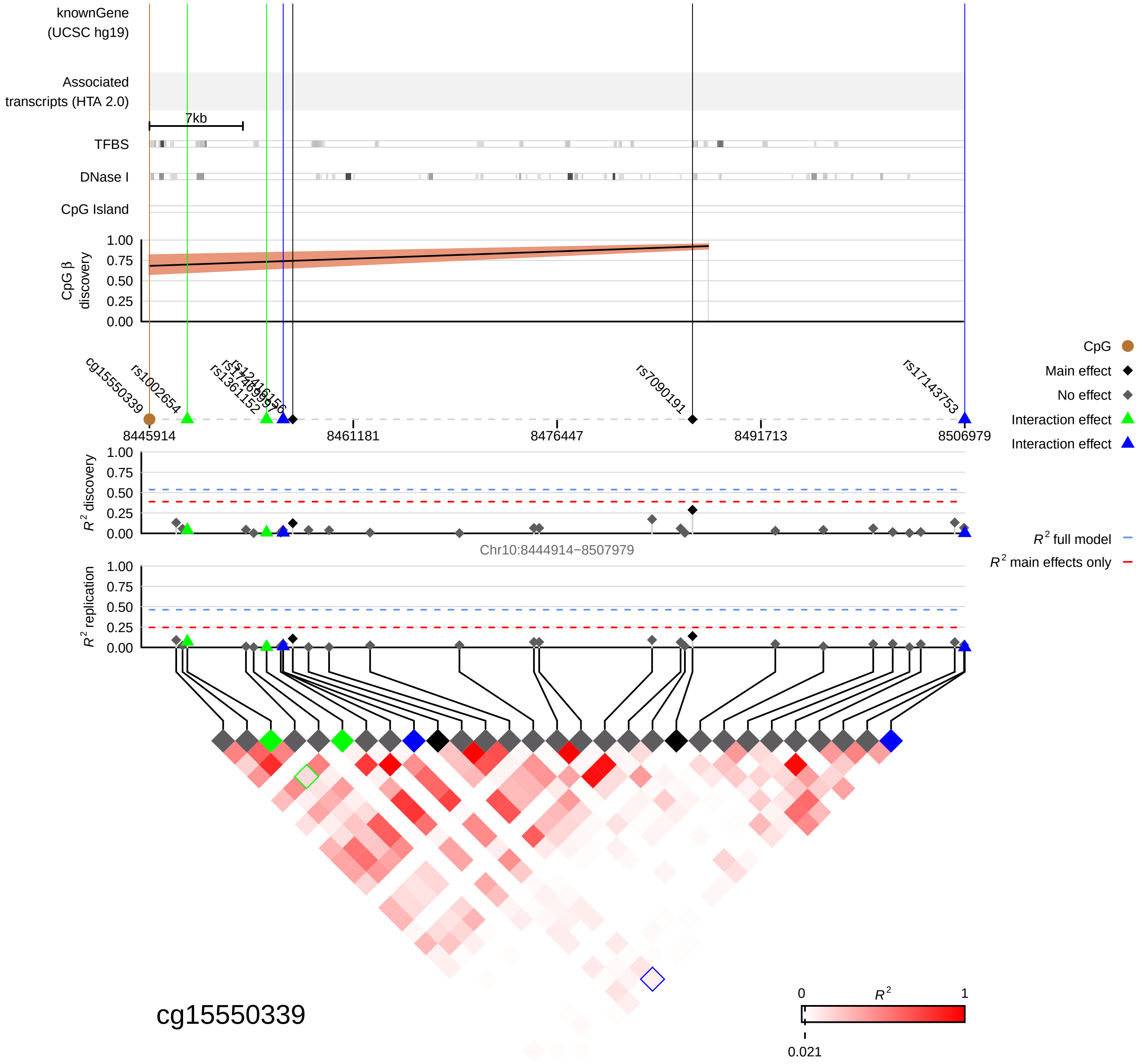
knownGene
(UCSC hg19)
Associated
transcripts (HTA 2.0)
7kb
TFBS
DNase I
CpG Island
CpG β
discovery
1.00
0.75
0.50
0.25
0.00
cg15550339
rs1002654
rs7090191
rs17143753
8445914
8461181
8476447
8491713
8506979
CpG
Main effect
No effect
Interaction effect
Interaction effect
R^2 discovery
R^2 full model
R^2 main effects only
Chr10:8444914−8507979
R^2 replication
0
R^2
1
0.021
cg15550339

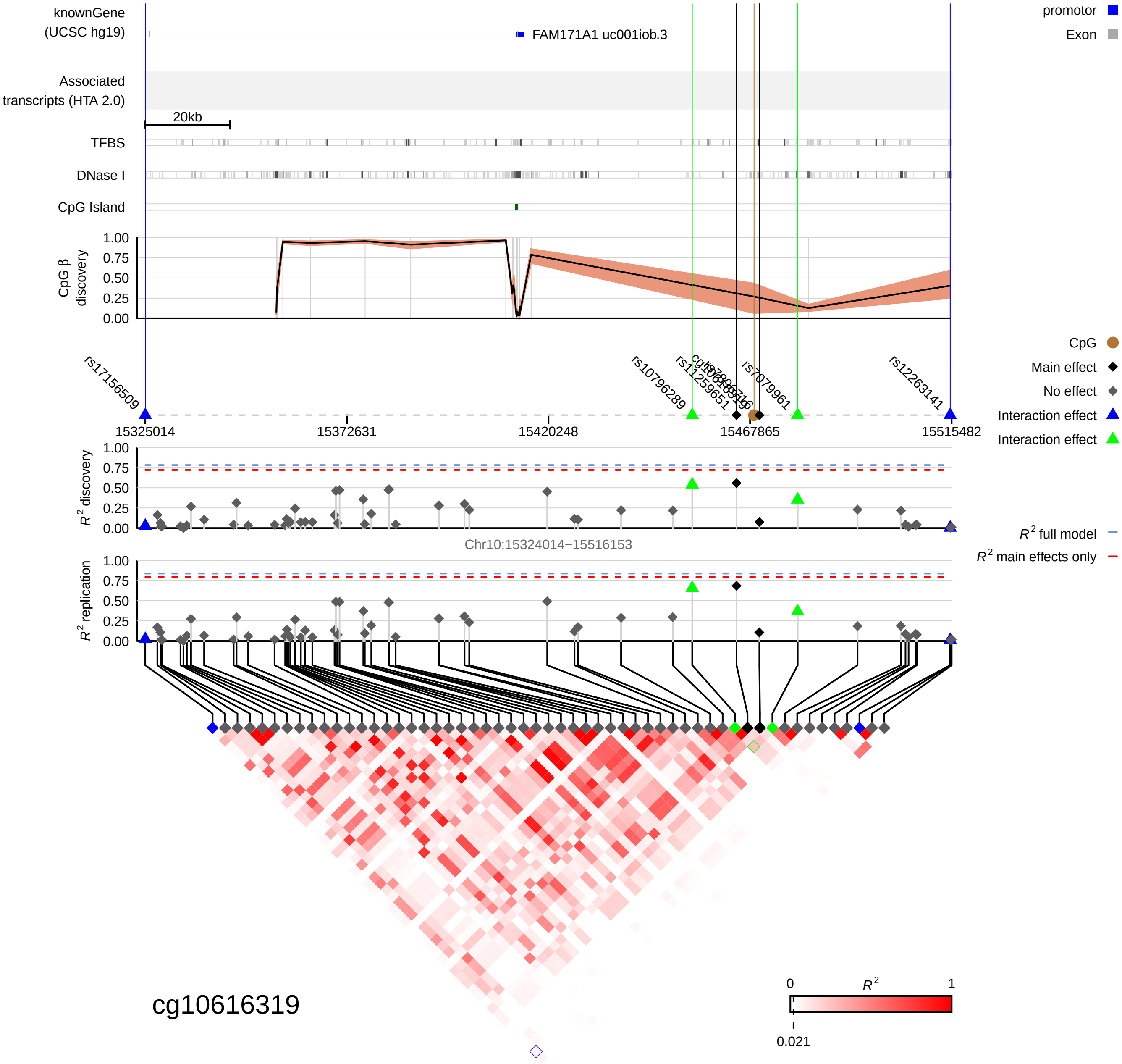
knownGene
(UCSC hg19)
FAM171A1 uc001iob.3
Associated
transcripts (HTA 2.0)
20kb
TFBS
DNase I
CpG Island
CpG β discovery
1.00
0.75
0.50
0.25
0.00
rs17156509
rs10796289
rs11259651
rs7079961
rs12263141
15325014
15372631
15420248
15467865
15515482
R^2 discovery
Chr10:15324014−15516153
R^2 replication
promotor
Exon
CpG
Main effect
No effect
Interaction effect
Interaction effect
R^2 full model
R^2 main effects only
0
R^2
1
0.021
cg10616319

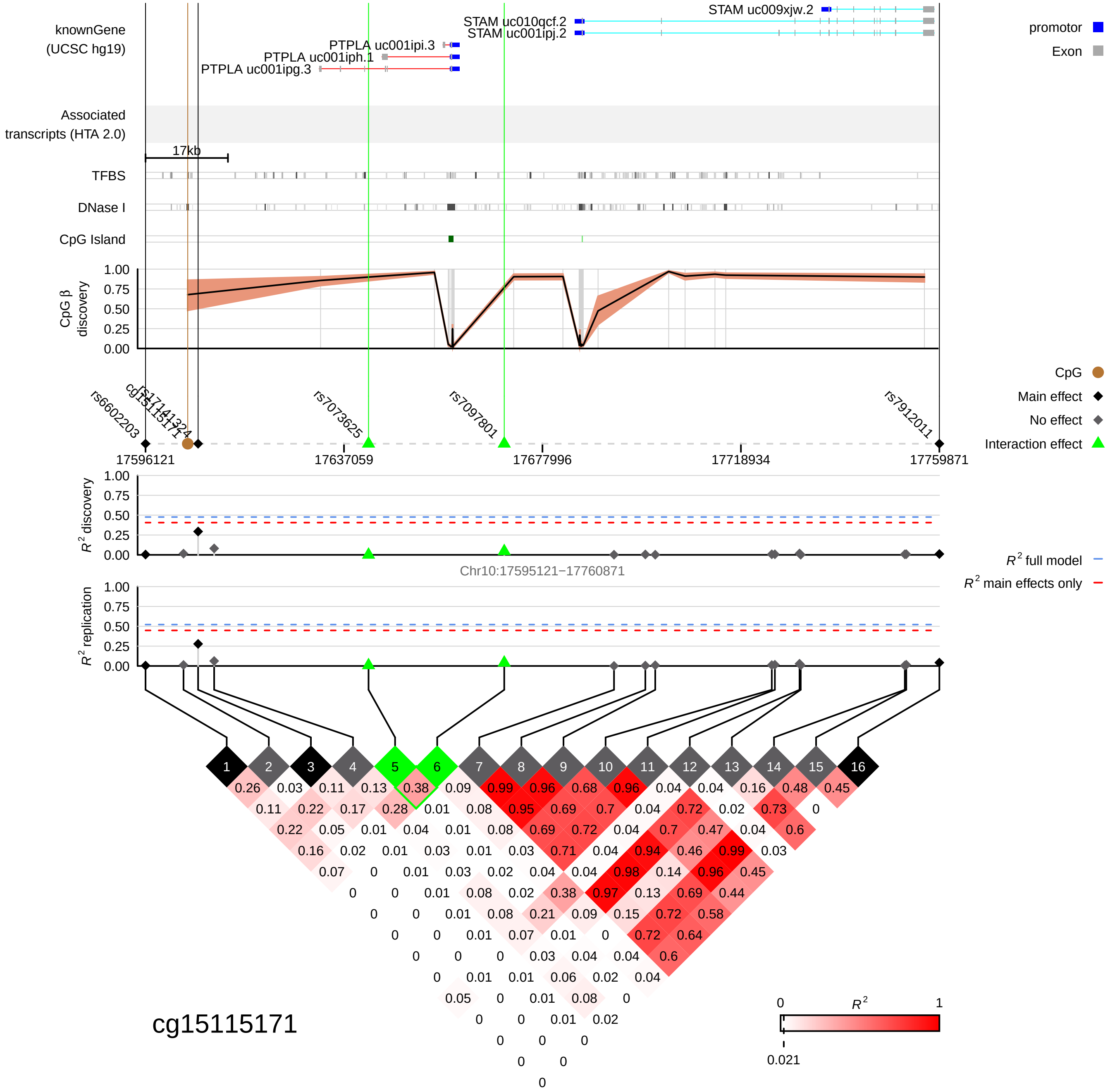

knownGene (UCSC hg19)
Associated transcripts (HTA 2.0)
TFBS
DNase I
CpG Island
CpG β discovery
STAM uc009xjw.2
STAM uc010qcf.2
STAM uc001ipj.2
PTPLA uc001ipi.3
PTPLA uc001iph.1
PTPLA uc001ipg.3
17kb
promotor
Exon
CpG
Main effect
No effect
Interaction effect
rs6602203
cg15115171
rs7073625
rs7097801
rs7912011
17596121
17637059
17677996
17718934
17759871
R^2 discovery
R^2 replication
Chr10:17595121−17760871
R^2 full model
R^2 main effects only
0.021

cg15115171

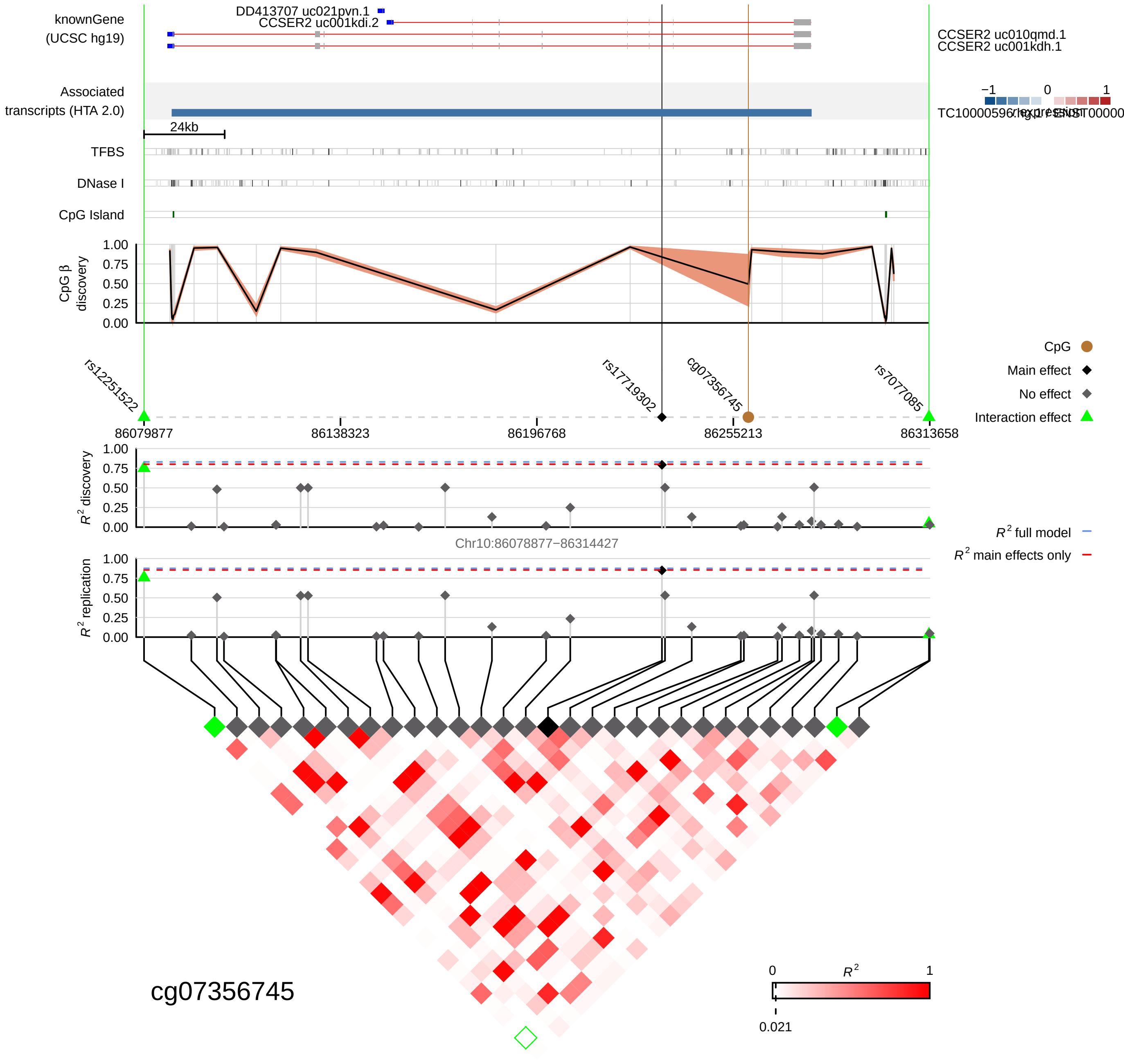

knownGene
(UCSC hg19)
DD413707 uc021pvn.1
CCSER2 uc001kdi.2
CCSER2 uc010qmd.1
CCSER2 uc001kdh.1
Associated
transcripts (HTA 2.0)
24kb
TFBS
DNase I
CpG Island
CpG β
discovery
rs12251522
rs17719302
cg07356745
rs7077085
86079877
86138323
86196768
86255213
86313658
CpG
Main effect
No effect
Interaction effect
R^2 discovery
Chr10:86078877−86314427
R^2 full model
R^2 main effects only
R^2 replication
cg07356745
0
R^2
1
0.021

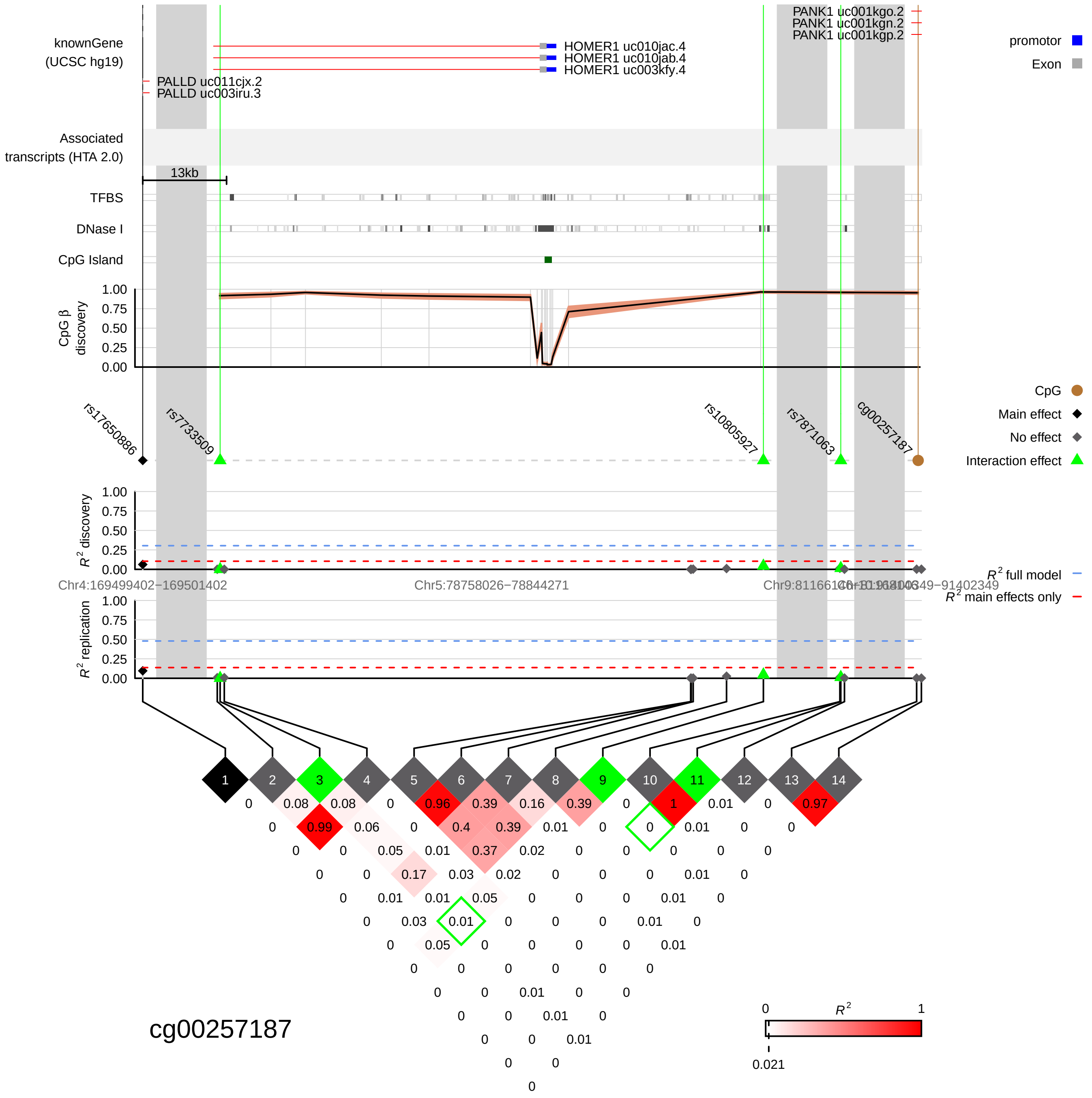

knownGene (UCSC hg19)
PANK1 uc001kgo.2
PANK1 uc001kgn.2
PANK1 uc001kgp.2
HOMER1 uc010jac.4
HOMER1 uc010jab.4
HOMER1 uc003kfy.4
PALLD uc011cjx.2
PALLD uc003iru.3
promotor
Exon
Associated transcripts (HTA 2.0)
13kb
TFBS
DNase I
CpG Island
CpG β discovery
rs17650886
rs7733509
rs10805927
rs7871063
cg00257187
CpG
Main effect
No effect
Interaction effect
R^2 discovery
R^2 full model
R^2 main effects only
Chr4:169499402−169501402
Chr5:78758026−78844271
Chr9:81166140−81196140
R^2 replication
R^2
0.021

cg00257187

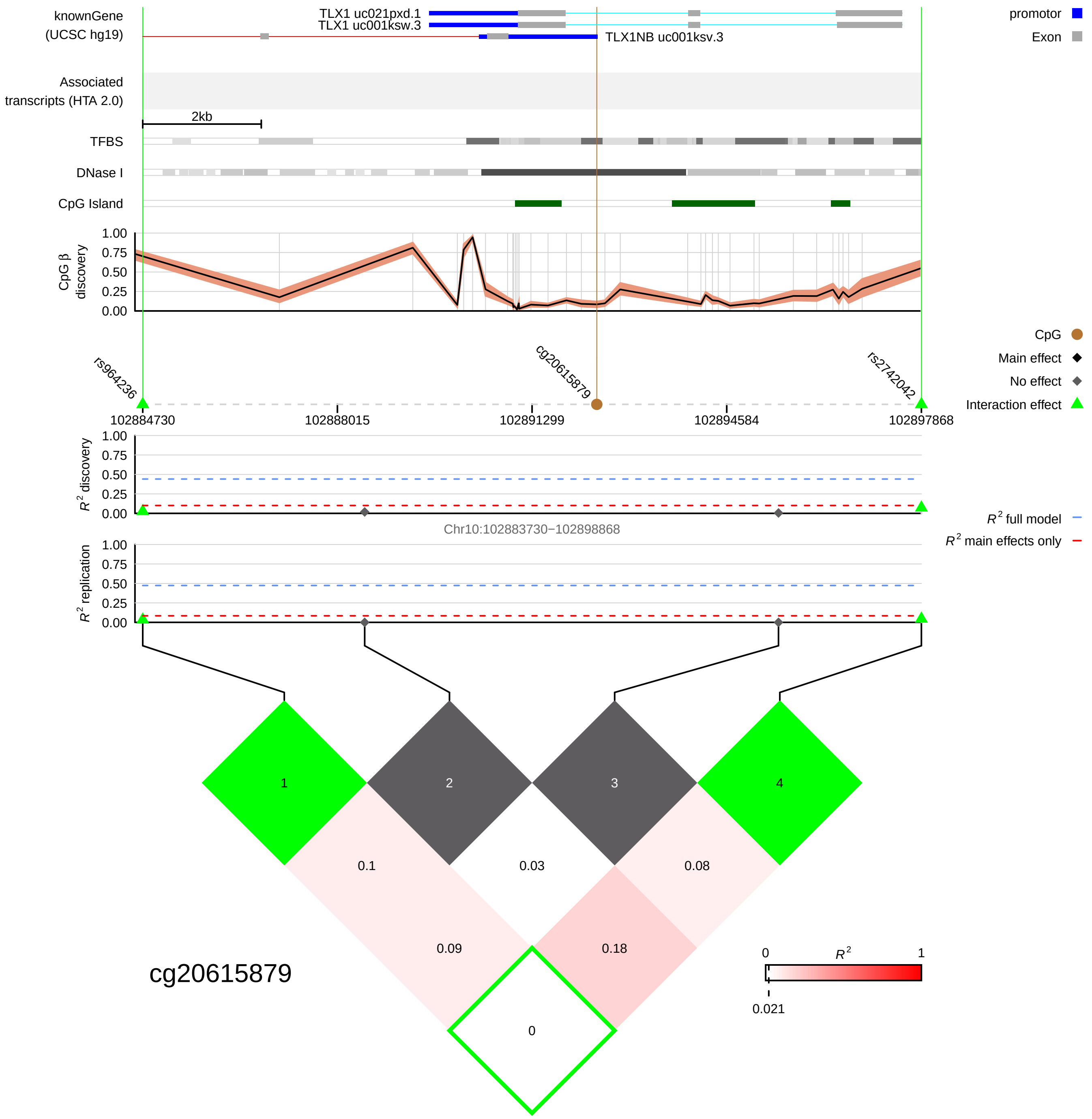

knownGene (UCSC hg19)
TLX1 uc021pxd.1
TLX1 uc001ksw.3
TLX1NB uc001ksv.3
promotor
Exon
Associated transcripts (HTA 2.0)
2kb
TFBS
DNase I
CpG Island
CpG β discovery
1.00
0.75
0.50
0.25
0.00
CpG
Main effect
No effect
Interaction effect
rs964236
cg20615879
rs2742042
102884730
102888015
102891299
102894584
102897868
R^2 discovery
Chr10:102883730−102898868
R^2 full model
R^2 main effects only
R^2 replication
1
2
3
4
0.1
0.03
0.08
0.09
0.18
0
cg20615879
0
R^2
1
0.021

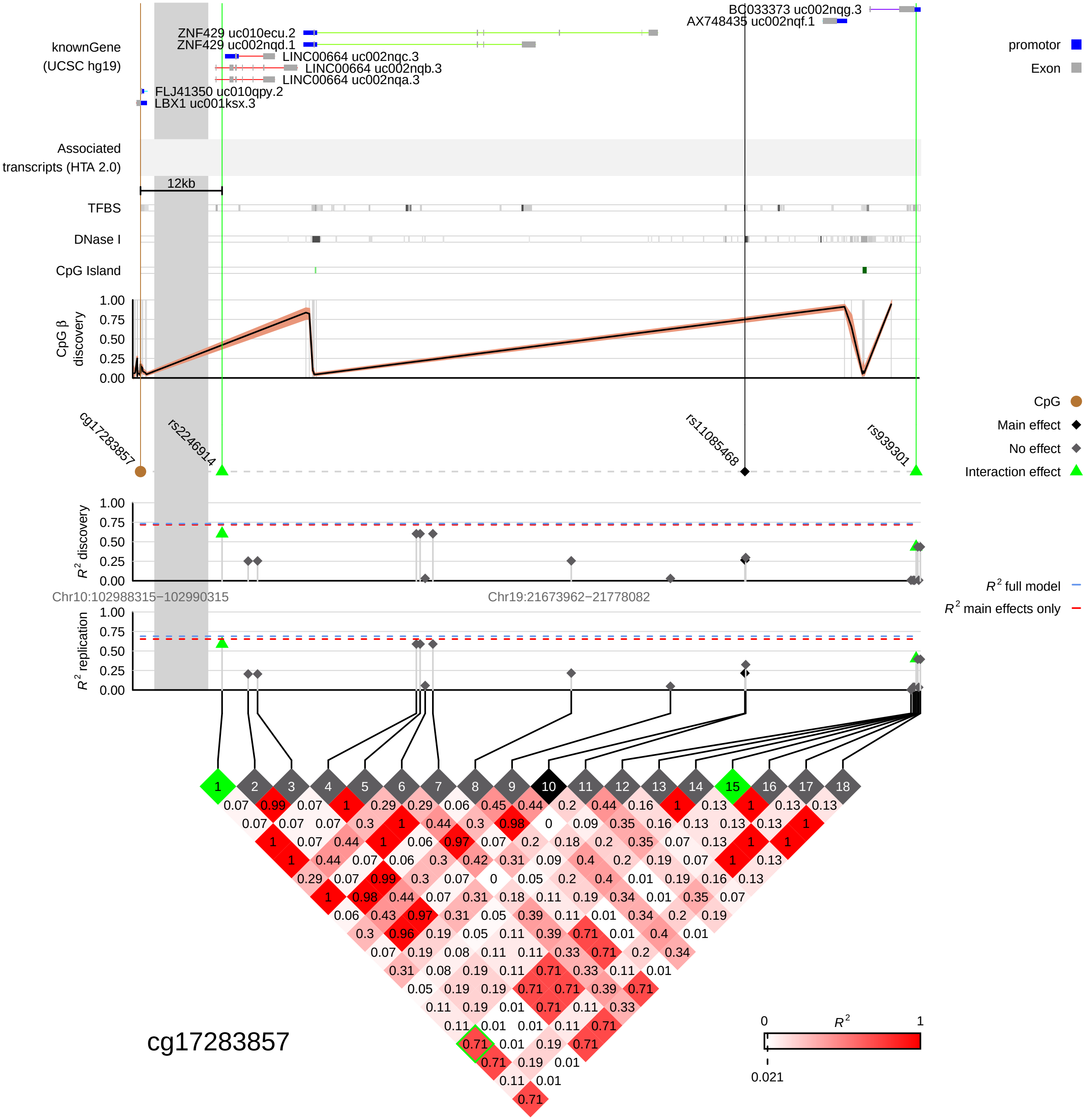

knownGene (UCSC hg19)
BC033373 uc002nqg.3
AX748435 uc002nqf.1
ZNF429 uc010ecu.2
ZNF429 uc002nqd.1
LINC00664 uc002nqc.3
LINC00664 uc002nqb.3
LINC00664 uc002nqa.3
FLJ41350 uc010qpy.2
LBX1 uc001ksx.3
promotor
Exon
Associated transcripts (HTA 2.0)
12kb
TFBS
DNase I
CpG Island
CpG β discovery
cg17283857
rs2246914
rs11085468
rs939301
CpG
Main effect
No effect
Interaction effect
R^2 discovery
Chr10:102988315−102990315
Chr19:21673962−21778082
R^2 full model
R^2 main effects only
R^2 replication
R^2
0.021
cg17283857

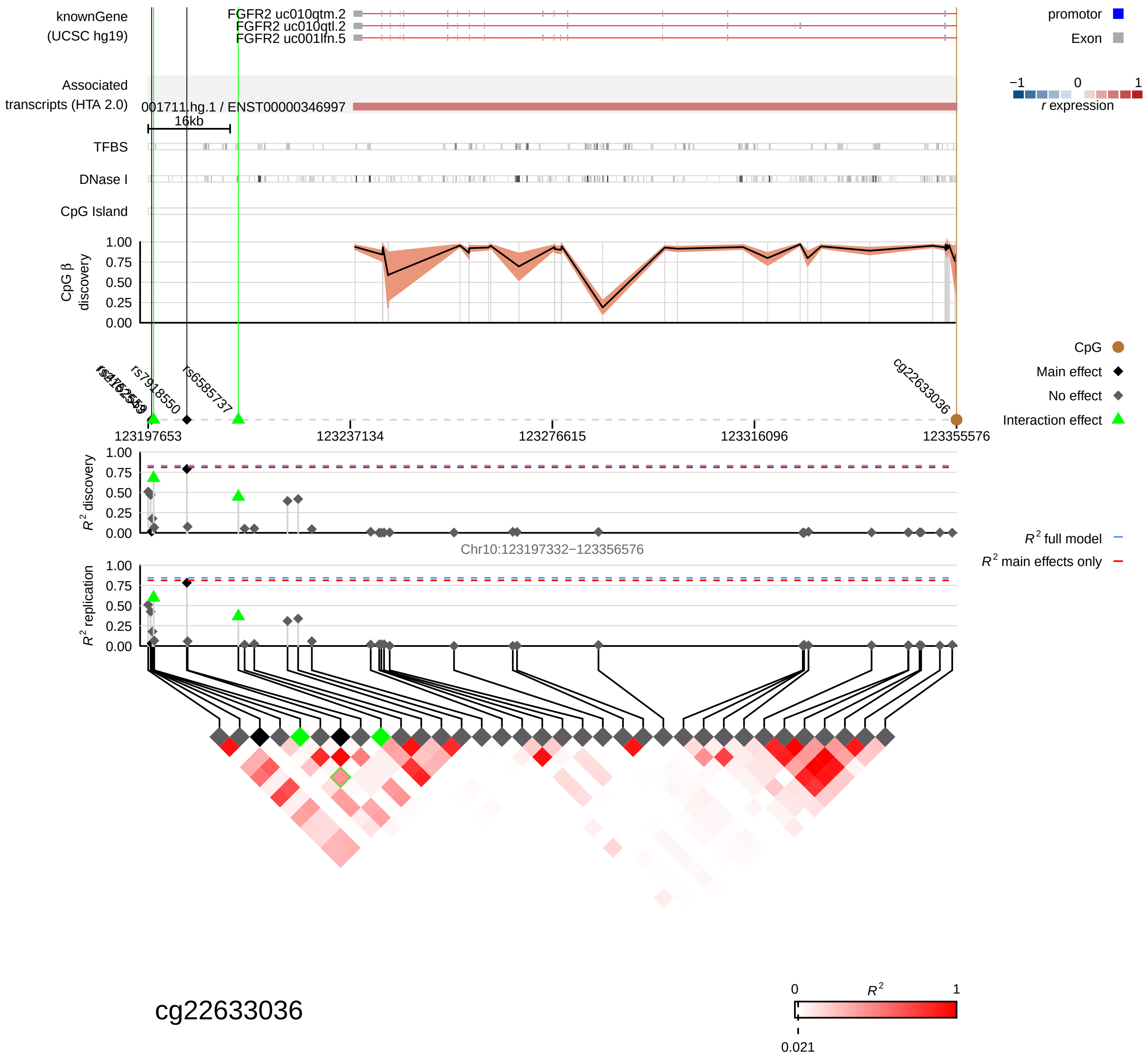

knownGene (UCSC hg19)
FGFR2 uc010qtm.2
FGFR2 uc010qtl.2
FGFR2 uc001lfn.5
promotor
Exon
Associated transcripts (HTA 2.0)
001711.hg.1 / ENST00000346997
−1
0
1
r expression
16kb
TFBS
DNase I
CpG Island
CpG β discovery
1.00
0.75
0.50
0.25
0.00
rs7918550
rs6585737
cg22633036
CpG
Main effect
No effect
Interaction effect
123197653
123237134
123276615
123316096
123355576
R² discovery
Chr10:123197332−123356576
R² full model
R² main effects only
R² replication
0
R²
1
0.021

cg22633036

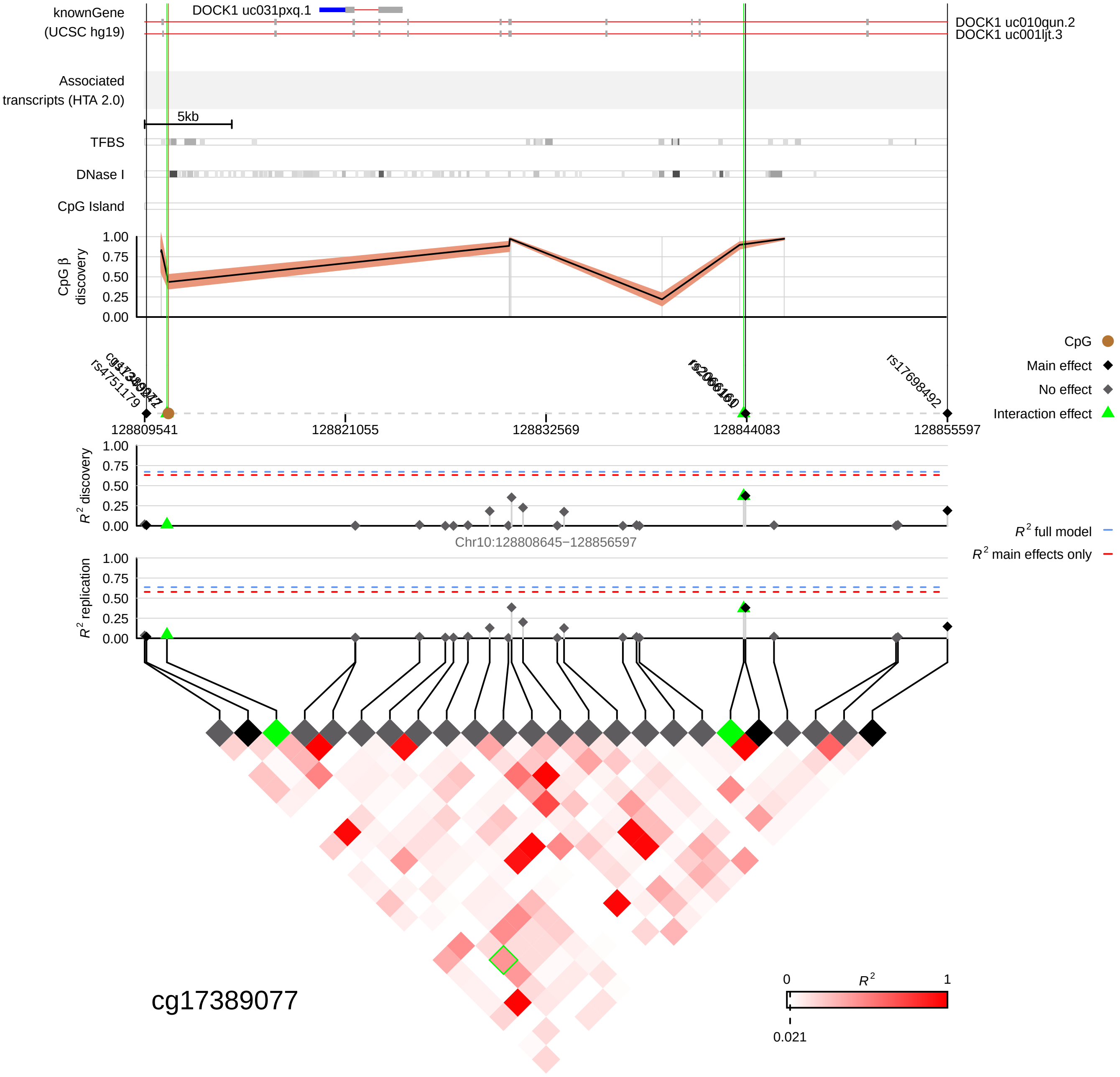

knownGene
(UCSC hg19)
DOCK1 uc031pxq.1
DOCK1 uc010qun.2
DOCK1 uc001ljt.3
Associated
transcripts (HTA 2.0)
5kb
TFBS
DNase I
CpG Island
CpG β
discovery
1.00
0.75
0.50
0.25
0.00
rs4751179
rs17698492
128809541
128821055
128832569
128844083
128855597
CpG
Main effect
No effect
Interaction effect
R^2 discovery
Chr10:128808645−128856597
R^2 full model
R^2 main effects only
R^2 replication
0
R^2
1
0.021
cg17389077

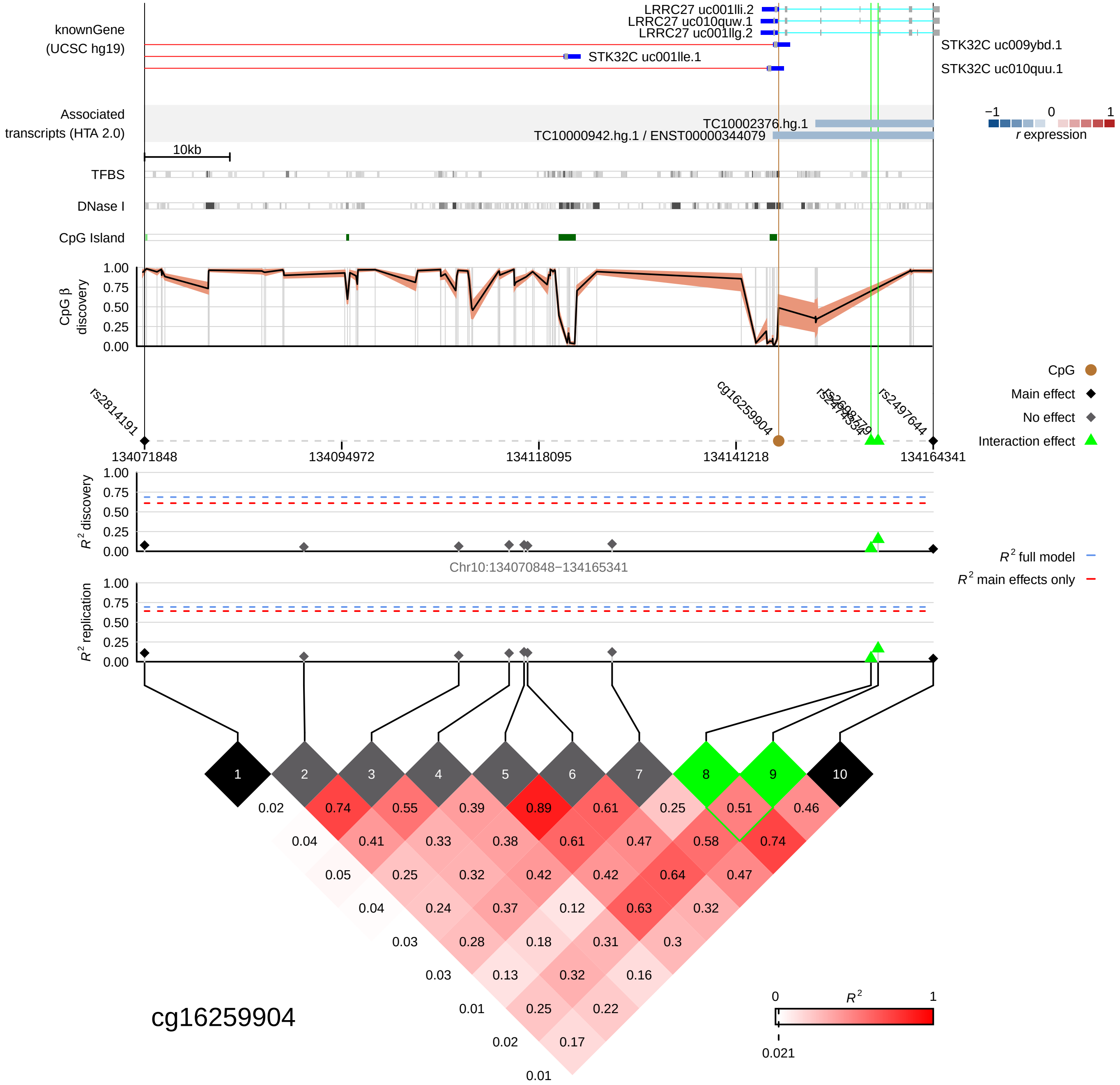
knownGene
(UCSC hg19)
LRRC27 uc001lli.2
LRRC27 uc010quw.1
LRRC27 uc001llg.2
STK32C uc009ybd.1
STK32C uc001lle.1
STK32C uc010quu.1
Associated
transcripts (HTA 2.0)
TC10002376.hg.1
TC10000942.hg.1 / ENST00000344079
−1
0
1
r expression
10kb
TFBS
DNase I
CpG Island
CpG β
discovery
1.00
0.75
0.50
0.25
0.00
rs2814191
cg16259904
rs2497644
134071848
134094972
134118095
134141218
134164341
CpG
Main effect
No effect
Interaction effect
R² discovery
Chr10:134070848−134165341
R² full model
R² main effects only
R² replication
1
2
3
4
5
6
7
8
9
10
0.02
0.74
0.55
0.39
0.89
0.61
0.25
0.51
0.46
0.04
0.41
0.33
0.38
0.61
0.47
0.58
0.74
0.05
0.25
0.32
0.42
0.42
0.64
0.47
0.04
0.24
0.37
0.12
0.63
0.32
0.03
0.28
0.18
0.31
0.3
0.03
0.13
0.32
0.16
0.01
0.25
0.22
0.02
0.17
0.01
0
R²
1
0.021
cg16259904

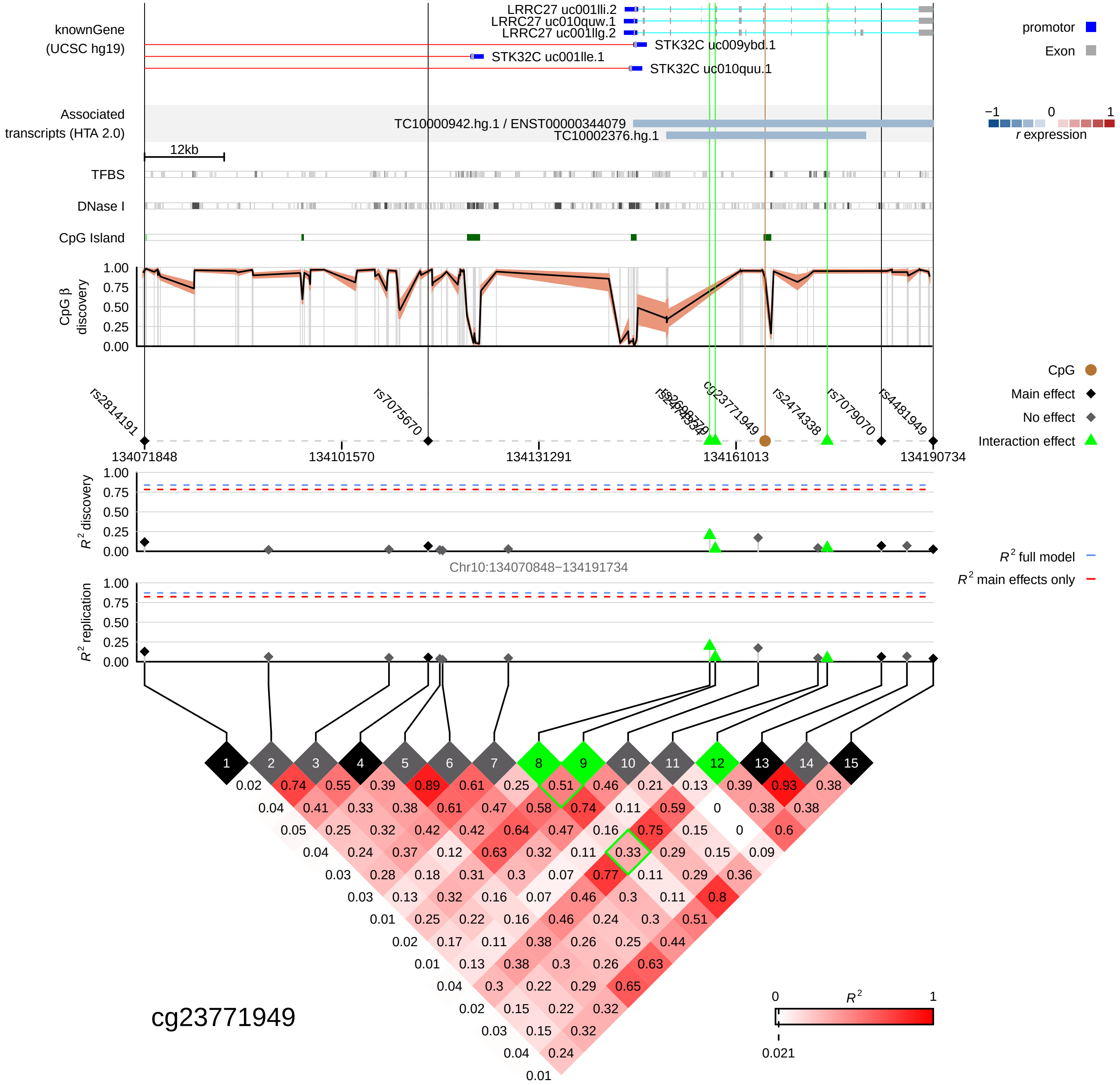

knownGene
(UCSC hg19)
LRRC27 uc001lli.2
LRRC27 uc010quw.1
LRRC27 uc001llg.2
STK32C uc009ybd.1
STK32C uc001lle.1
STK32C uc010quu.1
promotor
Exon
Associated
transcripts (HTA 2.0)
TC10000942.hg.1 / ENST00000344079
TC10002376.hg.1
−1
0
1
r expression
12kb
TFBS
DNase I
CpG Island
CpG β
discovery
1.00
0.75
0.50
0.25
0.00
rs2814191
rs7075670
cg23771949
rs2474338
rs7079070
rs4481949
CpG
Main effect
No effect
Interaction effect
134071848
134101570
134131291
134161013
134190734
R² discovery
Chr10:134070848−134191734
R² full model
R² main effects only
R² replication
1 2 3 4 5 6 7 8 9 10 11 12 13 14 15
0.02 0.74 0.55 0.39 0.89 0.61 0.25 0.51 0.46 0.21 0.13 0.39 0.93 0.38
0.04 0.41 0.33 0.38 0.61 0.47 0.58 0.74 0.11 0.59 0 0.38 0.38
0.05 0.25 0.32 0.42 0.42 0.64 0.47 0.16 0.75 0.15 0 0.6
0.04 0.24 0.37 0.12 0.63 0.32 0.11 0.33 0.29 0.15 0.09
0.03 0.28 0.18 0.31 0.3 0.07 0.77 0.11 0.29 0.36
0.03 0.13 0.32 0.16 0.07 0.46 0.3 0.11 0.8
0.01 0.25 0.22 0.16 0.46 0.24 0.3 0.51
0.02 0.17 0.11 0.38 0.26 0.25 0.44
0.01 0.13 0.38 0.3 0.26 0.63
0.04 0.3 0.22 0.29 0.65
0.02 0.15 0.22 0.32
0.03 0.15 0.32
0.04 0.24
0.01
0
R²
1
0.021

cg23771949

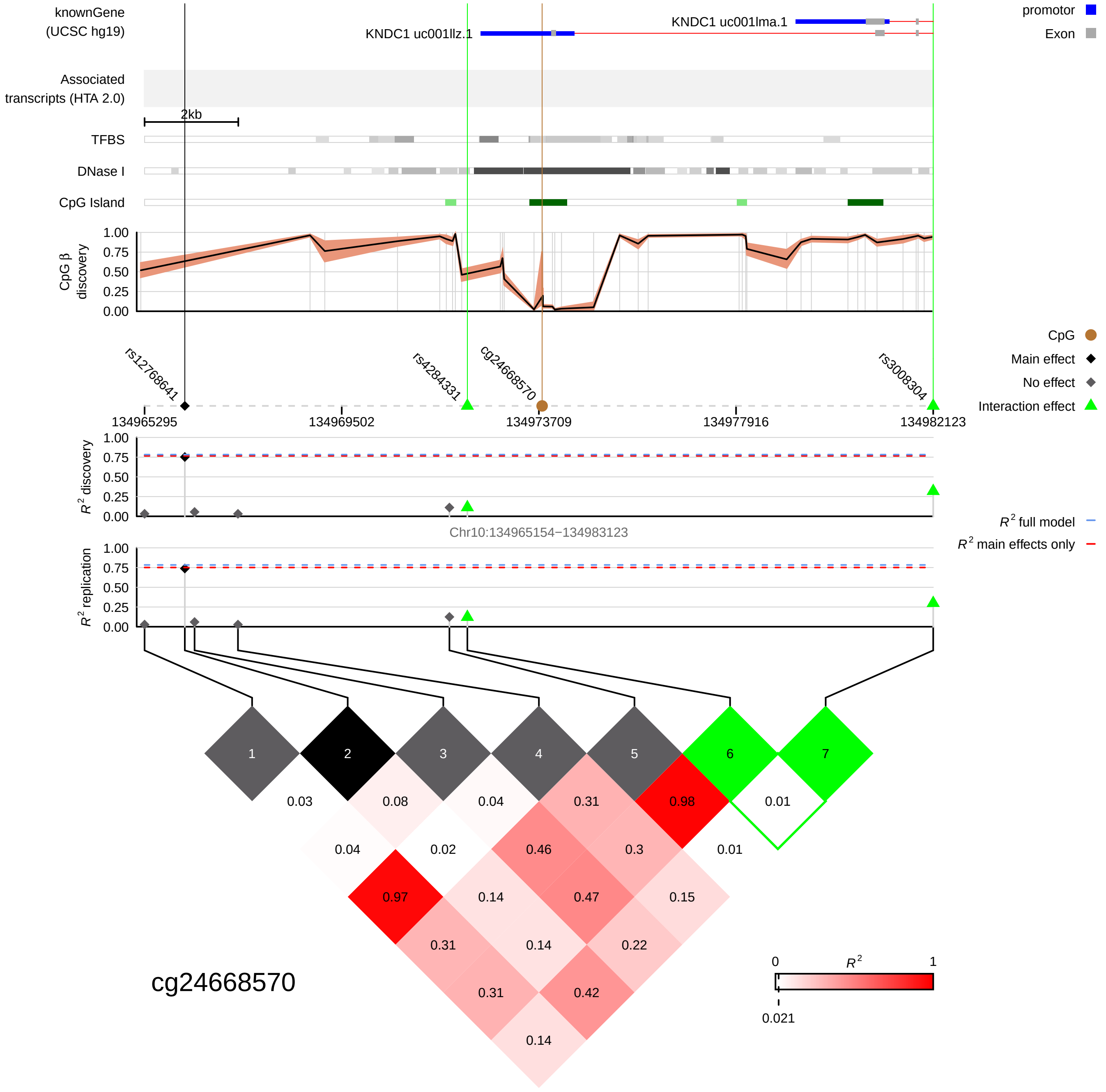
knownGene (UCSC hg19)
KNDC1 uc001llz.1
KNDC1 uc001lma.1
promotor
Exon
Associated transcripts (HTA 2.0)
2kb
TFBS
DNase I
CpG Island
CpG β discovery
rs12768641
rs4284331
cg24668570
rs3008304
CpG
Main effect
No effect
Interaction effect
134965295
134969502
134973709
134977916
134982123
R² discovery
Chr10:134965154−134983123
R² full model
R² main effects only
R² replication
cg24668570
R²
0.021

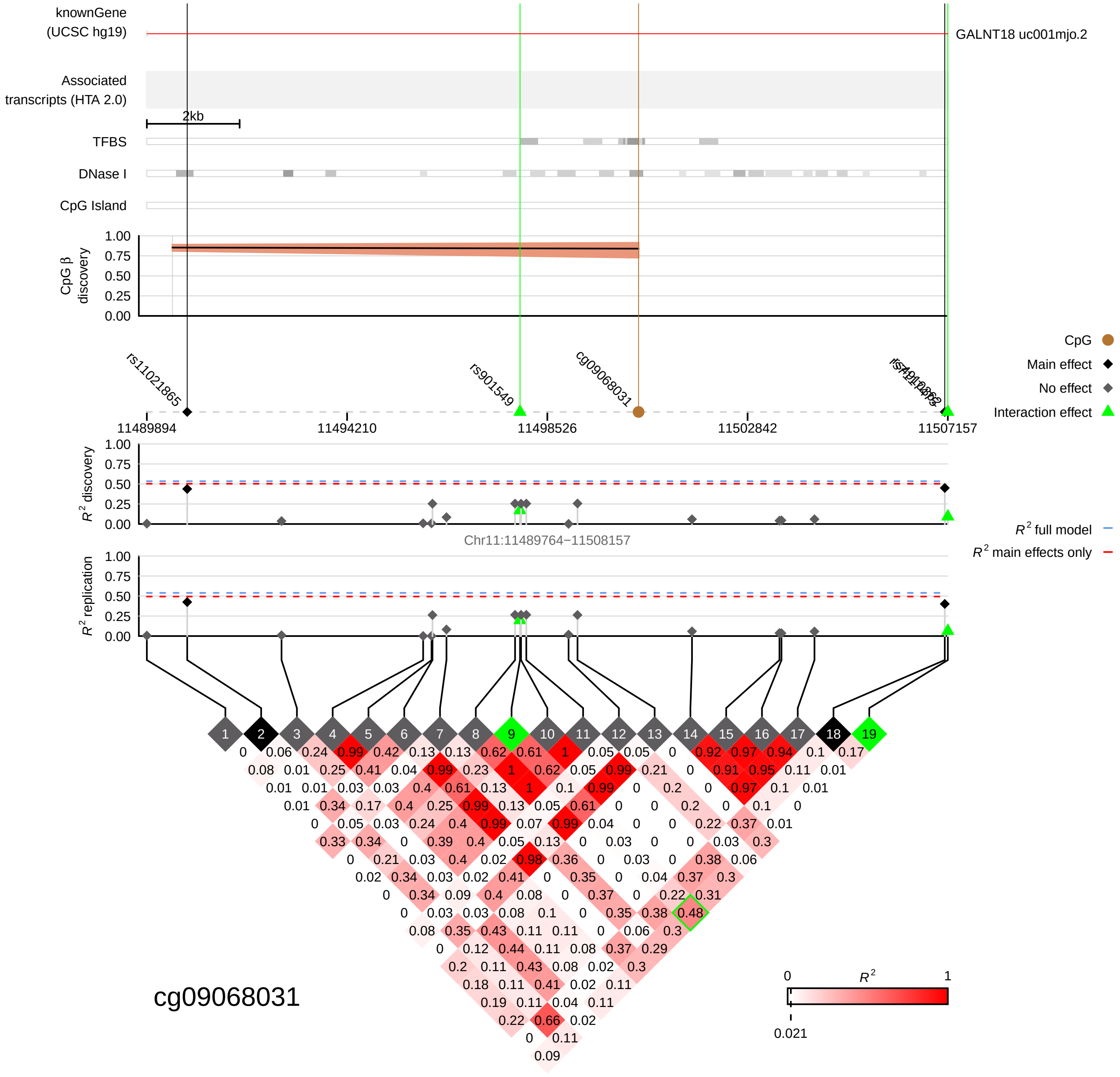
knownGene
(UCSC hg19)
GALNT18 uc001mjo.2
Associated
transcripts (HTA 2.0)
2kb
TFBS
DNase I
CpG Island
CpG β
discovery
rs11021865
rs901549
cg09068031
11489894
11494210
11498526
11502842
11507157
CpG
Main effect
No effect
Interaction effect
R^2 discovery
Chr11:11489764−11508157
R^2 full model
R^2 main effects only
R^2 replication
R^2
0.021
cg09068031

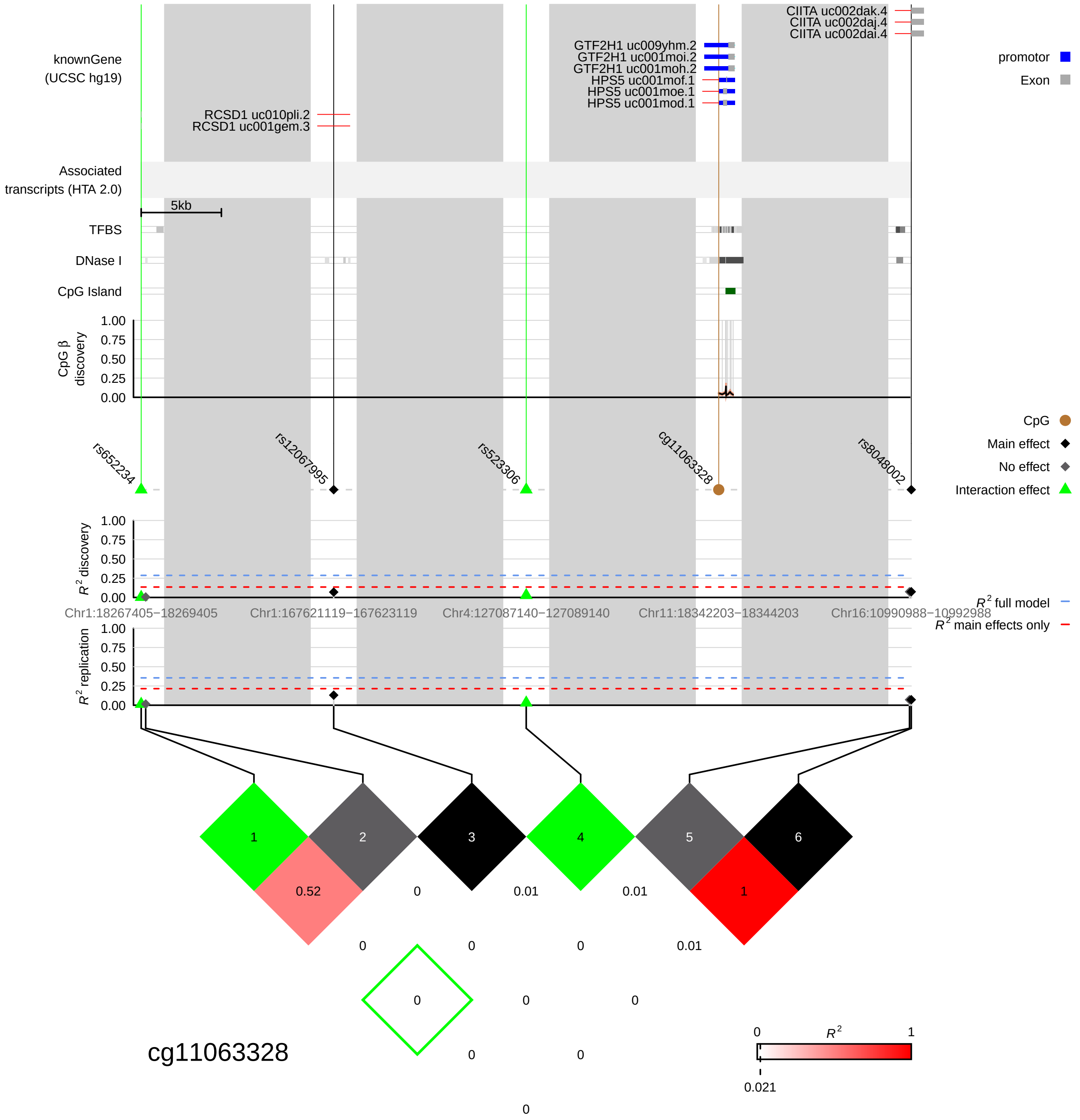

knownGene (UCSC hg19)
Associated transcripts (HTA 2.0)
TFBS
DNase I
CpG Island
CpG β discovery
R^2 discovery
R^2 replication
5kb
RCSD1 uc010pli.2
RCSD1 uc001gem.3
GTF2H1 uc009yhm.2
GTF2H1 uc001moi.2
GTF2H1 uc001moh.2
HPS5 uc001mof.1
HPS5 uc001moe.1
HPS5 uc001mod.1
CIITA uc002dak.4
CIITA uc002daj.4
CIITA uc002dai.4
promotor
Exon
rs652234
rs12067995
rs523306
cg11063328
rs8048002
CpG
Main effect
No effect
Interaction effect
Chr1:18267405−18269405
Chr1:167621119−167623119
Chr4:127087140−127089140
Chr11:18342203−18344203
Chr16:10990988−10992988
R^2 full model
R^2 main effects only
cg11063328
0.021

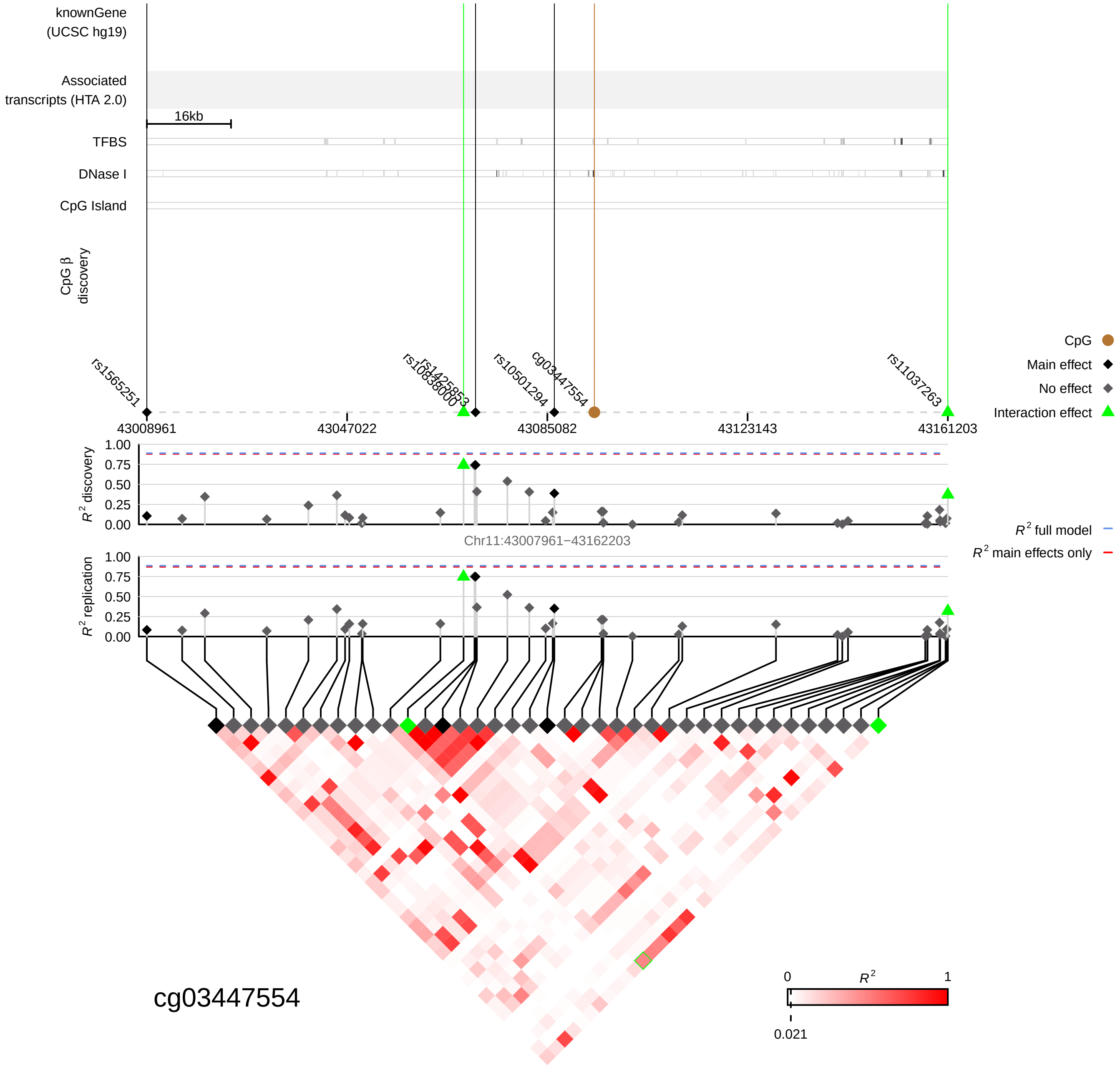
knownGene
(UCSC hg19)
Associated
transcripts (HTA 2.0)
16kb
TFBS
DNase I
CpG Island
CpG β
discovery
rs1565251
rs10838000
rs1425853
rs10501294
cg03447554
rs11037263
43008961
43047022
43085082
43123143
43161203
CpG
Main effect
No effect
Interaction effect
R^2 discovery
Chr11:43007961−43162203
R^2 replication
R^2 full model
R^2 main effects only
0
R^2
1
0.021
cg03447554

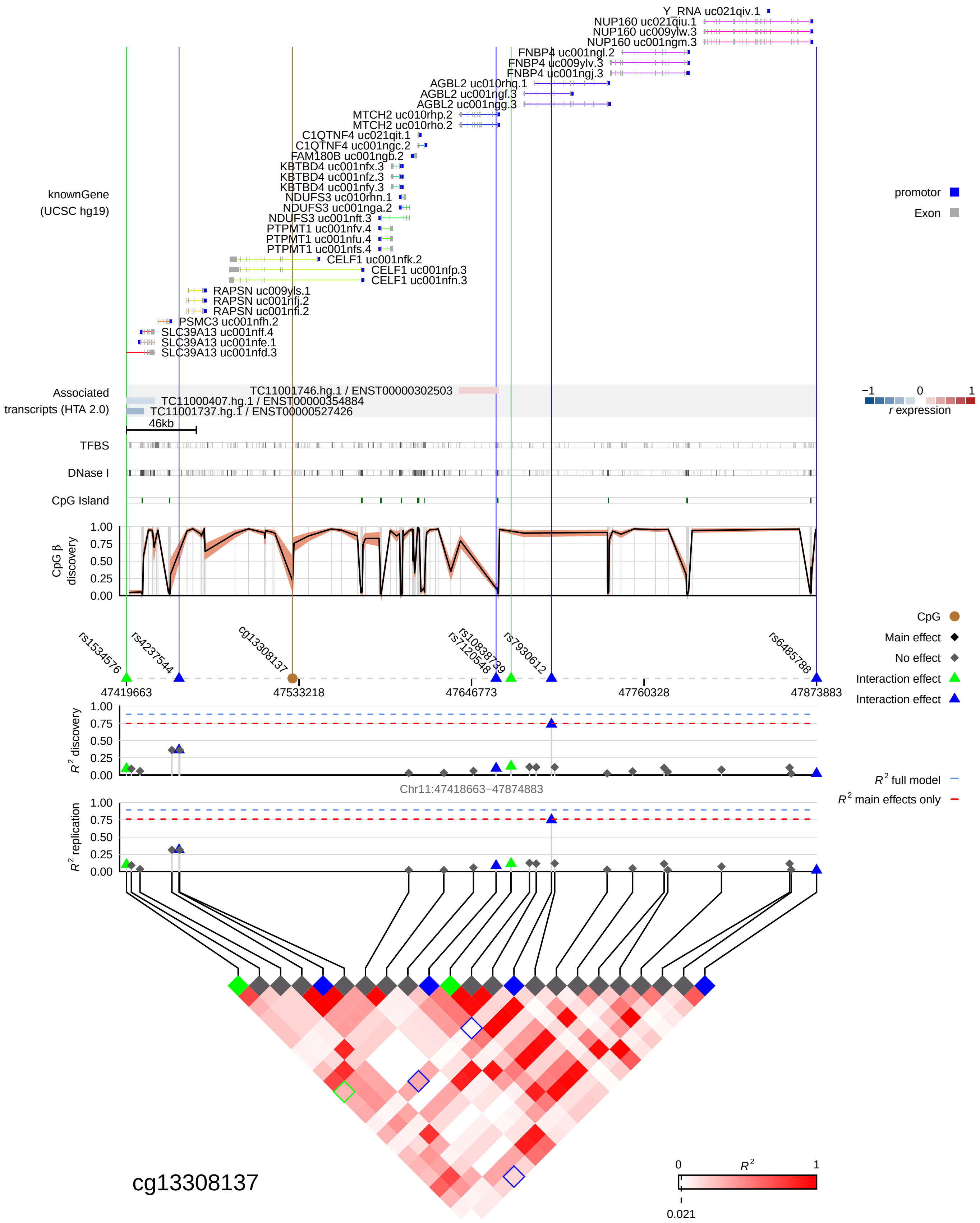

knownGene
(UCSC hg19)
promotor
Exon
Associated
transcripts (HTA 2.0)
r expression
TFBS
DNase I
CpG Island
CpG β
discovery
CpG
Main effect
No effect
Interaction effect
Interaction effect
rs1534576
rs4237544
cg13308137
rs10838739
rs7120548
rs7930612
rs6485788
47419663
47533218
47646773
47760328
47873883
R² discovery
Chr11:47418663−47874883
R² full model
R² main effects only
R² replication
cg13308137
0.021

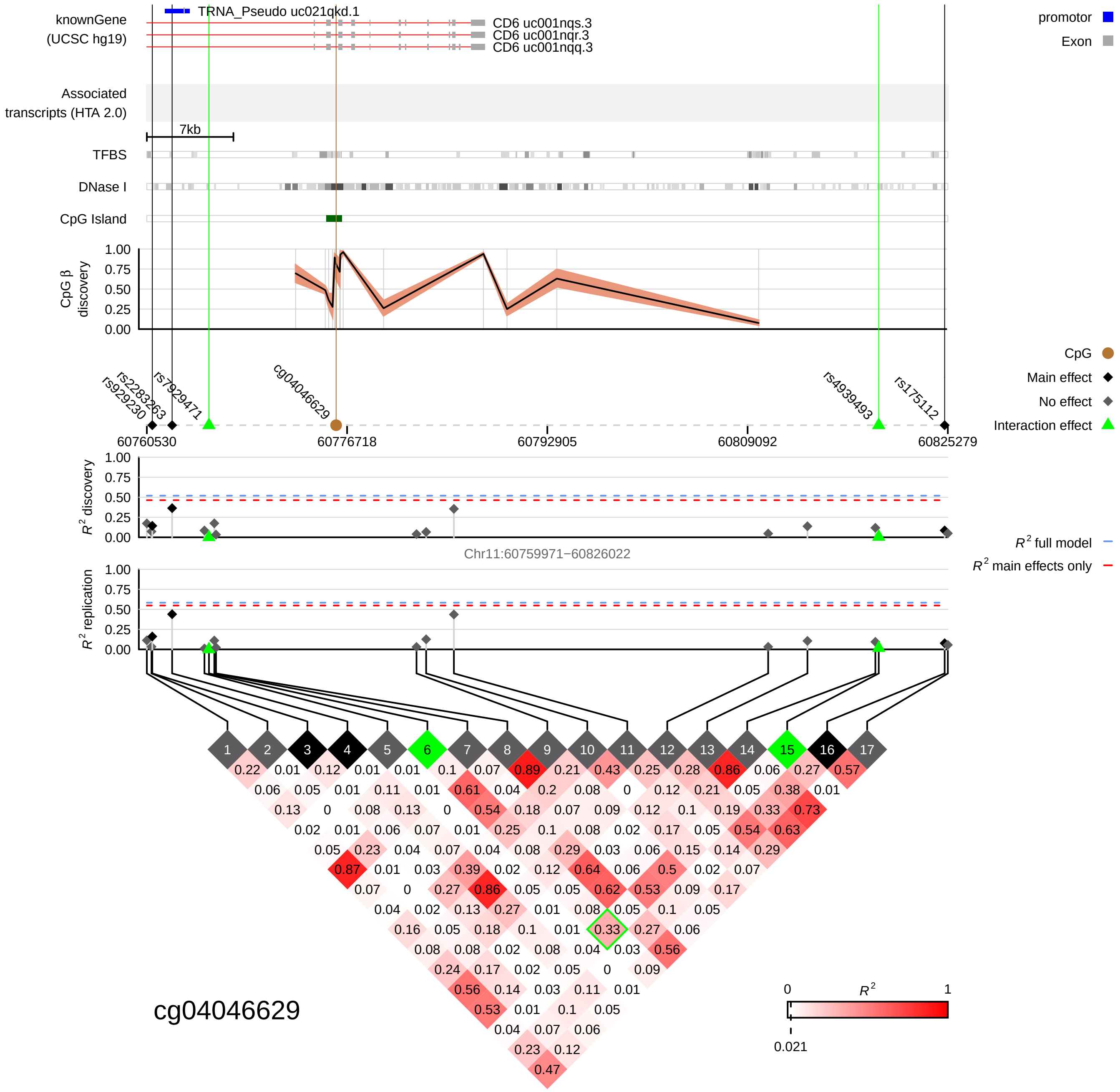

knownGene (UCSC hg19)
TRNA_Pseudo uc021qkd.1
CD6 uc001nqs.3
CD6 uc001nqr.3
CD6 uc001nqq.3
promotor
Exon
Associated transcripts (HTA 2.0)
7kb
TFBS
DNase I
CpG Island
CpG β discovery
rs929230
rs2283263
rs7929471
cg04046629
rs4939493
rs175112
CpG
Main effect
No effect
Interaction effect
60760530
60776718
60792905
60809092
60825279
R^2 discovery
Chr11:60759971−60826022
R^2 full model
R^2 main effects only
R^2 replication
R^2
0.021
cg04046629

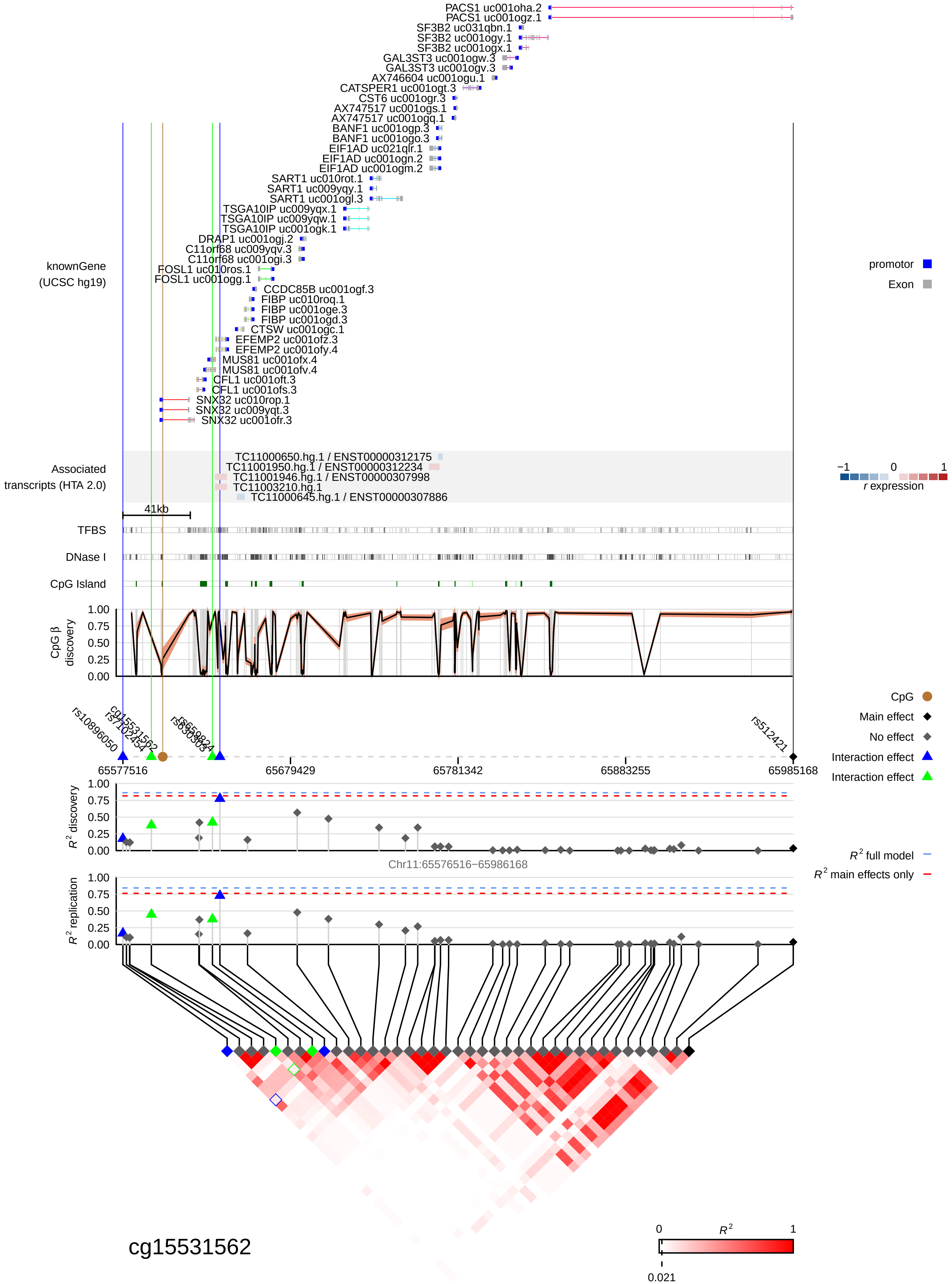

knownGene (UCSC hg19)
Associated transcripts (HTA 2.0)
TFBS
DNase I
CpG Island
CpG β discovery
promotor
Exon
r expression
41kb
CpG
Main effect
No effect
Interaction effect
Interaction effect
rs10896050
cg15531562
rs7102454
rs659824
rs630303
rs512421
65577516
65679429
65781342
65883255
65985168
R^2 discovery
Chr11:65576516−65986168
R^2 replication
R^2 full model
R^2 main effects only
R^2
0.021
cg15531562

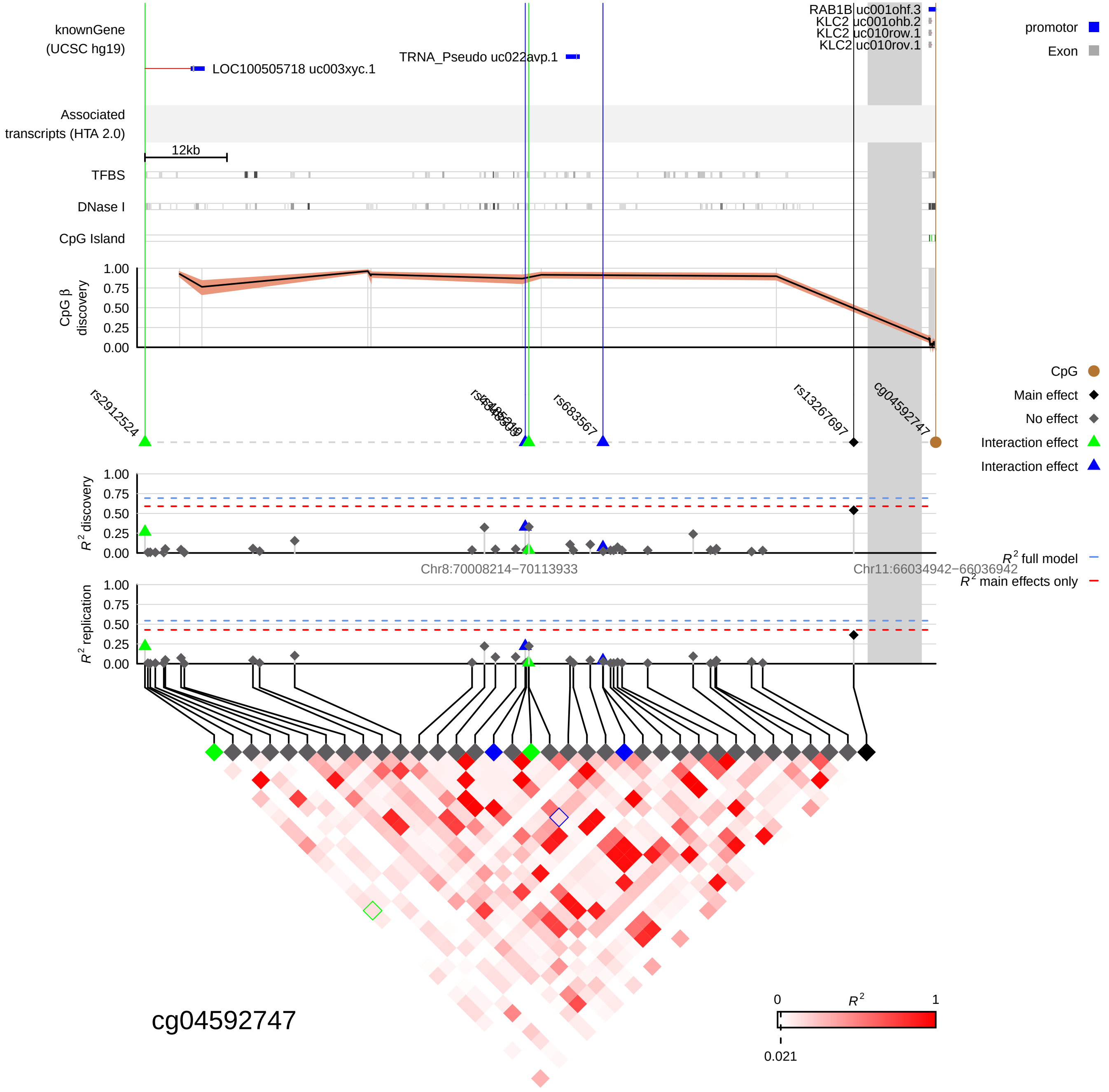

knownGene
(UCSC hg19)
LOC100505718 uc003xyc.1
TRNA_Pseudo uc022avp.1
RAB1B uc001ohf.3
KLC2 uc001ohb.2
KLC2 uc010row.1
KLC2 uc010rov.1
promotor
Exon
Associated
transcripts (HTA 2.0)
12kb
TFBS
DNase I
CpG Island
CpG β discovery
rs2912524
rs6683567
rs13267697
cg04592747
CpG
Main effect
No effect
Interaction effect
Interaction effect
R² discovery
Chr8:70008214−70113933
Chr11:66034942−66036942
R² full model
R² main effects only
R² replication
cg04592747
0
R²
1
0.021

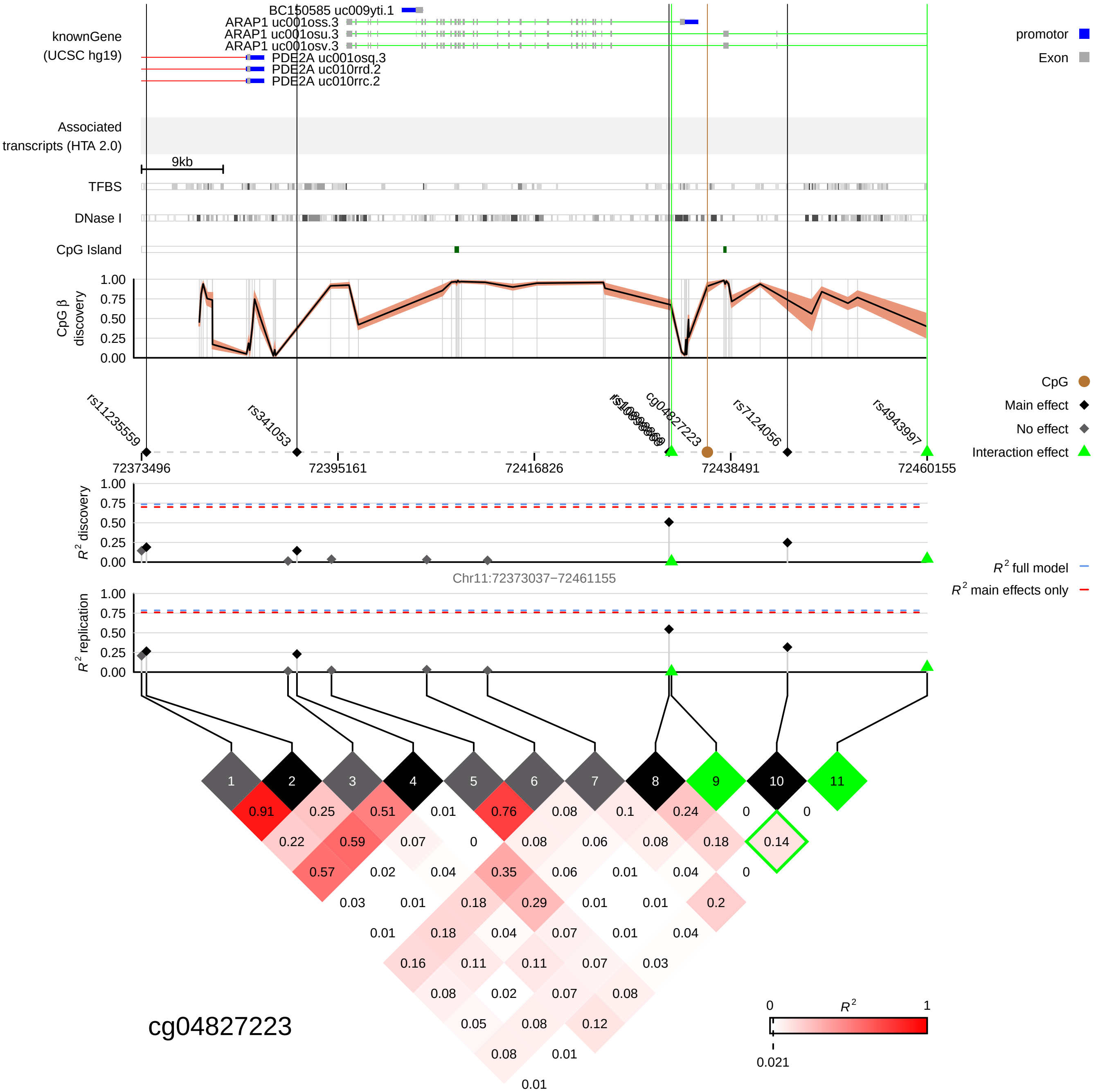

knownGene
(UCSC hg19)
BC150585 uc009yti.1
ARAP1 uc001oss.3
ARAP1 uc001osu.3
ARAP1 uc001osv.3
PDE2A uc001osq.3
PDE2A uc010rrd.2
PDE2A uc010rrc.2
promotor
Exon
Associated
transcripts (HTA 2.0)
9kb
TFBS
DNase I
CpG Island
CpG β
discovery
1.00
0.75
0.50
0.25
0.00
rs11235559
rs341053
cg04827223
rs7124056
rs4943997
CpG
Main effect
No effect
Interaction effect
72373496
72395161
72416826
72438491
72460155
R^2 discovery
Chr11:72373037−72461155
R^2 full model
R^2 main effects only
R^2 replication
1
2
3
4
5
6
7
8
9
10
11
0.91
0.25
0.51
0.01
0.76
0.08
0.1
0.24
0
0
0.22
0.59
0.07
0
0.08
0.06
0.08
0.18
0.14
0.57
0.02
0.04
0.35
0.06
0.01
0.04
0
0.03
0.01
0.18
0.29
0.01
0.01
0.2
0.01
0.18
0.04
0.07
0.01
0.04
0.16
0.11
0.11
0.07
0.03
0.08
0.02
0.07
0.08
0.05
0.08
0.12
0.08
0.01
0.01
cg04827223
0
R^2
1
0.021

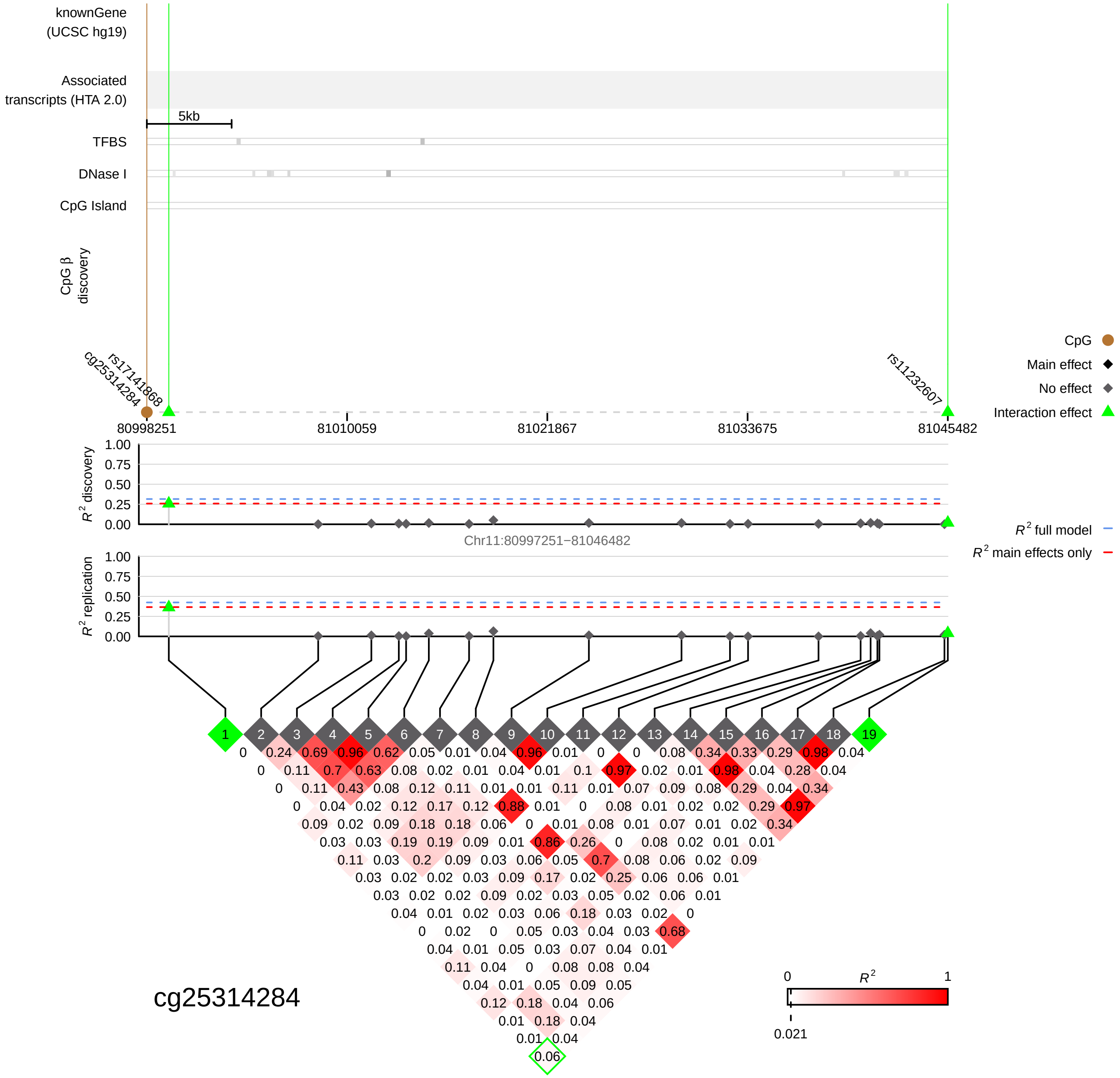
knownGene
(UCSC hg19)
Associated
transcripts (HTA 2.0)
5kb
TFBS
DNase I
CpG Island
CpG β
discovery
cg25314284
rs17141868
rs11232607
80998251
81010059
81021867
81033675
81045482
CpG
Main effect
No effect
Interaction effect
R^2 discovery
1.00
0.75
0.50
0.25
0.00
Chr11:80997251−81046482
R^2 full model
R^2 main effects only
R^2 replication
1
2
3
4
5
6
7
8
9
10
11
12
13
14
15
16
17
18
19
cg25314284
0
R^2
1
0.021

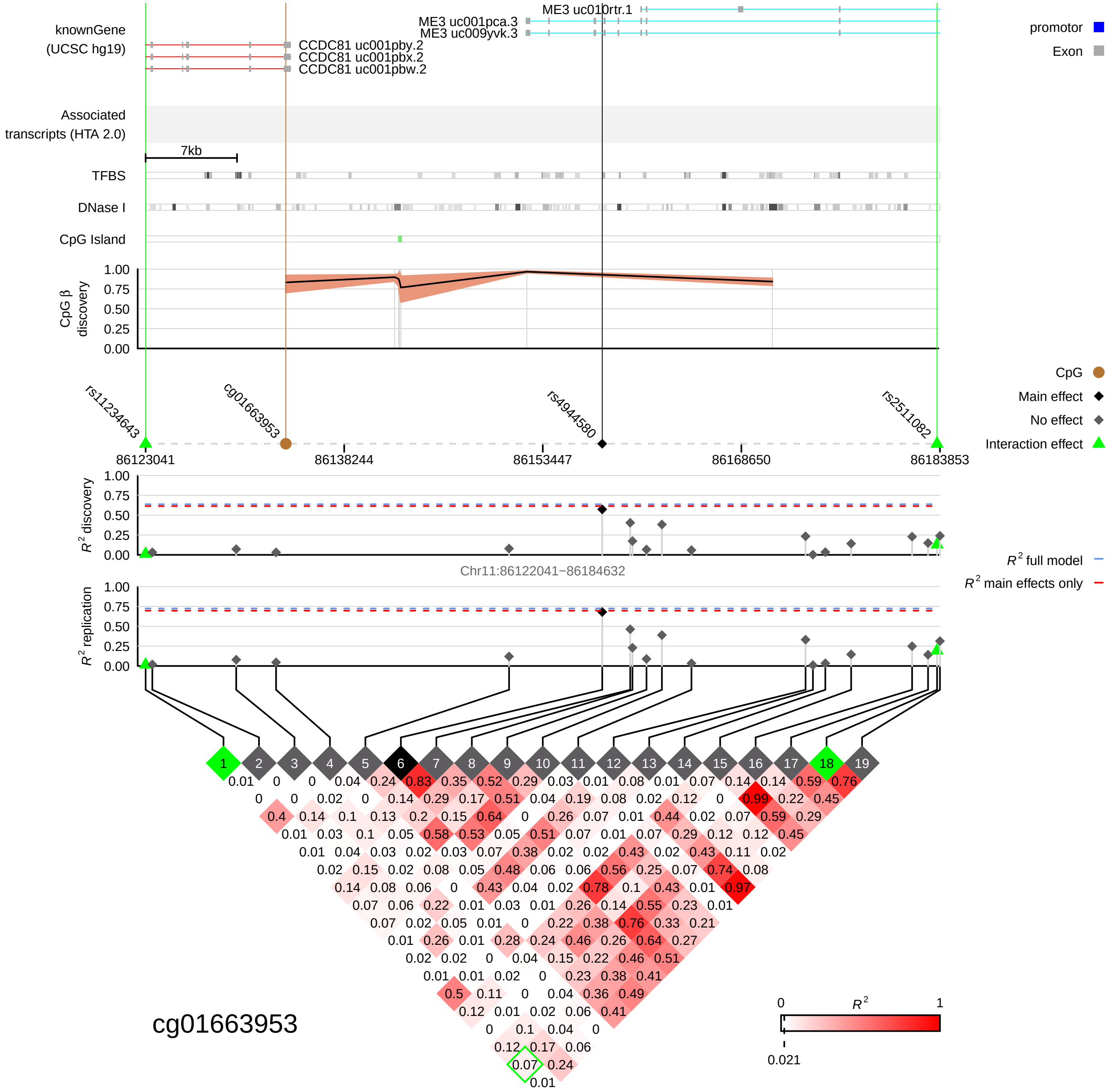
knownGene (UCSC hg19)
ME3 uc010rtr.1
ME3 uc001pca.3
ME3 uc009yvk.3
CCDC81 uc001pby.2
CCDC81 uc001pbx.2
CCDC81 uc001pbw.2
promotor
Exon
Associated transcripts (HTA 2.0)
7kb
TFBS
DNase I
CpG Island
CpG β discovery
rs11234643
cg01663953
rs4944580
rs2511082
86123041
86138244
86153447
86168650
86183853
CpG
Main effect
No effect
Interaction effect
R^2 discovery
Chr11:86122041−86184632
R^2 full model
R^2 main effects only
R^2 replication
R^2
0.021
cg01663953

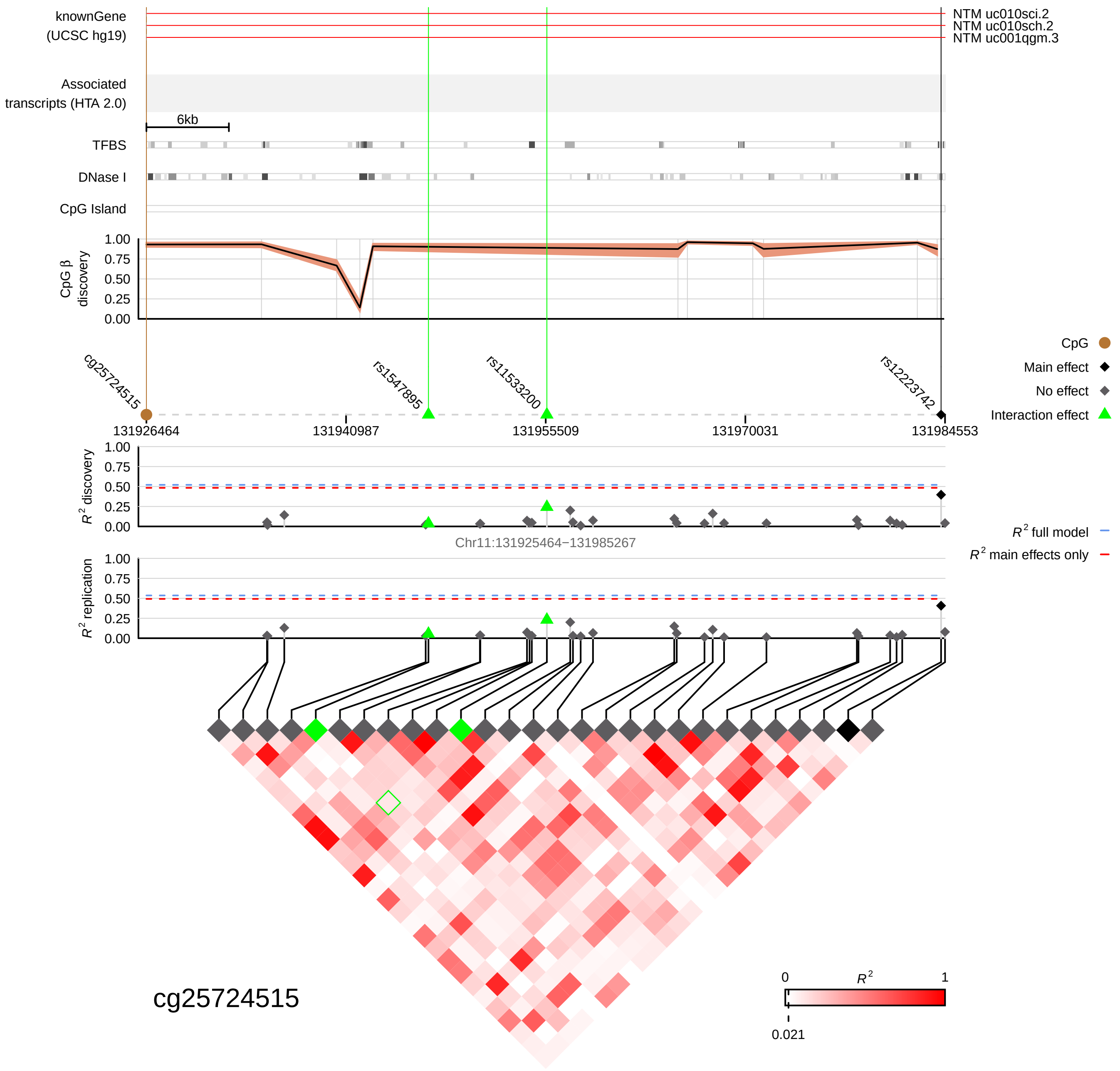

knownGene (UCSC hg19)
NTM uc010sci.2
NTM uc010sch.2
NTM uc001qgm.3
Associated transcripts (HTA 2.0)
6kb
TFBS
DNase I
CpG Island
CpG β discovery
1.00
0.75
0.50
0.25
0.00
cg25724515
rs1547895
rs11533200
rs12223742
131926464
131940987
131955509
131970031
131984553
CpG
Main effect
No effect
Interaction effect
R² discovery
Chr11:131925464−131985267
R² replication
R² full model
R² main effects only
0
R²
1
0.021

cg25724515

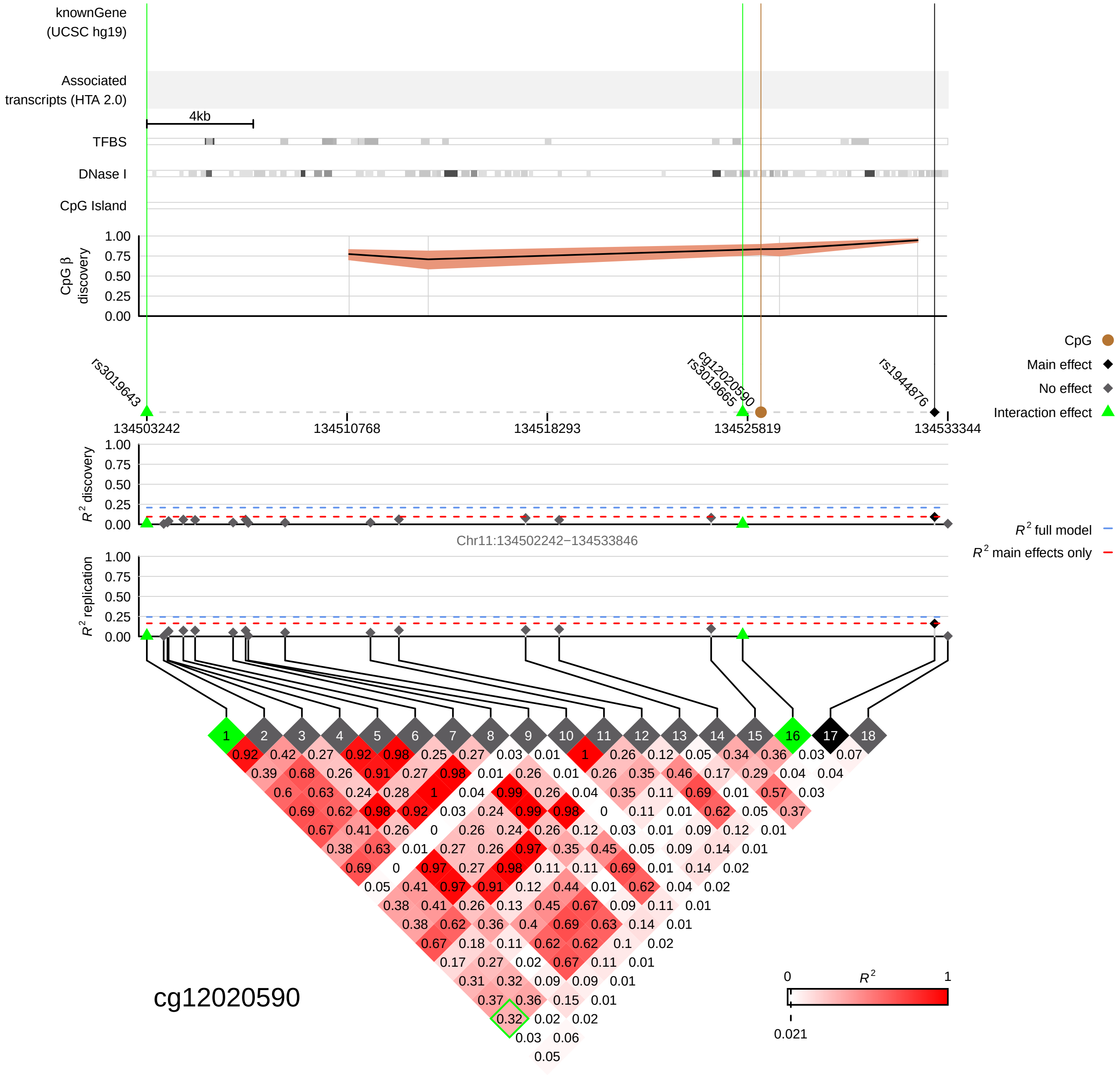

knownGene
(UCSC hg19)
Associated
transcripts (HTA 2.0)
4kb
TFBS
DNase I
CpG Island
CpG β
discovery
1.00
0.75
0.50
0.25
0.00
rs3019643
cg12020590
rs3019665
rs1944876
134503242
134510768
134518293
134525819
134533344
CpG
Main effect
No effect
Interaction effect
R^2 discovery
Chr11:134502242−134533846
R^2 full model
R^2 main effects only
R^2 replication
1
2
3
4
5
6
7
8
9
10
11
12
13
14
15
16
17
18
cg12020590
0
R^2
1
0.021

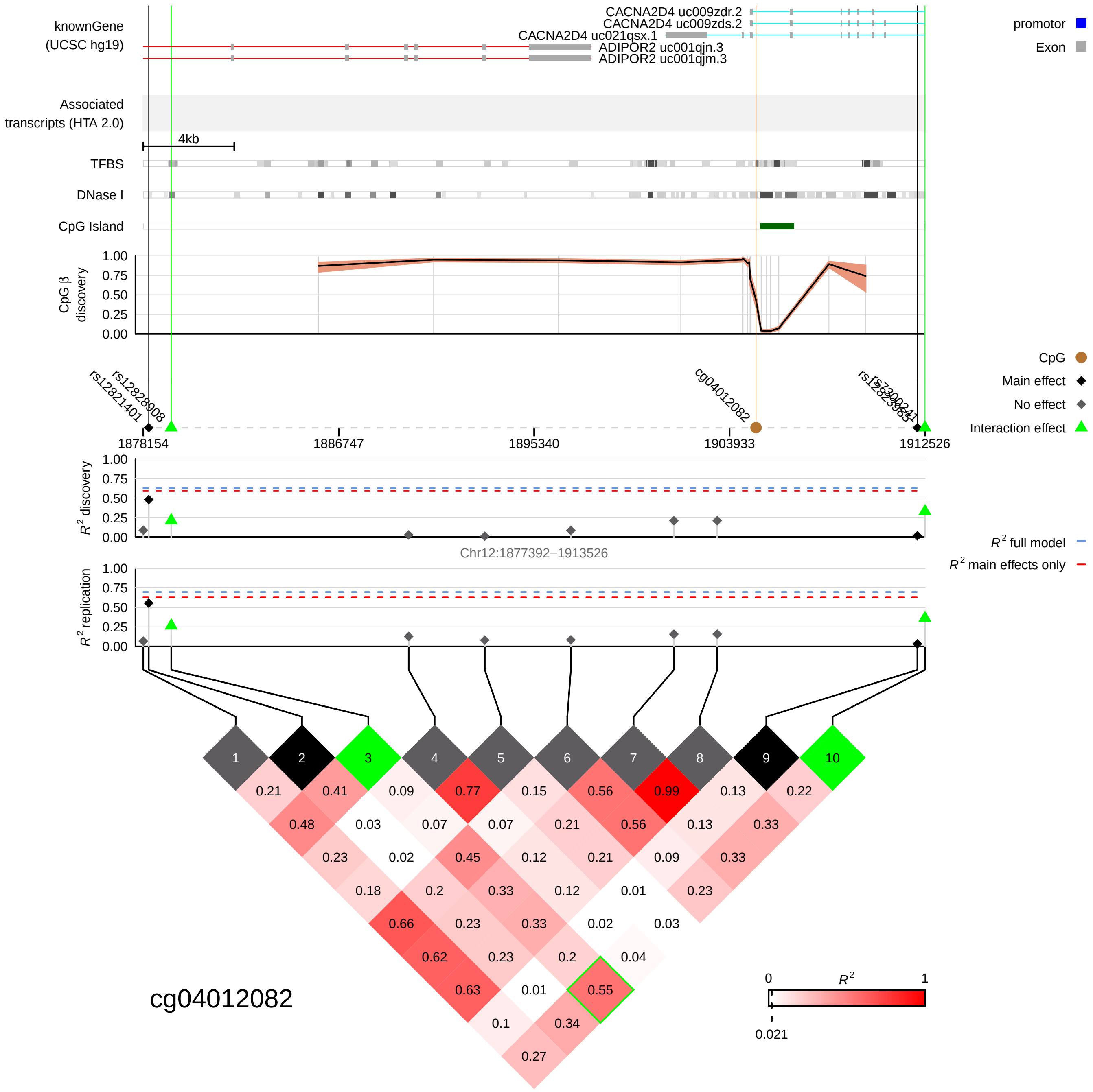

knownGene
(UCSC hg19)
CACNA2D4 uc009zdr.2
CACNA2D4 uc009zds.2
CACNA2D4 uc021qsx.1
ADIPOR2 uc001qjn.3
ADIPOR2 uc001qjm.3
promotor
Exon
Associated
transcripts (HTA 2.0)
4kb
TFBS
DNase I
CpG Island
CpG β
discovery
cg04012082
1878154
1886747
1895340
1903933
1912526
CpG
Main effect
No effect
Interaction effect
R² discovery
Chr12:1877392−1913526
R² full model
R² main effects only
R² replication
0.021
cg04012082

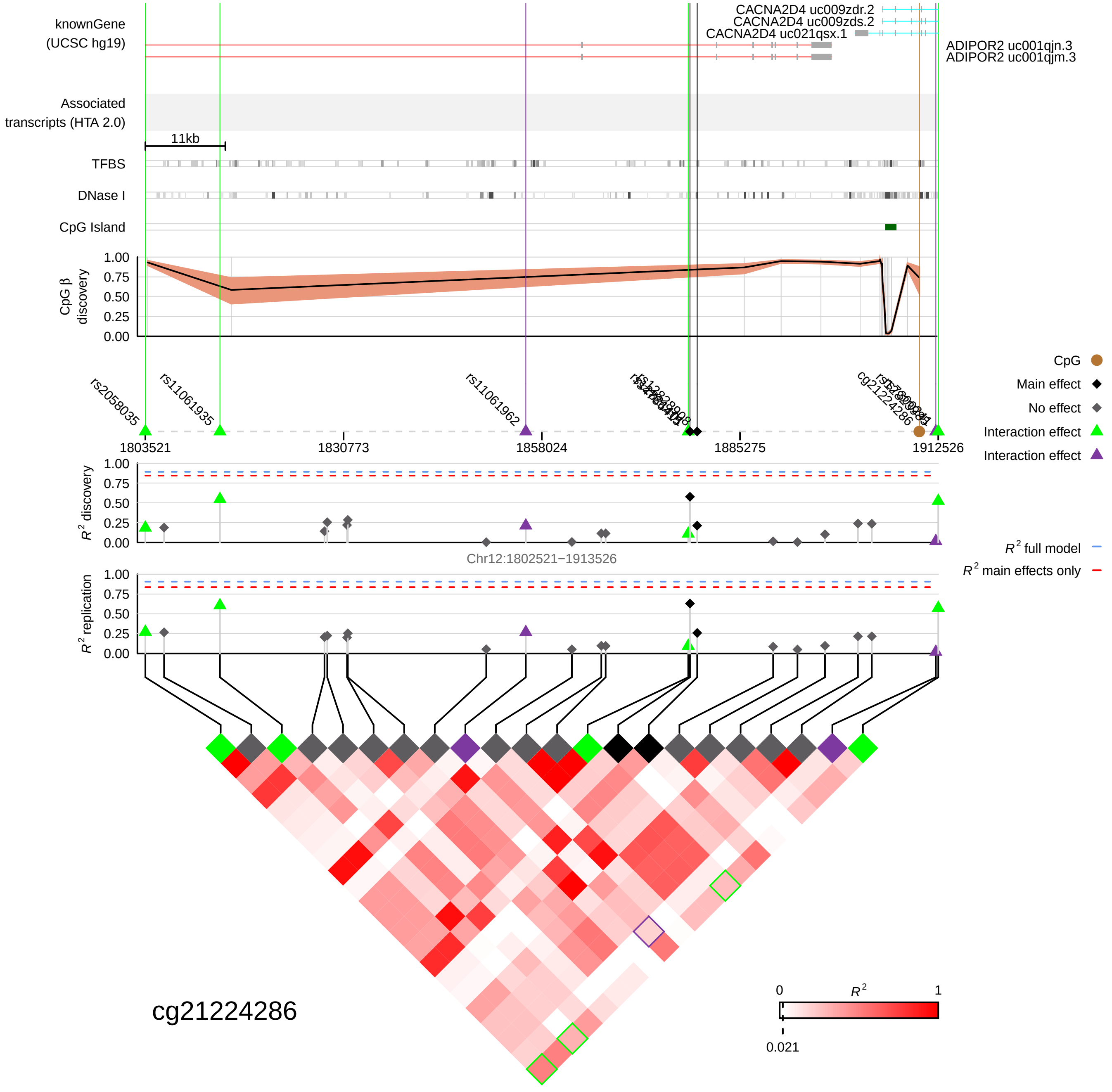

knownGene (UCSC hg19)
CACNA2D4 uc009zdr.2
CACNA2D4 uc009zds.2
CACNA2D4 uc021qsx.1
ADIPOR2 uc001qjn.3
ADIPOR2 uc001qjm.3
Associated transcripts (HTA 2.0)
11kb
TFBS
DNase I
CpG Island
CpG β discovery
rs2058035
rs11061935
rs11061962
cg21224286
1803521
1830773
1858024
1885275
1912526
CpG
Main effect
No effect
Interaction effect
Interaction effect
R² discovery
Chr12:1802521−1913526
R² full model
R² main effects only
R² replication
0
R²
1
0.021
cg21224286

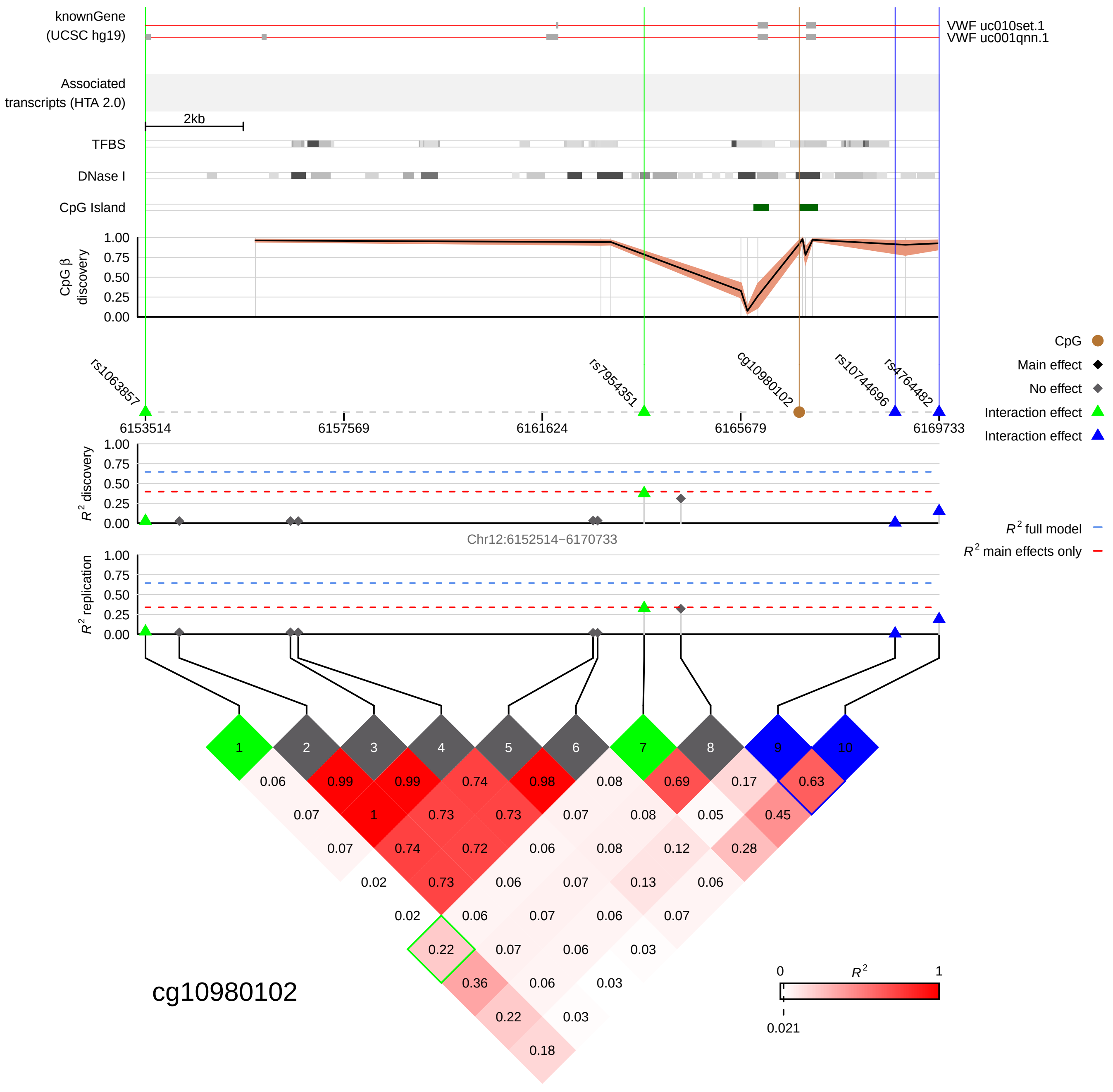

knownGene (UCSC hg19)
VWF uc010set.1
VWF uc001qnn.1
Associated transcripts (HTA 2.0)
2kb
TFBS
DNase I
CpG Island
CpG β discovery
rs1063857
rs7954351
cg10980102
rs10744696
rs4764482
6153514
6157569
6161624
6165679
6169733
CpG
Main effect
No effect
Interaction effect
Interaction effect
R² discovery
Chr12:6152514−6170733
R² full model
R² main effects only
R² replication
cg10980102
R²
0
1
0.021

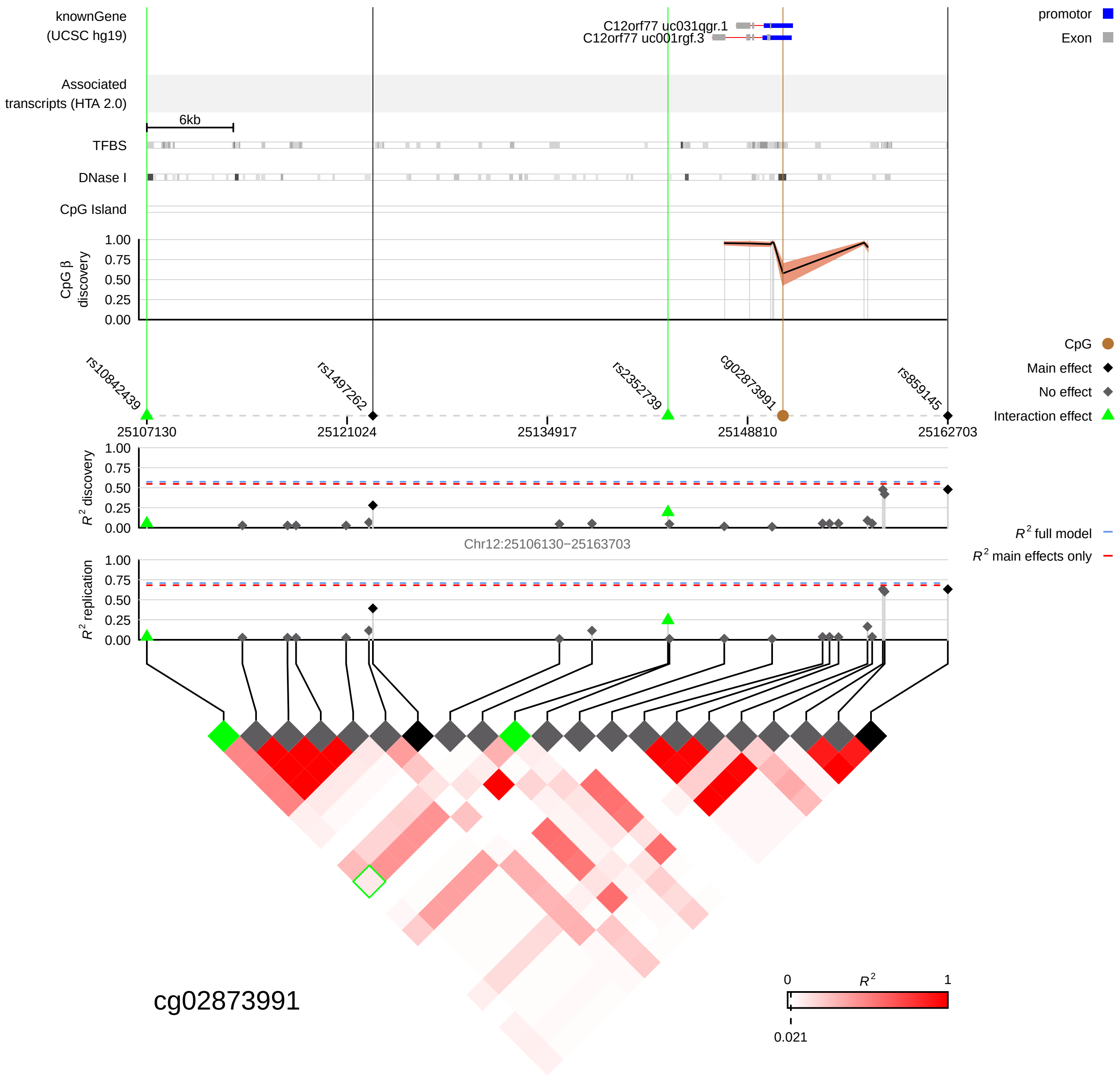
knownGene (UCSC hg19)
C12orf77 uc031qgr.1
C12orf77 uc001rgf.3
promotor
Exon
Associated transcripts (HTA 2.0)
6kb
TFBS
DNase I
CpG Island
CpG β discovery
1.00
0.75
0.50
0.25
0.00
rs10842439
rs1497262
rs2352739
cg02873991
rs859145
CpG
Main effect
No effect
Interaction effect
25107130
25121024
25134917
25148810
25162703
R^2 discovery
Chr12:25106130−25163703
R^2 full model
R^2 main effects only
R^2 replication
cg02873991
0
R^2
1
0.021

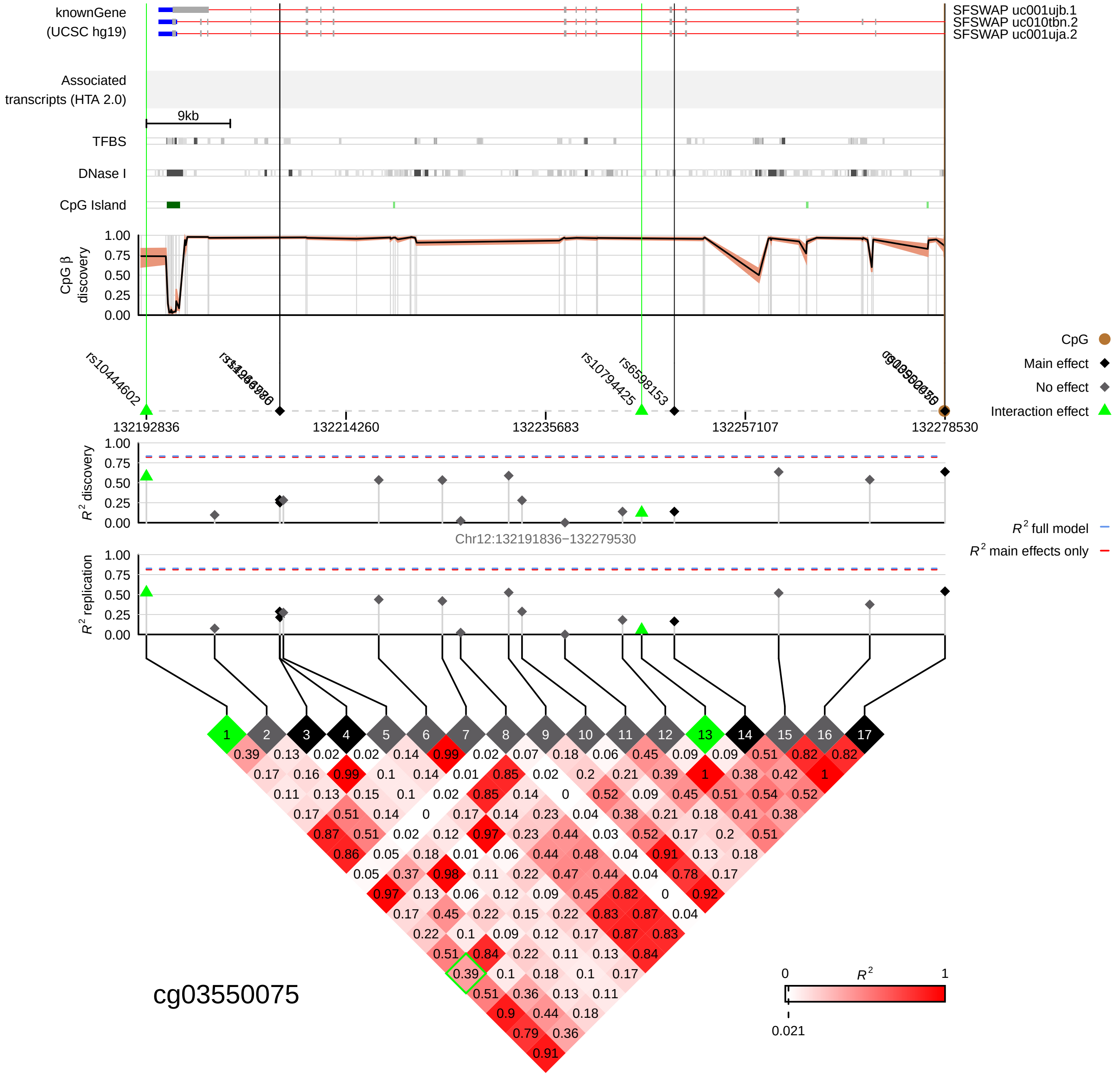

knownGene (UCSC hg19)
SFSWAP uc001ujb.1
SFSWAP uc010tbn.2
SFSWAP uc001uja.2
Associated transcripts (HTA 2.0)
9kb
TFBS
DNase I
CpG Island
CpG β discovery
rs10444602
rs10794425
rs6598153
132192836
132214260
132235683
132257107
132278530
CpG
Main effect
No effect
Interaction effect
R^2 discovery
Chr12:132191836−132279530
R^2 full model
R^2 main effects only
R^2 replication
cg03550075
R^2
0.021

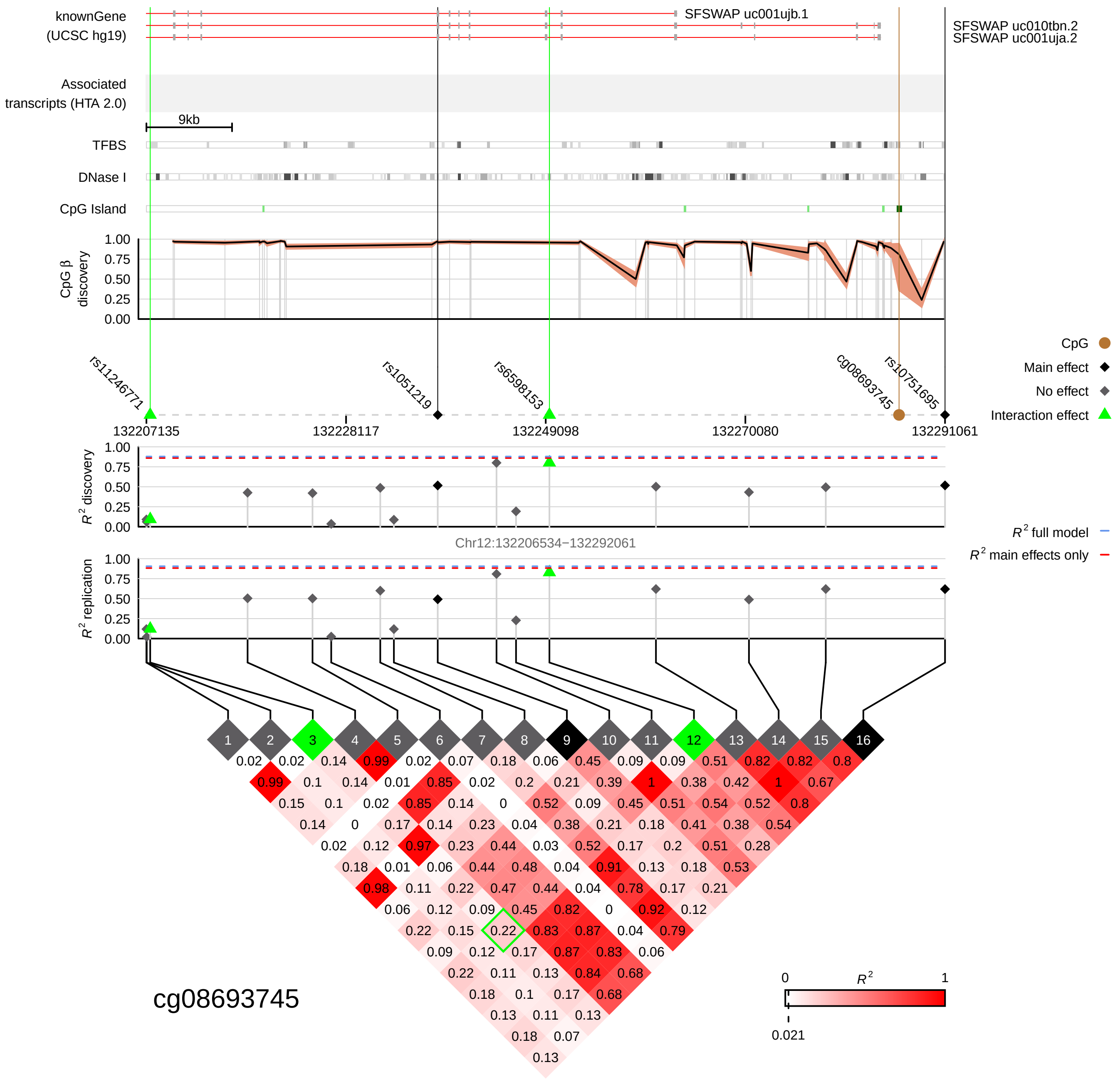

knownGene
(UCSC hg19)
SFSWAP uc001ujb.1
SFSWAP uc010tbn.2
SFSWAP uc001uja.2
Associated transcripts (HTA 2.0)
9kb
TFBS
DNase I
CpG Island
CpG β discovery
rs11246771
rs1051219
rs6598153
cg08693745
rs10751695
132207135
132228117
132249098
132270080
132291061
CpG
Main effect
No effect
Interaction effect
R^2 discovery
Chr12:132206534−132292061
R^2 replication
R^2 full model
R^2 main effects only
cg08693745
R^2
0.021

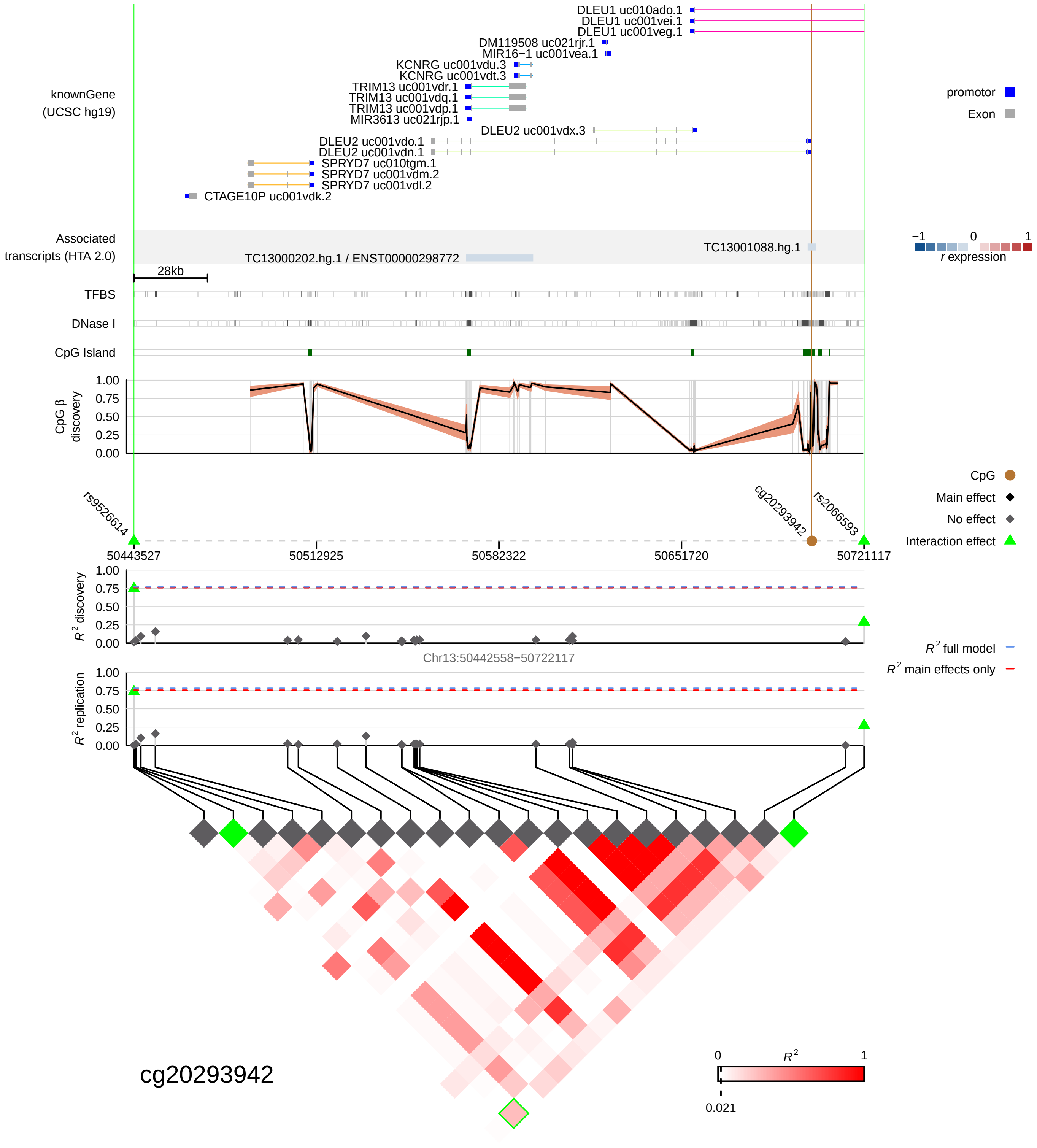

DLEU1 uc010ado.1
DLEU1 uc001vei.1
DLEU1 uc001veg.1
DM119508 uc021rjr.1
MIR16−1 uc001vea.1
KCNRG uc001vdu.3
KCNRG uc001vdt.3
TRIM13 uc001vdr.1
TRIM13 uc001vdq.1
TRIM13 uc001vdp.1
MIR3613 uc021rjp.1
DLEU2 uc001vdx.3
DLEU2 uc001vdo.1
DLEU2 uc001vdn.1
SPRYD7 uc010tgm.1
SPRYD7 uc001vdm.2
SPRYD7 uc001vdl.2
CTAGE10P uc001vdk.2
knownGene (UCSC hg19)
promotor
Exon
Associated transcripts (HTA 2.0)
TC13001088.hg.1
TC13000202.hg.1 / ENST00000298772
−1 0 1
r expression
28kb
TFBS
DNase I
CpG Island
CpG β discovery
1.00
0.75
0.50
0.25
0.00
rs9526614
cg20293942
rs2066593
CpG
Main effect
No effect
Interaction effect
50443527
50512925
50582322
50651720
50721117
R^2 discovery
Chr13:50442558−50722117
R^2 full model
R^2 main effects only
R^2 replication
0
R^2
1
0.021
cg20293942

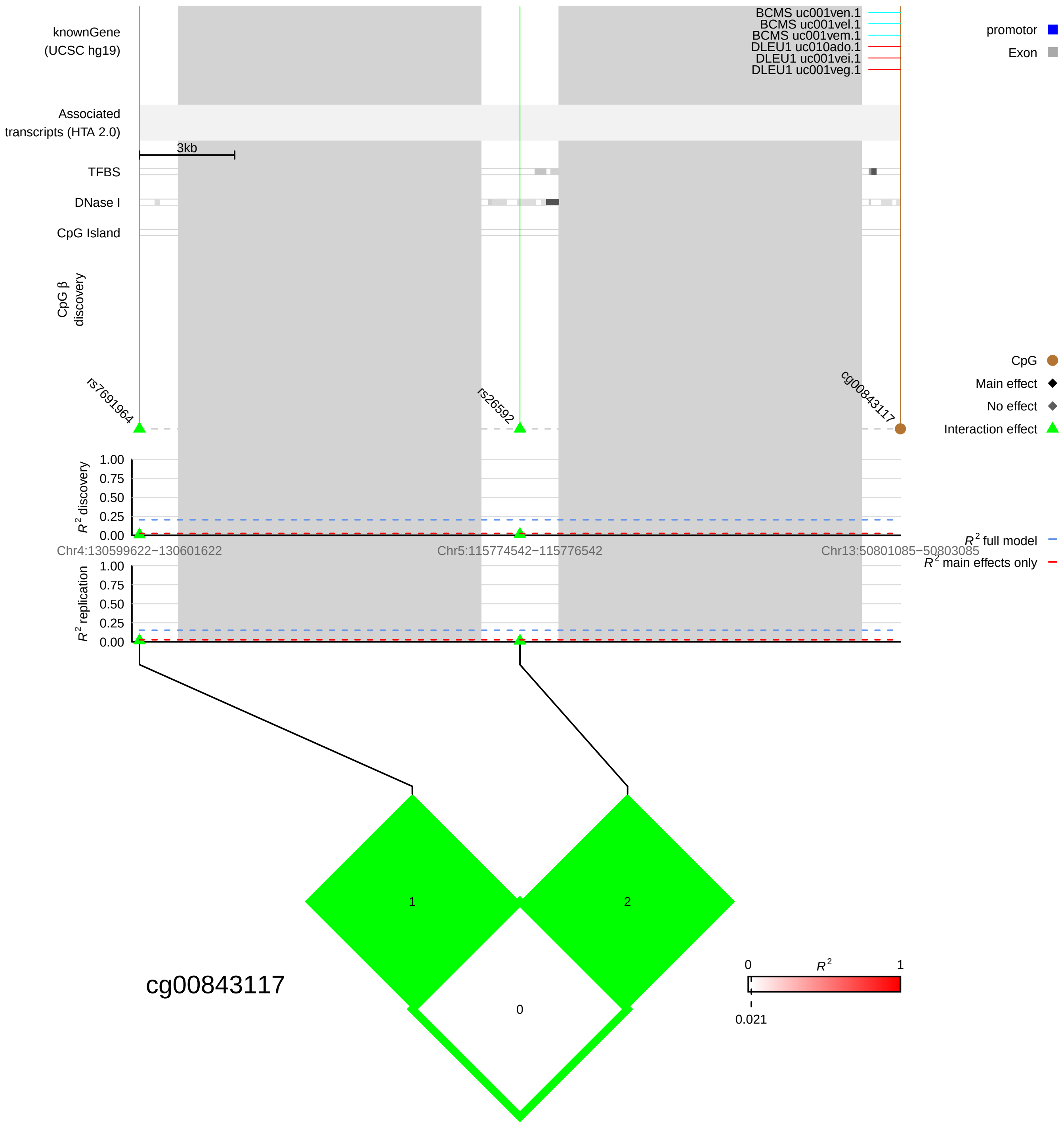

knownGene
(UCSC hg19)
Associated
transcripts (HTA 2.0)
3kb
TFBS
DNase I
CpG Island
CpG β
discovery
BCMS uc001ven.1
BCMS uc001vel.1
BCMS uc001vem.1
DLEU1 uc010ado.1
DLEU1 uc001vei.1
DLEU1 uc001veg.1
promotor
Exon
rs7691964
rs26592
cg00843117
CpG
Main effect
No effect
Interaction effect
R^2 discovery
1.00
0.75
0.50
0.25
0.00
Chr4:130599622−130601622
Chr5:115774542−115776542
Chr13:50801085−50803085
R^2 full model
R^2 main effects only
R^2 replication
1.00
0.75
0.50
0.25
0.00
1
2
0
cg00843117
0
R^2
1
0.021

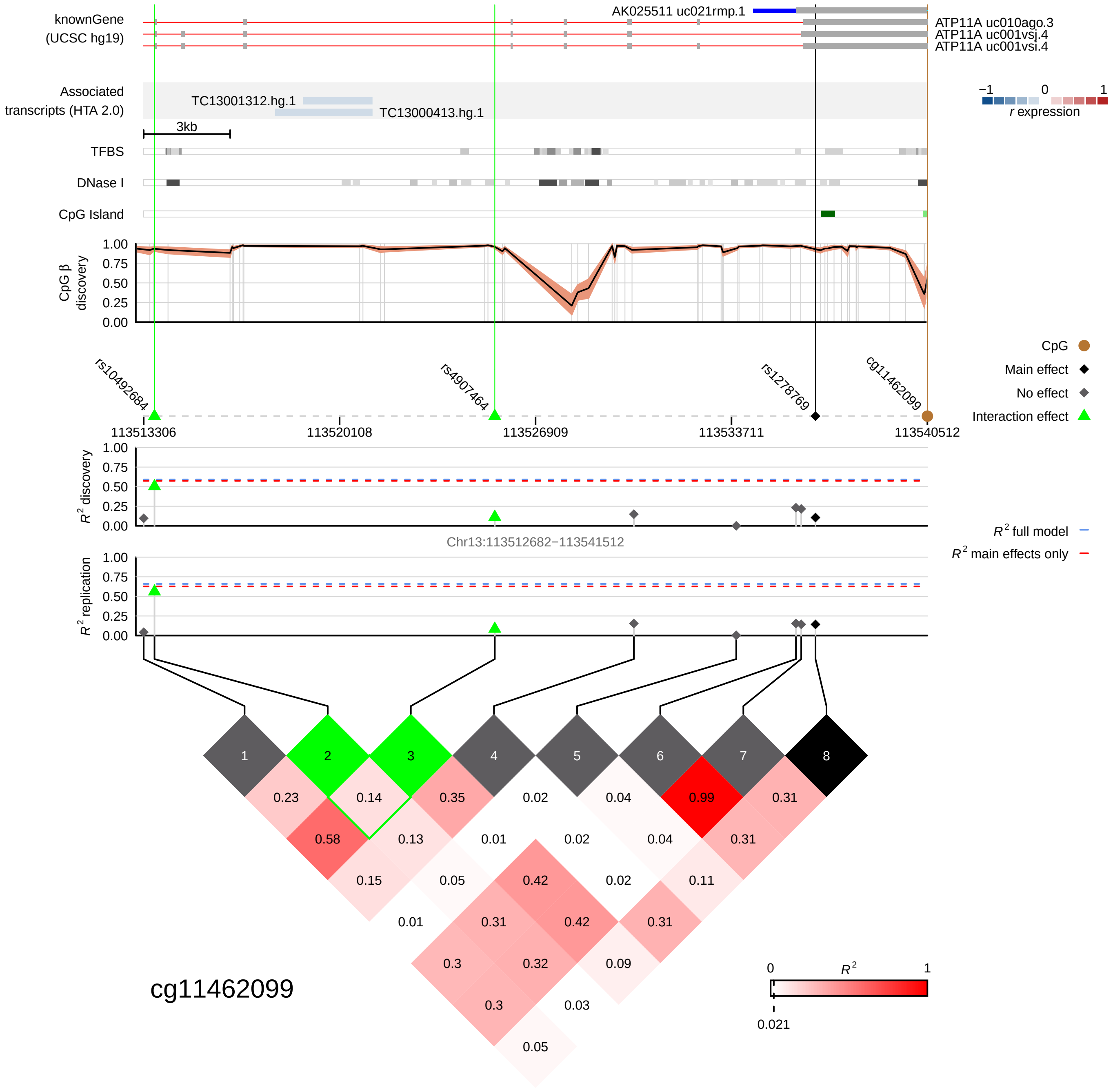

knownGene (UCSC hg19)
AK025511 uc021rmp.1
ATP11A uc010ago.3
ATP11A uc001vsj.4
ATP11A uc001vsi.4
Associated transcripts (HTA 2.0)
TC13001312.hg.1
TC13000413.hg.1
3kb
TFBS
DNase I
CpG Island
CpG β discovery
r expression
CpG
Main effect
No effect
Interaction effect
rs10492684
rs4907464
rs1278769
cg11462099
113513306
113520108
113526909
113533711
113540512
R² discovery
Chr13:113512682−113541512
R² replication
R² full model
R² main effects only
cg11462099
R²
0.021

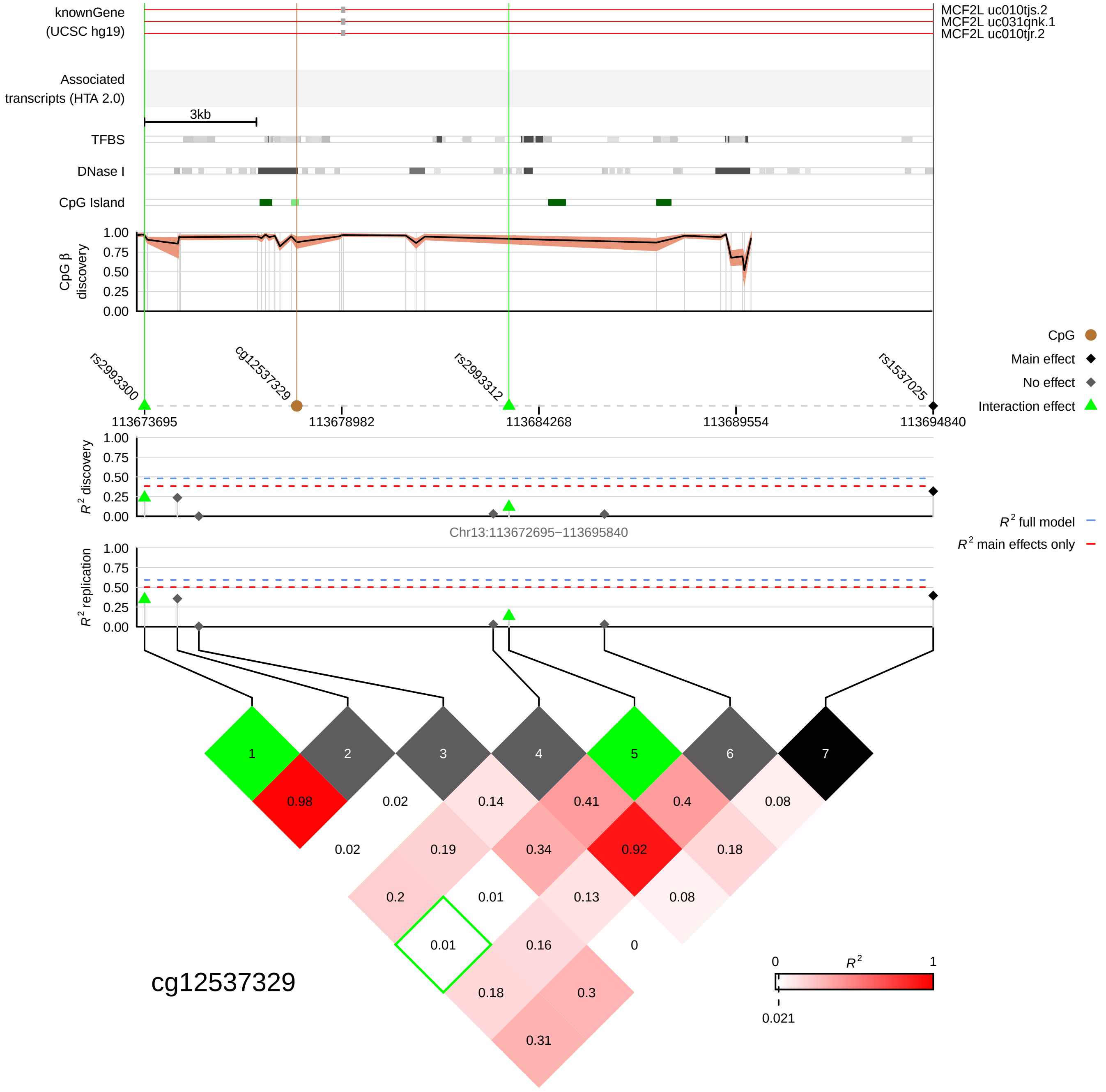
knownGene (UCSC hg19)
MCF2L uc010tjs.2
MCF2L uc031qnk.1
MCF2L uc010tjr.2
Associated transcripts (HTA 2.0)
3kb
TFBS
DNase I
CpG Island
CpG β discovery
rs2993300
cg12537329
rs2993312
rs1537025
113673695
113678982
113684268
113689554
113694840
CpG
Main effect
No effect
Interaction effect
R^2 discovery
Chr13:113672695−113695840
R^2 replication
R^2 full model
R^2 main effects only
cg12537329
0.021

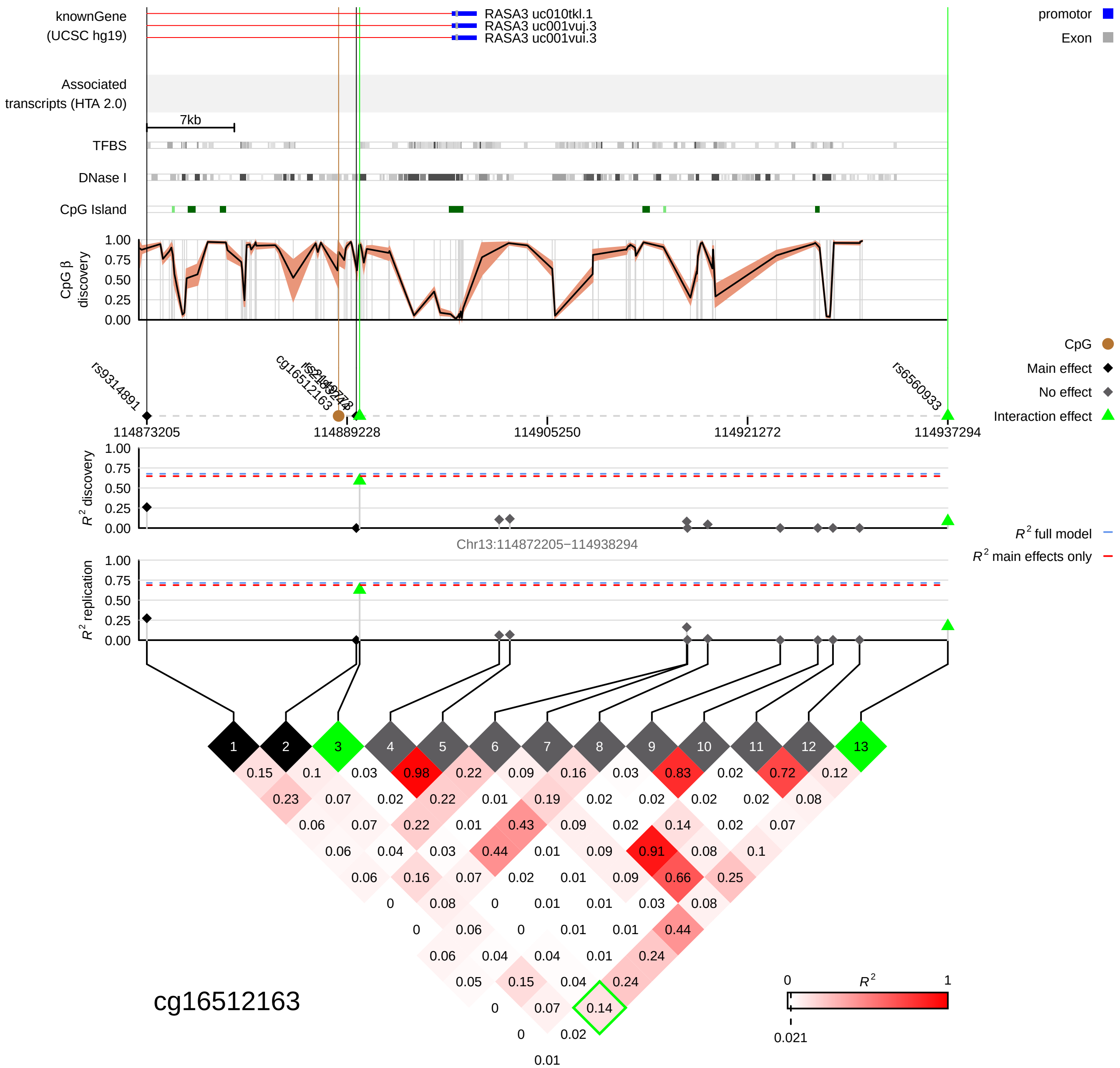
knownGene
(UCSC hg19)
RASA3 uc010tkl.1
RASA3 uc001vuj.3
RASA3 uc001vui.3
promotor
Exon
Associated
transcripts (HTA 2.0)
7kb
TFBS
DNase I
CpG Island
CpG β
discovery
CpG
Main effect
No effect
Interaction effect
rs9314891
cg16512163
rs6560933
114873205
114889228
114905250
114921272
114937294
R^2 discovery
Chr13:114872205−114938294
R^2 full model
R^2 main effects only
R^2 replication
cg16512163
R^2
0.021

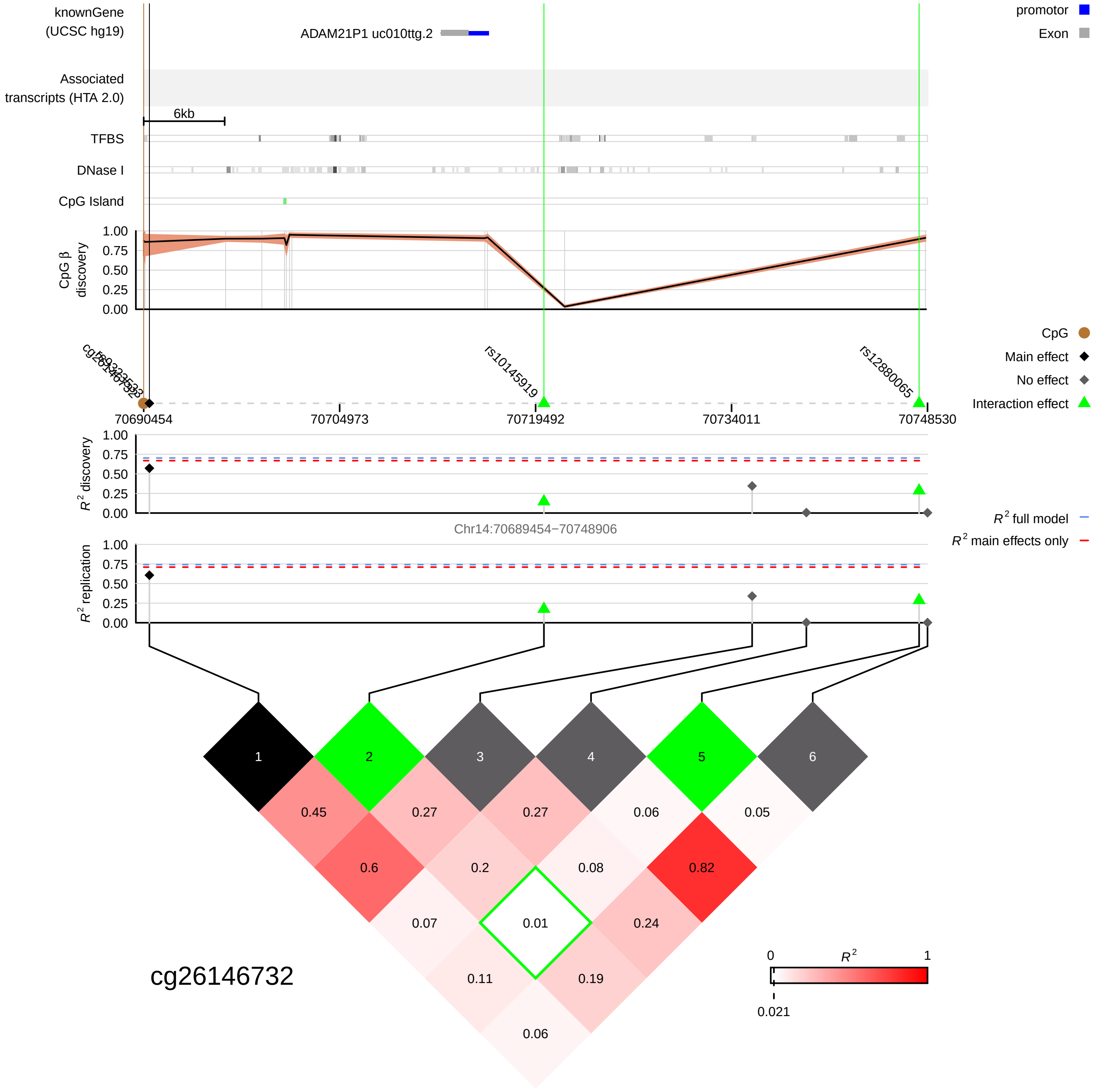
knownGene (UCSC hg19)
ADAM21P1 uc010ttg.2
promotor
Exon
Associated transcripts (HTA 2.0)
6kb
TFBS
DNase I
CpG Island
CpG β discovery
CpG
Main effect
No effect
Interaction effect
rs10145919
rs12880065
70690454
70704973
70719492
70734011
70748530
R² discovery
Chr14:70689454−70748906
R² full model
R² main effects only
R² replication
1
2
3
4
5
6
0.45
0.27
0.27
0.06
0.05
0.6
0.2
0.08
0.82
0.07
0.01
0.24
0.11
0.19
0.06
cg26146732
0
R²
1
0.021

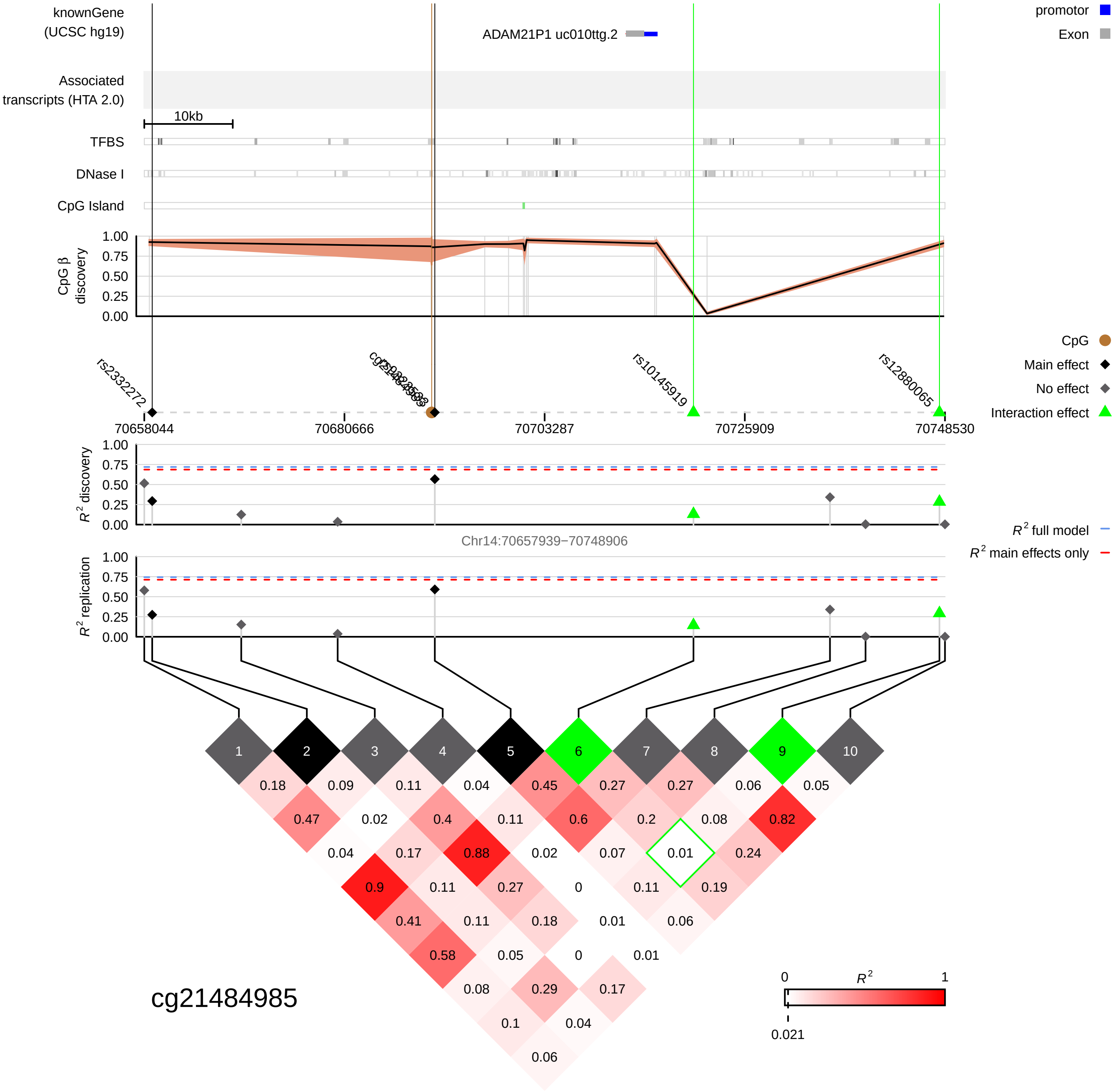
knownGene
(UCSC hg19)
ADAM21P1 uc010ttg.2
promotor
Exon
Associated
transcripts (HTA 2.0)
10kb
TFBS
DNase I
CpG Island
CpG β
discovery
1.00
0.75
0.50
0.25
0.00
rs2332272
rs10145919
rs12880065
CpG
Main effect
No effect
Interaction effect
70658044
70680666
70703287
70725909
70748530
R² discovery
Chr14:70657939−70748906
R² full model
R² main effects only
R² replication
1
2
3
4
5
6
7
8
9
10
cg21484985
0
R²
1
0.021

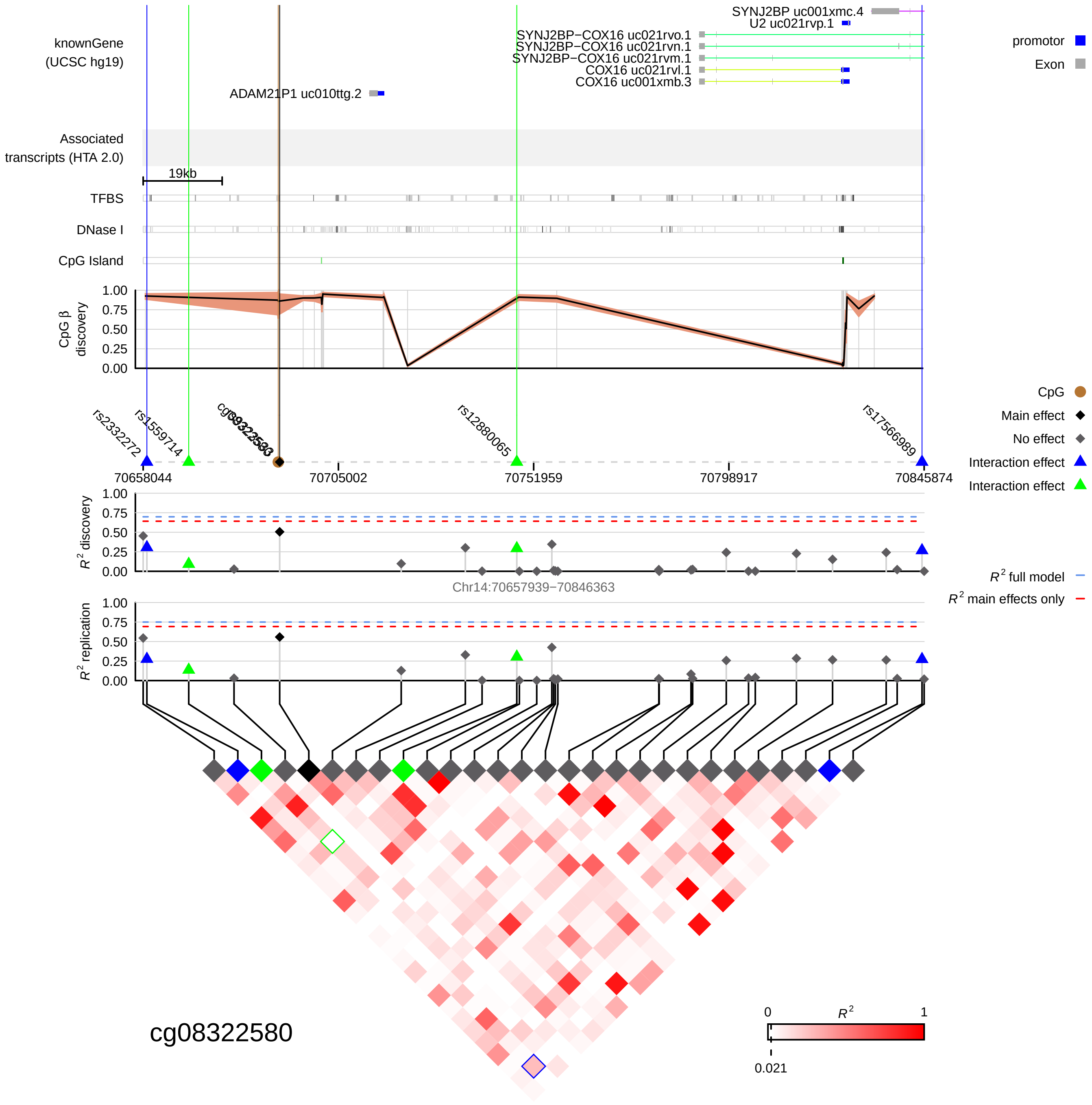

knownGene
(UCSC hg19)
SYNJ2BP uc001xmc.4
U2 uc021rvp.1
SYNJ2BP−COX16 uc021rvo.1
SYNJ2BP−COX16 uc021rvn.1
SYNJ2BP−COX16 uc021rvm.1
COX16 uc021rvl.1
COX16 uc001xmb.3
ADAM21P1 uc010ttg.2
promotor
Exon
Associated transcripts (HTA 2.0)
19kb
TFBS
DNase I
CpG Island
CpG β discovery
1.00
0.75
0.50
0.25
0.00
rs2332272
rs1559714
cg08322580
rs12880065
rs17566989
70658044
70705002
70751959
70798917
70845874
CpG
Main effect
No effect
Interaction effect
Interaction effect
R² discovery
Chr14:70657939−70846363
R² full model
R² main effects only
R² replication
cg08322580
0
R²
1
0.021

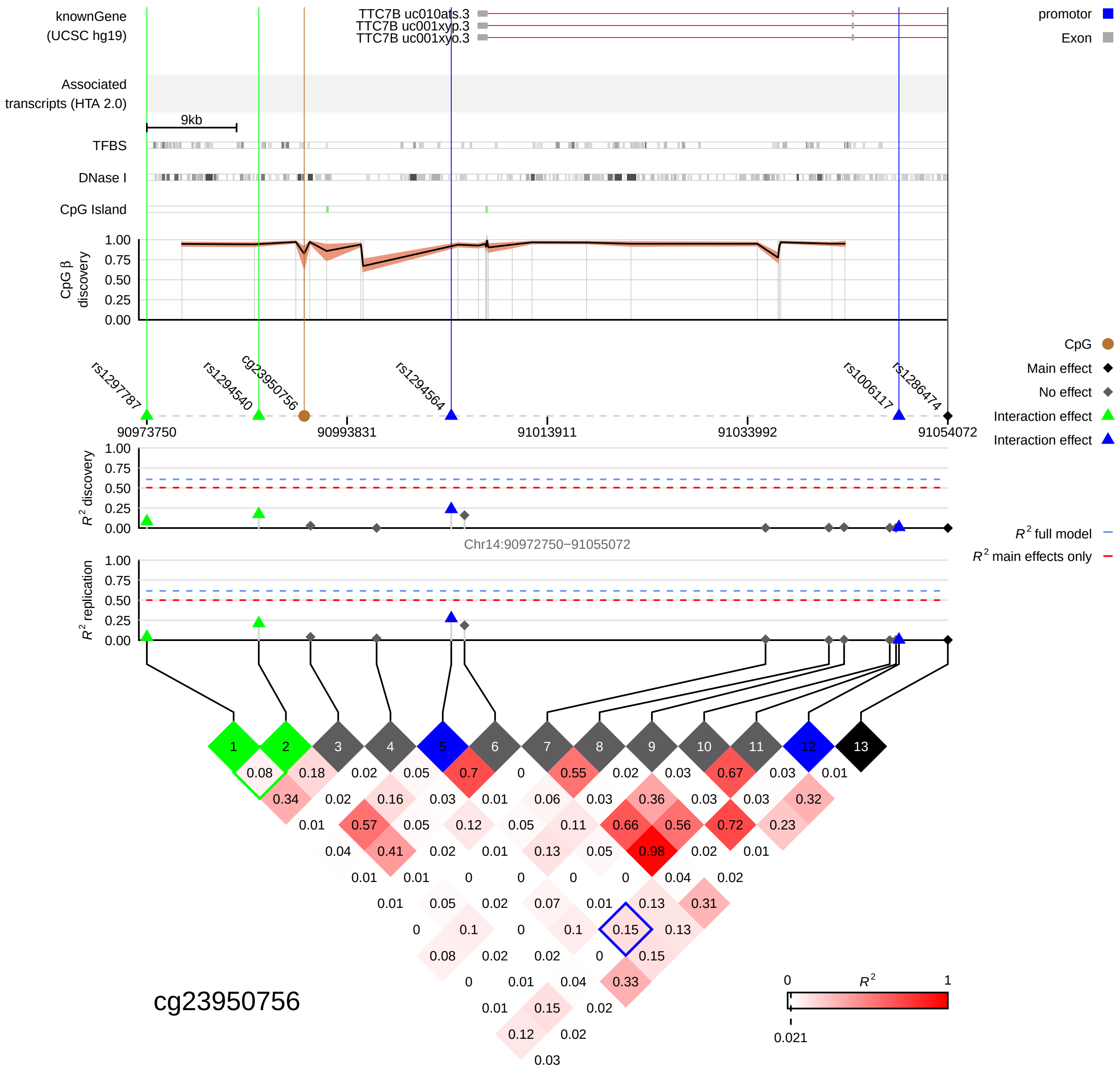

knownGene (UCSC hg19)
TTC7B uc010ats.3
TTC7B uc001xyp.3
TTC7B uc001xyo.3
promotor
Exon
Associated transcripts (HTA 2.0)
9kb
TFBS
DNase I
CpG Island
CpG β discovery
rs1297787
rs1294540
cg23950756
rs1294564
rs1006117
rs1286474
90973750
90993831
91013911
91033992
91054072
CpG
Main effect
No effect
Interaction effect
Interaction effect
R^2 discovery
Chr14:90972750−91055072
R^2 full model
R^2 main effects only
R^2 replication
cg23950756
R^2
0.021

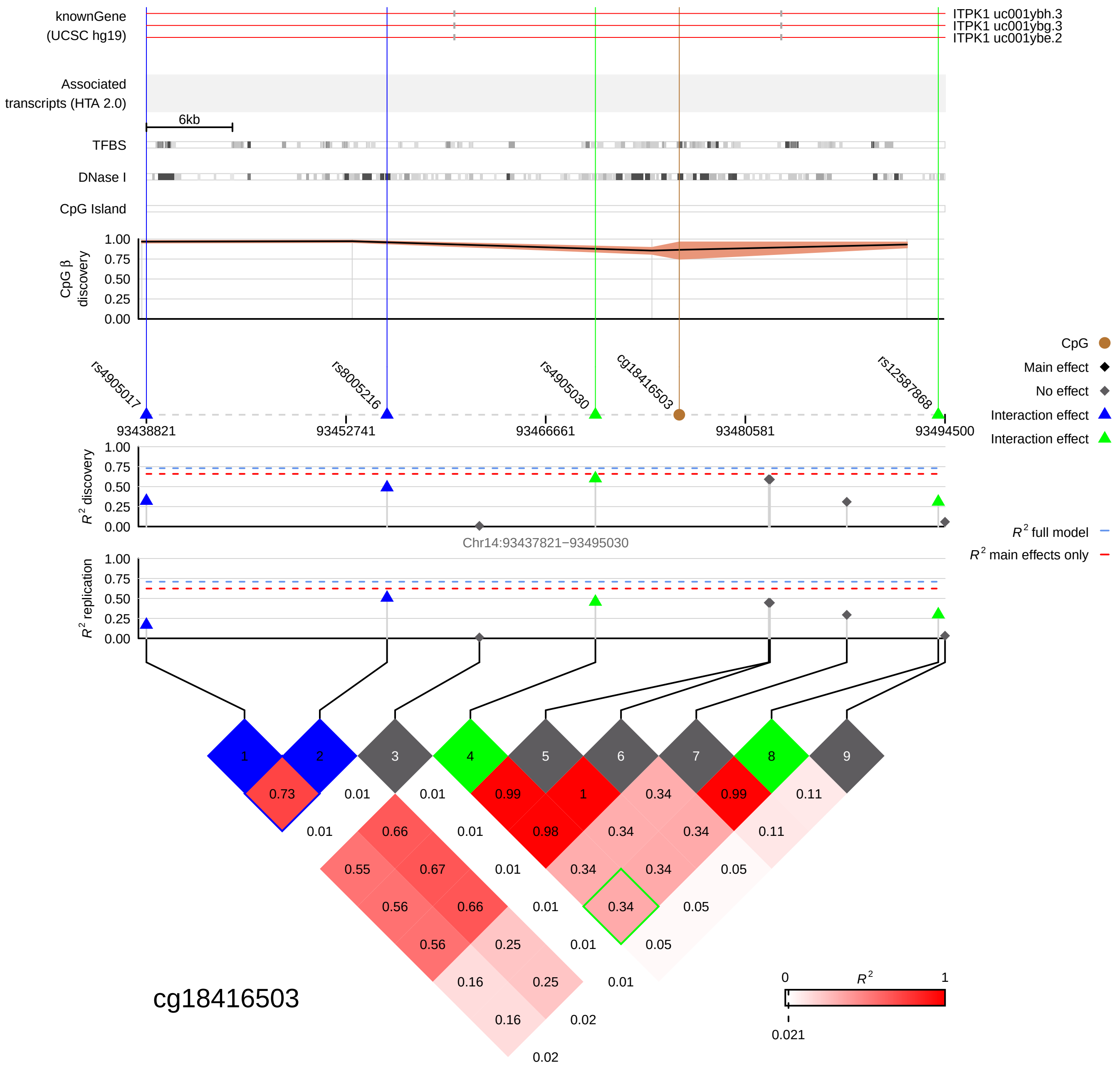

knownGene
(UCSC hg19)
ITPK1 uc001ybh.3
ITPK1 uc001ybg.3
ITPK1 uc001ybe.2
Associated transcripts (HTA 2.0)
6kb
TFBS
DNase I
CpG Island
CpG β discovery
rs4905017
rs8005216
rs4905030
cg18416503
rs12587868
93438821
93452741
93466661
93480581
93494500
CpG
Main effect
No effect
Interaction effect
Interaction effect
R^2 discovery
Chr14:93437821−93495030
R^2 full model
R^2 main effects only
R^2 replication
cg18416503
R^2
0.021

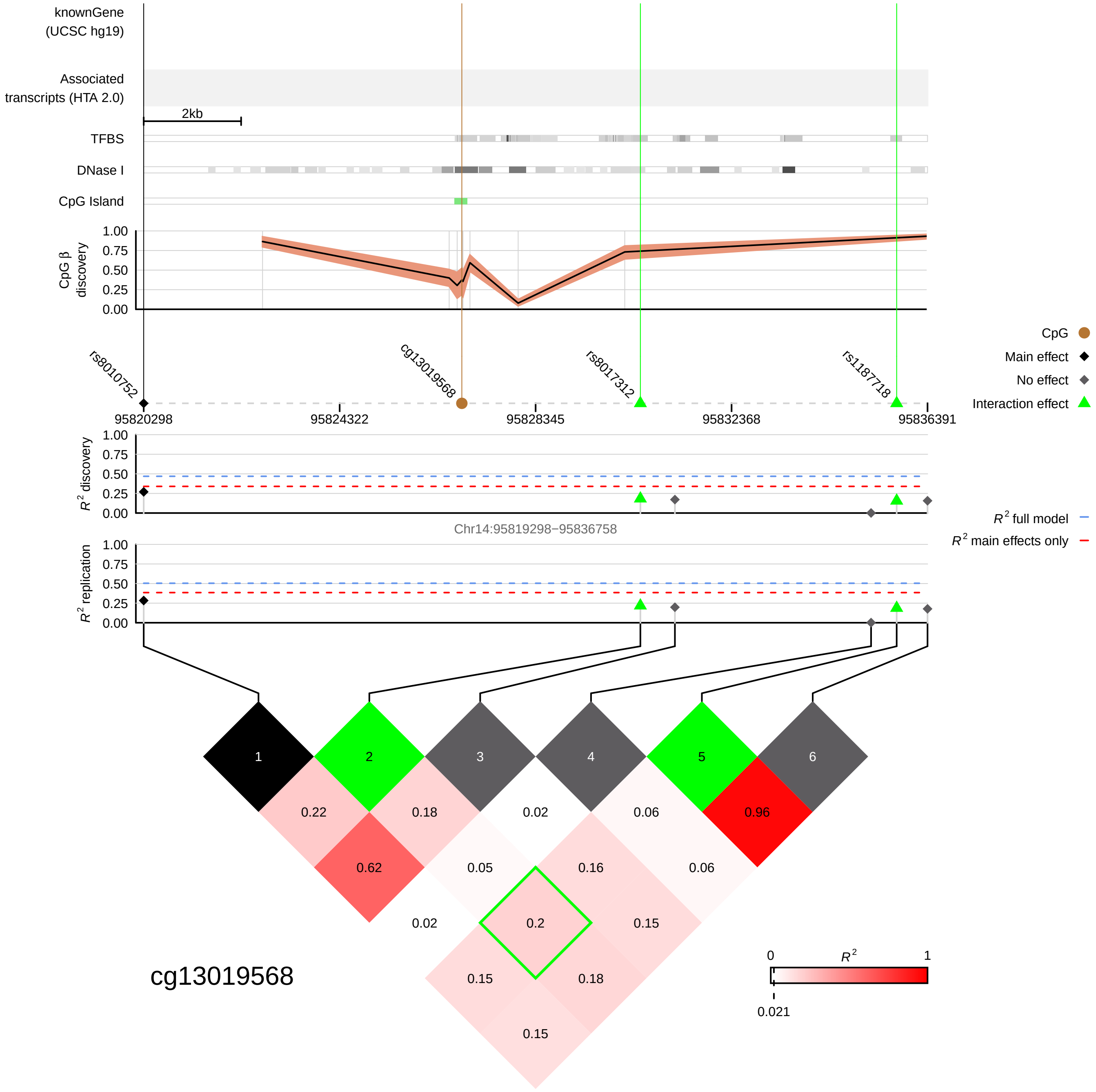

knownGene (UCSC hg19)
Associated transcripts (HTA 2.0)
2kb
TFBS
DNase I
CpG Island
CpG β discovery
1.00
0.75
0.50
0.25
0.00
rs8010752
cg13019568
rs8017312
rs1187718
95820298
95824322
95828345
95832368
95836391
CpG
Main effect
No effect
Interaction effect
R² discovery
Chr14:95819298−95836758
R² full model
R² main effects only
R² replication
1
2
3
4
5
6
0.22
0.18
0.02
0.06
0.96
0.62
0.05
0.16
0.06
0.02
0.2
0.15
0.15
0.18
0.15
cg13019568
0
R²
1
0.021

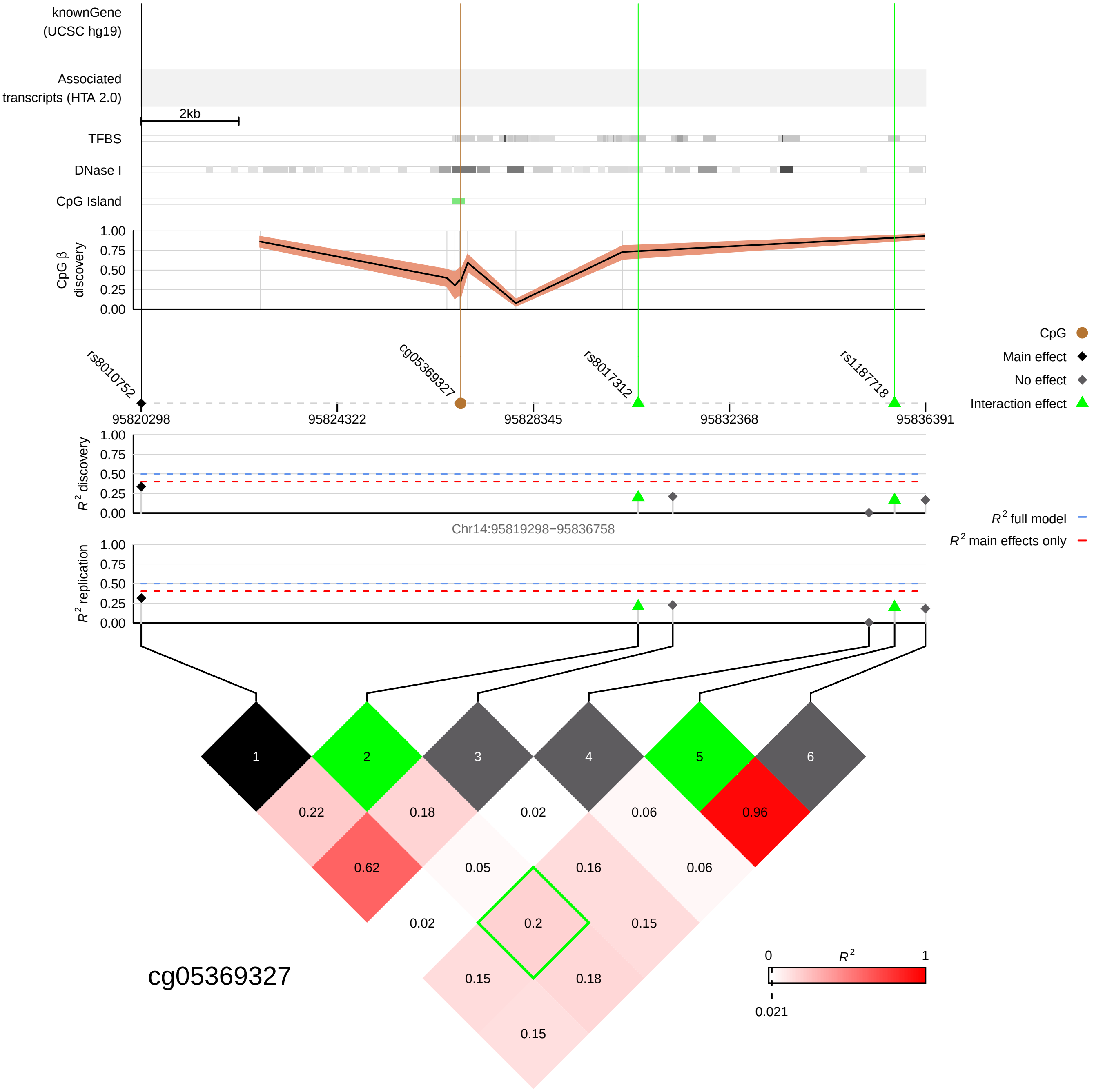
knownGene (UCSC hg19)
Associated transcripts (HTA 2.0)
2kb
TFBS
DNase I
CpG Island
CpG β discovery
rs8010752
cg05369327
rs8017312
rs1187718
95820298
95824322
95828345
95832368
95836391
CpG
Main effect
No effect
Interaction effect
R2 discovery
Chr14:95819298−95836758
R2 full model
R2 main effects only
R2 replication
1
2
3
4
5
6
0.22
0.18
0.02
0.06
0.96
0.62
0.05
0.16
0.06
0.02
0.2
0.15
0.15
0.18
0.15
cg05369327
0
R2
1
0.021

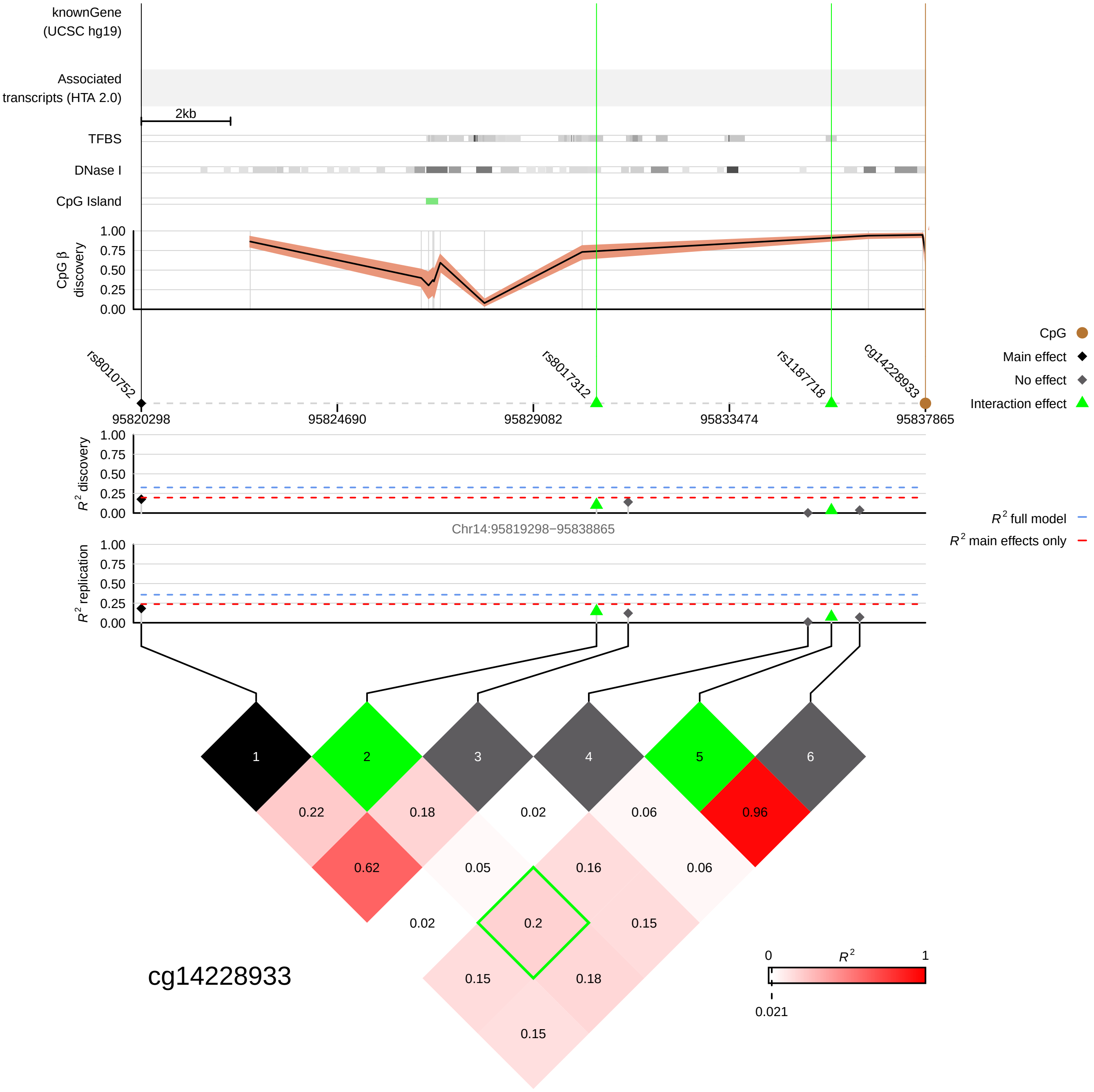

knownGene (UCSC hg19)
Associated transcripts (HTA 2.0)
2kb
TFBS
DNase I
CpG Island
CpG β discovery
1.00
0.75
0.50
0.25
0.00
rs8010752
rs8017312
rs1187718
cg14228933
95820298
95824690
95829082
95833474
95837865
CpG
Main effect
No effect
Interaction effect
R^2 discovery
Chr14:95819298−95838865
R^2 full model
R^2 main effects only
R^2 replication
1
2
3
4
5
6
0.22
0.18
0.02
0.06
0.96
0.62
0.05
0.16
0.06
0.02
0.2
0.15
0.15
0.18
0.15
cg14228933
0
R^2
1
0.021

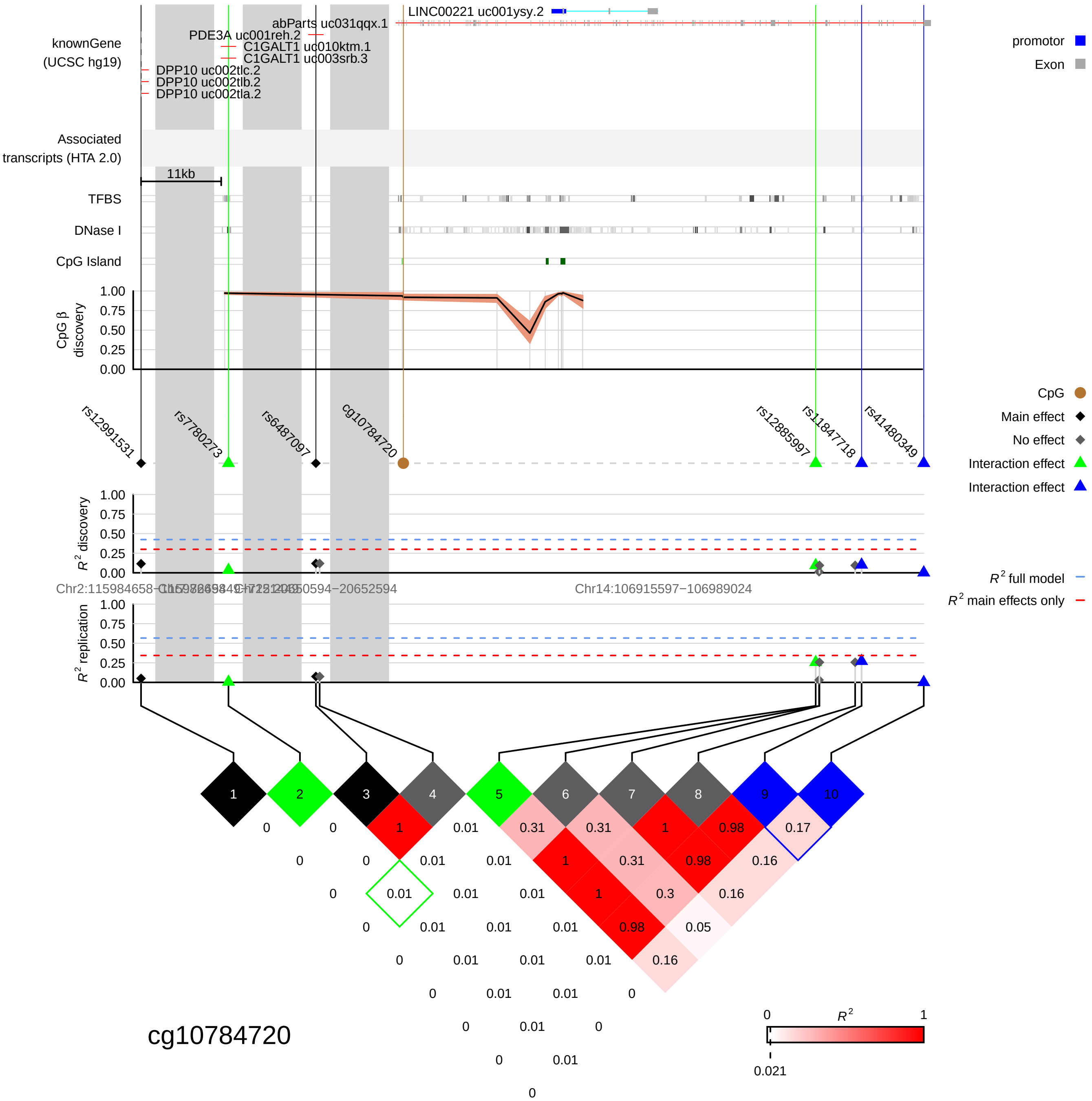

knownGene (UCSC hg19)
LINC00221 uc001ysy.2
abParts uc031qqx.1
PDE3A uc001reh.2
C1GALT1 uc010ktm.1
C1GALT1 uc003srb.3
DPP10 uc002tlc.2
DPP10 uc002tlb.2
DPP10 uc002tla.2
Associated transcripts (HTA 2.0)
11kb
TFBS
DNase I
CpG Island
CpG β discovery
1.00
0.75
0.50
0.25
0.00
rs12991531
rs7780273
rs6487097
cg10784720
rs12885997
rs11847718
rs41480349
R^2 discovery
Chr14:106915597−106989024
R^2 replication
promotor
Exon
CpG
Main effect
No effect
Interaction effect
Interaction effect
R^2 full model
R^2 main effects only
R^2
0
1
0.021
cg10784720

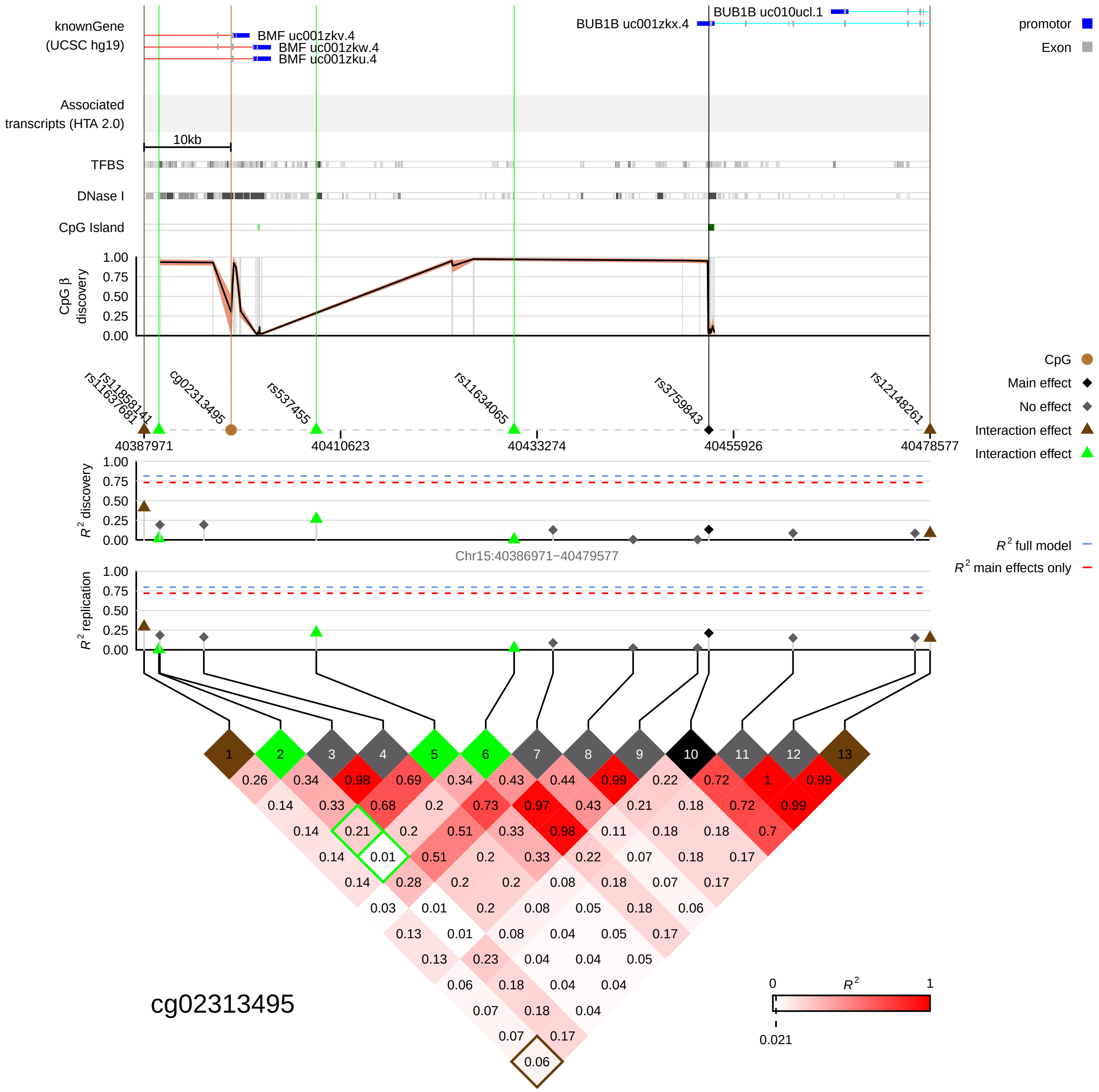
knownGene (UCSC hg19)
BMF uc001zkv.4
BMF uc001zkw.4
BMF uc001zku.4
BUB1B uc001zkx.4
BUB1B uc010ucl.1
promotor
Exon
Associated transcripts (HTA 2.0)
10kb
TFBS
DNase I
CpG Island
CpG β discovery
rs11858141
rs11637681
cg02313495
rs537455
rs11634065
rs3759843
rs12148261
40387971
40410623
40433274
40455926
40478577
CpG
Main effect
No effect
Interaction effect
Interaction effect
R² discovery
Chr15:40386971−40479577
R² full model
R² main effects only
R² replication
cg02313495
0
R²
1
0.021

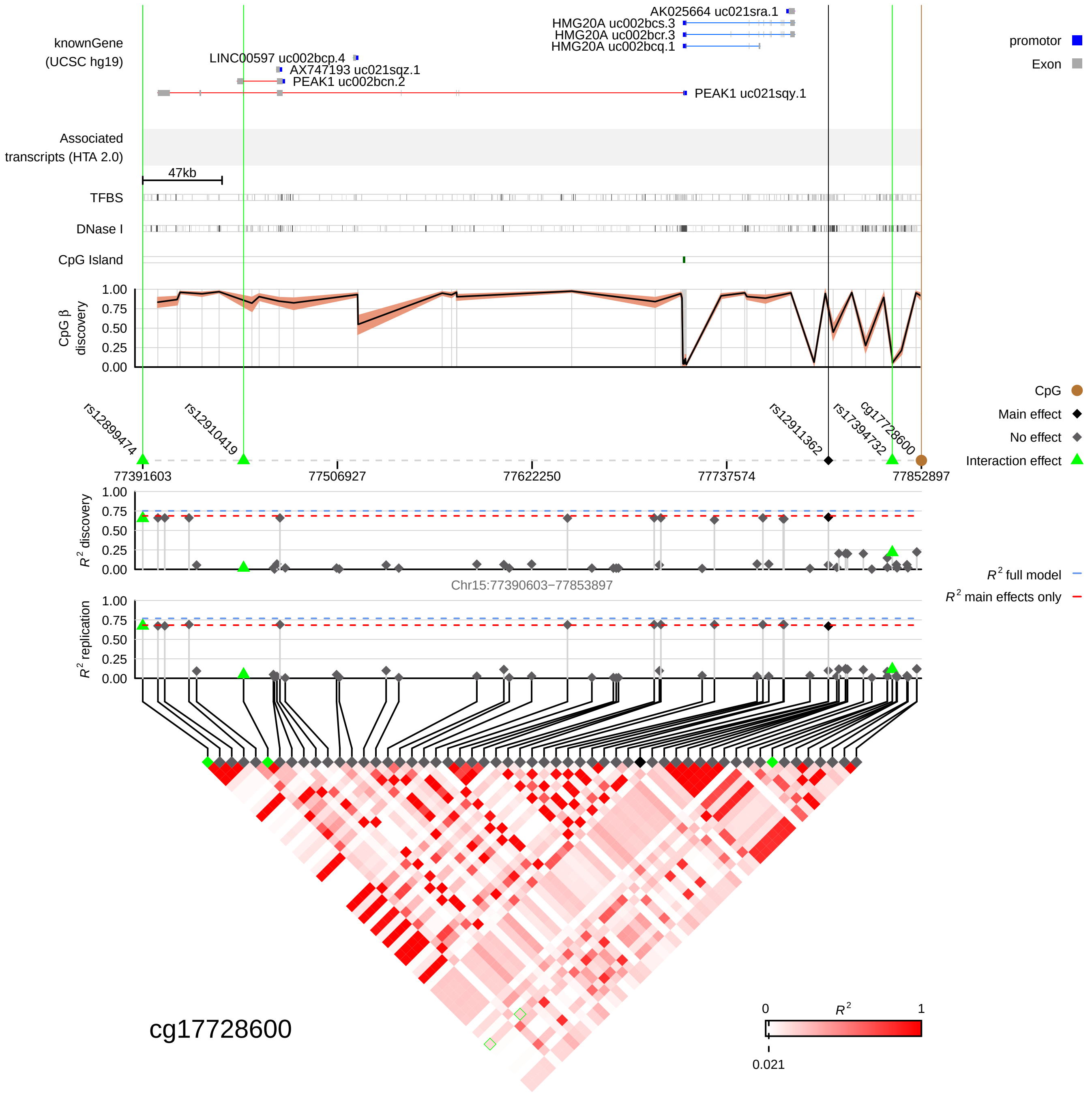

knownGene (UCSC hg19)
AK025664 uc021sra.1
HMG20A uc002bcs.3
HMG20A uc002bcr.3
HMG20A uc002bcq.1
LINC00597 uc002bcp.4
AX747193 uc021sqz.1
PEAK1 uc002bcn.2
PEAK1 uc021sqy.1
promotor
Exon
Associated transcripts (HTA 2.0)
47kb
TFBS
DNase I
CpG Island
CpG β discovery
rs12899474
rs12910419
rs12911362
rs17394732
cg17728600
CpG
Main effect
No effect
Interaction effect
77391603
77506927
77622250
77737574
77852897
R^2 discovery
Chr15:77390603−77853897
R^2 full model
R^2 main effects only
R^2 replication
cg17728600
0
R^2
1
0.021

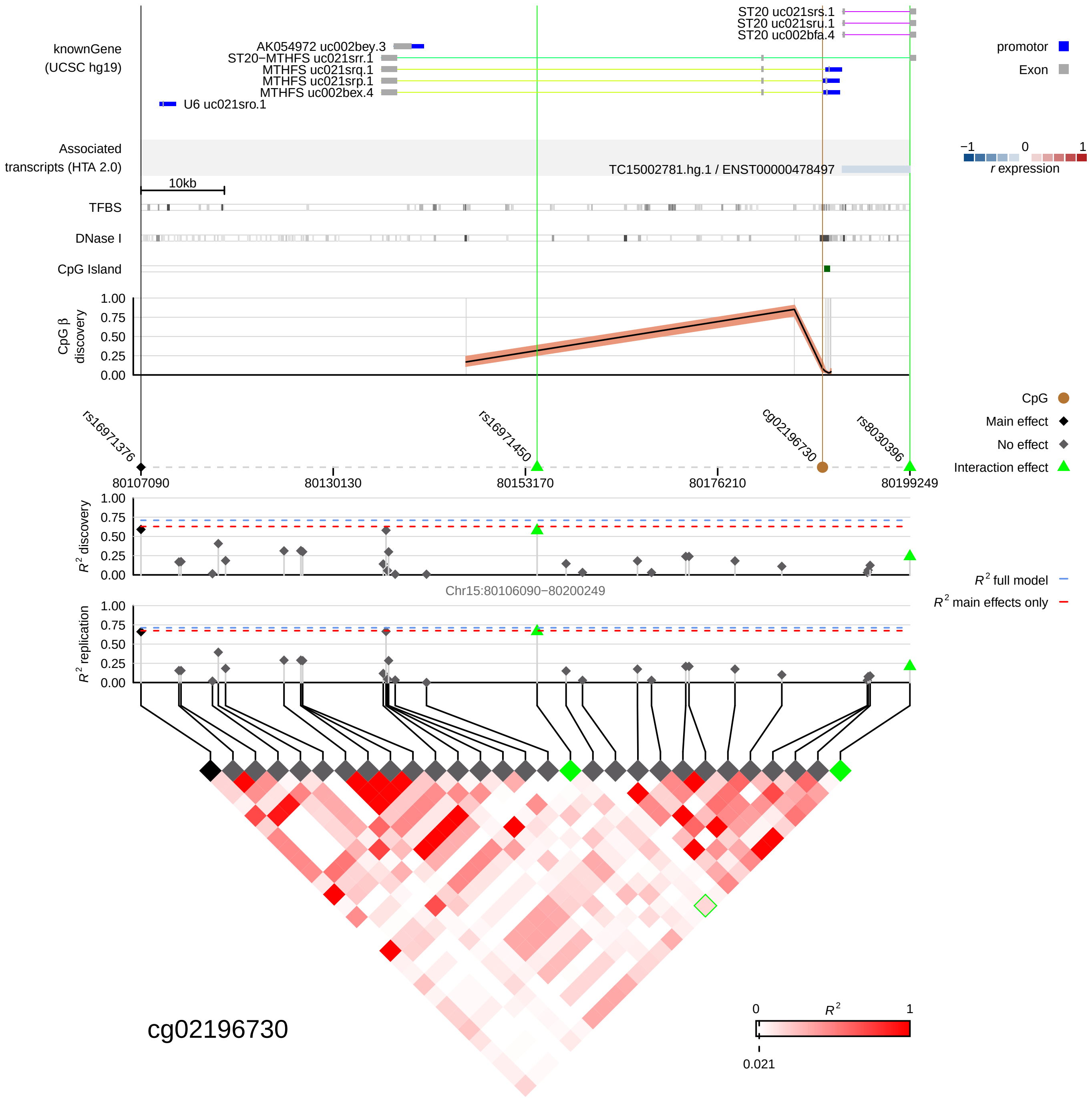
knownGene
(UCSC hg19)
ST20 uc021srs.1
ST20 uc021sru.1
ST20 uc002bfa.4
AK054972 uc002bey.3
ST20−MTHFS uc021srr.1
MTHFS uc021srq.1
MTHFS uc021srp.1
MTHFS uc002bex.4
U6 uc021sro.1
promotor
Exon
Associated
transcripts (HTA 2.0)
TC15002781.hg.1 / ENST00000478497
−1
0
1
r expression
10kb
TFBS
DNase I
CpG Island
CpG β
discovery
1.00
0.75
0.50
0.25
0.00
rs16971376
rs16971450
cg02196730
rs8030396
CpG
Main effect
No effect
Interaction effect
80107090
80130130
80153170
80176210
80199249
R2 discovery
Chr15:80106090−80200249
R2 full model
R2 main effects only
R2 replication
0
R2
1
0.021
cg02196730

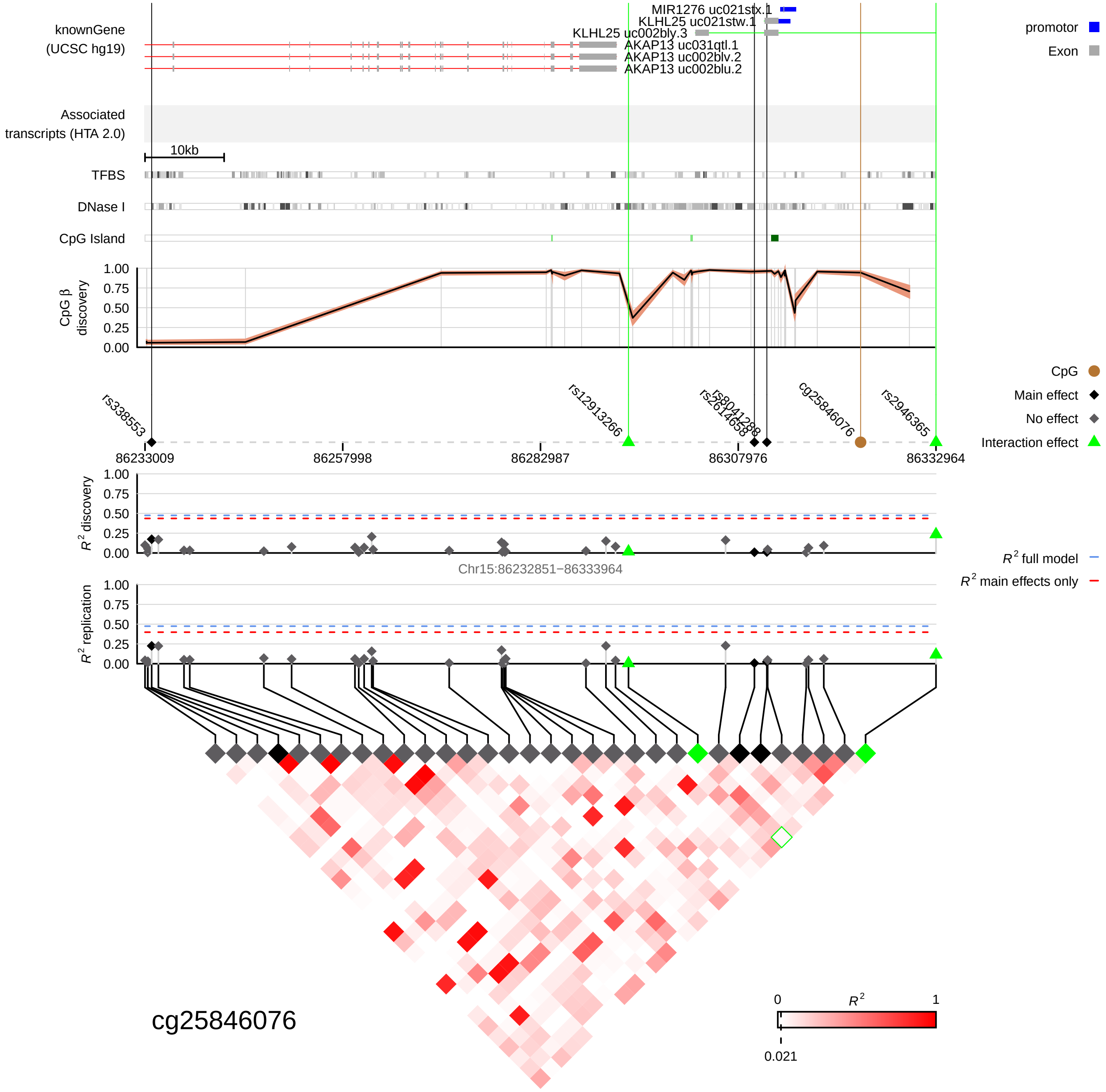

knownGene
(UCSC hg19)
MIR1276 uc021stx.1
KLHL25 uc021stw.1
KLHL25 uc002bly.3
AKAP13 uc031qtl.1
AKAP13 uc002blv.2
AKAP13 uc002blu.2
promotor
Exon
Associated
transcripts (HTA 2.0)
10kb
TFBS
DNase I
CpG Island
CpG β
discovery
1.00
0.75
0.50
0.25
0.00
rs338553
rs12913266
rs2614658
rs8041288
cg25846076
rs2946365
86233009
86257998
86282987
86307976
86332964
CpG
Main effect
No effect
Interaction effect
R^2 discovery
Chr15:86232851−86333964
R^2 full model
R^2 main effects only
R^2 replication
0
R^2
1
0.021
cg25846076

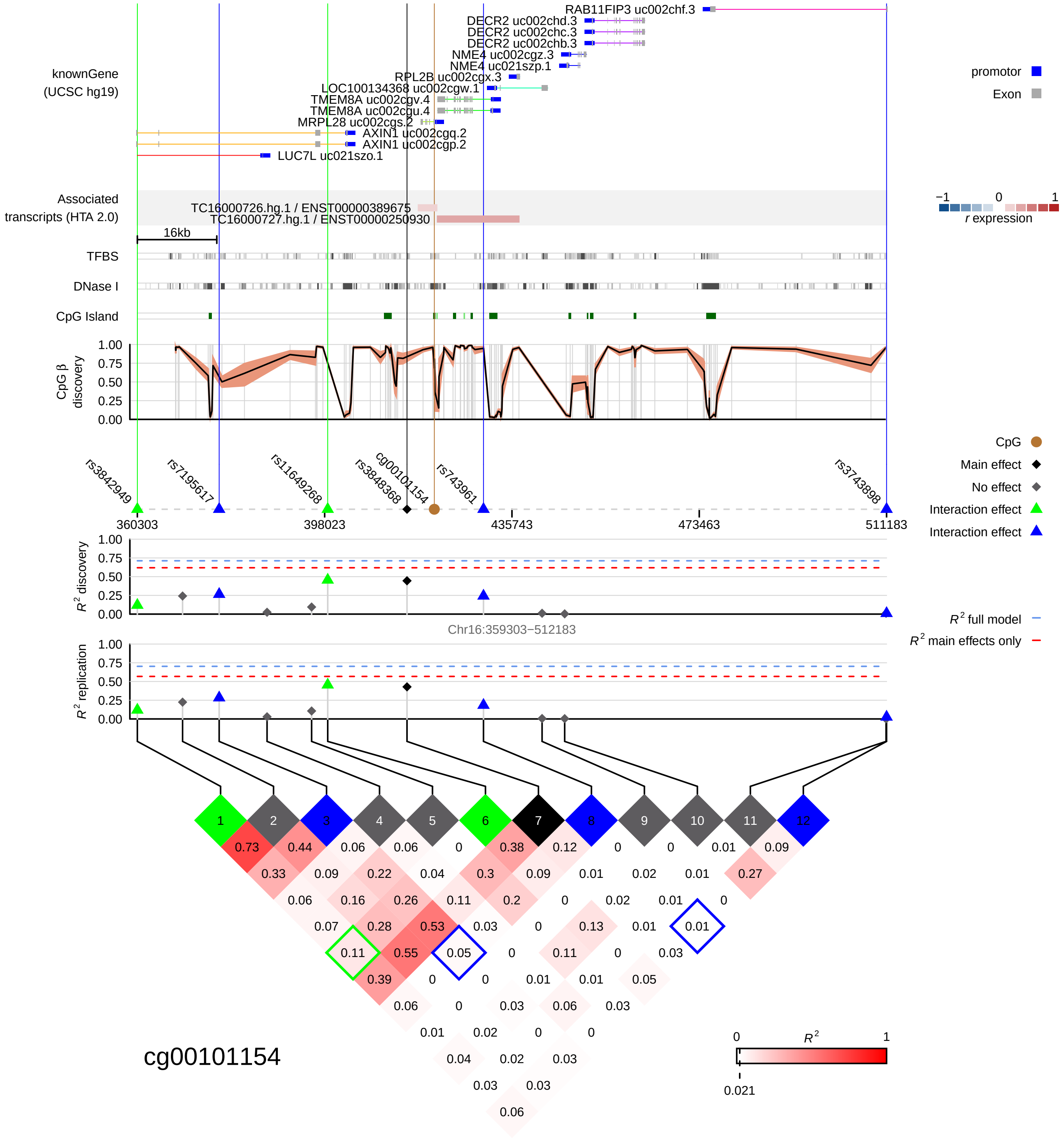
knownGene (UCSC hg19)
RAB11FIP3 uc002chf.3
DECR2 uc002chd.3
DECR2 uc002chc.3
DECR2 uc002chb.3
NME4 uc002cgz.3
NME4 uc021szp.1
RPL2B uc002cgx.3
LOC100134368 uc002cgw.1
TMEM8A uc002cgv.4
TMEM8A uc002cgu.4
MRPL28 uc002cgs.2
AXIN1 uc002cgq.2
AXIN1 uc002cgp.2
LUC7L uc021szo.1
promotor
Exon
Associated transcripts (HTA 2.0)
TC16000726.hg.1 / ENST00000389675
TC16000727.hg.1 / ENST00000250930
r expression
16kb
TFBS
DNase I
CpG Island
CpG β discovery
rs3842949
rs7195617
rs11649268
rs3848368
cg00101154
rs743961
rs3743898
360303
398023
435743
473463
511183
CpG
Main effect
No effect
Interaction effect
Interaction effect
R^2 discovery
Chr16:359303−512183
R^2 full model
R^2 main effects only
R^2 replication
cg00101154
R^2
0.021

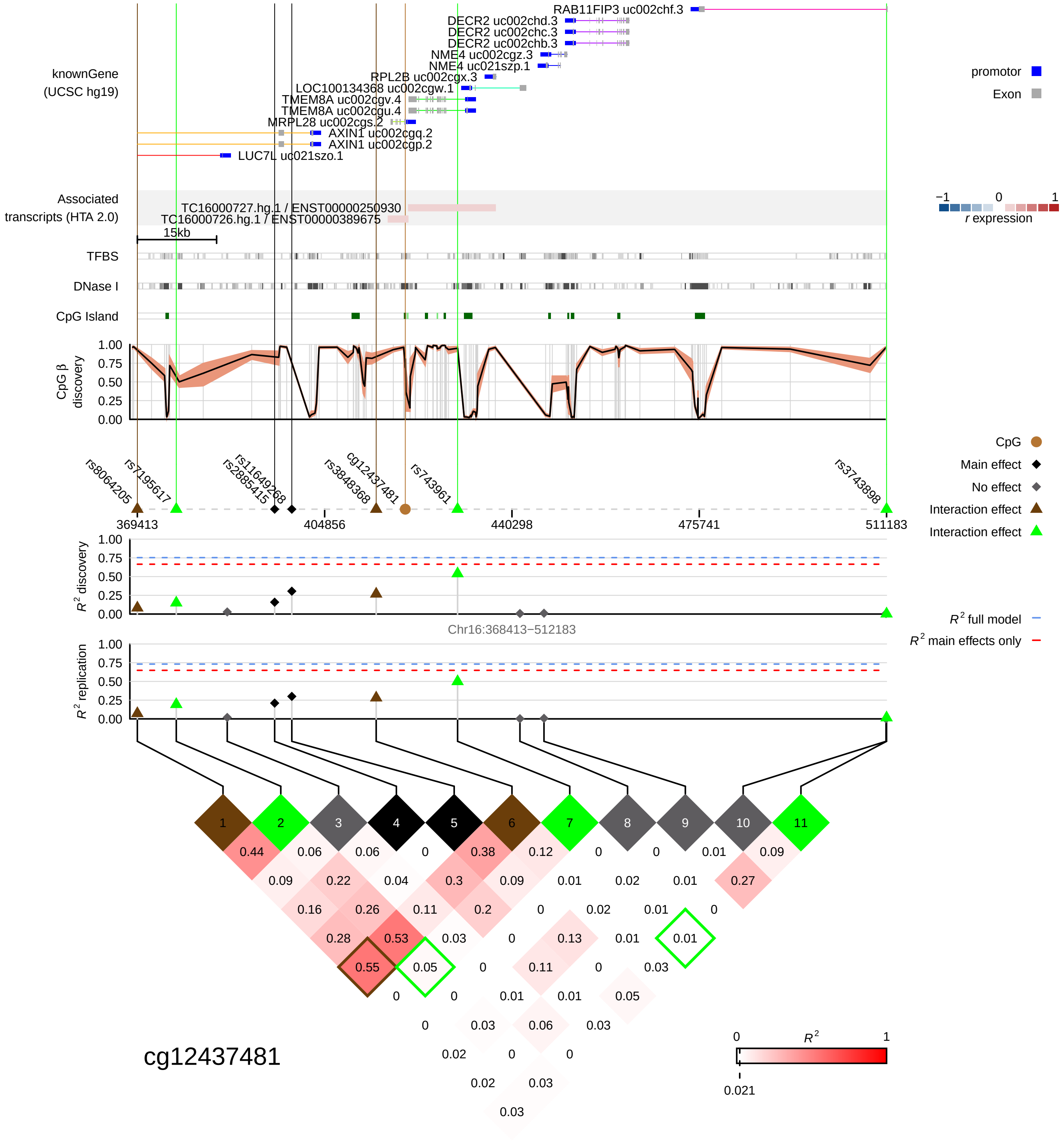

knownGene (UCSC hg19)
RAB11FIP3 uc002chf.3
DECR2 uc002chd.3
DECR2 uc002chc.3
DECR2 uc002chb.3
NME4 uc002cgz.3
NME4 uc021szp.1
RPL28 uc002cgx.3
LOC100134368 uc002cgw.1
TMEM8A uc002cgv.4
TMEM8A uc002cgu.4
MRPL28 uc002cgs.2
AXIN1 uc002cgq.2
AXIN1 uc002cgp.2
LUC7L uc021szo.1
promotor
Exon
Associated transcripts (HTA 2.0)
TC16000727.hg.1 / ENST00000250930
TC16000726.hg.1 / ENST00000389675
15kb
−1 0 1
r expression
TFBS
DNase I
CpG Island
CpG β discovery
rs8064205
rs7195617
rs11649268
rs2885415
rs3848368
cg12437481
rs743961
rs3743898
369413
404856
440298
475741
511183
CpG
Main effect
No effect
Interaction effect
Interaction effect
R^2 discovery
Chr16:368413−512183
R^2 full model
R^2 main effects only
R^2 replication
1 2 3 4 5 6 7 8 9 10 11
cg12437481
0 R^2 1
0.021

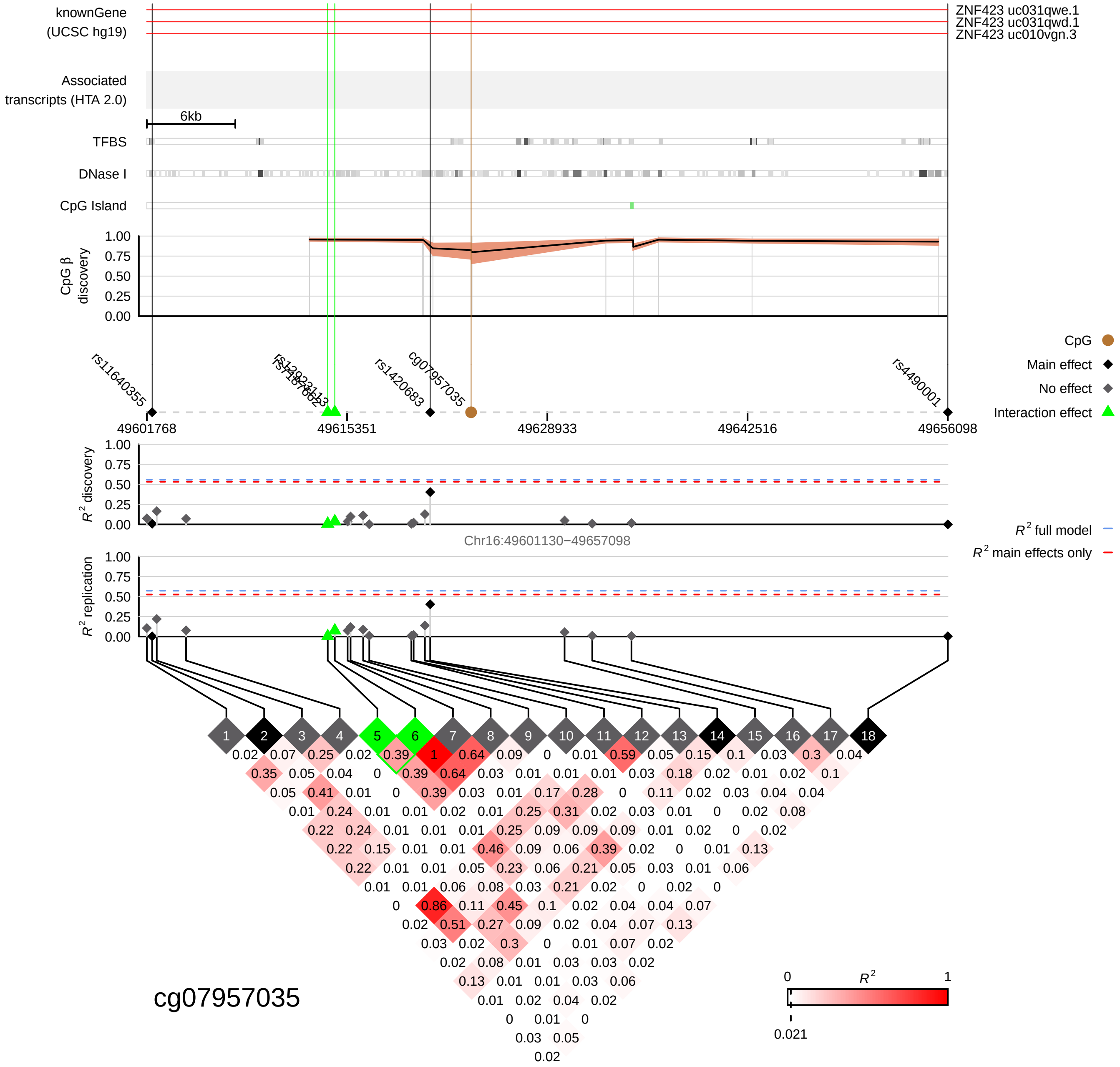

knownGene
(UCSC hg19)
ZNF423 uc031qwe.1
ZNF423 uc031qwd.1
ZNF423 uc010vgn.3
Associated
transcripts (HTA 2.0)
6kb
TFBS
DNase I
CpG Island
CpG β
discovery
1.00
0.75
0.50
0.25
0.00
rs11640355
rs12923113
rs7187062
rs1420683
cg07957035
rs4490001
49601768
49615351
49628933
49642516
49656098
CpG
Main effect
No effect
Interaction effect
R² discovery
Chr16:49601130−49657098
R² full model
R² main effects only
R² replication
1 2 3 4 5 6 7 8 9 10 11 12 13 14 15 16 17 18
0.02 0.07 0.25 0.02 0.39 1 0.64 0.09 0 0.01 0.59 0.05 0.15 0.1 0.03 0.3 0.04
0.35 0.05 0.04 0 0.39 0.64 0.03 0.01 0.01 0.01 0.03 0.18 0.02 0.01 0.02 0.1
0.05 0.41 0.01 0 0.39 0.03 0.01 0.17 0.28 0 0.11 0.02 0.03 0.04 0.04
0.01 0.24 0.01 0.01 0.02 0.01 0.25 0.31 0.02 0.03 0.01 0 0.02 0.08
0.22 0.24 0.01 0.01 0.01 0.25 0.09 0.09 0.09 0.01 0.02 0 0.02
0.22 0.15 0.01 0.01 0.46 0.09 0.06 0.39 0.02 0 0.01 0.13
0.22 0.01 0.01 0.05 0.23 0.06 0.21 0.05 0.03 0.01 0.06
0.01 0.01 0.06 0.08 0.03 0.21 0.02 0 0.02 0
0 0.86 0.11 0.45 0.1 0.02 0.04 0.04 0.07
0.02 0.51 0.27 0.09 0.02 0.04 0.07 0.13
0.03 0.02 0.3 0 0.01 0.07 0.02
0.02 0.08 0.01 0.03 0.03 0.02
0.13 0.01 0.01 0.03 0.06
0.01 0.02 0.04 0.02
0 0.01 0
0.03 0.05
0.02
cg07957035
0
R²
1
0.021

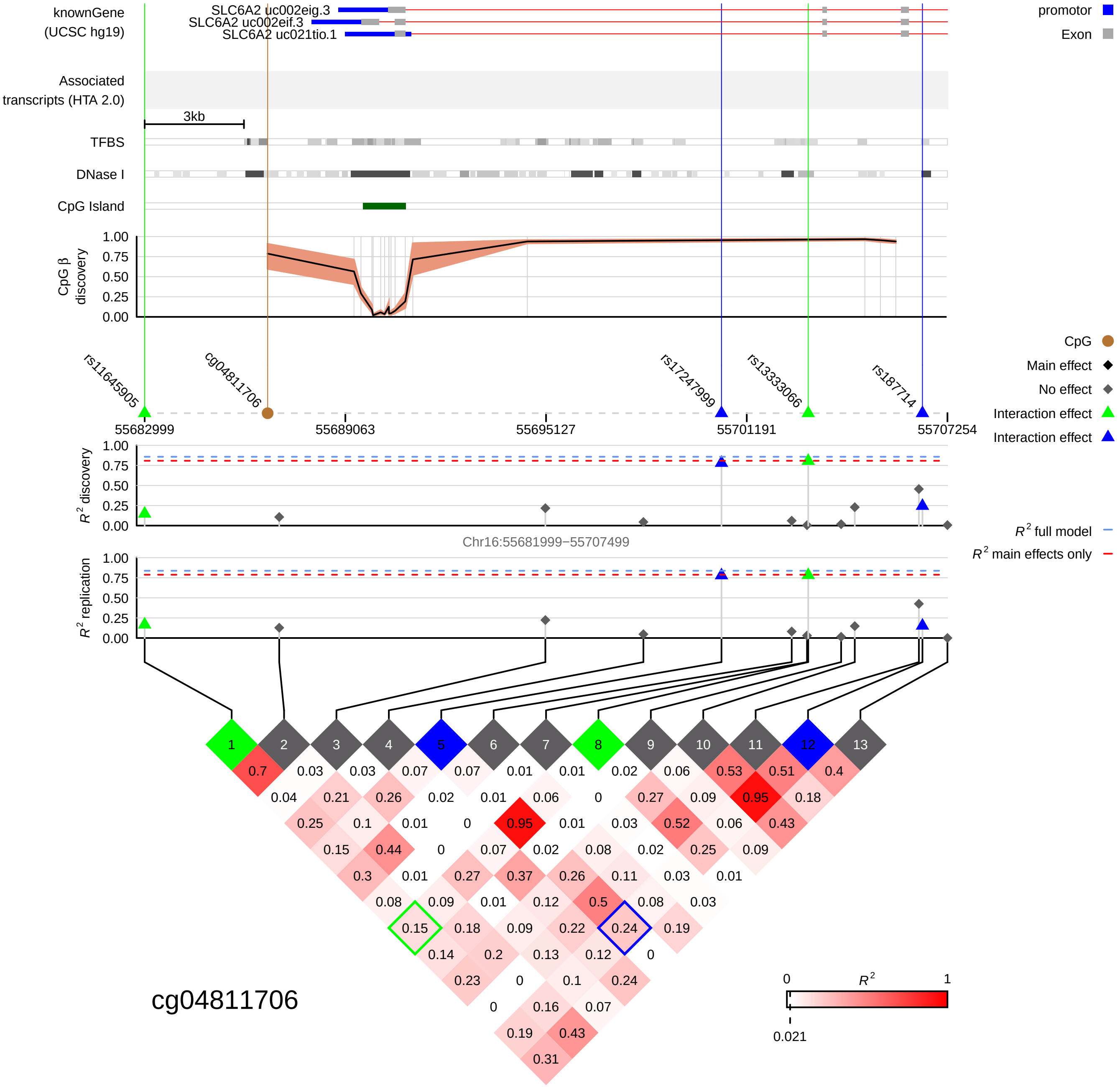
knownGene (UCSC hg19)
SLC6A2 uc002eig.3
SLC6A2 uc002eif.3
SLC6A2 uc021tio.1
promotor
Exon
Associated transcripts (HTA 2.0)
3kb
TFBS
DNase I
CpG Island
CpG β discovery
rs11645905
cg04811706
rs17247999
rs13333066
rs187714
55682999
55689063
55695127
55701191
55707254
CpG
Main effect
No effect
Interaction effect
Interaction effect
R^2 discovery
Chr16:55681999−55707499
R^2 replication
R^2 full model
R^2 main effects only
cg04811706
R^2
0
1
0.021

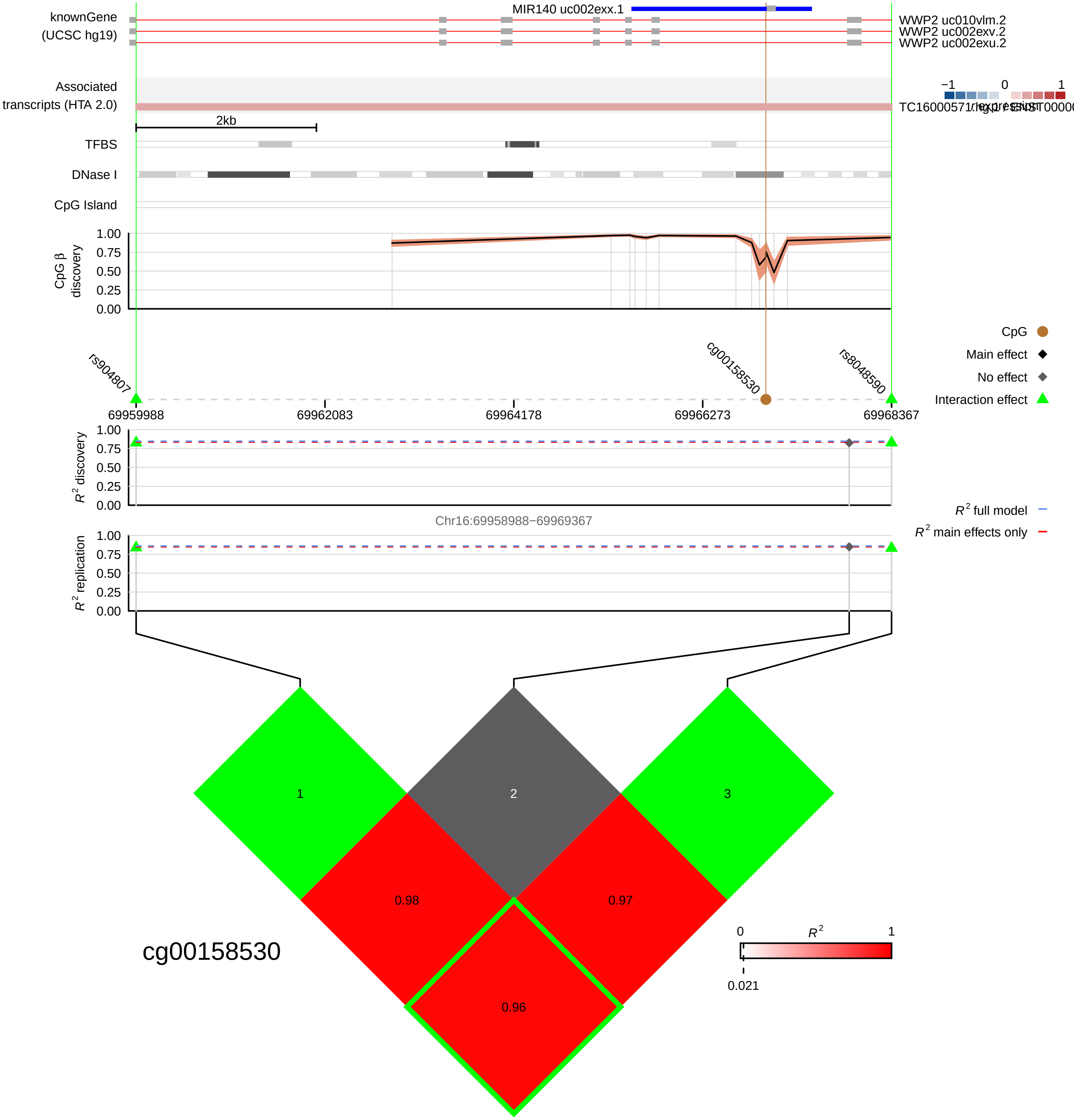

knownGene (UCSC hg19)
MIR140 uc002exx.1
WWP2 uc010vlm.2
WWP2 uc002exv.2
WWP2 uc002exu.2
Associated transcripts (HTA 2.0)
2kb
TFBS
DNase I
CpG Island
CpG β discovery
1.00
0.75
0.50
0.25
0.00
CpG
Main effect
No effect
Interaction effect
rs904807
cg00158530
rs8048590
69959988
69962083
69964178
69966273
69968367
R^2 discovery
R^2 full model
R^2 main effects only
Chr16:69958988−69969367
R^2 replication
1
2
3
0.98
0.97
0.96
cg00158530
0
R^2
1
0.021

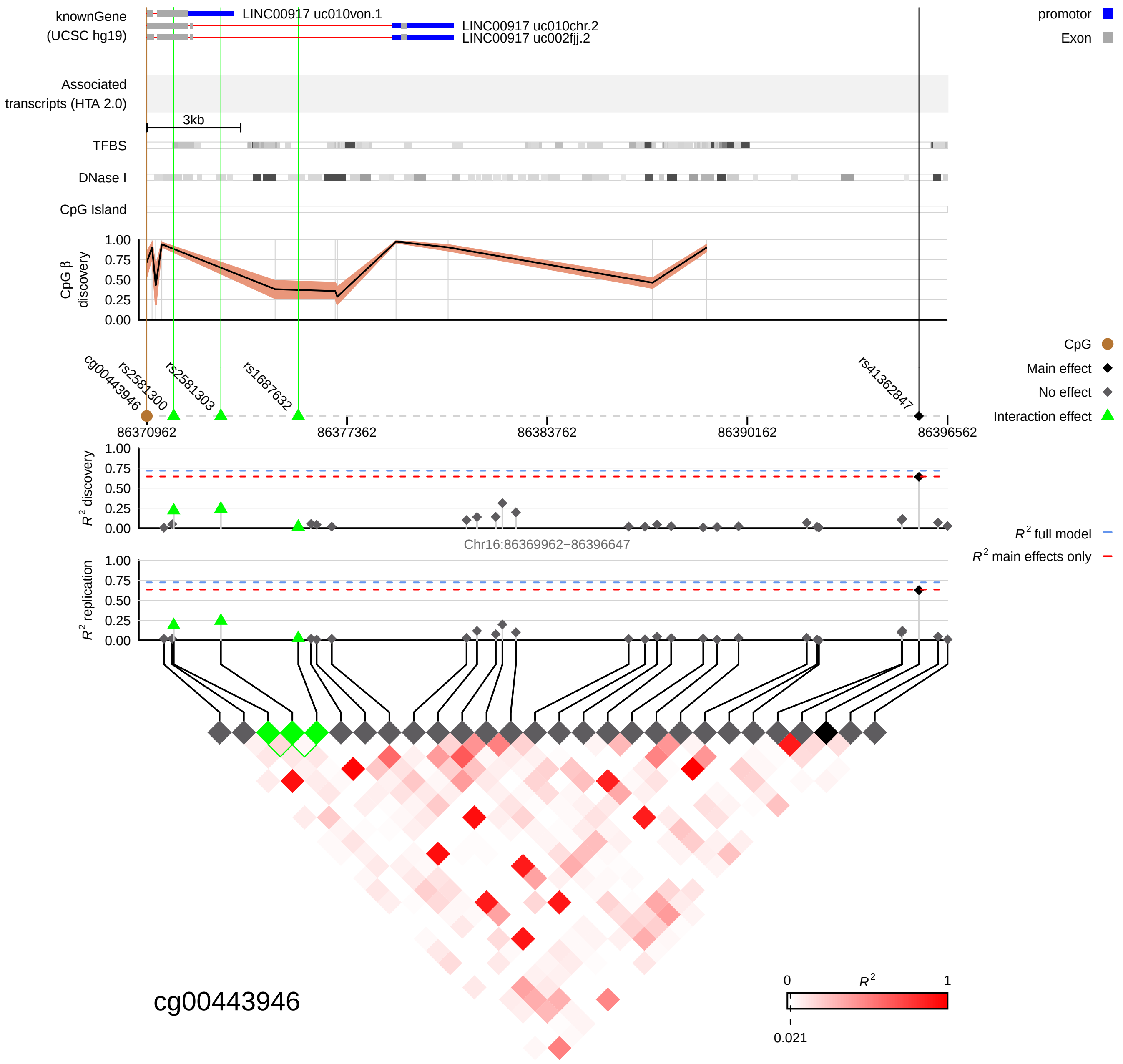
knownGene
(UCSC hg19)
LINC00917 uc010von.1
LINC00917 uc010chr.2
LINC00917 uc002fjj.2
promotor
Exon
Associated
transcripts (HTA 2.0)
3kb
TFBS
DNase I
CpG Island
CpG β
discovery
1.00
0.75
0.50
0.25
0.00
cg00443946
rs2581300
rs2581303
rs1687632
rs41362847
CpG
Main effect
No effect
Interaction effect
86370962
86377362
86383762
86390162
86396562
R^2 discovery
Chr16:86369962−86396647
R^2 full model
R^2 main effects only
R^2 replication
cg00443946
0
R^2
1
0.021

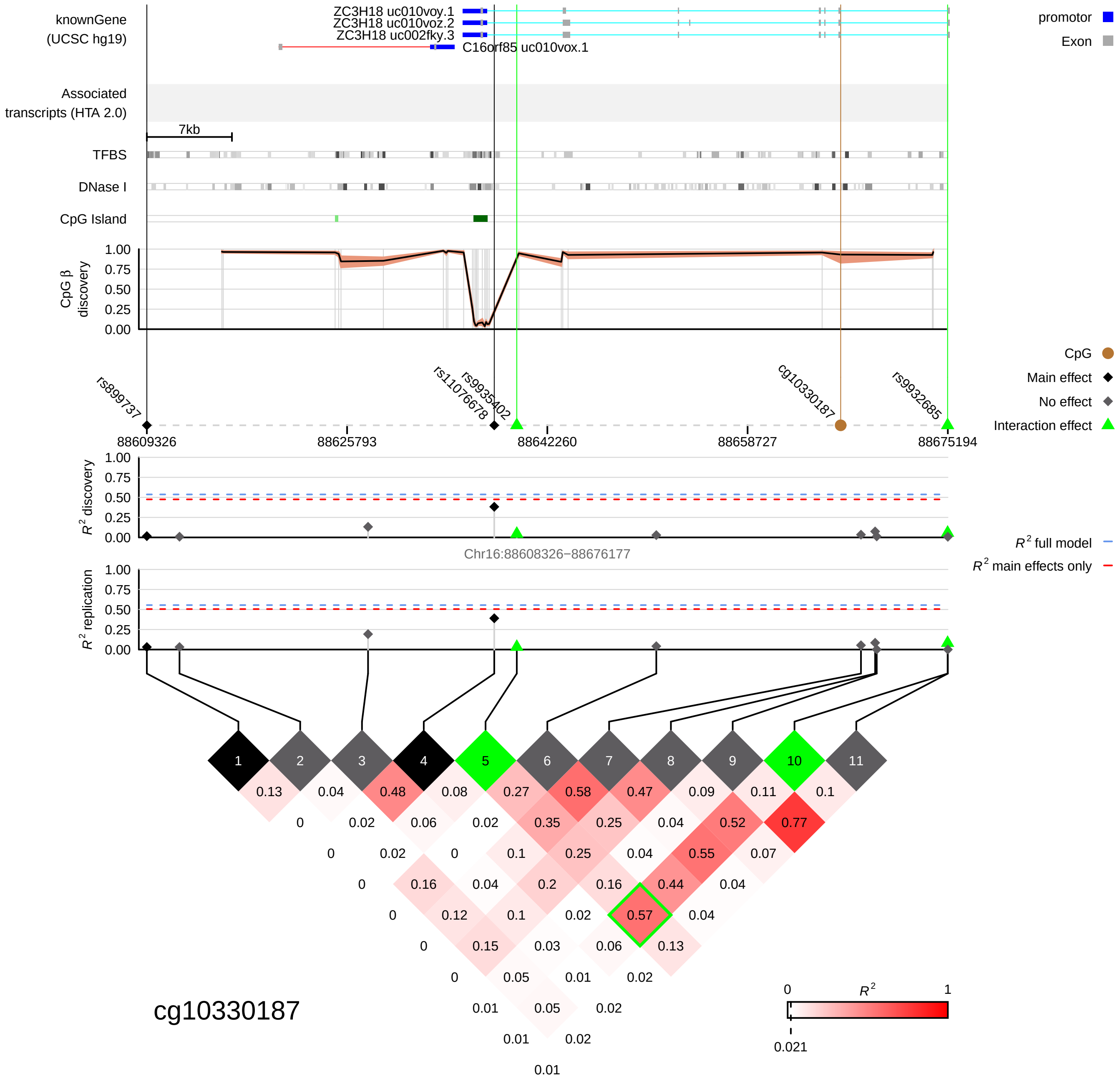

knownGene (UCSC hg19)
ZC3H18 uc010voy.1
ZC3H18 uc010voz.2
ZC3H18 uc002fky.3
C16orf85 uc010vox.1
promotor
Exon
Associated transcripts (HTA 2.0)
7kb
TFBS
DNase I
CpG Island
CpG β discovery
rs899737
rs11076678
rs9935402
cg10330187
rs9932685
88609326
88625793
88642260
88658727
88675194
CpG
Main effect
No effect
Interaction effect
R^2 discovery
Chr16:88608326−88676177
R^2 replication
R^2 full model
R^2 main effects only
R^2
0.021
cg10330187

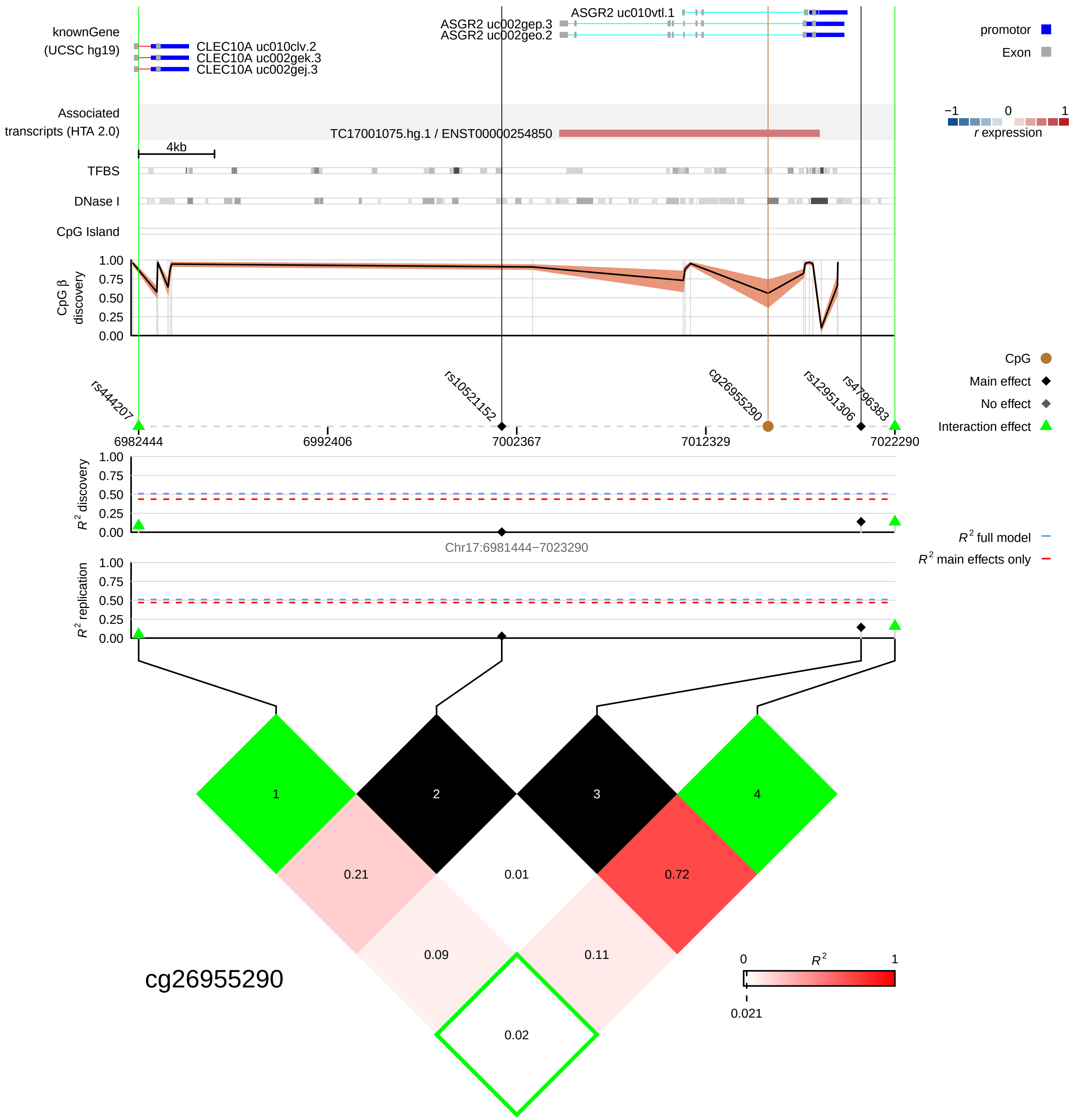

knownGene
(UCSC hg19)
CLEC10A uc010clv.2
CLEC10A uc002gek.3
CLEC10A uc002gej.3
ASGR2 uc010vtl.1
ASGR2 uc002gep.3
ASGR2 uc002geo.2
promotor
Exon
Associated
transcripts (HTA 2.0)
TC17001075.hg.1 / ENST00000254850
−1
0
1
r expression
4kb
TFBS
DNase I
CpG Island
CpG β
discovery
1.00
0.75
0.50
0.25
0.00
rs444207
rs10521152
cg26955290
rs12951306
rs4796383
CpG
Main effect
No effect
Interaction effect
6982444
6992406
7002367
7012329
7022290
R² discovery
Chr17:6981444−7023290
R² full model
R² main effects only
R² replication
1
2
3
4
0.21
0.01
0.72
0.09
0.11
0.02
cg26955290
0
R²
1
0.021

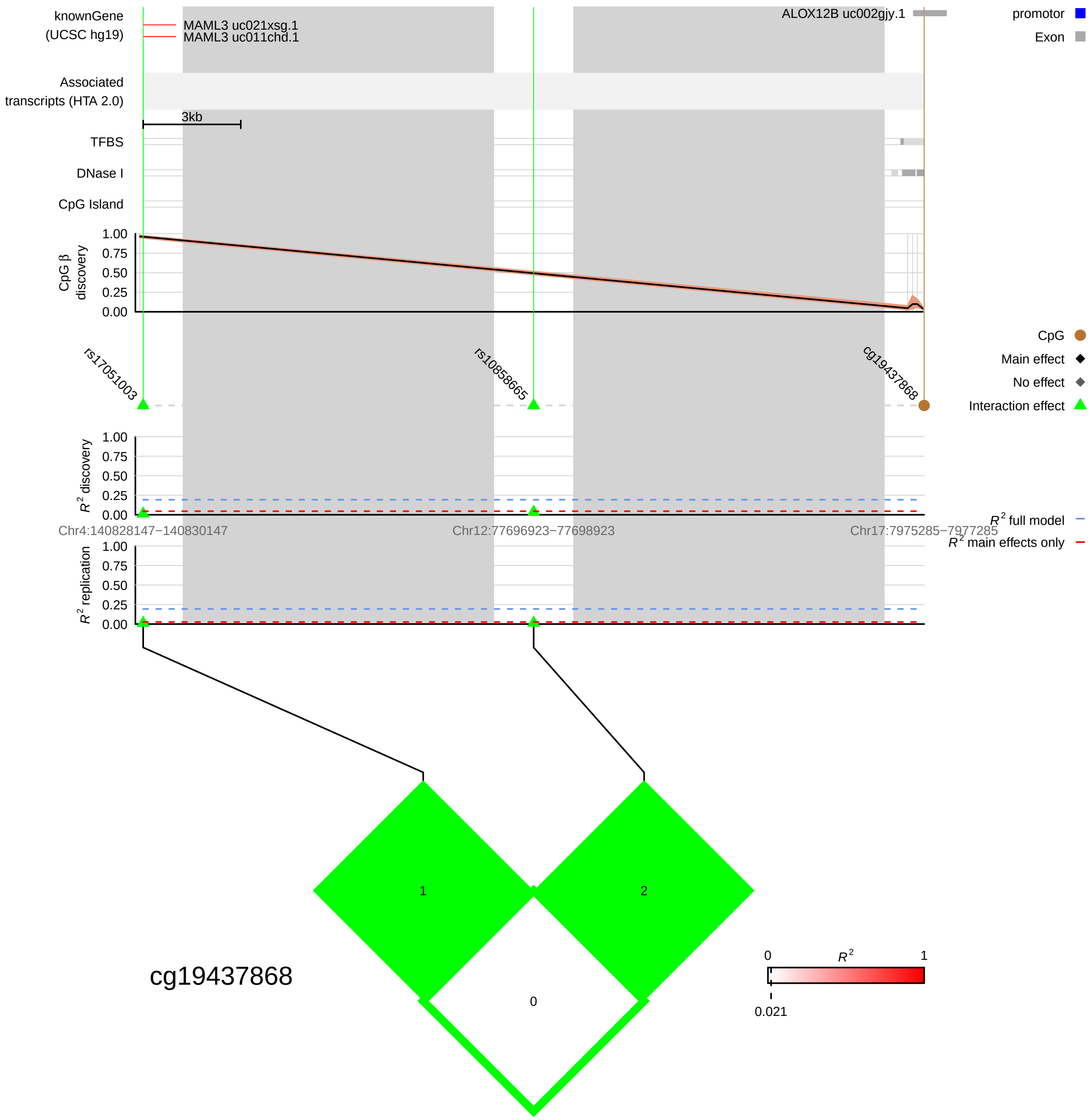
knownGene
(UCSC hg19)
MAML3 uc021xsg.1
MAML3 uc011chd.1
ALOX12B uc002gjy.1
promotor
Exon
Associated
transcripts (HTA 2.0)
3kb
TFBS
DNase I
CpG Island
CpG β
discovery
1.00
0.75
0.50
0.25
0.00
rs17051003
rs10858665
cg19437868
CpG
Main effect
No effect
Interaction effect
R2 discovery
Chr4:140828147−140830147
Chr12:77696923−77698923
Chr17:7975285−7977285
R2 full model
R2 main effects only
R2 replication
1
2
0
cg19437868
0
R2
1
0.021

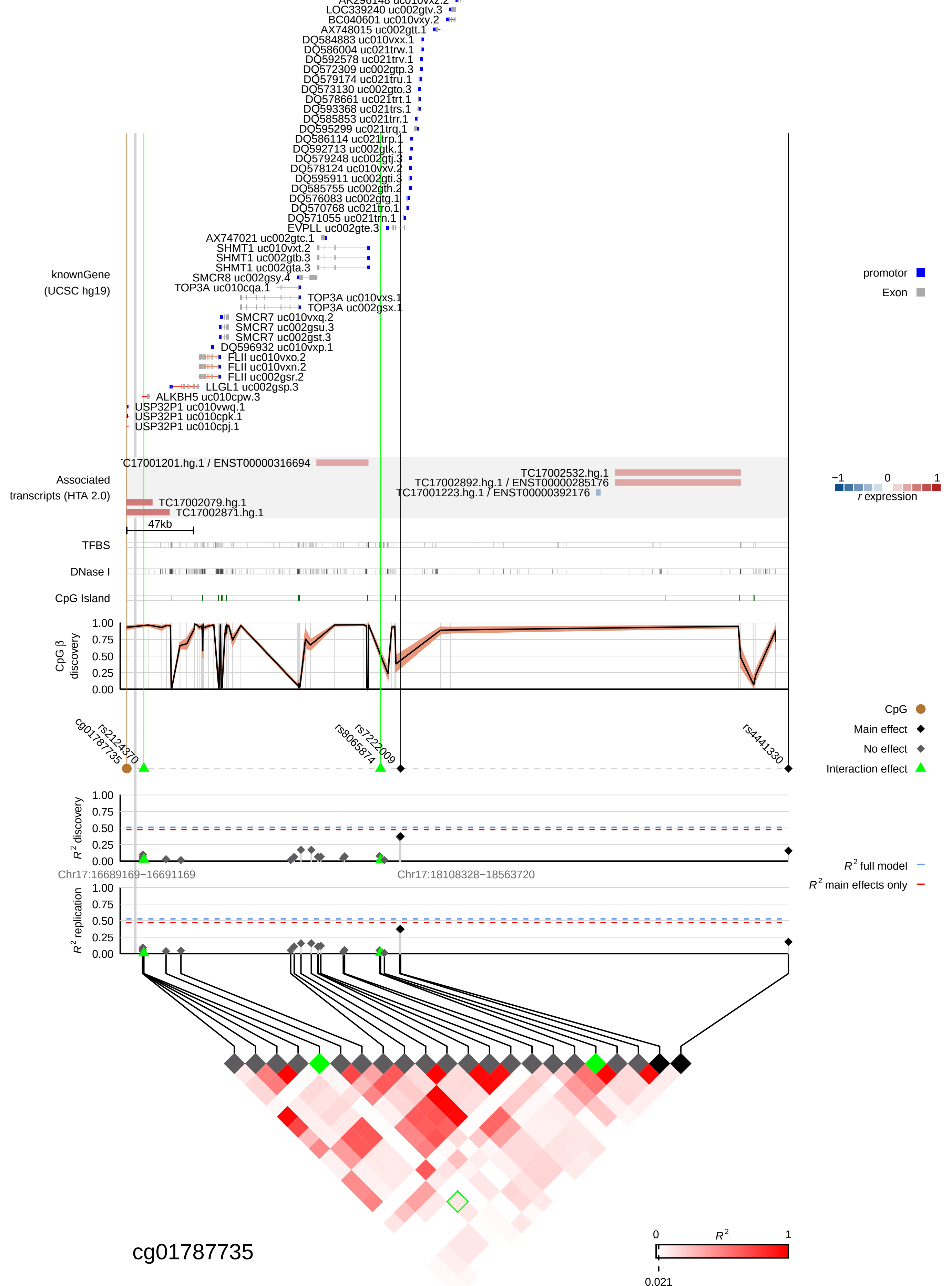

knownGene
(UCSC hg19)
promotor
Exon
Associated
transcripts (HTA 2.0)
TC17001201.hg.1 / ENST00000316694
TC17002532.hg.1
TC17002892.hg.1 / ENST00000285176
TC17001223.hg.1 / ENST00000392176
TC17002079.hg.1
TC17002871.hg.1
r expression
47kb
TFBS
DNase I
CpG Island
CpG β discovery
cg01787735
rs2124370
rs8065874
rs7222009
rs4441330
CpG
Main effect
No effect
Interaction effect
R2 discovery
R2 replication
Chr17:16689169−16691169
Chr17:18108328−18563720
R2 full model
R2 main effects only
0.021
cg01787735

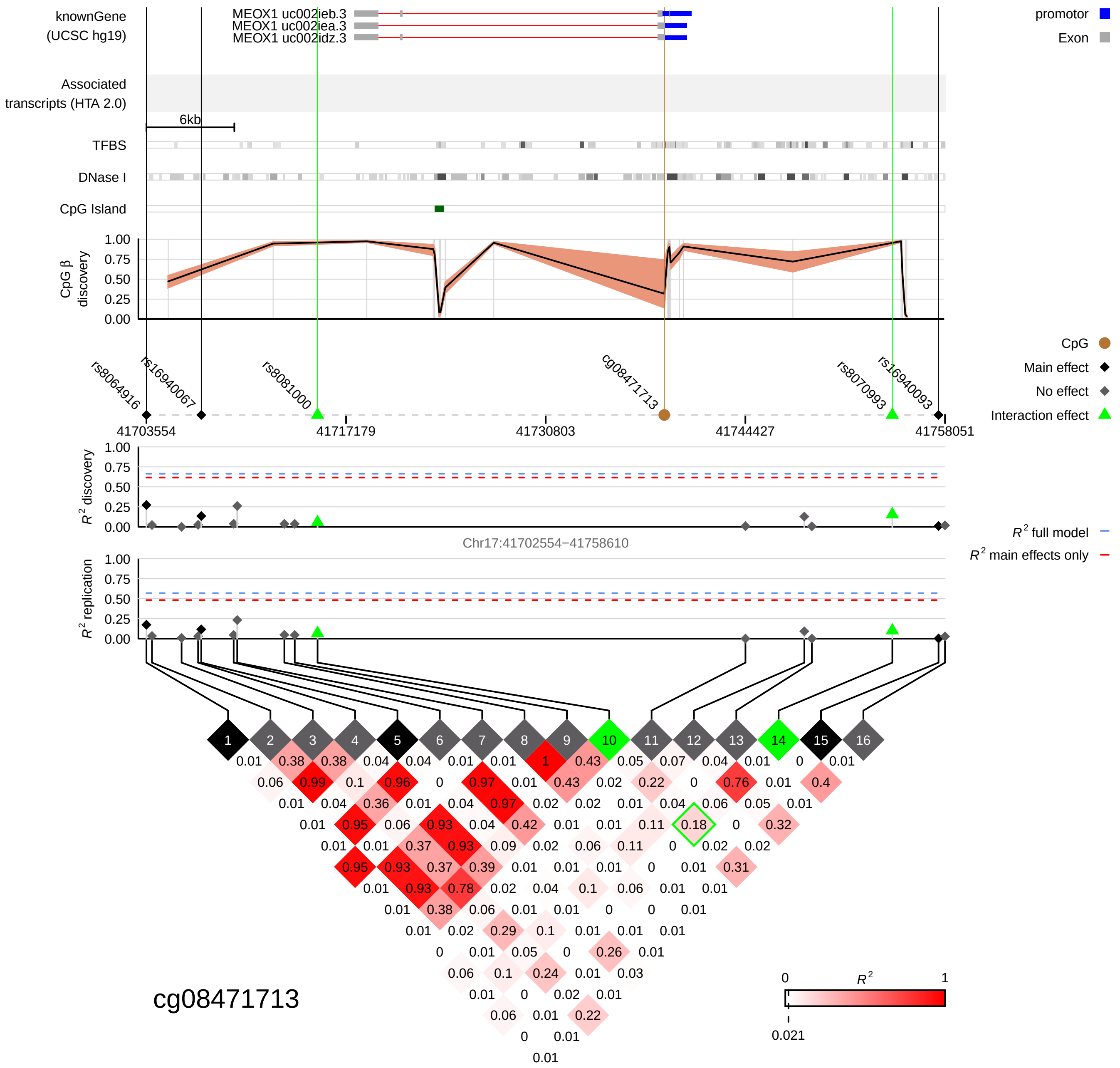
knownGene (UCSC hg19)
MEOX1 uc002ieb.3
MEOX1 uc002iea.3
MEOX1 uc002idz.3
promotor
Exon
Associated transcripts (HTA 2.0)
6kb
TFBS
DNase I
CpG Island
CpG β discovery
rs8064916
rs16940067
rs8081000
cg08471713
rs8070993
rs16940093
41703554
41717179
41730803
41744427
41758051
CpG
Main effect
No effect
Interaction effect
R^2 discovery
Chr17:41702554−41758610
R^2 full model
R^2 main effects only
R^2 replication
R^2
0.021
cg08471713

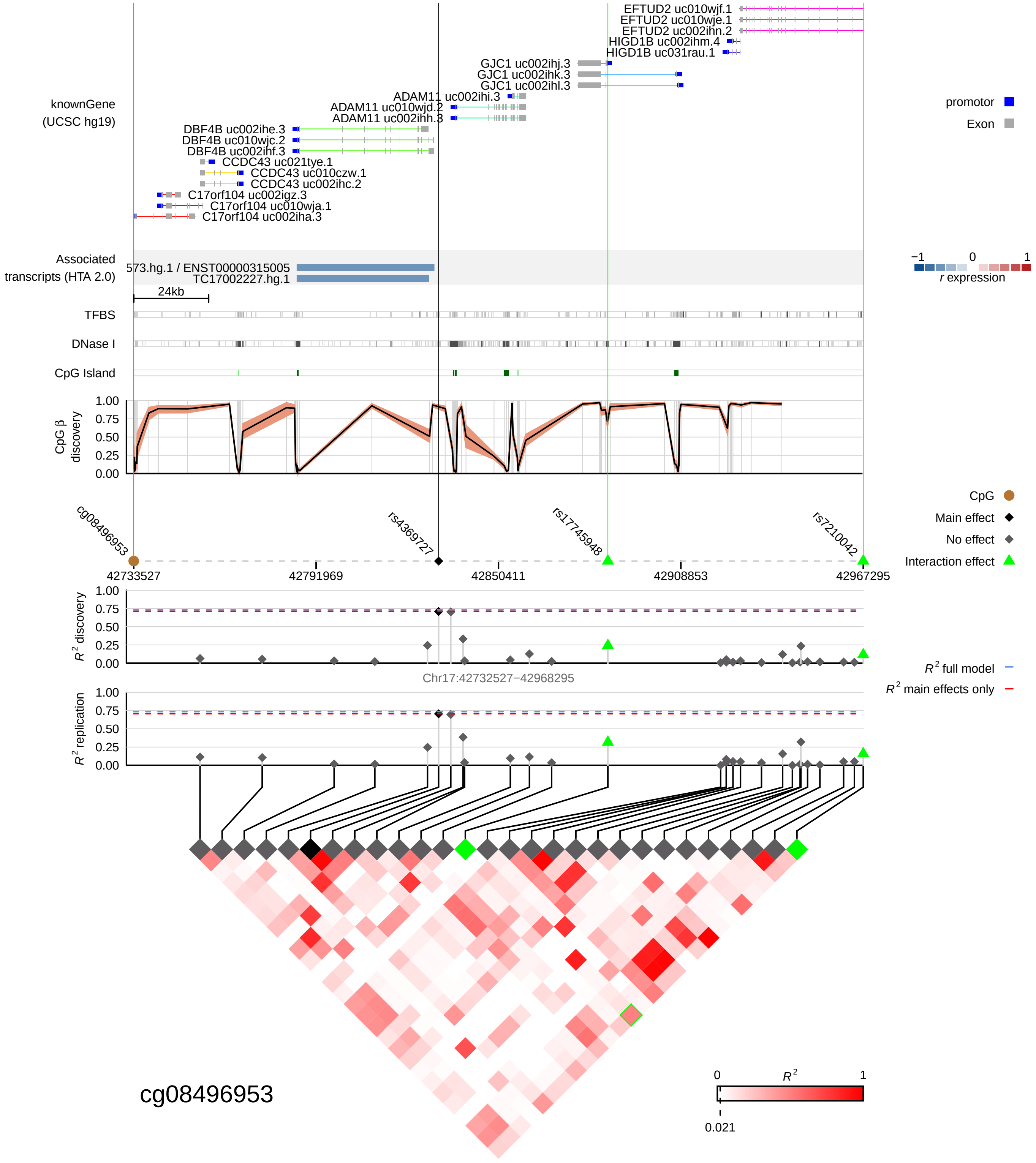
knownGene
(UCSC hg19)
EFTUD2 uc010wjf.1
EFTUD2 uc010wje.1
EFTUD2 uc002ihn.2
HIGD1B uc002ihm.4
HIGD1B uc031rau.1
GJC1 uc002ihj.3
GJC1 uc002ihk.3
GJC1 uc002ihl.3
ADAM11 uc002ihi.3
ADAM11 uc010wjd.2
ADAM11 uc002ihh.3
DBF4B uc002ihe.3
DBF4B uc010wjc.2
DBF4B uc002ihf.3
CCDC43 uc021tye.1
CCDC43 uc010czw.1
CCDC43 uc002ihc.2
C17orf104 uc002igz.3
C17orf104 uc010wja.1
C17orf104 uc002iha.3
promotor
Exon
Associated
transcripts (HTA 2.0)
573.hg.1 / ENST00000315005
TC17002227.hg.1
−1
0
1
r expression
24kb
TFBS
DNase I
CpG Island
CpG β
discovery
1.00
0.75
0.50
0.25
0.00
cg08496953
rs4369727
rs17745948
rs7210042
CpG
Main effect
No effect
Interaction effect
42733527
42791969
42850411
42908853
42967295
R² discovery
Chr17:42732527−42968295
R² full model
R² main effects only
R² replication
0
R²
1
0.021
cg08496953

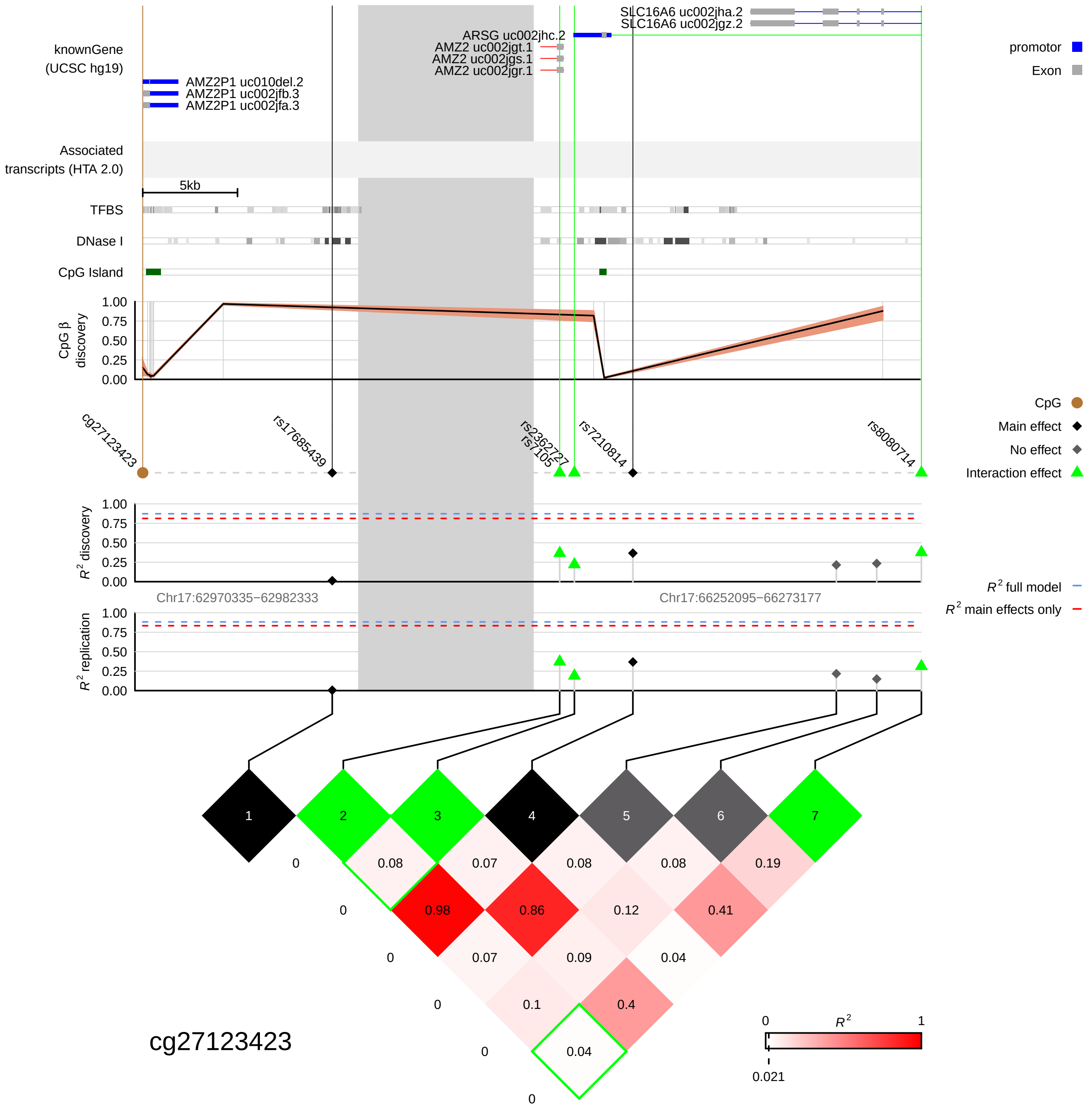

knownGene (UCSC hg19)
SLC16A6 uc002jha.2
SLC16A6 uc002jgz.2
ARSG uc002jhc.2
AMZ2 uc002jgt.1
AMZ2 uc002jgs.1
AMZ2 uc002jgr.1
AMZ2P1 uc010del.2
AMZ2P1 uc002jfb.3
AMZ2P1 uc002jfa.3
promotor
Exon
Associated transcripts (HTA 2.0)
5kb
TFBS
DNase I
CpG Island
CpG β discovery
1.00
0.75
0.50
0.25
0.00
cg27123423
rs17685439
rs2362727
rs7105
rs7210814
rs8080714
CpG
Main effect
No effect
Interaction effect
R² discovery
Chr17:62970335−62982333
Chr17:66252095−66273177
R² full model
R² main effects only
R² replication
1
2
3
4
5
6
7
0.08
0.07
0.08
0.08
0.19
0.98
0.86
0.12
0.41
0.07
0.09
0.04
0.1
0.4
0.04
0
R²
1
0.021
cg27123423

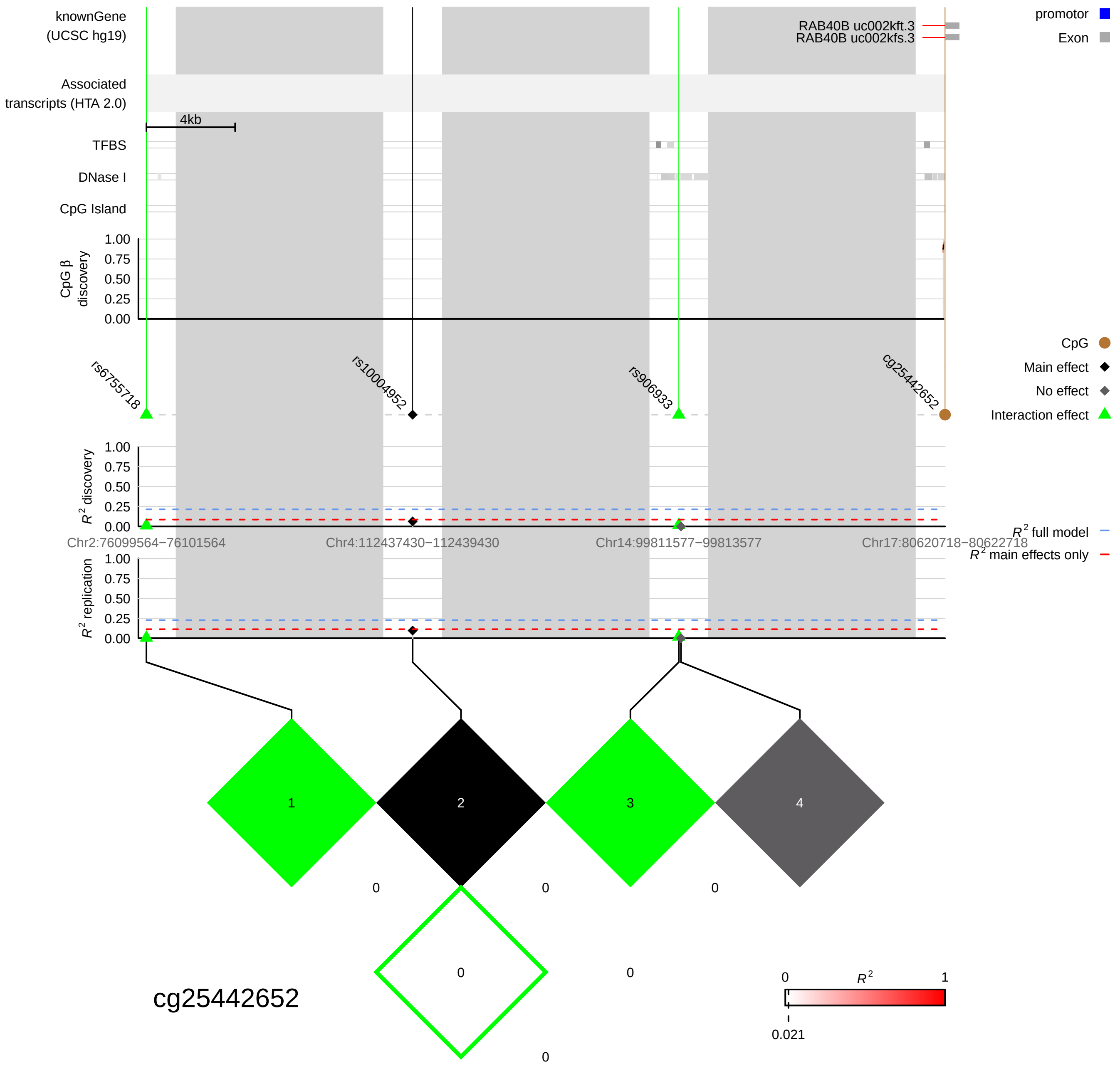
knownGene (UCSC hg19)
Associated transcripts (HTA 2.0)
4kb
TFBS
DNase I
CpG Island
CpG β discovery
RAB40B uc002kft.3
RAB40B uc002kfs.3
promotor
Exon
rs6755718
rs10004952
rs906933
cg25442652
CpG
Main effect
No effect
Interaction effect
R^2 discovery
R^2 replication
Chr2:76099564−76101564
Chr4:112437430−112439430
Chr14:99811577−99813577
Chr17:80620718−80622718
R^2 full model
R^2 main effects only
1.00
0.75
0.50
0.25
0.00
1
2
3
4
0
cg25442652
R^2
1
0.021

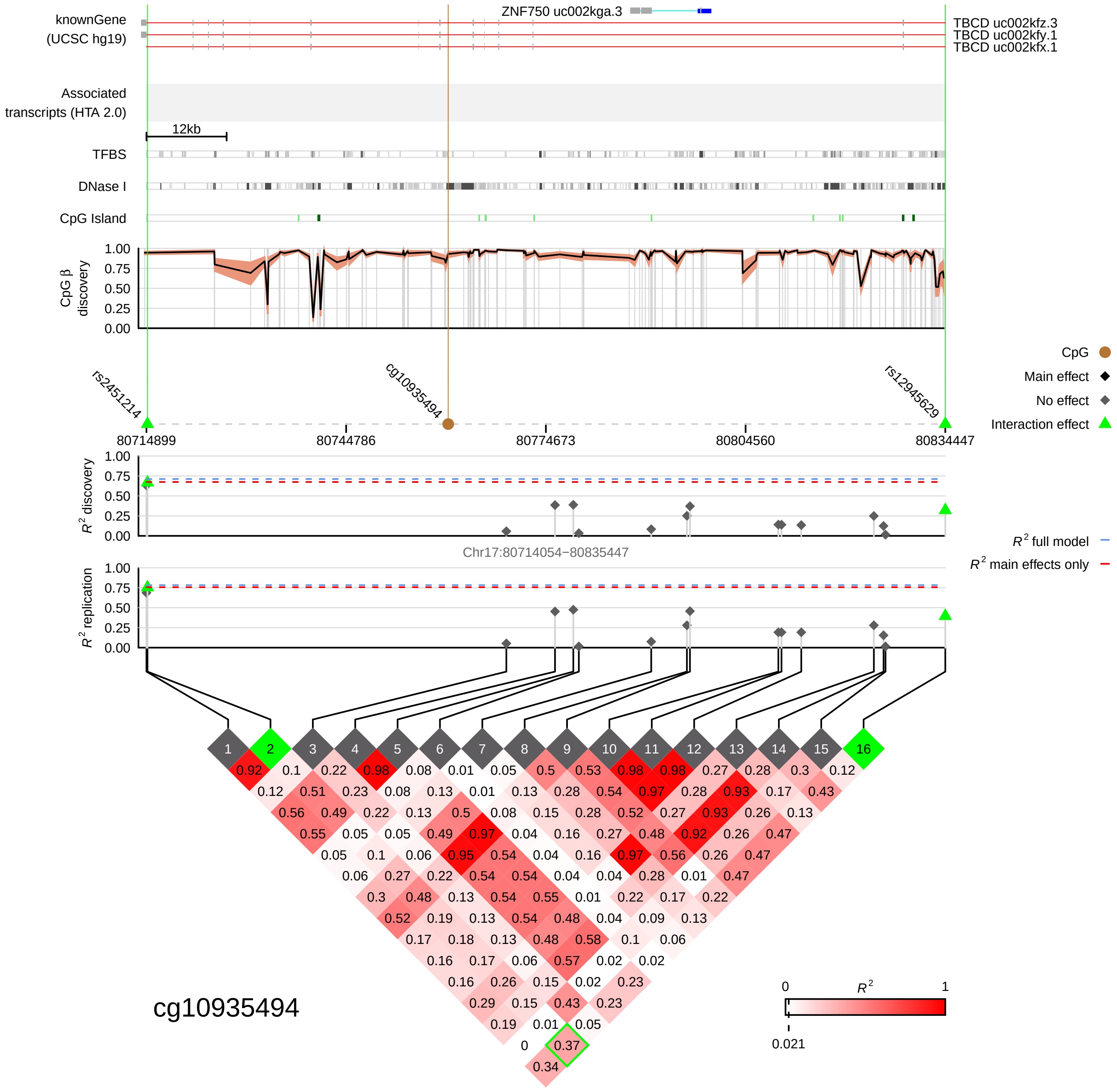

knownGene (UCSC hg19)
ZNF750 uc002kga.3
TBCD uc002kfz.3
TBCD uc002kfy.1
TBCD uc002kfx.1
Associated transcripts (HTA 2.0)
12kb
TFBS
DNase I
CpG Island
CpG β discovery
rs2451214
cg10935494
rs12945629
80714899
80744786
80774673
80804560
80834447
CpG
Main effect
No effect
Interaction effect
R^2 discovery
R^2 full model
R^2 main effects only
Chr17:80714054−80835447
R^2 replication
R^2
0.021
cg10935494

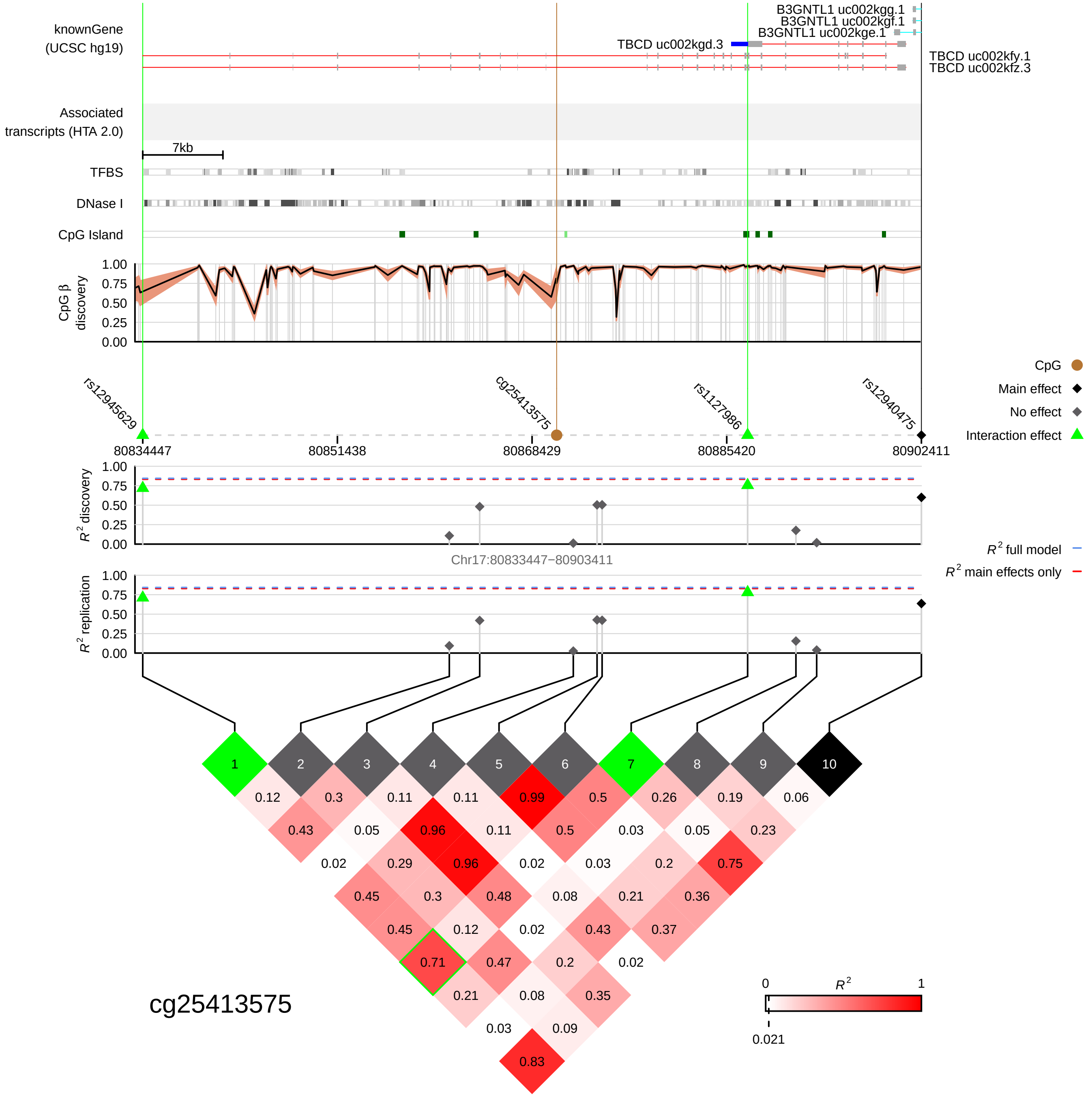
knownGene (UCSC hg19)
Associated transcripts (HTA 2.0)
TFBS
DNase I
CpG Island
CpG β discovery
B3GNTL1 uc002kgg.1
B3GNTL1 uc002kgf.1
B3GNTL1 uc002kge.1
TBCD uc002kgd.3
TBCD uc002kfy.1
TBCD uc002kfz.3
7kb
rs12945629
cg25413575
rs1127986
rs12940475
80834447
80851438
80868429
80885420
80902411
CpG
Main effect
No effect
Interaction effect
R^2 discovery
Chr17:80833447−80903411
R^2 full model
R^2 main effects only
R^2 replication
cg25413575
R^2
0.021

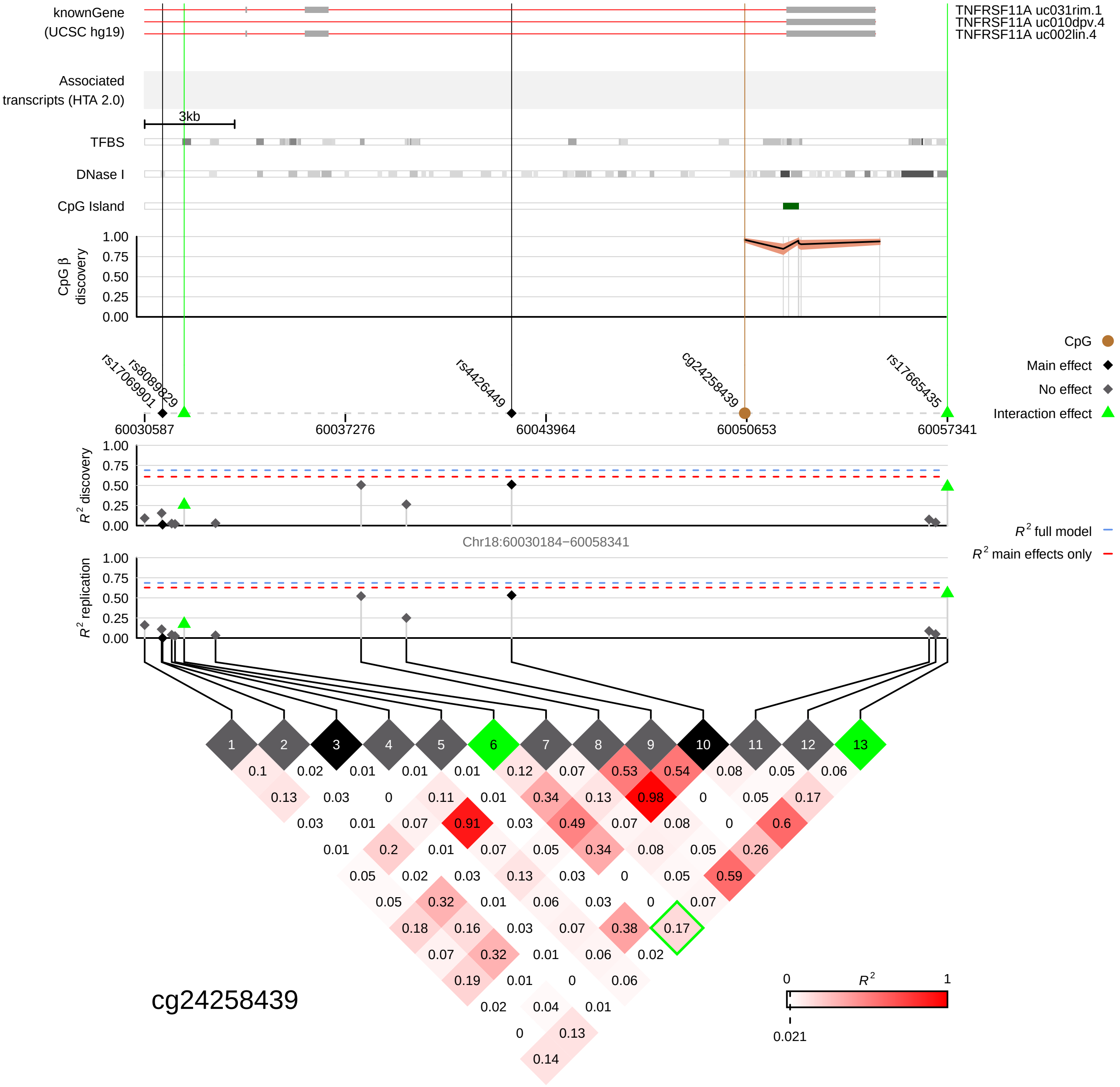

knownGene (UCSC hg19)
TNFRSF11A uc031rim.1
TNFRSF11A uc010dpv.4
TNFRSF11A uc002lin.4
Associated transcripts (HTA 2.0)
3kb
TFBS
DNase I
CpG Island
CpG β discovery
rs17069901
rs8089829
rs4426449
cg24258439
rs17665435
60030587
60037276
60043964
60050653
60057341
CpG
Main effect
No effect
Interaction effect
R^2 discovery
Chr18:60030184−60058341
R^2 full model
R^2 main effects only
R^2 replication
cg24258439
R^2
0
1
0.021

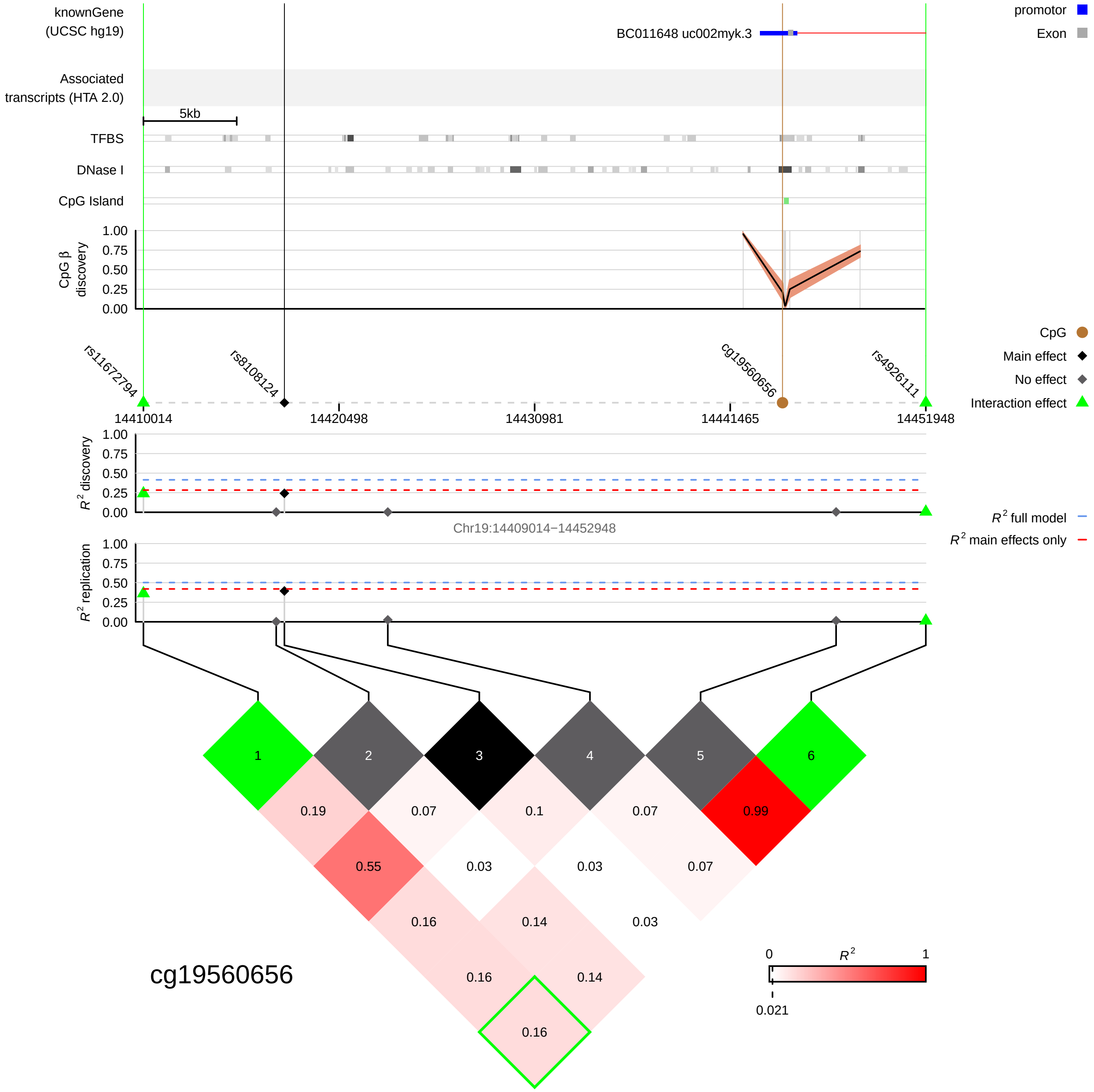

knownGene (UCSC hg19)
BC011648 uc002myk.3
promotor
Exon
Associated transcripts (HTA 2.0)
5kb
TFBS
DNase I
CpG Island
CpG β discovery
1.00
0.75
0.50
0.25
0.00
rs11672794
rs8108124
cg19560656
rs4926111
CpG
Main effect
No effect
Interaction effect
14410014
14420498
14430981
14441465
14451948
R² discovery
Chr19:14409014−14452948
R² full model
R² main effects only
R² replication
1
2
3
4
5
6
0.19
0.07
0.1
0.07
0.99
0.55
0.03
0.03
0.07
0.16
0.14
0.03
0.16
0.14
0.16
cg19560656
0
R²
1
0.021

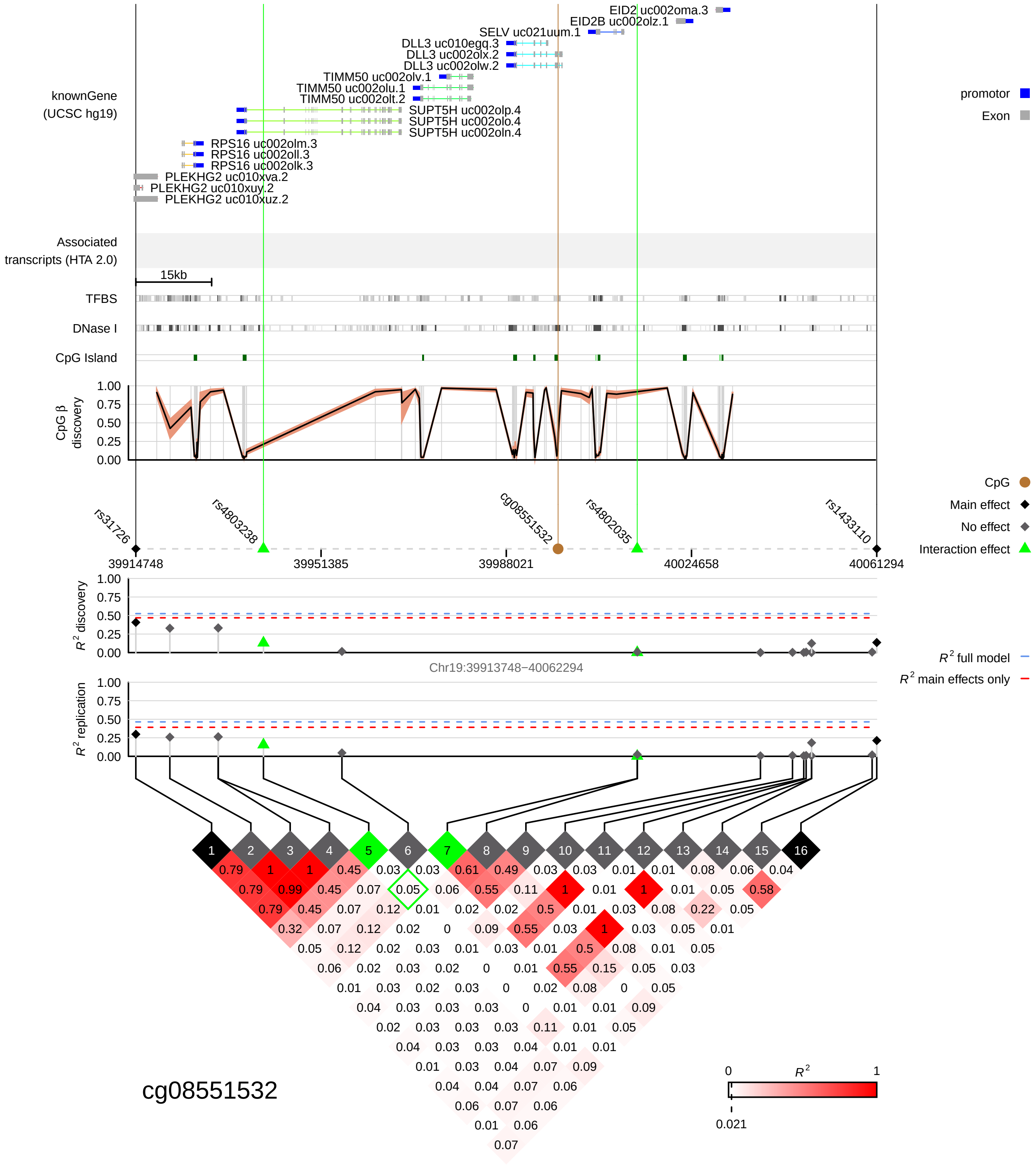

knownGene
(UCSC hg19)
EID2 uc002oma.3
EID2B uc002olz.1
SELV uc021uum.1
DLL3 uc010egq.3
DLL3 uc002olx.2
DLL3 uc002olw.2
TIMM50 uc002olv.1
TIMM50 uc002olu.1
TIMM50 uc002olt.2
SUPT5H uc002olp.4
SUPT5H uc002olo.4
SUPT5H uc002oln.4
RPS16 uc002olm.3
RPS16 uc002oll.3
RPS16 uc002olk.3
PLEKHG2 uc010xva.2
PLEKHG2 uc010xuy.2
PLEKHG2 uc010xuz.2
promotor
Exon
Associated transcripts (HTA 2.0)
15kb
TFBS
DNase I
CpG Island
CpG β discovery
rs31726
rs4803238
cg08551532
rs4802035
rs1433110
CpG
Main effect
No effect
Interaction effect
39914748
39951385
39988021
40024658
40061294
R² discovery
Chr19:39913748−40062294
R² full model
R² main effects only
R² replication
cg08551532
R²
0.021

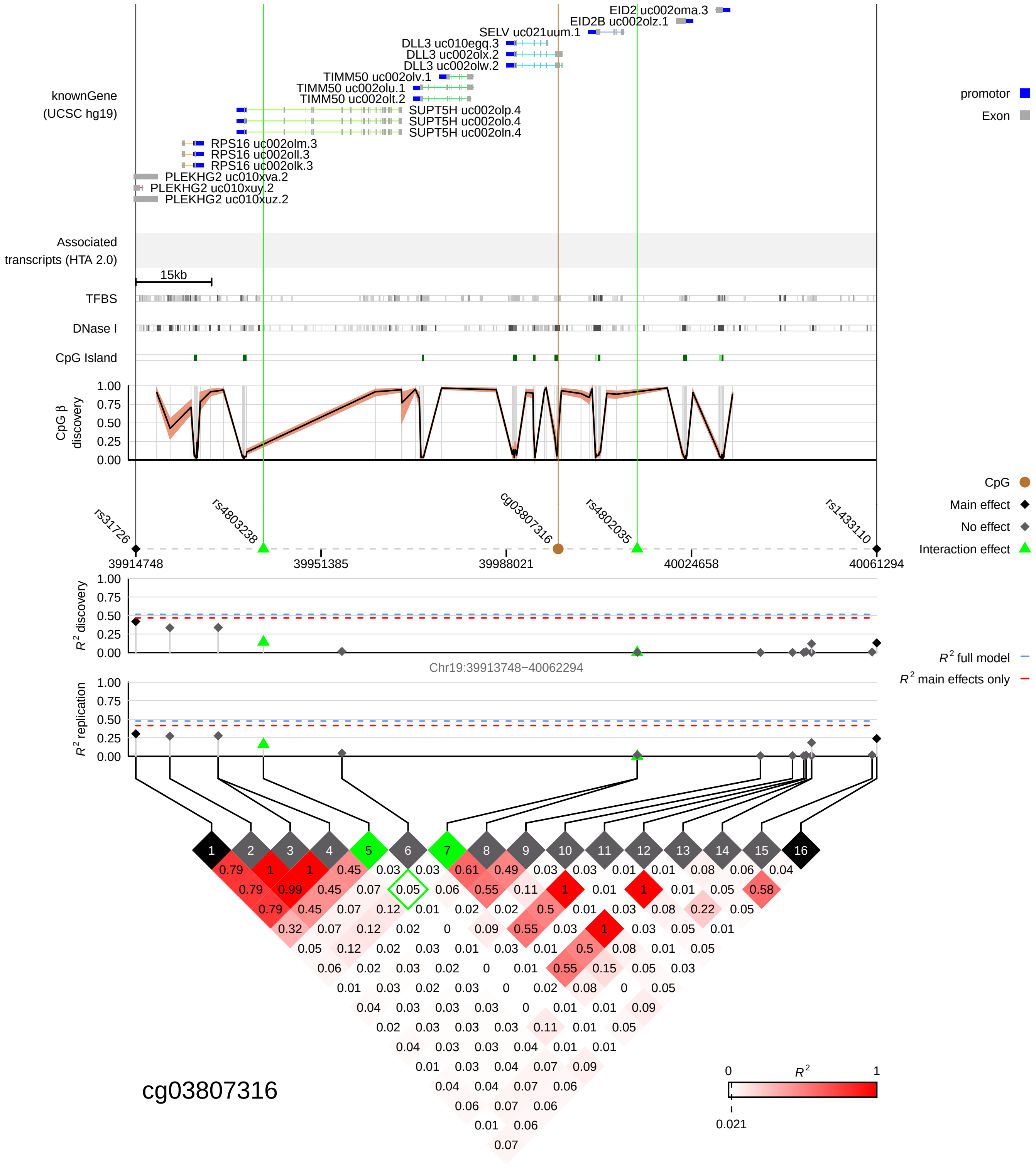
EID2 uc002oma.3
EID2B uc002olz.1
SELV uc021uum.1
DLL3 uc010egq.3
DLL3 uc002olx.2
DLL3 uc002olw.2
TIMM50 uc002olv.1
TIMM50 uc002olu.1
TIMM50 uc002olt.2
SUPT5H uc002olp.4
SUPT5H uc002olo.4
SUPT5H uc002oln.4
RPS16 uc002olm.3
RPS16 uc002oll.3
RPS16 uc002olk.3
PLEKHG2 uc010xva.2
PLEKHG2 uc010xuy.2
PLEKHG2 uc010xuz.2
knownGene
(UCSC hg19)
promotor
Exon
Associated
transcripts (HTA 2.0)
15kb
TFBS
DNase I
CpG Island
CpG β discovery
1.00
0.75
0.50
0.25
0.00
rs31726
rs4803238
cg03807316
rs4802035
rs1433110
39914748
39951385
39988021
40024658
40061294
CpG
Main effect
No effect
Interaction effect
R² discovery
Chr19:39913748−40062294
R² full model
R² main effects only
R² replication
1
2
3
4
5
6
7
8
9
10
11
12
13
14
15
16
0
R²
1
0.021
cg03807316

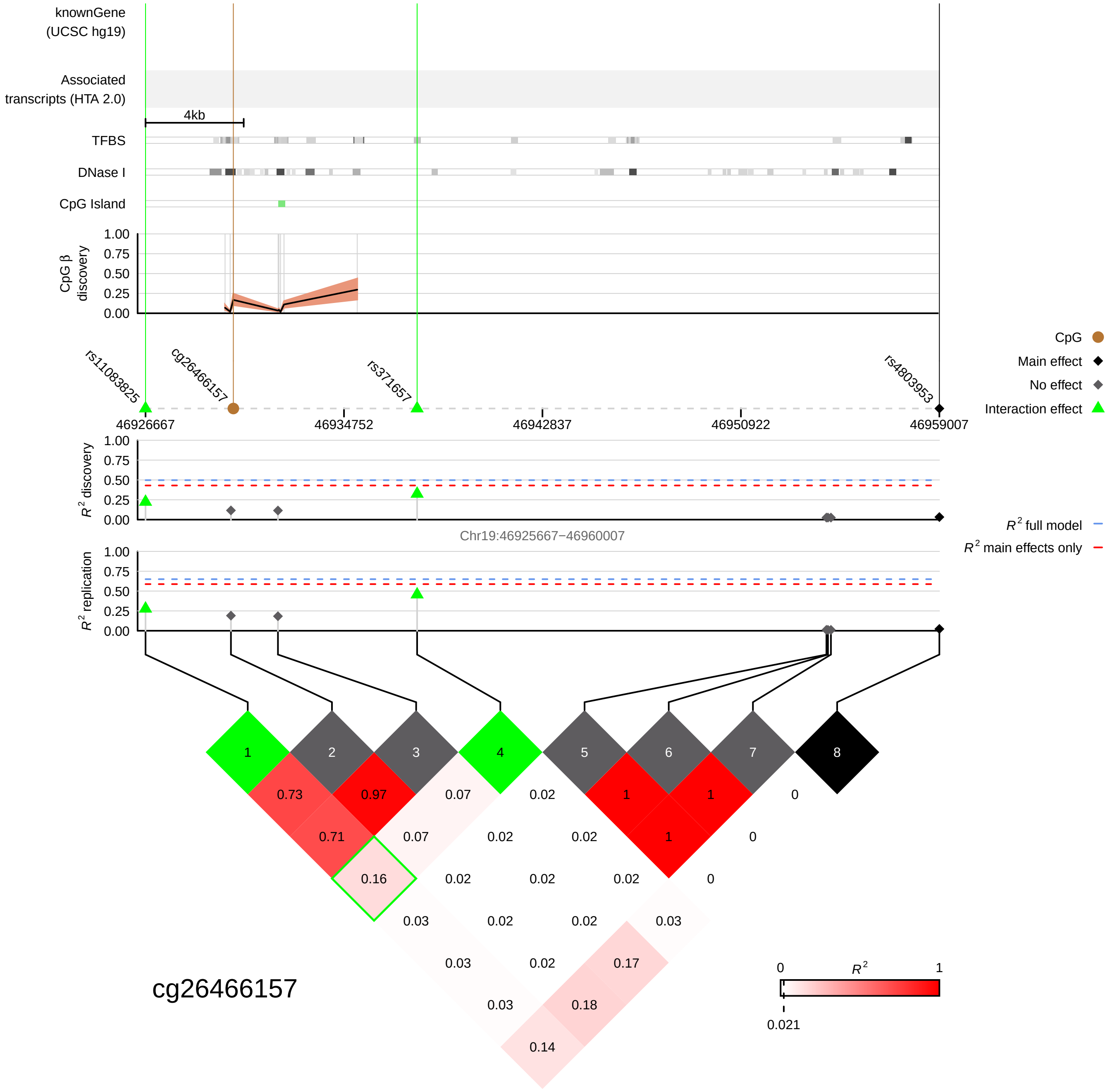

knownGene (UCSC hg19)
Associated transcripts (HTA 2.0)
4kb
TFBS
DNase I
CpG Island
CpG β discovery
rs11083825
cg26466157
rs371657
rs4803953
46926667
46934752
46942837
46950922
46959007
CpG
Main effect
No effect
Interaction effect
R^2 discovery
Chr19:46925667−46960007
R^2 full model
R^2 main effects only
R^2 replication
0.73
0.97
0.07
0.02
0.71
0.16
0.03
0.17
0.18
0.14
cg26466157
0.021

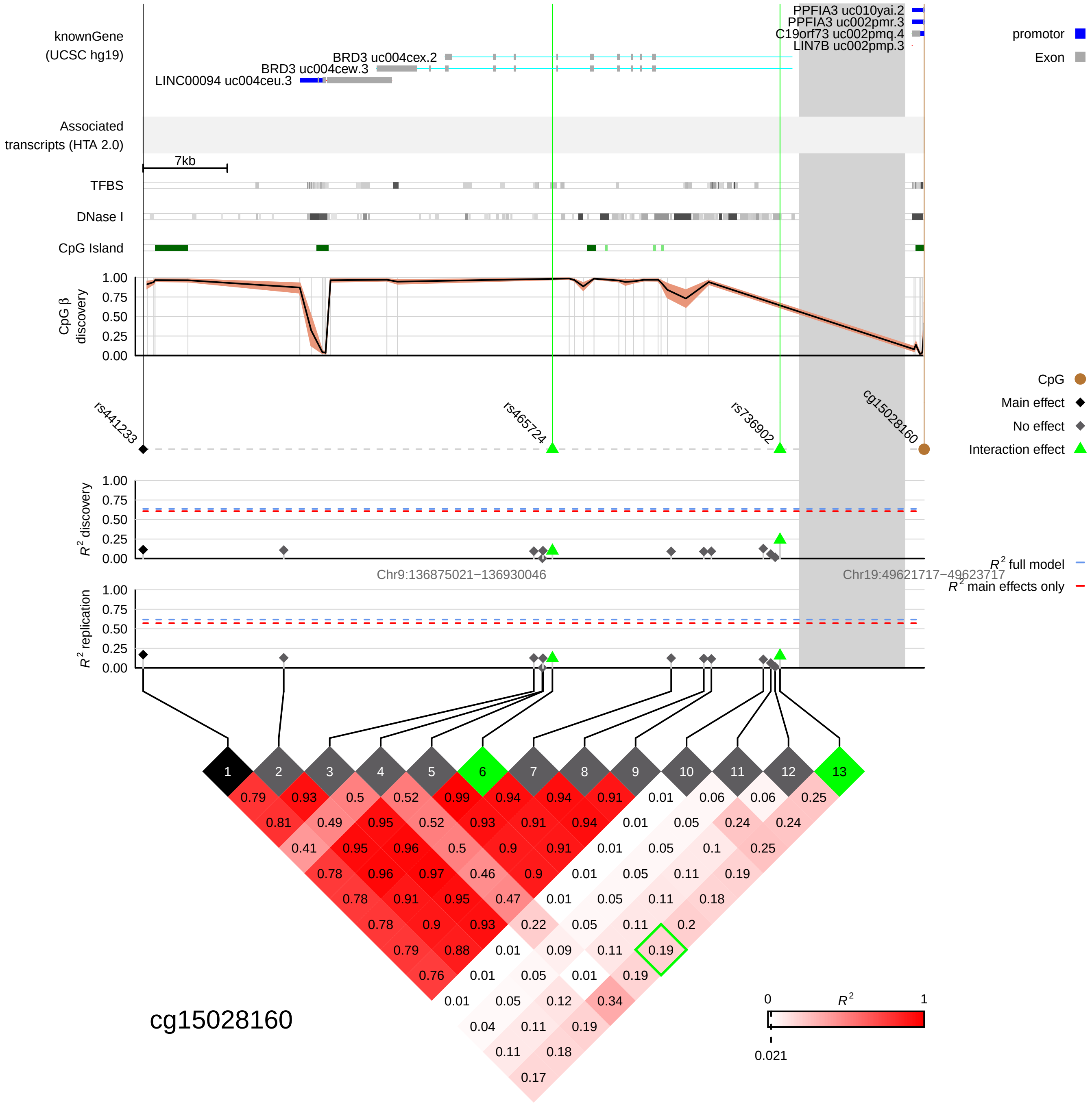

knownGene (UCSC hg19)
PPFIA3 uc010yai.2
PPFIA3 uc002pmr.3
C19orf73 uc002pmq.4
LIN7B uc002pmp.3
BRD3 uc004cex.2
BRD3 uc004cew.3
LINC00094 uc004ceu.3
promotor
Exon
Associated transcripts (HTA 2.0)
7kb
TFBS
DNase I
CpG Island
CpG β discovery
rs441233
rs465724
rs736902
cg15028160
CpG
Main effect
No effect
Interaction effect
R^2 discovery
Chr9:136875021−136930046
Chr19:49621717−49623717
R^2 full model
R^2 main effects only
R^2 replication
cg15028160
R^2
0.021

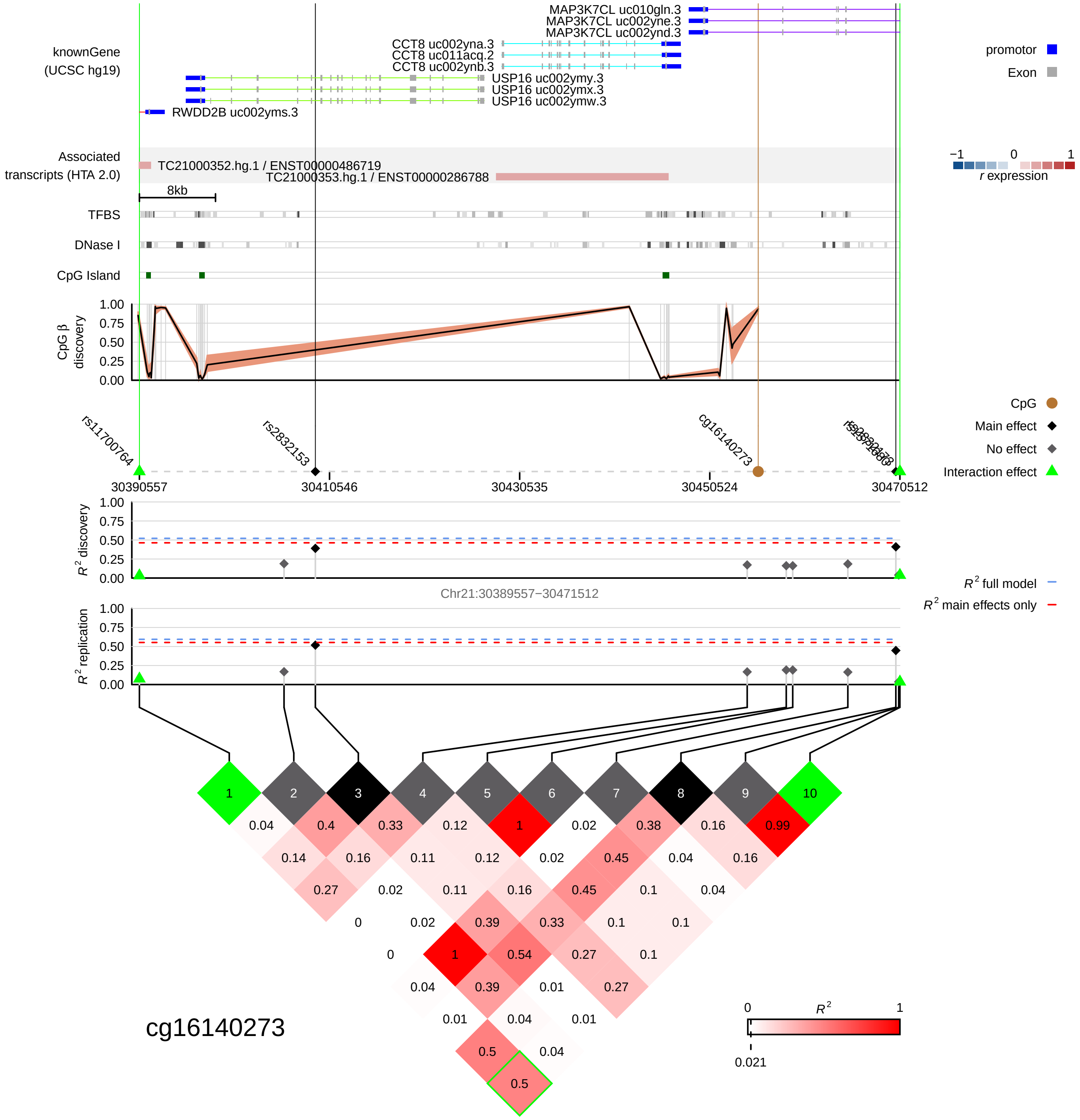
knownGene (UCSC hg19)
MAP3K7CL uc010gln.3
MAP3K7CL uc002yne.3
MAP3K7CL uc002ynd.3
CCT8 uc002yna.3
CCT8 uc011acq.2
CCT8 uc002ynb.3
USP16 uc002ymy.3
USP16 uc002ymx.3
USP16 uc002ymw.3
RWDD2B uc002yms.3
promotor
Exon
Associated transcripts (HTA 2.0)
TC21000352.hg.1 / ENST00000486719
TC21000353.hg.1 / ENST00000286788
r expression
8kb
TFBS
DNase I
CpG Island
CpG β discovery
rs11700764
rs2832153
cg16140273
CpG
Main effect
No effect
Interaction effect
30390557
30410546
30430535
30450524
30470512
R² discovery
Chr21:30389557−30471512
R² full model
R² main effects only
R² replication
0.021
cg16140273

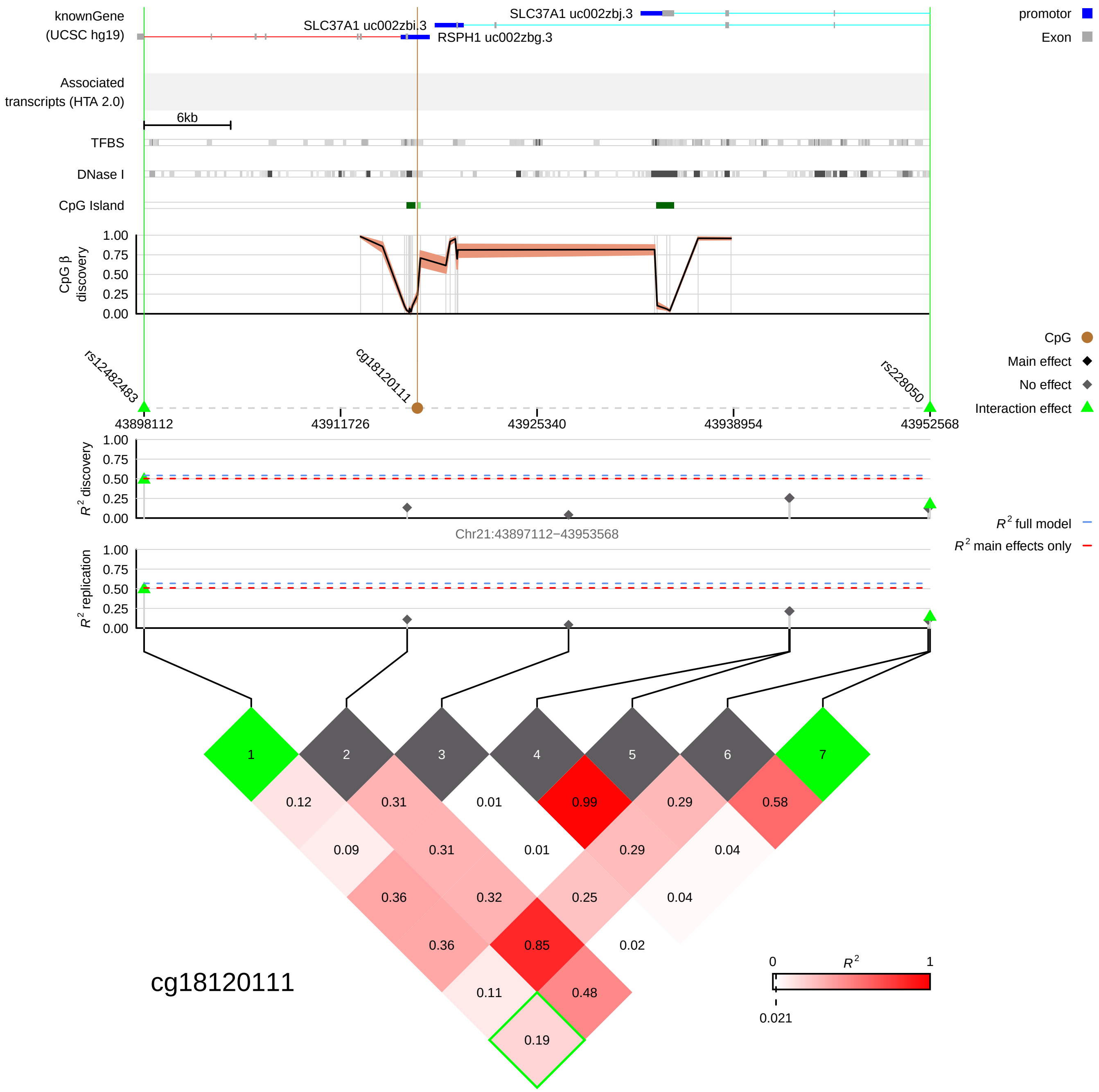
knownGene (UCSC hg19)
SLC37A1 uc002zbj.3
SLC37A1 uc002zbi.3
RSPH1 uc002zbg.3
promotor
Exon
Associated transcripts (HTA 2.0)
6kb
TFBS
DNase I
CpG Island
CpG β discovery
CpG
Main effect
No effect
Interaction effect
rs12482483
cg18120111
rs228050
43898112
43911726
43925340
43938954
43952568
R^2 discovery
Chr21:43897112−43953568
R^2 full model
R^2 main effects only
R^2 replication
cg18120111
0
R^2
1
0.021

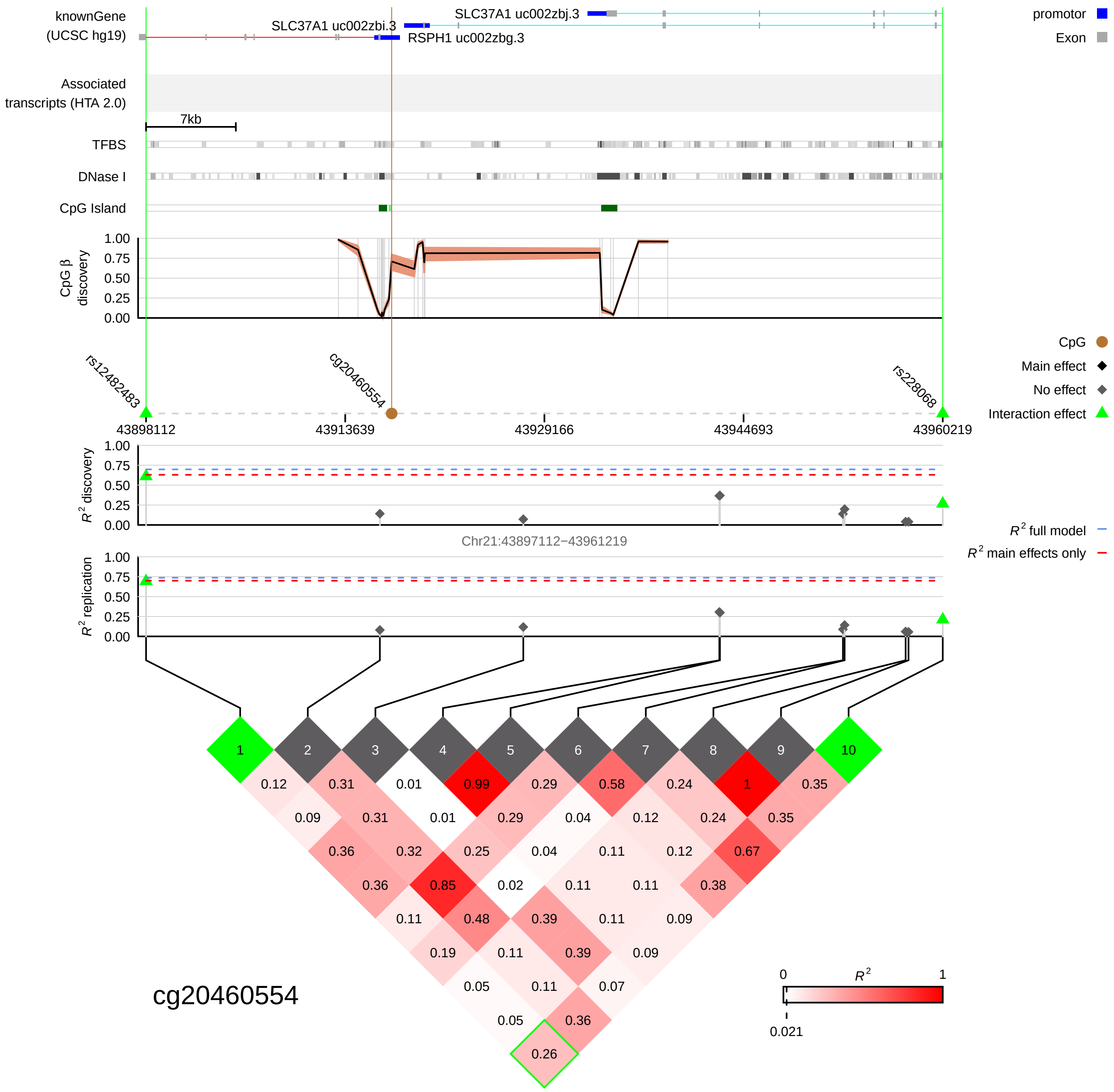
knownGene
(UCSC hg19)
SLC37A1 uc002zbj.3
SLC37A1 uc002zbi.3
RSPH1 uc002zbg.3
promotor
Exon
Associated
transcripts (HTA 2.0)
7kb
TFBS
DNase I
CpG Island
CpG β discovery
1.00
0.75
0.50
0.25
0.00
CpG
Main effect
No effect
Interaction effect
rs12482483
cg20460554
rs228068
43898112
43913639
43929166
43944693
43960219
R2 discovery
Chr21:43897112−43961219
R2 full model
R2 main effects only
R2 replication
1
2
3
4
5
6
7
8
9
10
0.12
0.31
0.01
0.99
0.29
0.58
0.24
1
0.35
0.09
0.31
0.01
0.29
0.04
0.12
0.24
0.35
0.36
0.32
0.25
0.04
0.11
0.12
0.67
0.36
0.85
0.02
0.11
0.11
0.38
0.11
0.48
0.39
0.11
0.09
0.19
0.11
0.39
0.09
0.05
0.11
0.07
0.05
0.36
0.26
cg20460554
0
R2
1
0.021
